# Insights into IrtAB: Iron Transport Facilitates Ultrasensitive Detection of *Mycobacteria* in Both Cellular and Clinical Environments

**DOI:** 10.1021/acscentsci.4c00676

**Published:** 2025-01-07

**Authors:** Dianmo Ni, Xiaoqiao Hong, Dingyi Liu, Xueyuan Li, Li Li, Wenwu Liu, Zhaogang Sun, Gang Liu

**Affiliations:** † School of Pharmaceutical Sciences, Tsinghua University, Haidian District, Beijing 100084, P. R. China; ‡ Translational Medicine Center, Beijing Chest Hospital, Capital Medical University, Tongzhou District, Beijing 101149, P. R. China; § Beijing Key Laboratory in Drug Resistant Tuberculosis Research, Beijing Tuberculosis & Thoracic Tumor Research Institute, Tongzhou District, Beijing 101149, P. R. China; ∥ Institute of Materia Medica, Chinese Academy of Medical Sciences & Peking Union Medical College, Xicheng District, Beijing 100050, P. R. China

## Abstract

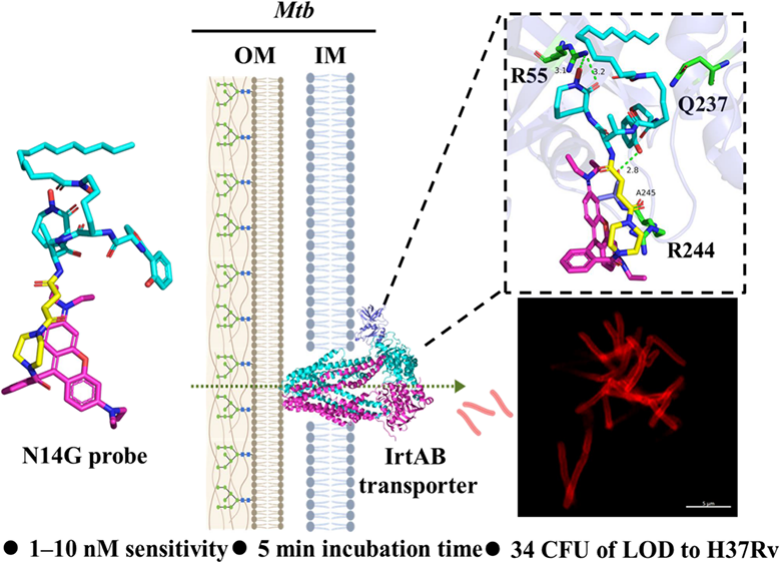

*Mycobacterium tuberculosis* (*Mtb*) utilizes a heterodimeric ABC transporter (IrtAB) to extract Fe^3+^ ions from host cells. This study demonstrates that ultrasensitive fluorescent probes, achieved through the conjugation of fluorophores with the ligand of IrtAB, N14G and N14G-Fe can detect *Mycobacterium smegmatis* at concentrations as low as 1–10 nM within an incubation period of less than 5 min. Furthermore, these probes effectively label *Mycobacterium bovis Bacille Calmette-Guérin* BCG and the wild-type *Mtb* strain H37Rv at a concentration of 0.1 μM after 10 min of incubation, achieving a limit of detection of 34 Colony-Forming Unit for the wild-type *Mtb* strain H37Rv. Both N14G and N14G-Fe successfully identified *Mtb* in sputum samples from patients diagnosed with tuberculosis, exhibiting exceptional fluorescence.

## Abstract 2

Siderophore-based fluorescent probes, such as N14G, have been developed for the detection of *mycobacteria* utilizing an active heterodimeric ABC transporter (IrtAB transporter). These probes exhibit ultrasensitivity with detection concentrations as low as 1−10 nM, yield results within a rapid 5 min incubation period, and possess a limit of detection of 34 CFU for the wild-type H37Rv strain of *Mycobacterium tuberculosis*. N14G and N14G-Fe are ultrasensitive fluorescent probes that utilize the IrtAB transporter for the specific detection of mycobacteria and the diagnosis of clinical sputum samples at concentrations ranging from 1 to 10 nM.

## Introduction

Bacteria require iron for survival and must acquire insoluble and trace amounts of ferric (Fe^3+^) ions from either host cells or their surrounding environment.^1,2^ Siderophores are molecules secreted by bacteria to facilitate the extracellular uptake of Fe^3+^ ions, typically exhibiting molecular weights ranging from 500 to 1,500 Da. Although over 500 siderophores have been identified, the structures of approximately 270 have been characterized.^1^

**Figure 1:**
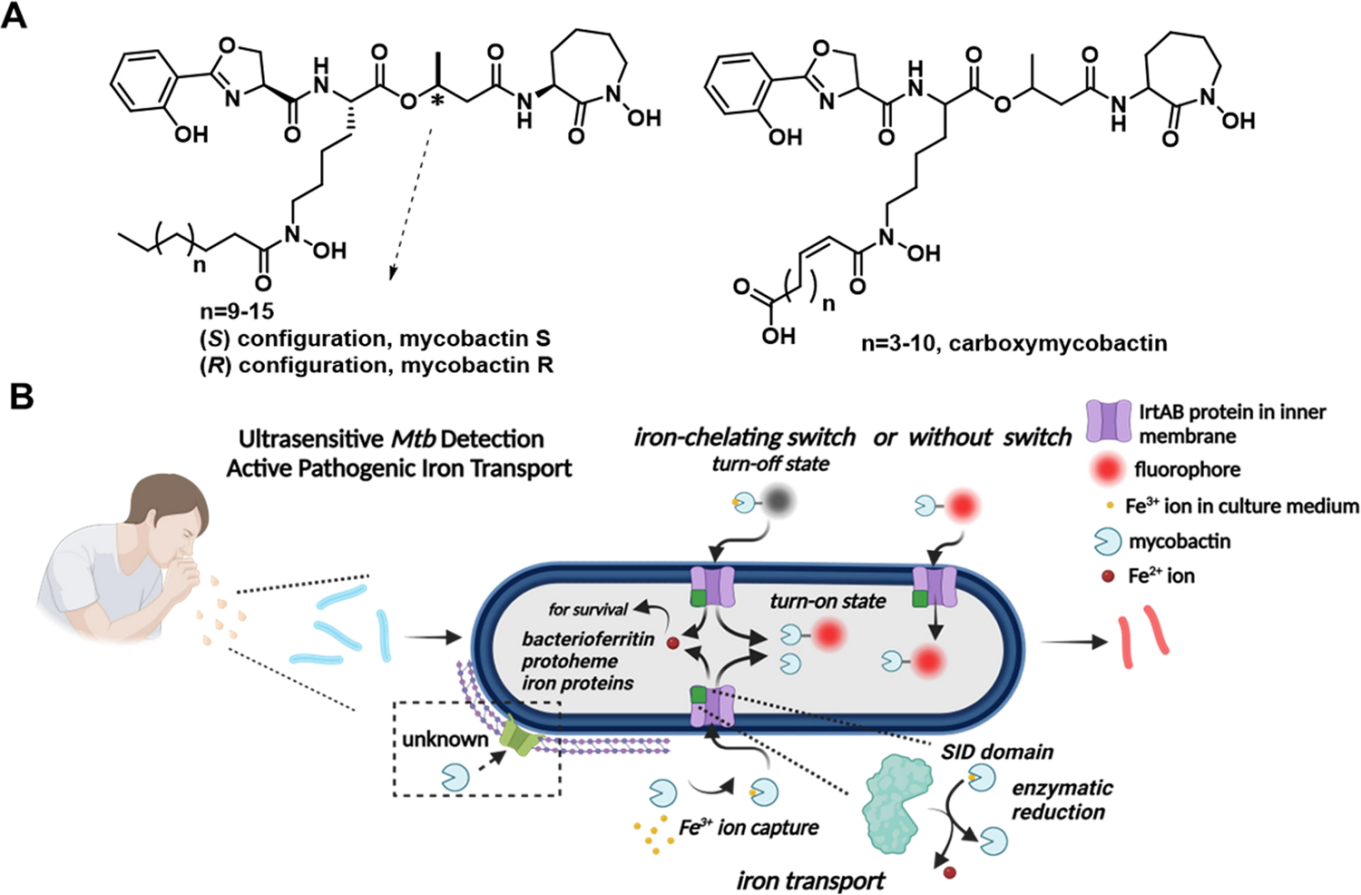
Transport of MbTFCp. (A) Structures of natural MbT *S*/*R* and cMbT. (B) Transport mechanism of probes N14G and N14G-Fe. The figure illustrates iron transport-based fluorescent probes designed for ultrasensitive detection of *mycobacteria*, both with and without an iron-chelating switch. MbT exhibits a high affinity for Fe^3+^ ions, forming the MbT-Fe complex, which is transported into *mycobacteria* through the inner membrane heterodimeric ABC transporter IrtAB, where Fe^3+^ ions are enzymatically reduced to Fe^2+^ ions in conjunction with the siderophore interaction domain. Both MbTFCps, regardless of iron chelation, are transported into *mycobacteria* via IrtAB. The fluorescence of MbTFCps containing the iron-chelating switch is quenched outside *mycobacteria* and reactivated when Fe^3+^ ions are reduced to Fe^2+^ ions and released within the bacteria.

*Mycobacterium tuberculosis* (*Mtb*), a highly virulent bacterial pathogen, is responsible for approximately 10.6 million new cases of tuberculosis (TB) and 1.3 million deaths annually.^3^*Mtb* utilizes carboxymycobactin (cMbT) and/or mycobactin (MbT) to scavenge Fe^3+^ ions from host cells,^4^ particularly within the phagosome of lung macrophages (Figure 1A). These ions are subsequently transported and reduced to soluble ferrous (Fe^2+^) ions for utilization. This process is mediated by the heterodimeric ABC transporter IrtAB, an inner membrane-anchored protein in *mycobacteria* (Figure 1B). cMbT and MbT both exhibit exceptionally high affinities for Fe^3+^ ions, enabling them to capture trace amounts of Fe^3+^ from host cells or the environment and form MbT-Fe and cMbT-Fe complexes for efficient transport. Recent studies indicate that the IrtAB protein in *Mycobacterium thermoresistibile* plays a critical role in iron uptake via MbT-Fe and cMbT-Fe, with a notable preference for MbT-Fe.^5^ As a conserved receptor for both MbT and cMbT, IrtAB represents a promising target for selectively and rapidly labeling *Mtb* through its unique active iron transport mechanism.^6^

To date, efforts to label Gram-negative bacteria using siderophore-fluorophore conjugates (SpFCs) have involved enterobactin, desferrioxamine, pyochelin, and biomimetic siderophores.^7−12^ Recently, a siderophore-conjugated chemiluminescent probe was developed for the detection of ESKAPE pathogens including *Enterococcus faecium*, *Staphylococcus aureus*, *Klebsiella pneumoniae*, *Acinetobacter baumannii*, *Pseudomonas aeruginosa*, and *Enterobacter spp*. utilizing an enzyme-activated switch.^13^ These studies have demonstrated that SpFCs are effective for pathogen detection. However, prior research on Gram-negative bacteria necessitated high probe concentrations (10–100 μM) and prolonged incubation time (1–12 h), which limited their clinical applicability. Notably, there is currently no research investigating the use of siderophore-based probes for detecting pathogenic *Mtb*.

Given the high incidence and mortality rates associated with TB caused by *Mtb*, which is characterized by slow replication, there remains a continuous demand for novel fluorescent probes capable of highly sensitive detection of *Mtb* cells or even single *Mtb* cell. Previous probes have relied on various mechanisms, including enzyme-sensitive methods,^14−19^ metabolism-dependent approaches,^20−26^ aggregation-induced emission mechanisms,^27^ and chemiluminescent proteases.^28^ These labeling methods utilize specific fluorescence off-to-on mechanisms, offering advantages over traditional staining methods such as auramine O (AO) and Ziehl-Neelsen stain that target mycolic acids in the *Mtb* cell wall while involving labor-intensive procedures to fix and remove excess dyes.^29^ Recently, Benjamin *et al*. introduced a more sensitive trehalose-based fluorogenic probe, RMR-Tre, for the detection of *mycobacteria*.^24^ However, many developed methods exhibit drawbacks including time-consuming processes (e.g., >1 h for slow enzymatic reactions or metabolic accumulation), high probe concentrations (e.g., 10 to 100 μM for weak fluorescence intensity), and chemical or photostability issues when used to stain clinical sputum samples from TB patients. The prolonged processing time associated with enzymatic or metabolic accumulation probes can be attributed to the slow replication and growth of pathogenic *Mtb*. Additionally, the unique lipid-rich cell wall of *Mtb* contributes to resistance against exogenous probe entry and upregulation of efflux mechanisms.^30^ Consequently, leveraging active transport mechanisms, i.e., IrtAB, for fluorescent probes is regarded as a promising strategy for diagnosing *Mtb*.

In this study, we drew inspiration from MbT and cMbT to develop a novel mycobactin-fluorophore conjugated probe (MbTFCp), designated N14G, which specifically targets the IrtAB-mediated iron transport pathway in *mycobacteria*. We demonstrate that N14G exhibits exceptional sensitivity for the selective detection of *mycobacteria*, with a probe concentration range of 1.0–10 nM and a rapid response time of less than 5 min for *M. smegmatis* detection. Furthermore, the fluorescence of N14G-Fe, which chelates Fe^3+^ ions capable of quenching fluorescence in the external environment,^31^ is effectively quenched; however, it is believed that the fluorescence is reactivated within *mycobacteria* following the reduction and release of Fe^2+^ ions (Figure 1B).

## Results and Discussion

### Design and Synthesis of MbTFCps

**Figure 2:**
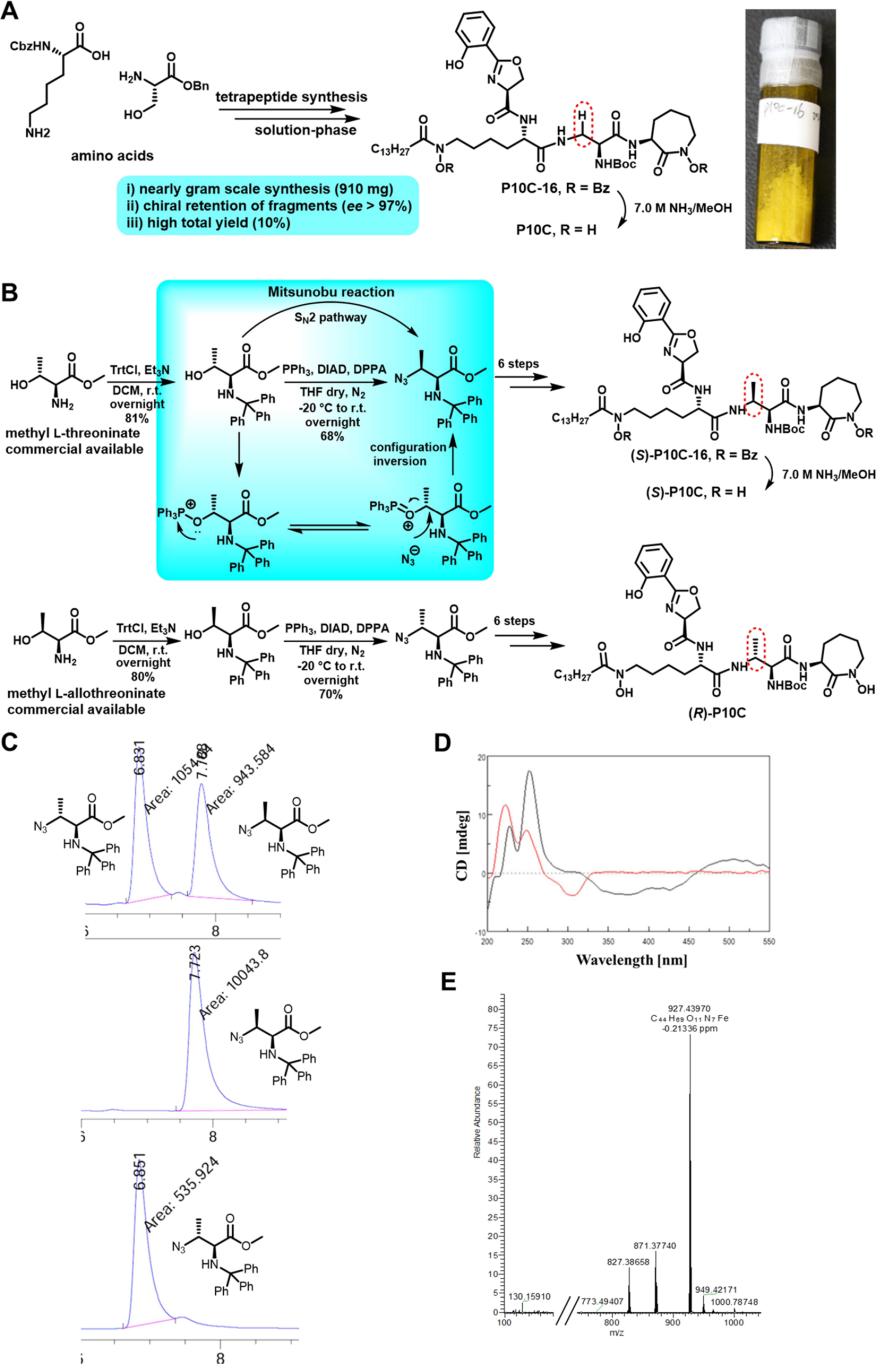
Scalable synthesis and characterization of MbT analogues. (A) Scalable synthesis of P10C-16. (B) Synthesis of chiral methyl building blocks, with (*S*)-P10C and (*R*)-P10C highlighted in red circles. (C) High-performance liquid chromatography chiral analysis of the methyl building blocks, showing retention times of 7.7 min for the (*S*-) configuration and 6.8 min for the (*R*-) configuration, respectively. (D) CD spectra of P10C (red) and the P10C-Fe complex (black). (E) HRMS (ESI-MS) *m*/*z* for P10C-Fe [M + H]^+^; Calcd for C_44_H_69_FeN_7_O_11_ 927.4399; Found 927.4397.

To investigate the efficient transport of fluorophore-labeled probes utilizing MbT, we developed a scalable synthesis for P10C-16, a benzoyl (Bz)-protected MbT derivative (Figure 2A), achieving an overall yield of approximately 10% (∼gram level).^32−34^ P10C was obtained by completely removing the Bz protection from P10C-16 in the presence of 7.0 M NH_3_/MeOH. To facilitate the scaled-up synthesis of P10C, we prepared the requisite chiral pure building blocks A2 and BocA7 (Figures S1 and S2). Commercially available chiral pure substrates, methyl l-threoninate and methyl l-allothreoninate, underwent Mitsunobu reaction, resulting in complete conversion to their corresponding amino acid derivatives (Figure 2B).^35^ The products exhibited high chiral purity with enantiomeric excess values exceeding 99% (Figure 2C) and were subsequently converted into Bz/*tert*-butoxycarbonyl (Boc)-protected forms: (*S*)-P10C-16 (Figure 2B) and (*R*)-P10C-16 (Figure S3). Following Bz removal using 7.0 M NH_3_/MeOH, Boc-protected forms of both enantiomers, (*S*)-P10C and (*R*)-P10C, were obtained as methyl MbT analogues known to be secreted by *Mtb* or nontuberculous mycobacteria (NTM), respectively.^36^ Synthetic schemes are illustrated in Schemes S1–S12. To confirm the ability of P10C to chelate iron, we mixed P10C with FeCl_3_ in a molar ratio of 1:2 in MeOH and purified the resultant product using a reverse-phase C18 column. This yielded the P10C–Fe complex as a brown powder, consistent with typical iron chelate color (Figure S4). Circular dichroism (CD) studies on both P10C and P10C–Fe confirmed successful iron chelation, evidenced by positive and negative Cotton effects observed within the range of 325–550 nm (Figure 2D). High-resolution mass spectrometry (HRMS) further validated the accurate molecular weight of the P10C–Fe complex as shown in Figures 2E and S4.

**Figure 3:**
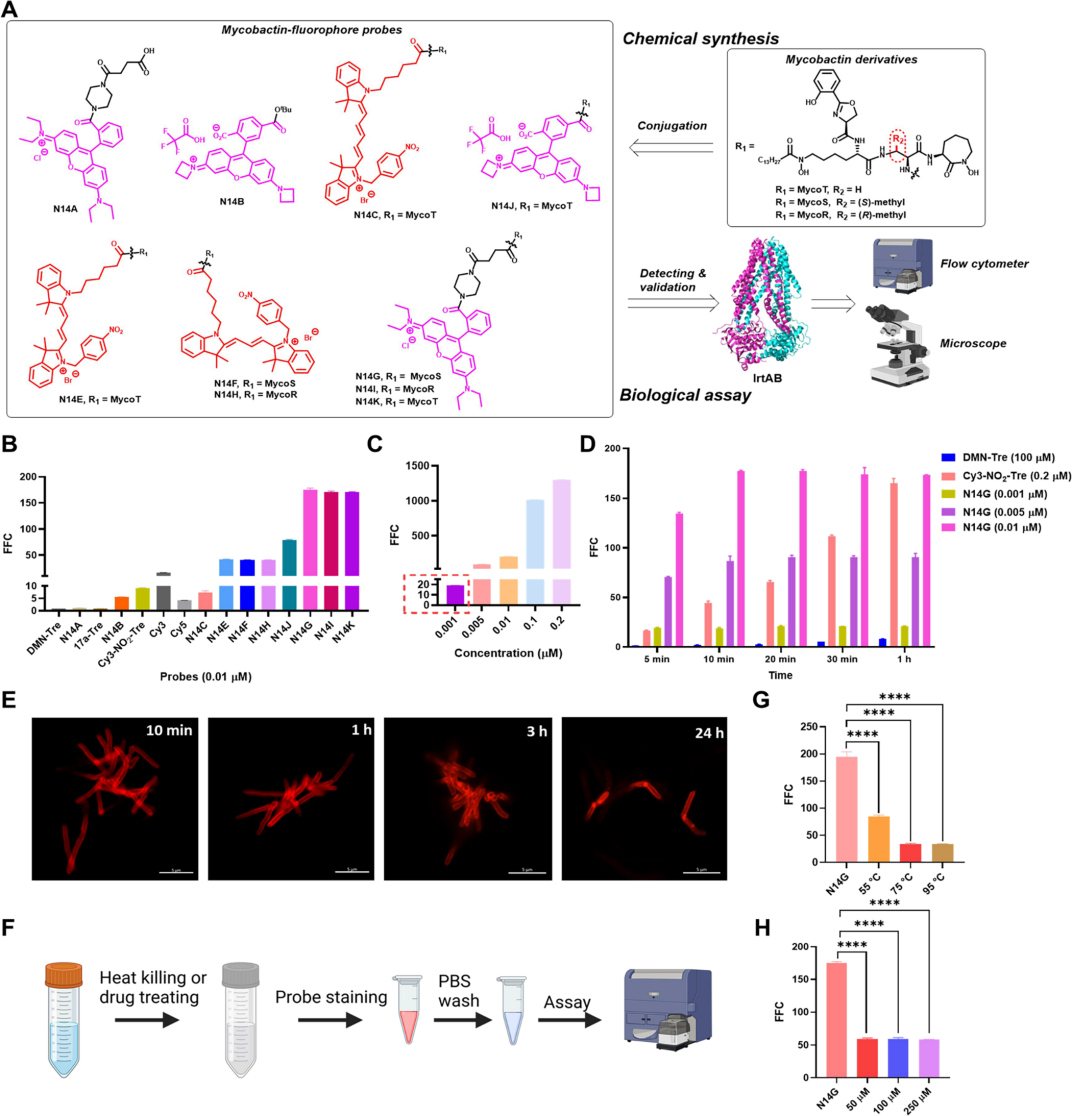
Characterization of MbTFCps. (A) Chemical structures of synthesized MbT derivatives, employed fluorophores, and their corresponding MbTFCps. (B) Flow cytometry analysis of *M. smegmatis* incubated with various probes at a concentration of 0.01 μM for 1 h. (C) Flow cytometry analysis of *M. smegmatis* incubated with N14G at different concentrations (0.001 μM, 0.005 μM, 0.01 μM, 0.1 μM, and 0.2 μM) for 1 h. (D) Flow cytometry analysis of *M. smegmatis* incubated with DMN-Tre (100 μM), Cy3-NO_2_-Tre (0.2 μM), and N14G (0.001 μM, 0.005 μM, and 0.01 μM) over various incubation times (5 min, 10 min, 20 min, 30 min, and 1 h). (E) Confocal images of live *M. smegmatis* labeled with N14G (0.01 μM) over different times (10 min, 30 min, 3 h, and 24 h). Scale bars, 5 μm. (F) Protocol for labeling *M. smegmatis* using N14G (0.01 μM) following heat-killing or anti-TB drug treatment. (G) Flow cytometry analysis of *M. smegmatis* pretreated by heat-killing at temperatures of 95 °C, 75 °C, and 55 °C for 30 min followed by incubation with N14G (0.01 μM) for additional 1 h. (H) Flow cytometry analysis of *M. smegmatis* pretreated with BTZ043 (50 μM, 100 μM, and 250 μM) for 5 h followed by incubation with N14G (0.01 μM) for additional 1 h.

The fluorescent pharmacophores selected for conjugation with MbT include cyanine and rhodamine derivatives, highlighted in red and magenta in Figure 3A, respectively. The MbT-fluorophore conjugates N14C, N14E, N14F, and N14H are nitroreductase-based cyanine analogs featuring a fluorescence off-to-on switch. In contrast, the compounds N14G, N14I, N14J, and N14K are rhodamine derivatives. The excitation and emission spectra of these final conjugates are detailed in Figure S5. Control probes consist of previously reported nitroreductase-based fluorescent molecules: 17a-Tre (a coumarin derivative,^37^Figure S6), Cy3-NO_2_-Tre (a cyanine derivative,^19^Figure S6), and DMN-Tre (a dimethylamino-1,8-naphthalimide derivative,^38^Figure S6), all structurally linked to trehalose. In *Mtb*, trehalose is specifically transported into the cytoplasm via the SugABC-LpqY transporter located in the inner membrane. Under the action of polyketide synthase 13 (Pks13), trehalose undergoes mycobacterial acidification resulting in the production of trehalose monomycolate, which is subsequently transported out of the *Mtb* cytoplasm and incorporated into its cell wall. Therefore, trehalose-based probes can ultimately be integrated onto the cell wall of *mycobacteria* for specific labeling of *Mtb* trehalose.^39^ Additionally, Cy3 and Cy5 cyanine probes without MbT conjugation were tested as control probes (Figure S6). Rhodamine derivatives N14A and N14B, lacking MbT conjugation, were included to verify the role of MbT in transport and labeling by MbTFCps within *Mtb*.^40,41^ Iron-limiting medium was used to culture bacteria for more observed fluorescence intensity than in normal medium, which may result from bacterial response to iron-deficiency environment.^7^

### Ultrasensitive Labeling of *Mycobacterium smegmatis* and *Mycobacterium bovis* Bacille Calmette-Guérin by MbTFCps

The efficacy of MbTFCps in labeling *M. smegmatis* was evaluated (Figures 3B–C and S7). All tested probes demonstrated concentration-dependent labeling from 1.0 nM to 0.2 μM over a duration of 1 h. Among these, N14G, N14I, and N14K (MbT-rhodamine probes) exhibited the highest fluorescence intensity, with N14G achieving approximately a 19-fold fluorescence fold change (FFC) at 1.0 nM and a 134-fold FFC at 10 nM within just 5 min (Figure 3D). In contrast, N14C (an MbT-cyanine probe) displayed the lowest fluorescence intensity among the MbTFCps (Figures 3B–C and S7). Another rhodamine-based probe, N14J, showed lower FFC compared to N14G, N14I, and N14K, suggesting that the piperazine-succinic linker influences performance. Consequently, N14L was synthesized; however, its FFC in *M. smegmatis* remained inferior to that of N14G (Figures S8 and S9). Nitroreductase-based probes such as 17a-Tre and Cy3-NO_2_-Tre exhibited less than a 10-fold FFC at a concentration of 10 nM while N14G achieved up to a 175-fold FFC after an incubation period of 1 h (Figure 3B), indicating that IrtAB-mediated active iron transport is more sensitive and rapid than trehalose-metabolism pathway or nitroreductase-switch approach. Notably, DMN-Tre did not exhibit any measurable FFC even at concentration as high as 0.2 μM (Figure S7). It was noted that N14B, unlike N14A, labeled *M. smegmatis* weakly at a concentration of 10 nM, indicating that N14B may diffuse into the bacterial cells (Figure 3B). Both Cy3 and Cy5 exhibited modest FFCs at a concentration of 10 nM, with Cy3 demonstrating superior performance compared to Cy5 (Figure 3B). The excitation and emission spectra of N14G, N14I, N14K, and their parent fluorescent probe, N14A, were comparable, confirming the role of MbT in the transport and labeling of *M. smegmatis* (Figure S10). The fluorescence intensity of *M. smegmatis* incubated with 10 nM N14G for 10 min was similar to that observed with 0.2 μM Cy3-NO_2_-Tre over a duration of 1 h, further indicating that MbT-based transport is significantly faster and more sensitive than nitroreductase-mediated metabolic and enzymatic pathways (Figure 3D). Consequently, N14G demonstrated its efficacy as an ultrasensitive probe for detecting *mycobacteria* at nanomolar concentrations; specifically, it achieved a 19-fold FFC in *M. smegmatis* at a concentration of 1.0 nM within a rapid incubation period of just 5 min. Further confocal microscopy revealed that N14G efficiently labeled *M. smegmatis* within 10 min while maintaining sustained labeling even after 24 h (Figure 3E). Prior to the application of N14G labeling, heat-killing susceptibility tests were performed. It was noted that treatment with N14G resulted in decreased FFC on heat-killed *M. smegmatis,* exhibiting an almost complete reduction at temperatures of both 95 and 75 °C, along with a decrease of approximately 57% at 55 °C (Figure 3G). Alternatively, treatment with the anti-TB drug BTZ043, a decaprenylphosphoryl-β-d-ribose-2′-epimerase (DprE1) inhibitor,^42^ resulted in over a 66% reduction in FFC among *M. smegmatis* pretreated with concentrations of 50 μM, 100 μM, and 250 μM for 5 h (Figure 3H). Despite BTZ043 treatment under these test conditions, some bacteria could still grow on 7H10 solid media (data not shown), indicating that drug treatment significantly reduced MbTFCp uptake in *M. smegmatis* while persisting cells to the BTZ043 drug could not be completely eradicated.

In microbiology, *Mycobacterium bovis* Bacille Calmette-Guérin (*M. bovis* BCG) is more closely related to *Mtb* than to *M. smegmatis*. The labeling of *M. bovis* BCG was investigated using various conjugate probes, which revealed strong fluorescent labeling at a concentration of 0.1 μM, likely attributable to the slow growth rate of *M. bovis* BCG. Furthermore, (*S*)-P10C-based N14G, (*R*)-P10C-based N14I, and P10C-based N14K exhibited comparable FFCs in *M. bovis* BCG, suggesting similar transport capacities for (*S*)-P10C, (*R*)-P10C, and P10C within *mycobacteria* (Figure S11).

### Characterization and Fluorescent Labeling Ability of Iron-Chelated MbTFCps

Fluorescence quenching can occur through iron chelation due to the paramagnetic properties and unfilled *d*-shell of Fe^3+^ ions.^31^ Specifically, Fe^3+^ ions facilitate the transformation of excited fluorescent molecules into nonluminescent triplet states. Furthermore, the unfilled *d*-shell of Fe^3+^ exerts a significant electron-withdrawing effect, thereby reducing the electron cloud density around the fluorescent group.^43^ To evaluate whether chelating Fe^3+^ ions could effectively quench the fluorescence of MbTFCps, N14G was mixed with FeCl_3_ in methanol to quantitatively prepare the N14G-Fe complex. The complex was purified using a reverse-phase C18 column, and its molecular weight was confirmed via HRMS (Figure S12). CD studies demonstrated that the Cotton effects observed for both N14G and the N14G-Fe complex were identical to those exhibited by P10C and P10C-Fe (Figure S13), indicating successful iron chelation to N14G. The binding affinity between (*S*)-P10C, the metal-binding template for N14G, and Fe^3+^ was assessed using UV–visible absorption spectroscopy in ethanol solution. Upon addition of varying concentrations of FeCl_3_ (ranging from 10 to 150 μM), the UV–visible absorption spectra for (*S*)-P10C at 100 μM displayed increased absorbance levels. The binding constant (*K*_B_) between (*S*)-P10C and Fe^3+^ was calculated as 1.19 × 10^5^ M^–1^ according to the Benesi–Hildebrand equation (Figure S14).^44^

**Figure 4:**
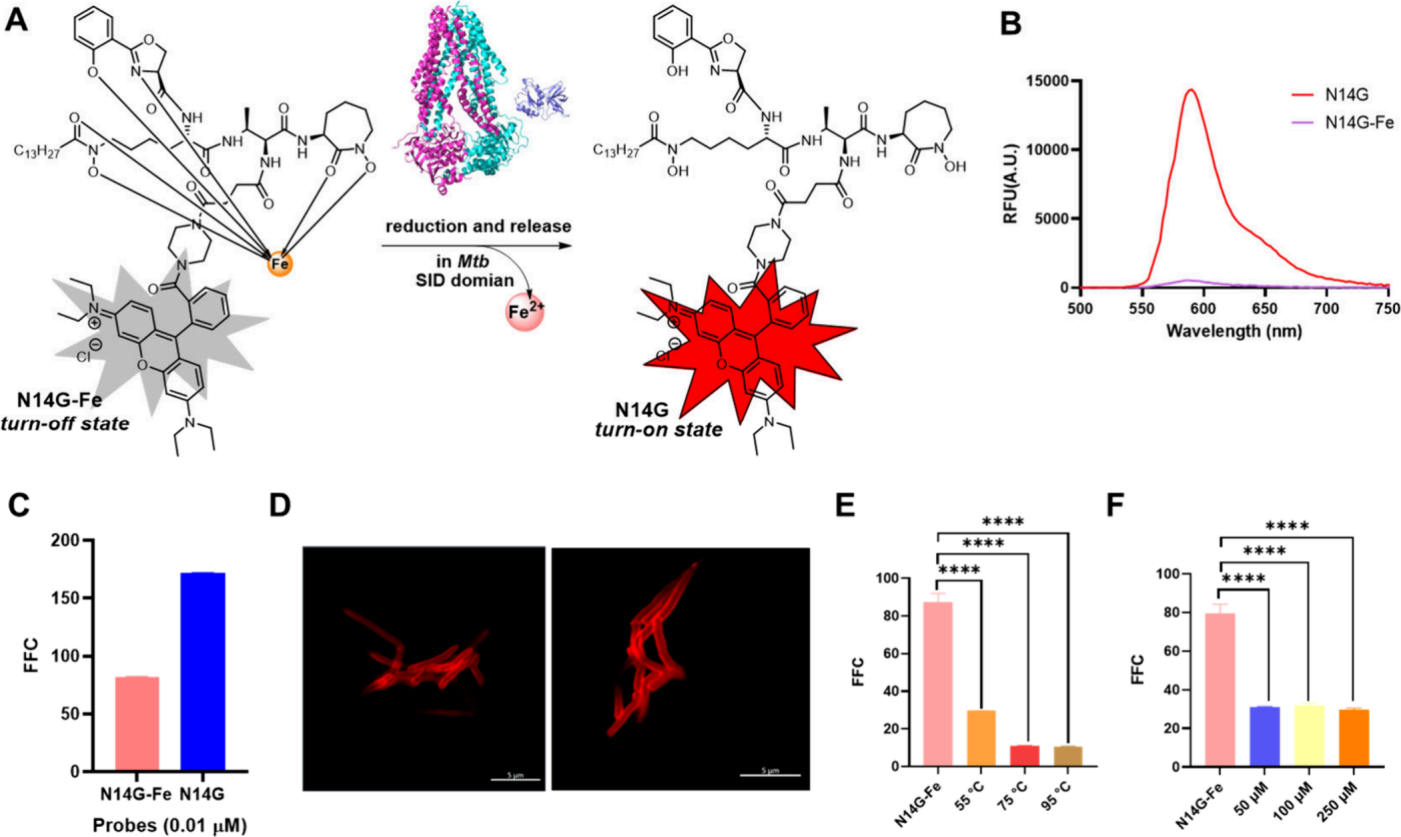
The fluorescence and biological tests of N14G-Fe. (A) The mechanism underlying the fluorescent switch in N14G-Fe. (B) Emission spectra of N14G (0.5 μM) and N14G-Fe (0.5 μM) in ddH_2_O. (C) Flow cytometry analysis of *M. smegmatis* incubated with N14G (0.01 μM) and N14G-Fe (0.01 μM) for 1 h. (D) Confocal image of live *M. smegmatis* labeled with N14G-Fe (0.01 μM) for 1 h. Scale bars, 5 μm. (E) Flow cytometry analysis of *M. smegmatis* pretreated by heat-killing at temperatures of 95 °C, 75 °C, and 55 °C for 30 min followed by incubation with N14G-Fe (0.01 μM) for additional 1 h. (F) Flow cytometry analysis of *M. smegmatis* pretreated with BTZ043 (50 μM, 100 μM, and 250 μM) for 5 h followed by incubation with N14G-Fe (0.01 μM) for additional 1 h.

The effective fluorescence quenching of N14G-Fe by Fe^3+^ ions was subsequently examined, revealing that the fluorescence intensity decreased to approximately 1/27 of that observed for N14G (Figure 4A and B). This result indicates that Fe^3+^ ions can function as an effective quenching switch when chelated with MbTFCps, turning off fluorescence outside the mycobacterial cell and reactivating it within the *mycobacteria* upon reduction and release of Fe^2+^ ions (Figure 4A). To test this hypothesis, *M. smegmatis* was incubated with 10 nM of N14G-Fe, resulting in an 81-fold FFC, demonstrating successful release of Fe^2+^ ions from N14G-Fe and reactivation of fluorescence within the *mycobacteria* following enzymatic reduction by IrtAB (Figure 4C). Even at a concentration of 1.0 nM, a nearly 15-fold FFC was observed in *M. smegmatis* after 1 h of incubation with N14G-Fe (Figure S15). Further investigation revealed that when equivalent or excess amounts of Fe^2+^ ions were added, the fluorescence intensity of N14G only reached approximately 50% of its original level observed in the absence of Fe^2+^ ion (Figure S16). This finding implies that regenerated MbT within *M. smegmatis* can also chelate Fe^2+^, leading to partial fluorescence quenching despite our inability to successfully prepare N14G chelated with Fe^2+^ ion. Additionally, similar results were obtained with the N14I-Fe complex in *M. smegmatis* as shown in Figure S17. Furthermore, imaging live *M. smegmatis* incubated with N14G-Fe demonstrated its capability for inducing fluorescent turn-on within *mycobacteria* (Figure 4D). Due to the specific fluorescent switching characteristics exhibited by N14G-Fe, direct imaging could be performed without requiring washing steps after incubation with *M. smegmatis* treated with this probe. Preheat-killing and pretreatment with BTZ043, a potent inhibitor targeting DprE1, significantly reduced FFC in *M. smegmatis* after incubation with N14G-Fe (Figure 4E and F). These findings are consistent with the FFC observed for N14G (Figure 3G and H), further indicating that both compounds can penetrate *mycobacteria* and that the fluorescence intensity of N14G-Fe can be reactivated through an IrtAB-mediated active pathogenic iron transport mechanism of live *mycobacteria*.

In summary, MbT can effectively capture trace amounts of Fe^3+^ ions from the environment due to its strong chelating ability. The chelated Fe^3^^+^ ions quench the fluorescence of MbTFCps and are subsequently reduced to release Fe^2+^ ions within *mycobacteria*. Consequently, the fluorescence of MbTFCps, specifically N14G-Fe, can be reactivated inside bacterial cells, enabling specific labeling of *Mtb* with a rapid response time using an ultralow concentration of the probe.

### Molecular Docking Studies of SID Interactions with N14G and N14G-Fe

**Figure 5:**
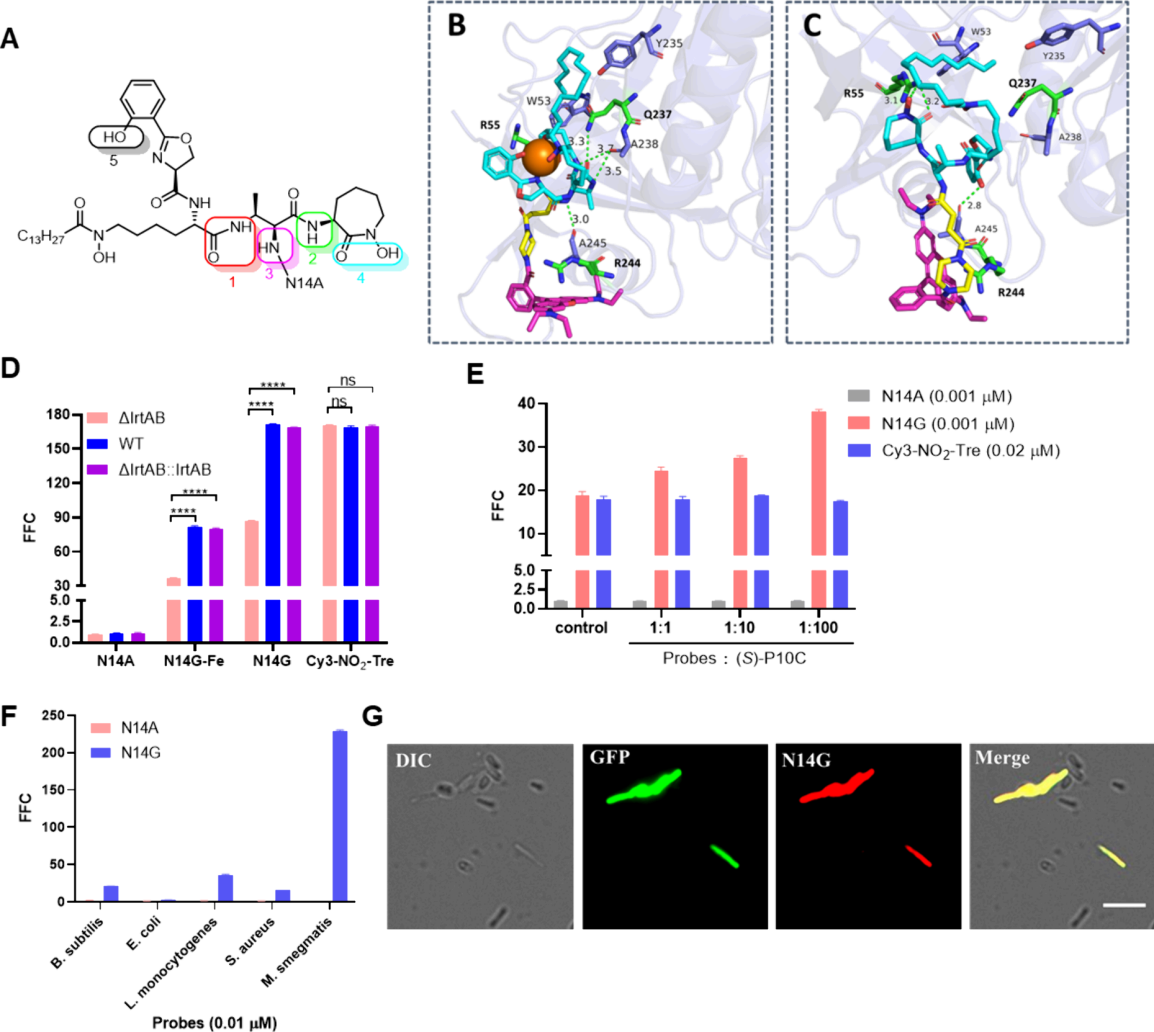
Specific transport of N14G and N14G-Fe by the heterodimeric ABC transporter IrtAB. (A–C) Docking studies of the MbT binding pocket in the SID (PDB code: 6TEK) with N14G-Fe (B) and N14G (C). Docking was conducted using MOE software, and images were generated with Pymol. Three conserved residues lining the MbT binding pocket, R55, R244, and Q237, are highlighted as green sticks. Green dashed lines indicate hydrogen bond interactions. The MbT binding pocket along with interacting residues is depicted in stick mode, where cyan represents MbT, magenta represents rhodamine, orange represents iron, and yellow represents the linker. (D) Flow cytometry analysis of knockout mutant ΔIrtAB, WT, and complemented ΔIrtAB::IrtAB *M. smegmatis* strains incubated with N14A (0.01 μM), N14G-Fe (0.01 μM), N14G (0.01 μM), and Cy3-NO_2_-Tre (0.2 μM) for 1 h. (E) Flow cytometry analysis of *M. smegmatis* incubated with N14A (0.001 μM), N14G (0.001 μM), and Cy3-NO_2_-Tre (0.02 μM) in the presence of varying concentrations of excess (*S*)-P10C for 1 h. (F) Flow cytometry analysis of *B. subtilis*, *E. coli, S. aureus*, *L. monocytogenes*, and *M. smegmatis*. All bacteria were treated with N14A (0.01 μM) and N14G (0.01 μM) for 1 h. (G) Specific labeling of GFP-expressing *M. smegmatis* in the presence of a mixed population of *E. coli*, *S. aureus*, *B. subtilis*, and *L. monocytogenes* with N14G (0.01 μM). Images were collected using differential interference contrast (DIC), GFP, or Cy3 channels. The “Merge” panel represents merged images from all channels. Scale bar, 5 μm.

To gain a deeper understanding of the interactions between N14G or N14G-Fe and the endogenous siderophore interaction domain (SID), a critical component of IrtAB responsible for the reduction of Fe^3+^ to Fe^2+^, molecular docking studies were conducted using Molecular Operating Environment (MOE) software (Chemical Computing Group ULC, 910–1010 Sherbrooke St. W., Montreal, QC H3A 2R7, Canada, 2024). Figures 5A–C and S18 illustrate the binding interactions of N14G and N14G-Fe within the MbT binding pocket, which is formed by three conserved amino acids (R55, Q237, and R244) in the SID region as previously reported by Arnold *et al*.^5^ The N14G-Fe-SID complex (Figure 5B) exhibited four identified hydrogen bonds (HBs). Specifically, the amide group of N14G-Fe (highlighted in red in Figure 5A) formed two HBs: one with the side chain hydrogen atom of SID residue Q237 at a distance of 3.3 Å and another with the main chain oxygen atom of SID residue A238 at a distance of 3.5 Å. Additionally, the amide hydrogen atom of N14G-Fe (highlighted in green in Figure 5A) established an HB with the main chain oxygen atom of SID residue A238 at a distance of 3.7 Å. Furthermore, another amide hydrogen atom from N14G-Fe (highlighted in magenta in Figure 5A) formed an HB with the main chain oxygen atom of SID residue A245 at a distance of 3.0 Å.

In the N14G-SID complex (Figure 5C), the MbT molecule of N14G, devoid of the chelated Fe^3+^ ion, also fit well into the MbT binding pocket, with three HBs identified. The oxygen atom and hydroxyl group of N14G (highlighted in cyan in Figure 5A) formed two critical HBs with the side chain of SID residue R55, previously recognized as one of the key amino acids within the MbT binding pocket.^5^ Additionally, the phenolic hydroxyl group of N14G (highlighted in black in Figure 5A) established an additional HB with the main chain carbonyl group of SID residue A245 at a distance of 2.8 Å. Notably, both N14G and N14G-Fe engaged in HB interactions with this main chain carbonyl group from SID residue A245. Previous studies have demonstrated that substituting adjacent SID residue R244 with E244 results in a loss of activity.^5^ We hypothesize that this R244E mutation induces a conformational change in SID residue A245, thereby disrupting its binding affinity for MbT. This suggests that SID residue A245 may be a crucial amino acid involved in facilitating transport of either N14G or N14G-Fe by IrtAB.

### Validation of Specific IrtAB Target within *Mycobacteria* for MbTFCps

To confirm the specific transport of N14G into *mycobacteria* via the IrtAB transporter, we generated an IrtAB-knockout *M. smegmatis* strain (ΔIrtAB) and its complemented strain (ΔIrtAB::IrtAB), which were verified by PCR and DNA sequencing (Figure S19). The fluorescence intensity of N14G decreased by 53% in the ΔIrtAB strain compared to that observed in the wild-type (WT) strain, while comparable fluorescence levels were noted between the WT and ΔIrtAB::IrtAB strains (Figure 5D). In control experiments, no significant changes in fluorescence intensity were detected in either the knockout or complemented strains when treated with N14A or Cy3-NO_2_-Tre (Figure 5D). Upon incubation with N14G-Fe, a similar decrease in fluorescence intensity was observed as with N14G, suggesting that both compounds were specifically transported into bacterial cells and activated by the IrtAB transporter, resulting in strong fluorescence within *mycobacteria*.

Analysis of the data from Figure 5B–D revealed that the fluorescence intensity of N14G-Fe was approximately half that of N14G in both WT and ΔIrtAB strains. This finding is consistent with previous observations in Figures 4C and S16, where the presence of reduced Fe^2+^ ions within *mycobacteria* resulted in a decrease of FFC to half of the original values. This suggests that both N14G and N14G-Fe can effectively traverse the cell wall and inner membrane region where IrtAB is located; however, iron-free N14G exhibits greater fluorescence capability than N14G-Fe.

To further confirm the role of IrtAB in the transport of N14G, a MbT competitive assay was conducted using (*S*)-P10C as a competitor. Notably, a concentration-dependent increase in fluorescence intensity was observed when *M. smegmatis* was treated with a combination of N14G and (*S*)-P10C at molar ratios of 1:1, 1:10, and 1:100 (Figure 5E). A similar enhancement in fluorescence was noted when equal or excess P10C relative to N14G was employed, as illustrated in Figure S20. High-performance liquid chromatography analysis revealed a 49% increase in N14G uptake by *M. smegmatis* in the presence of a 1:1 molar ratio of P10C to N14G (Figure S21). This fluorescence enhancement was also evident in the ΔIrtAB strain (Figure S22). Importantly, this phenomenon has been previously documented in labeling studies involving *E. coli* and *P. aeruginosa* by Klahn *et al*.^10^ This may be attributed to an unidentified alternative transporter for MbT located on the cell wall (Figure 1). Subsequently, we protected two *N*-hydroxyl groups within MbT with benzoyl groups to create an MbT analogue (N14G-Bz) that cannot chelate Fe^3+^ ions. Interestingly, N14G-Bz exhibited comparable intracellular fluorescence intensity to that of N14G (Figure S23), also suggesting the existence of a new noniron-dependent MbT transporter within mycobacterial cell wall. We also attempted to synthesize an MbT analogue devoid of any hydroxyl groups or fully protect all *N*-hydroxyl and phenolic hydroxyl groups through methylation or benzylation strategies. These modifications were intended to prevent Fe^3+^ chelation; however, we encountered challenges due to instability issues with the phenolic hydroxyl-protected A3 intermediate under various chemical conditions (Figure S23).

The literature indicates that the SID is essential for reducing MbT-Fe but not cMbT-Fe.^5^ The cMbT was further successfully synthesized, and its binding constant (*K*_B_) to FeCl_3_ was determined to be 2.74 × 10^3^ M^–1^ using UV–visible absorption spectroscopy and the Benesi–Hildebrand eq (Figures S24 and S25). However, attempts to prepare cMbTFCp and cMbTFCp-Fe conjugates were unsuccessful in our experiments due to compound instability under various reaction conditions. Notably, cMbT also increased the FFC of N14G labeling *M. smegmatis*, providing valuable insights for future research on cMbT transport and further suggesting a potential additional MbT transporter located on the mycobacterial cell wall (Figure S26).

The specificity of N14G labeling for *mycobacteria* was investigated. A mixture of *M. smegmatis*, *Bacillus subtilis* (*B. subtilis*), *Escherichia coli* (*E. coli*), *Staphylococcus aureus* (*S. aureus*), and *Listeria monocytogenes* (*L. monocytogenes*) was analyzed using flow cytometry, which demonstrated that N14G specifically labeled *M. smegmatis* more effectively than the other bacterial strains (Figure 5F). Similarly, iron-chelated N14G-Fe also showed preferential labeling of *M. smegmatis* over other bacteria (Figure S27). Confocal imaging further confirmed the selective labeling of *M. smegmatis* expressing green fluorescent protein (GFP) when mixed with *E. coli*, *S. aureus*, *B. subtilis*, and *L. monocytogenes* (Figure 5G). These results indicate that IrtAB functions as a specific transporter in *mycobacteria*. Notably, the IrtAB/MbT system presents a promising novel and specific target for detecting *mycobacteria*.

### Detection of *Mtb* in Sputum Samples from Patients with TB

**Figure 6:**
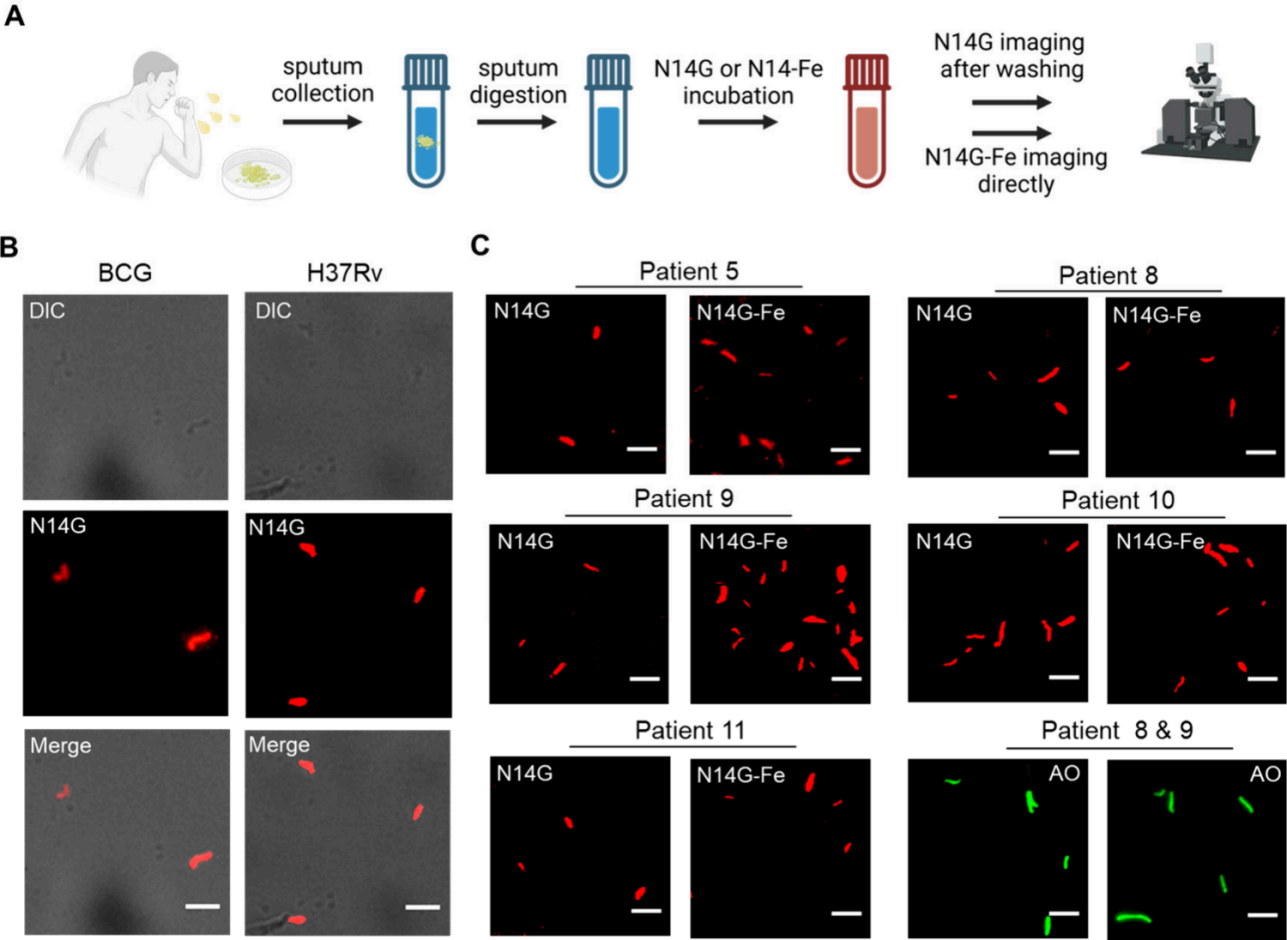
Detection of *Mtb* in sputum samples from patients with tuberculosis. (A) Illustration of the procedure for incubating sputum samples with N14G or N14G-Fe. Decontaminated samples were treated with either N14G (0.1 μM) or N14G-Fe (0.1 μM) for 10 min. Following incubation, samples were either washed and imaged (N14G) or imaged directly without washing (N14G-Fe). (B) Image depicting the labeling of *M. bovis* BCG and H37Rv using N14G (0.1 μM) for 10 min. Scale bars, 5 μm. (C) Images of sputum samples from patients with TB treated with N14G (0.1 μM) and N14G-Fe (0.1 μM) for 10 min, or AO reagent. Scale bars, 5 μm.

Prior to detecting *Mtb* in sputum samples from patients with TB (Figure 6A), we labeled *M. bovis* BCG and the laboratory strain H37Rv with N14G and N14G-Fe to demonstrate the capability of MbTFCps for *Mtb* detection. As illustrated in Figures 6B and S28–S31, both *M. bovis* BCG and H37Rv were effectively labeled by N14G and N14G-Fe at a concentration of 0.1 μM after a 10 min incubation period. The limit of detection (LOD) for N14G was determined to be 34 Colony-Forming Unit (CFU) for H37Rv (Figure S31), significantly surpassing that of the previously reported Cy3-NO_2_-Tre probe (LOD = 430 CFU).^19^

Detection of *Mtb* in sputum samples from patients with TB was successfully accomplished using both N14G and N14G-Fe. The probes were evaluated on sputum samples from 11 TB patients, employing a concentration of 0.1 μM for either N14G or N14G-Fe with a 10 min incubation following decontamination with NALC/NaOH (Figures 6C and S33–S37). The iron-free probe N14G effectively labeled *Mtb* in patient sputum, exhibiting red fluorescence, while N14G-Fe displayed comparable fluorescence intensity, indicating that both compounds could serve as potential chemical tools for clinical applications due to the Fe^3^^+^ switch. Control experiments included staining sputum samples from healthy donors with either N14G or N14G-Fe, as well as staining TB patient samples with AO reagent (Figures S38–S40). No fluorescence was detected in the sputum of healthy donors. These results suggest that mycobactins, whether secreted by *mycobacteria* or synthesized in the laboratory, can function effectively as sensitive and specific diagnostic tools for *Mtb* in clinical settings.

## Conclusions

In this article, we successfully employed the conjugation of MbT with fluorescent probes to achieve ultrasensitive and specific diagnosis of *Mtb*. By chelating Fe^3+^ ions, these probes effectively quenched the fluorescence of MbTFCps, including N14G-Fe and N14I-Fe. The IrtAB transporter was identified as a specific target, playing a dual role in both transporting the probes and activating the fluorescence of the iron-chelated probe N14G-Fe. The iron-free probe N14G demonstrated selective detection of *mycobacteria* and achieved a 19-fold and a 134-fold increased FFC, respectively, at 1.0 nM or 10 nM after just 5 min of incubation with *M. smegmatis*; meanwhile, both *M. bovis* BCG and wild-type *Mtb* strain H37Rv were effectively labeled with either N14G or N14G-Fe at a concentration of 0.1 μM for 10 min of incubation. Furthermore, N14G achieved an impressive LOD of 34 CFU for H37Rv. Both N14G and N14G-Fe successfully diagnosed *Mtb* in clinical sputum samples from patients with TB. These findings highlight a rapid and ultrasensitive approach for detecting *Mtb* utilizing the Fe^3+^ ion transport system.

In summary, this study demonstrates how the IrtAB iron transport can be utilized for ultrasensitive detection of *Mtb* in both cellular and clinical settings. By focusing on mechanisms related to iron acquisition, new diagnostic strategies could enhance infection detection, including those involving drug-resistant strains, leading to improved patient management and outcomes in combating TB. Additionally, the data presented herein suggest there may exist an unidentified noniron-dependent transporter for mycobactin on the cell wall of *mycobacteria*, which warrants further investigation.

## Supporting Information Available

Supplementary figures; Experimental procedures of chemistry; Experimental procedures of biology; CD spectral analysis and molecular docking; ^1^H, ^13^C, and ^19^F NMR spectra (PDF)
