# Supplementary material for: Insights into IrtAB: Iron Transport Facilitates Ultrasensitive Detection of Mycobacteria in Both Cellular and Clinical Environments
Source: ACS Cent Sci. 2025 Jan 7;11(2):261–71. doi: 10.1021/acscentsci.4c00676 (PMC11869132; doi:10.1021/acscentsci.4c00676)
Supplement: Supplementary file 1 — oc4c00676_si_001.pdf [file oc4c00676_si_001.pdf]

Insights into IrtAB: Iron Transport Facilitates Ultrasensitive Detection of *Mycobacteria* in Both Cellular and Clinical Environments

Dianmo Ni[1,#], Xiaoqiao Hong[1,#], Dingyi Liu[2,3], Xueyuan Li[1], Li Li[4], Wenwu Liu[1], Zhaogang Sun[2,3], Gang Liu[1,*]

[1] School of Pharmaceutical Sciences, Tsinghua University, Haidian District, Beijing 100084, P. R. China

[2] Translational Medicine Center, Beijing Chest Hospital, Capital Medical University, Tongzhou District, Beijing, 101149, P. R. China

[3] Beijing Key Laboratory in Drug Resistant Tuberculosis Research, Beijing Tuberculosis & Thoracic Tumor Research Institute, Tongzhou District, Beijing, 101149, P. R. China

[4] Institute of Materia Medica, Chinese Academy of Medical Sciences & Peking Union Medical College, Xicheng District, Beijing, 100050, P. R. China

Content

I. Supplementary Figures S5

Figure S1. Chiral analysis of building block A2. S5
Figure S2. Chiral analysis of building block BocA7. S6
Figure S3. Structure of (*R*)-P10C-16. S6
Figure S4. Structure and mass spectrum detection of P10C-Fe. S7
Figure S5. Excitation and emission spectra of conjugates (0.5 μM) in ddH_2O. S8
Figure S6. Structures of Cy3-NO_2-Tre, DMN-Tre, 17a-Tre, Cy3, and Cy5. S8
Figure S7. Flow cytometry analysis of *M. smegmatis* incubated with different probes at different concentrations (0.001 μM, 0.005 μM, 0.01 μM, 0.1 μM, and 0.2 μM, respectively) for 1 h. S9
Figure S8. Structure of N14L. S9
Figure S9. Flow cytometry analysis of *M. smegmatis* incubated with N14L (0.001 μM, 0.005 μM, 0.01 μM, 0.1 μM, and 0.2 μM, respectively) for 1 h. S10
Figure S10. Excitation and emission spectra of N14G, N14I, and N14K (0.5 μM) in ddH_2O. S10
Figure S11. Flow cytometry analysis of *M. bovis* BCG incubated with different probes (0.1 μM) for 1 h. S10
Figure S12. HRMS of N14G-Fe complex. S11
Figure S13. CD spectra of N14G-Fe complex (black) and N14G (red). S11
Figure S14. UV-visible spectra of (*S*)-P10C (100 μM) in the presence of increasing concentrations of $FeCl_3$ (from 10 μM to 150 μM) in EtOH. S12
Figure S15. Flow cytometry analysis of *M. smegmatis* treated with N14G-Fe in different concentrations (0.001 μM, 0.005 μM, 0.01 μM, 0.1 μM, and 0.2 μM, respectively) for 1 h. S12
Figure S16. Fluorescent spectrum of N14G (0.5 μM) when combined with different concentration of Fe^{2+} ion in ddH_2O. S12
Figure S17. Flow cytometry analysis of *M. smegmatis* incubated with N14G-Fe (0.1 μM) and N14I-Fe (0.1 μM) for 1 h. S13
Figure S18. Docking studies of compound N14G-Fe, N14G, (*S*)-P10C-Fe, and (*S*)-P10C with the mycobactin pocket of SID. S13
Figure S19. Verification of the construction of knockout mutant strain ΔIrtAB *M. smegmatis* and complemented strain ΔIrtAB::IrtAB *M. smegmatis*. S14
Figure S20. Flow cytometry analysis of wild-type *M. smegmatis* incubated with N14A (0.001 μM) and N14G (0.001 μM) in presence of excess of P10C (0.01 μM and 0.1 μM) for 1 h. S14
Figure S21. Quantitative analysis by analytical HPLC about fluorescence enhancement in presence of excess of P10C. S15
Figure S22. Flow cytometry analysis of ΔIrtAB *M. smegmatis* incubated with N14A (0.001 μM) and N14G (0.001 μM) in presence of excess of P10C (0.01 μM and 0.1 μM) for 1 h. S16
Figure S23. Structure of N14G-Bz and flow cytometry analysis of *M. smegmatis*

incubated with N14G-Bz (0.001 μM, 0.005 μM, and 0.01 μM, respectively) for 1 h. S16
Figure S24. Structure of synthesized cMbT. S16
Figure S25. UV-visible spectra of cMbT (100 μM) in the presence of increasing concentrations of $FeCl_3$ (from 10 μM to 150 μM) in EtOH. S17
Figure S26. Flow cytometry analysis of *M. smegmatis* incubated with N14A (0.001 μM), N14G (0.001 μM), and Cy3-NO_2-Tre (0.02 μM), respectively, in the presence of excess of cMbT with different concentrations for 1 h. S17
Figure S27. Flow cytometry analysis of *B. subtilis*, *E. coli*, *S. aureus*, *L. monocytogenes,* and *M. smegmatis*. All bacteria were treated with N14G-Fe (0.01 μM) for 1 h. S17
Figure S28. Imaging of *M. bovis* BCG with N14G. S18
Figure S29. Imaging of *M. bovis* BCG with N14G-Fe. S18
Figure S30. Imaging of H37Rv with N14G. S19
Figure S31. Imaging of H37Rv with N14G-Fe. S19
Figure S32. Correlation of integrated fluorescence and the number of H37Rv in culture medium. H37Rv were imaged after incubation with N14G (1.0 μM) for 1 h. S20
Figure S33. Images of sputum samples of patients 1–4 with TB treated with N14G (0.1 μM). S20
Figure S34. Images of sputum samples of patients 5–7 with TB treated with N14G (0.1 μM). S21
Figure S35. Images of sputum samples of patients 5–7 with TB treated with N14G-Fe (0.1 μM). S21
Figure S36. Images of sputum samples of patients 8–11 with TB treated with N14G (0.1 μM). S22
Figure S37. Images of sputum samples of patients 8–11 with TB treated with N14G-Fe (0.1 μM). S22
Figure S38. Images of sputum samples of patients 8–11 with TB treated with standard AO staining method. S23
Figure S39. Images of sputum samples of healthy donors treated with N14G or N14G-Fe (0.01 μM). S24
Figure S40. Images of sputum samples of healthy donors treated with standard AO staining method. S24

II. Experimental Procedures of Chemistry

2.1 Synthesis of mycobactin analogues P10C-16 and P10C S25
2.2 Synthesis of mycobactin analogues (*S*)-P10C-16 and (*R*)-P10C-16 S33
2.3 Synthesis of (*S*)-P10C and (*R*)-P10C S40
2.4 Synthesis of fluorophores S41
2.5 Synthesis of MbTFCps (N14C~N14L) S42
2.6 Synthesis of P10C-Fe, N14G-Fe and N14I-Fe S47
2.7 Synthesis of cMbT S47
2.8 HPLC analysis of representative target compounds S51

III. Experimental Procedures of Biology **S57**
3.1 Bacterial culture condition S57
3.2 Construction of knockout strain ΔIrtAB *M. smegmatis* S57
3.3 Construction of complemented strain ΔIrtAB::ΔIrtAB *M. smegmatis* S58
3.4 Fluorescence fold change of bacteria analyzed by flow cytometry S59
3.5 Heat-killing experiment S60
3.6 Drug treating experiment S60
3.7 Fluorescence microscopy S60
3.8 Photophysical properties S60
3.9 UV-Visible absorption spectroscopy S60
3.10 Detection of bacteria in culture S61
3.11 Staining of sputum samples with N14G S61
3.12 Statistical analysis S61
IV. CD Spectral Analysis and Molecular Docking **S62**
4.1 CD spectral analysis S62
4.2 Molecular docking studies S62
V. ^{1}H,^{13}C and ^{19}F NMR Spectra **S63**

I. Supplementary Figures

Machine: Agilent Technologies 1260 Infinity
Chiral column: HPLC Chiralpak® AD-H column, particle size 5 μm, dimensions 4.6 mm×250 mm
Elution gradient: (*n*-hexane with 1‰ TFA)/(isopropanol with 1‰ TFA) = 99:1
Flow rate: 0.5 mL/min
Detection wavelength = 254 nm

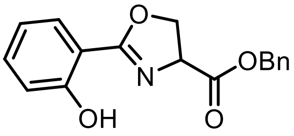

rac-A2

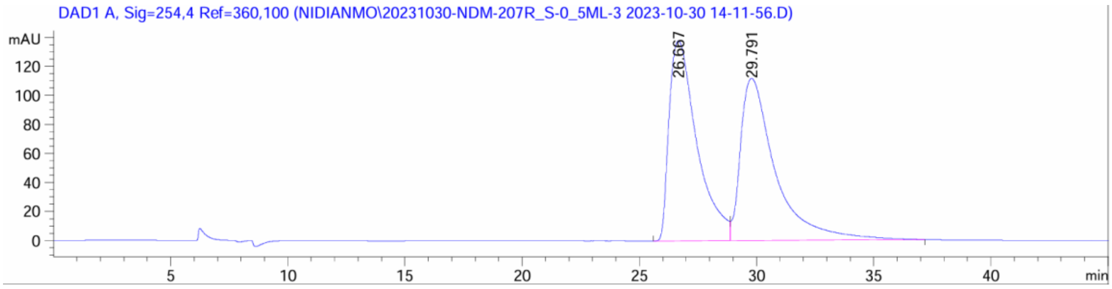

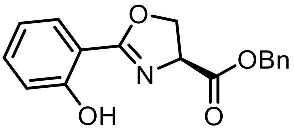

A2

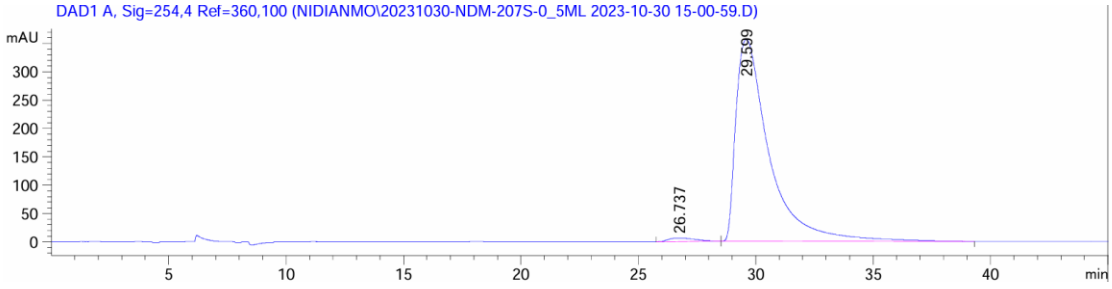

Retention Time (min)	Area (mAU* s)	Area%
26.7	500.62625	1.3998
29.5	$3.52627e^4$	98.6002
totals	$3.57633e^4$	100

As the chiral HPLC data of A2 showed, the cyclization has chirality retention with *ee* value > 97%.

Figure S1. Chiral analysis of building block A2.

Machine: Agilent Technologies 1260 Infinity
Chiral column: HPLC Chiralpak® AD-H column, particle size 5 μm, dimensions 4.6 mm×250 mm

Elution gradient: (*n*-hexane with 1‰ TFA)/(isopropanol with 1‰ TFA) = 90:10
Flow rate: 1 mL/min
Detection wavelength = 230 nm

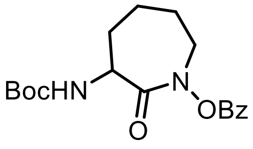

***rac*-BocA7**

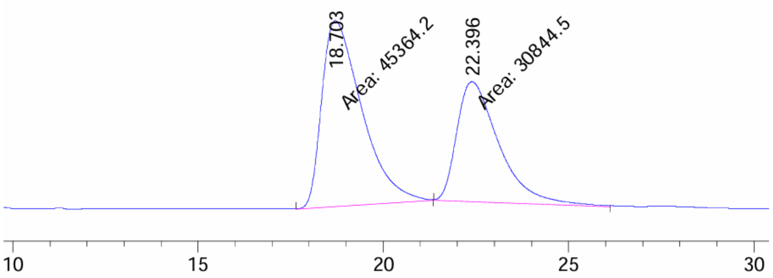

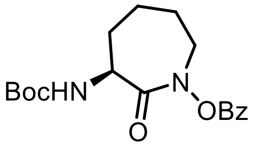

BocA7

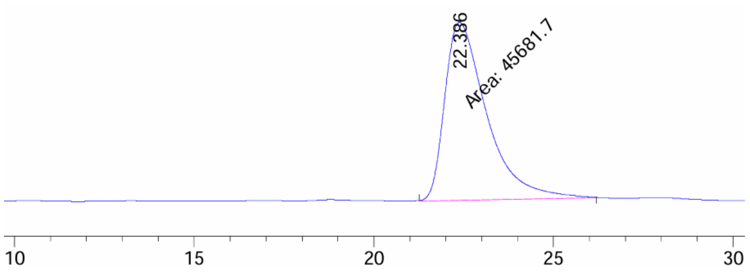

Retention Time (min)	Area (mAU* s)	Area%
22.3	45681.7	100
totals	45681.7	100

As the chiral HPLC data of BocA7 showed, the cyclization has chirality retention with *ee* value > 99%.

Figure S2. Chiral analysis of building block BocA7.

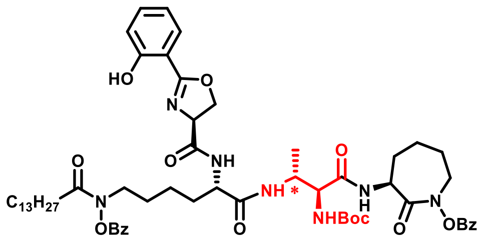

(*R*)-P10C-16

Figure S3. Structure of (*R*)-P10C-16.

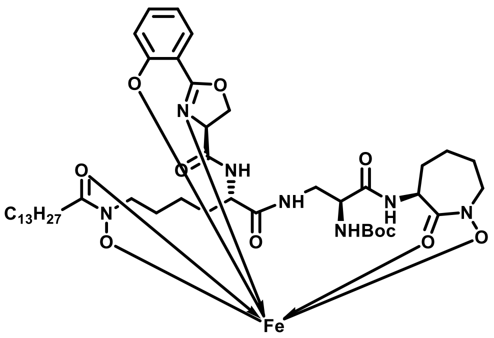

P10C-Fe

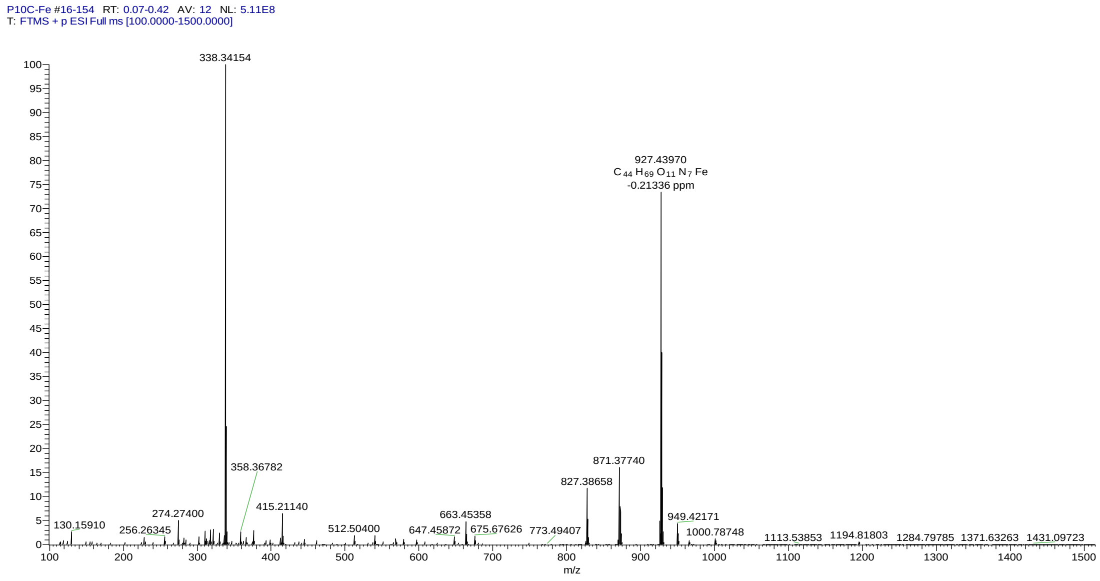

Figure S4. Structure and mass spectrum detection of P10C-Fe. HRMS (ESI-MS) *m/z*: $[M+H]^+$; Calcd for $C_{44}H_{69}FeN_7O_{11}$ 927.4399; Found 927.4397.

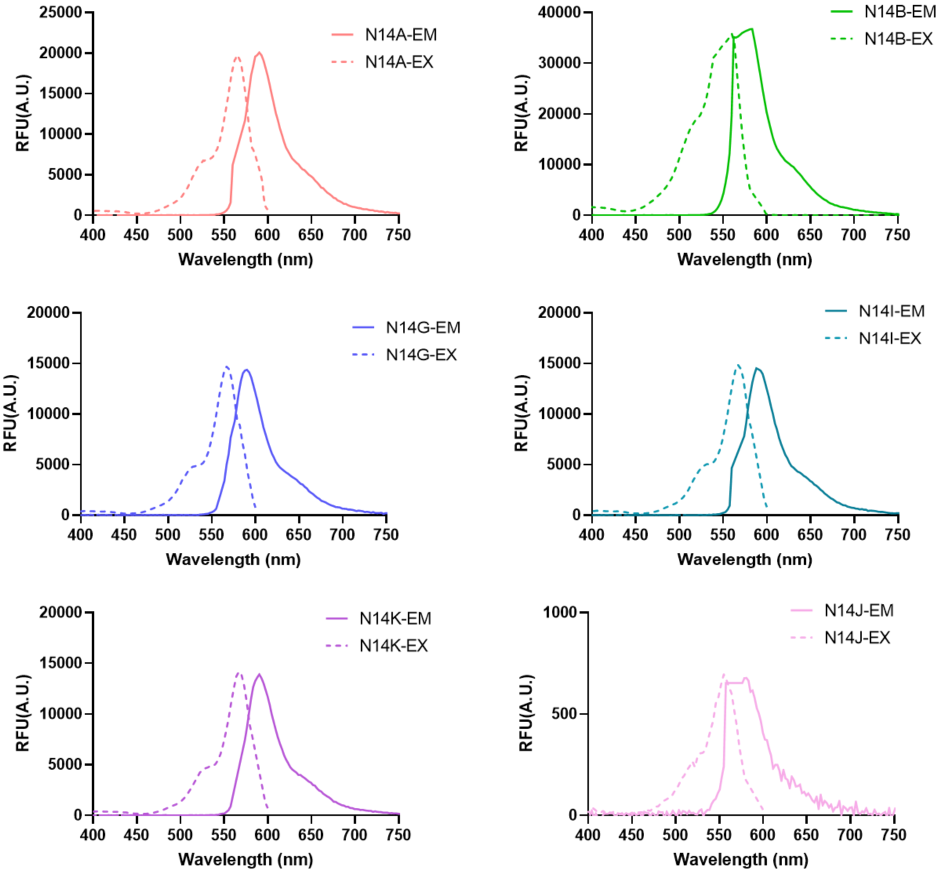

Figure S5. Excitation and emission spectra of conjugates (0.5 μM) in ddH_2O.

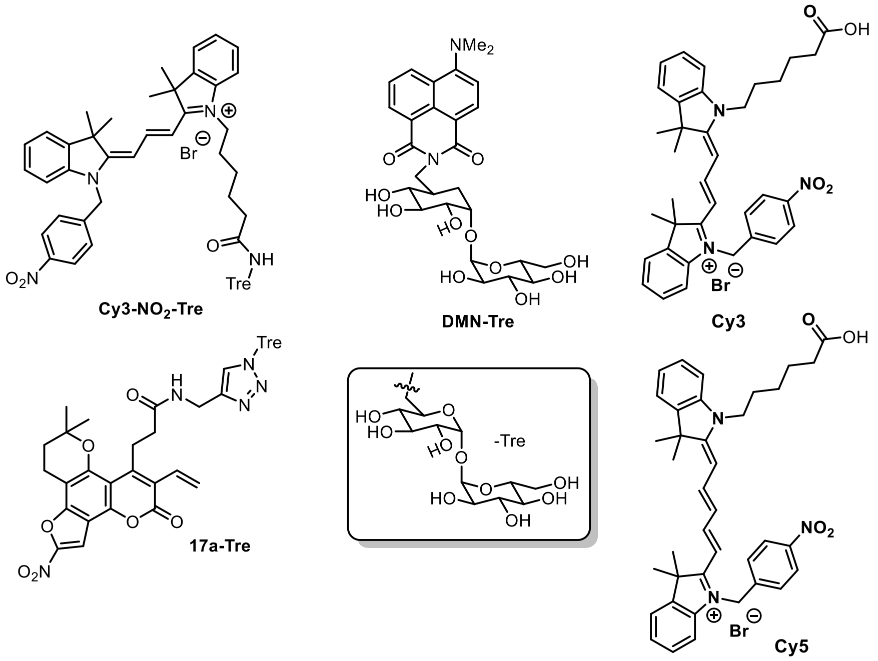

Figure S6. Structures of Cy3-NO_2-Tre, DMN-Tre, 17a-Tre, Cy3, and Cy5.

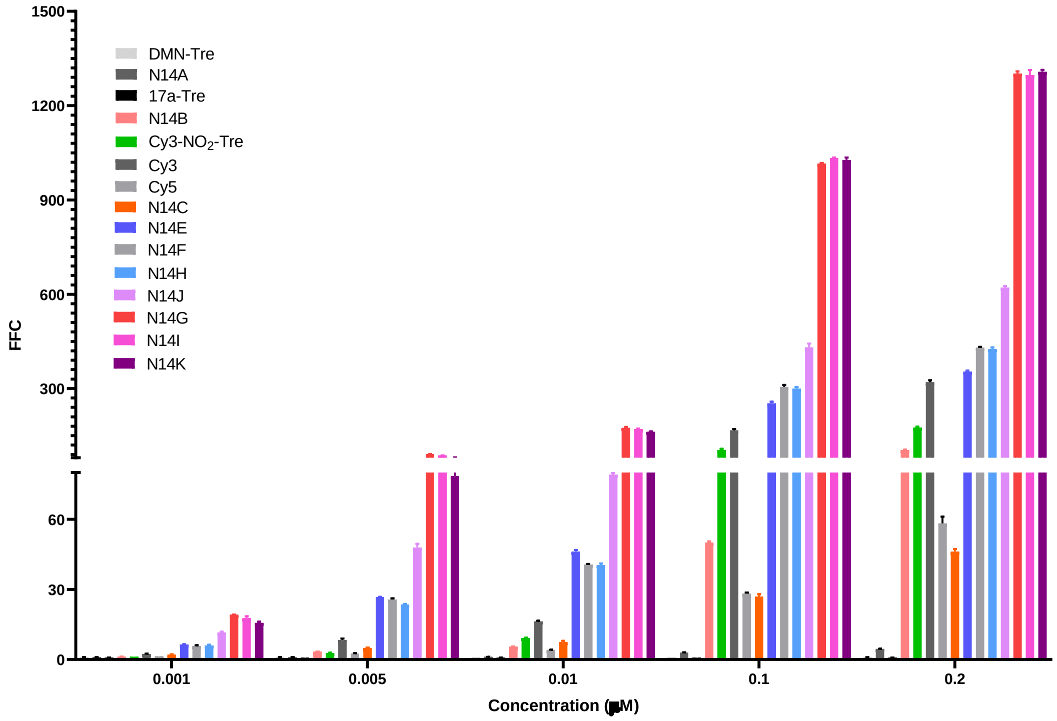

Figure S7. Flow cytometry analysis of *M. smegmatis* incubated with different probes at different concentrations (0.001 μM, 0.005 μM, 0.01 μM, 0.1 μM, and 0.2 μM, respectively) for 1 h.

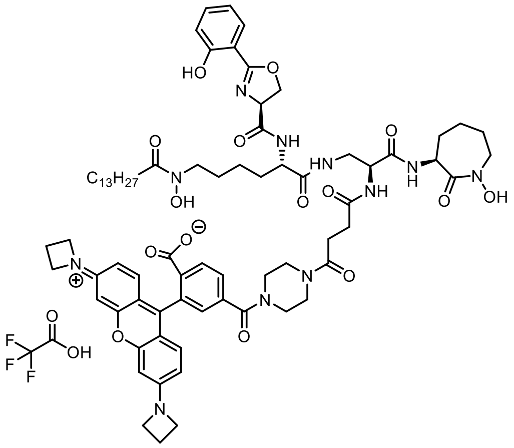

Figure S8. Structure of N14L.

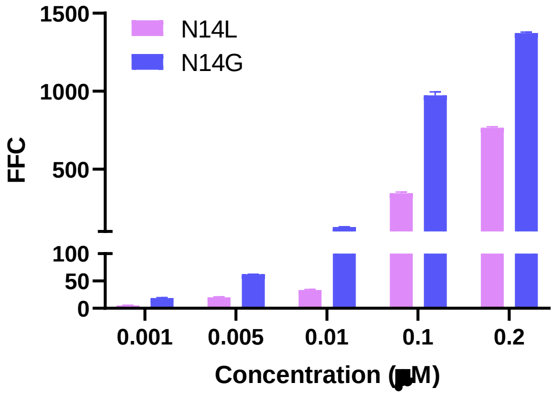

Figure S9. Flow cytometry analysis of *M. smegmatis* incubated with N14L (0.001 μM, 0.005 μM, 0.01 μM, 0.1 μM, and 0.2 μM, respectively) for 1 h.

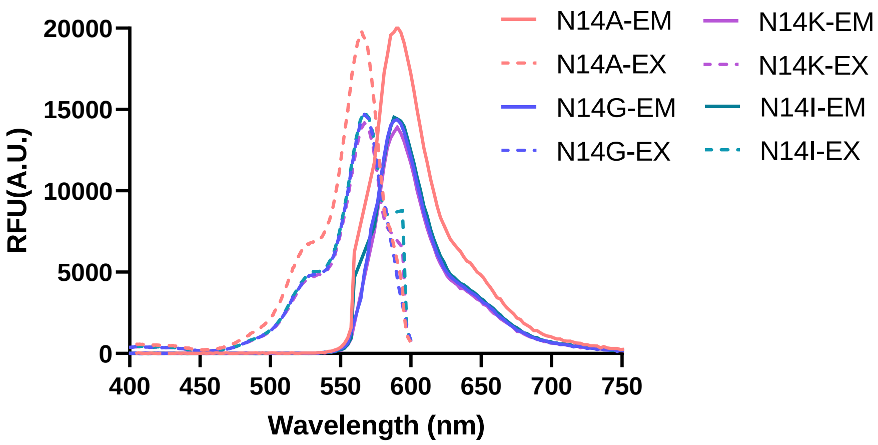

Figure S10. Excitation and emission spectra of N14G, N14I, and N14K (0.5 μM) in ddH_2O.

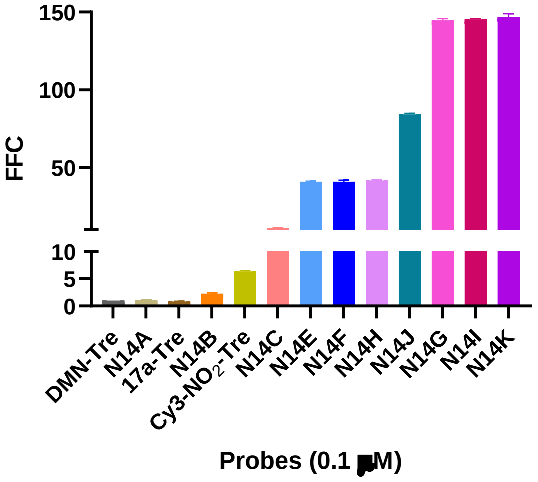

Figure S11. Flow cytometry analysis of *M. bovis* BCG incubated with different probes (0.1 μM) for 1 h.

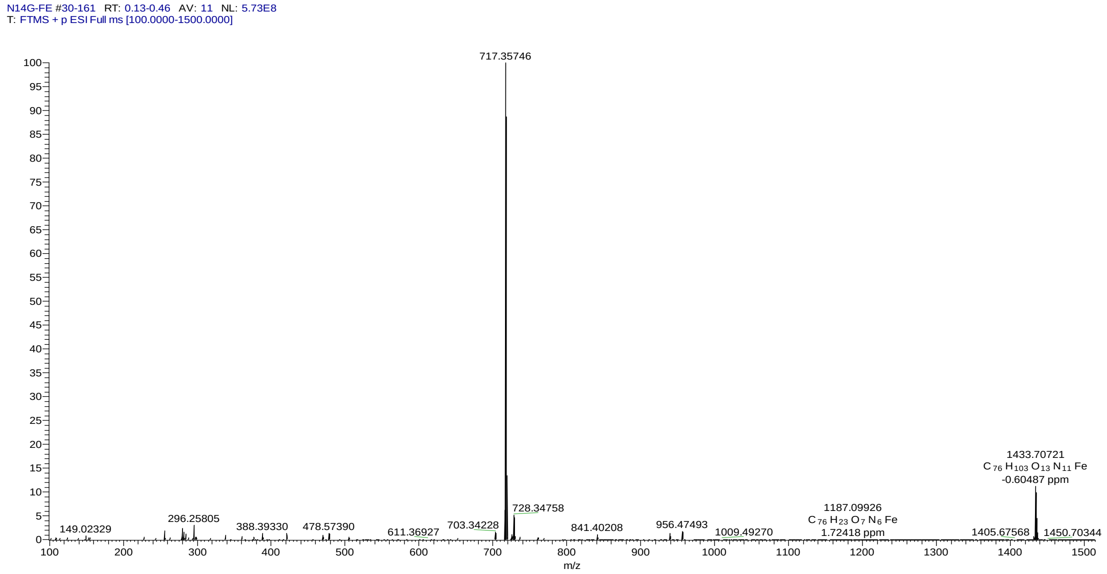

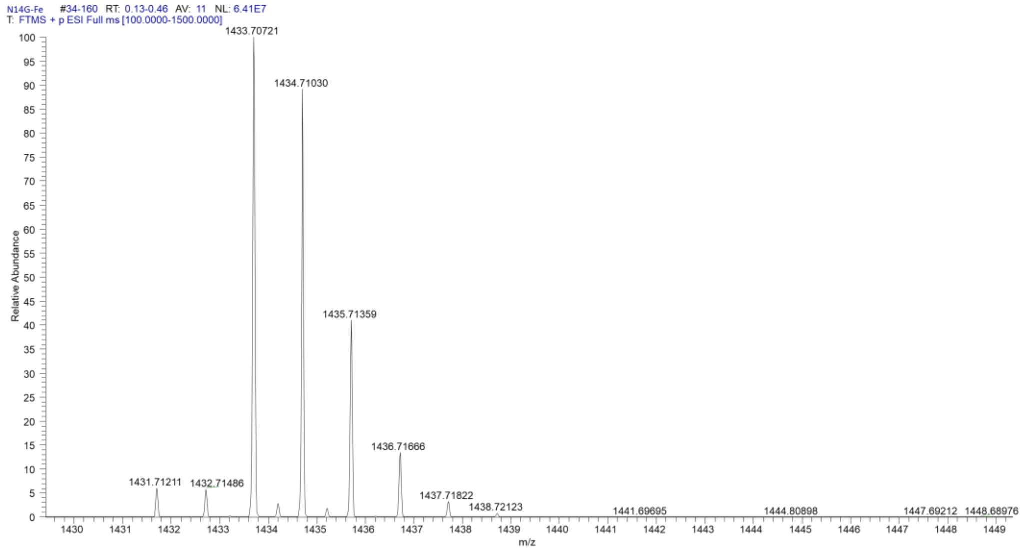

Figure S12. HRMS of N14G-Fe complex. HRMS (ESI-MS) *m/z*: $[M+H]^+$; Calcd for $C_{76}H_{103}FeN_{11}O_{13}$ 1433.7081; Found 1433.7072.

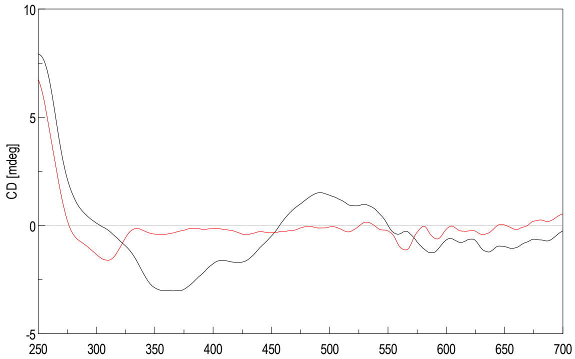

Figure S13. CD spectra of N14G-Fe complex (black) and N14G (red).

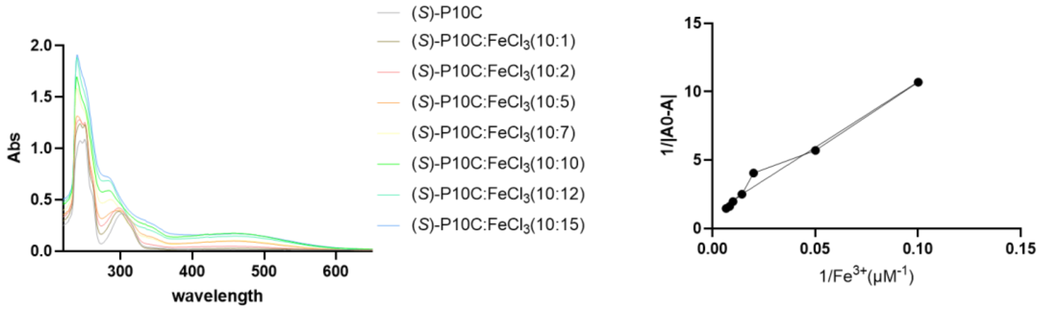

Figure S14. UV-visible spectra of (*S*)-P10C (100 μM) in the presence of increasing concentrations of $FeCl_3$ (from 10 μM to 150 μM) in EtOH.

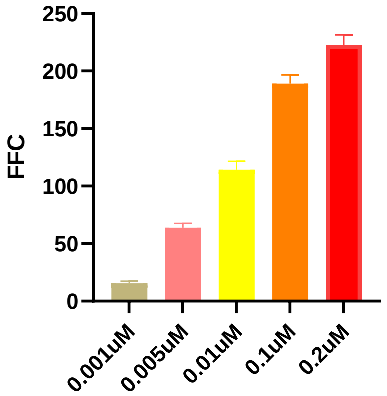

Figure S15. Flow cytometry analysis of *M. smegmatis* treated with N14G-Fe in different concentrations (0.001 μM, 0.005 μM, 0.01 μM, 0.1 μM, and 0.2 μM) for 1 h.

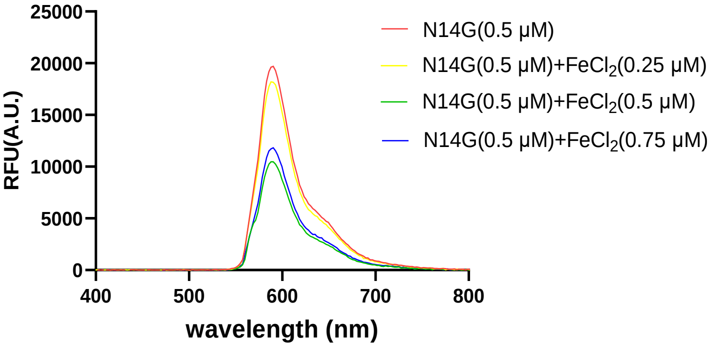

Figure S16. Fluorescent spectrum of N14G (0.5 μM) when combined with different concentration of Fe^{2+} ion in ddH_2O.

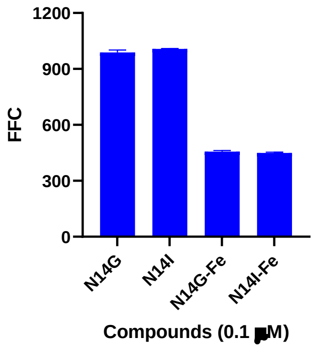

Figure S17. Flow cytometry analysis of *M. smegmatis* incubated with N14G-Fe (0.1 μM) and N14I-Fe (0.1 μM) for 1 h.

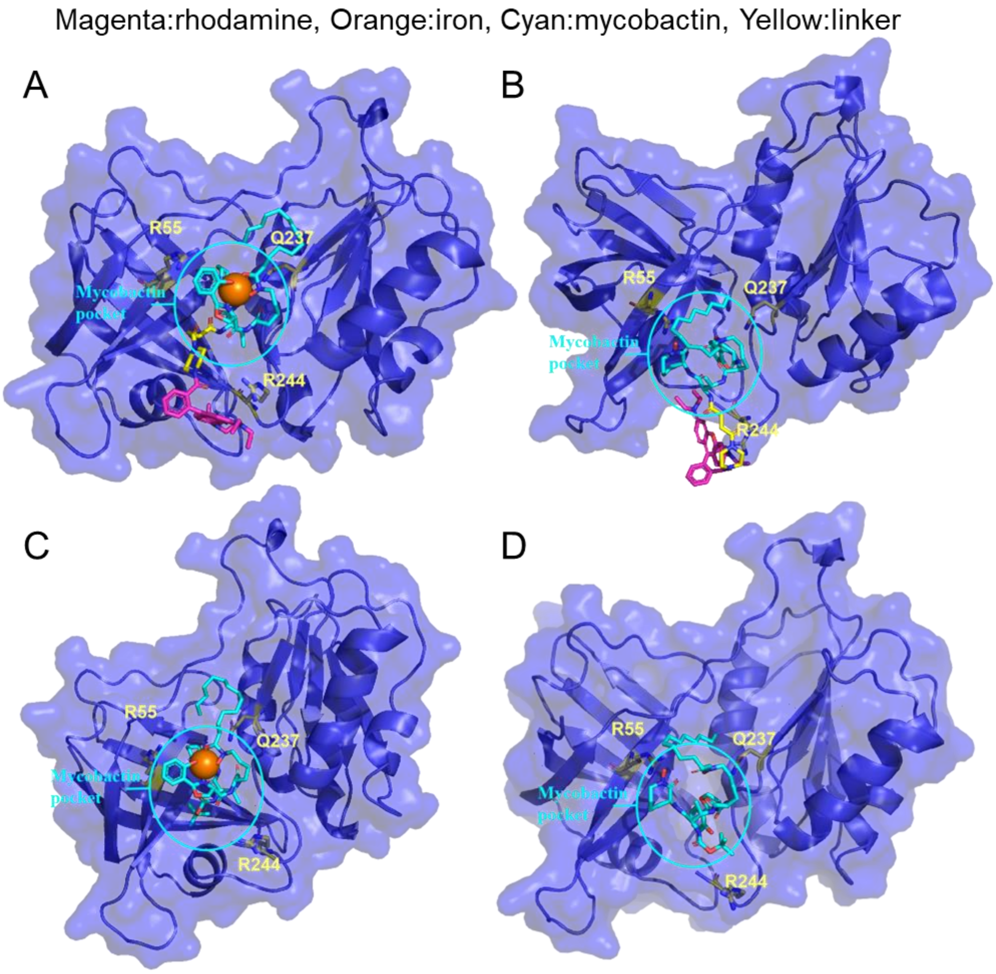

Figure S18. Docking studies of compound N14G-Fe (A), N14G (B), (*S*)-P10C-Fe (C), and (*S*)-P10C (D) with the mycobactin pocket of SID (PDB code: 6TEK). Docking was performed with MOE, and images were generated with Pymol. Three conserved residues lining the mycobactin pocket highlighted in yellow.

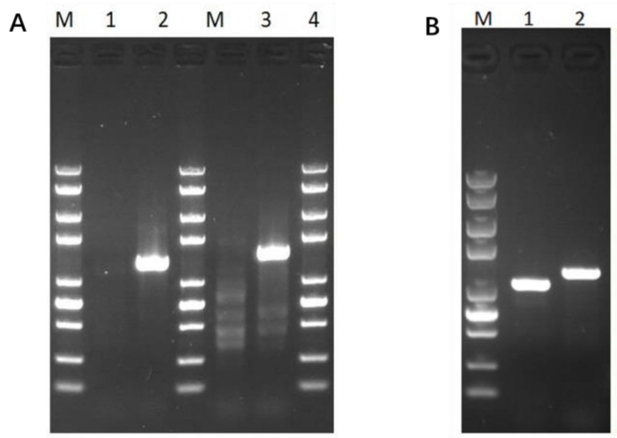

Figure S19. Verification of the construction of knockout mutant strain ΔIrtAB *M. smegmatis* and complemented strain ΔIrtAB::IrtAB *M. smegmatis*. (A) PCR products from wild-type *M. smegmatis* and knockout mutant clones using primers LYZFP/LYZRP and RYZFP/RYZRP. Lane Marker, DNA ladder; Lane 1 and 3, wild-type *M. smegmatis* using primers LYZFP/LYZRP and RYZFP/RYZRP, respectively; Lane 2 and 4, knockout mutant clones using primers LYZFP/LYZRP and RYZFP/RYZRP, respectively. (B) PCR products from complemented mutant clones using primers JD361FP-1/JD361RP-1 and JD361FP-2/JD361RP-2. Lane Marker, DNA ladder; Lane 1 and 2, complemented mutant clones using primers JD361FP-1/JD361RP-1 and JD361FP-2/JD361RP-2, respectively.

LYZFP: GGAACCTTCCTGTGGCTGATCGT;
LYZRP: GTGGACCTCGACGACCCTAG;
RYZFP: TGGATCTCTCCGGCTTCACC;
RYZRP: GGTTCCGACGACAGCGACTGCA;
JD361FP-1: GTGGCAGCGAGGACAACTTG;
JD361RP-1: AGCACGAAGCTCACGTCGTCGA;
JD361FP-2: TGAGCGAACTGACGCCTGCCAT;
JD361RP-2: CTAGCCACCTGACGTCGGG.

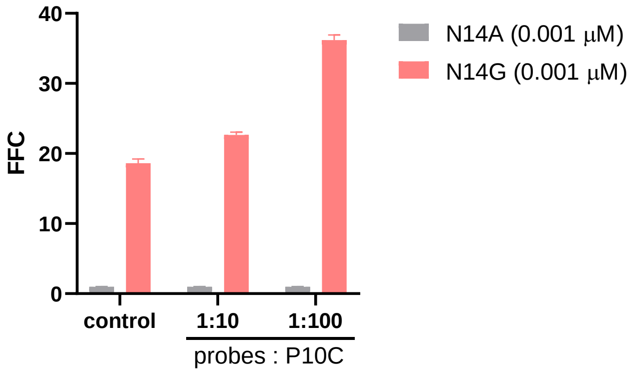

Figure S20. Flow cytometry analysis of wild-type *M. smegmatis* incubated with N14A (0.001 μM) and N14G (0.001 μM) in presence of excess of P10C (0.01 μM and 0.1 μM) for 1 h.

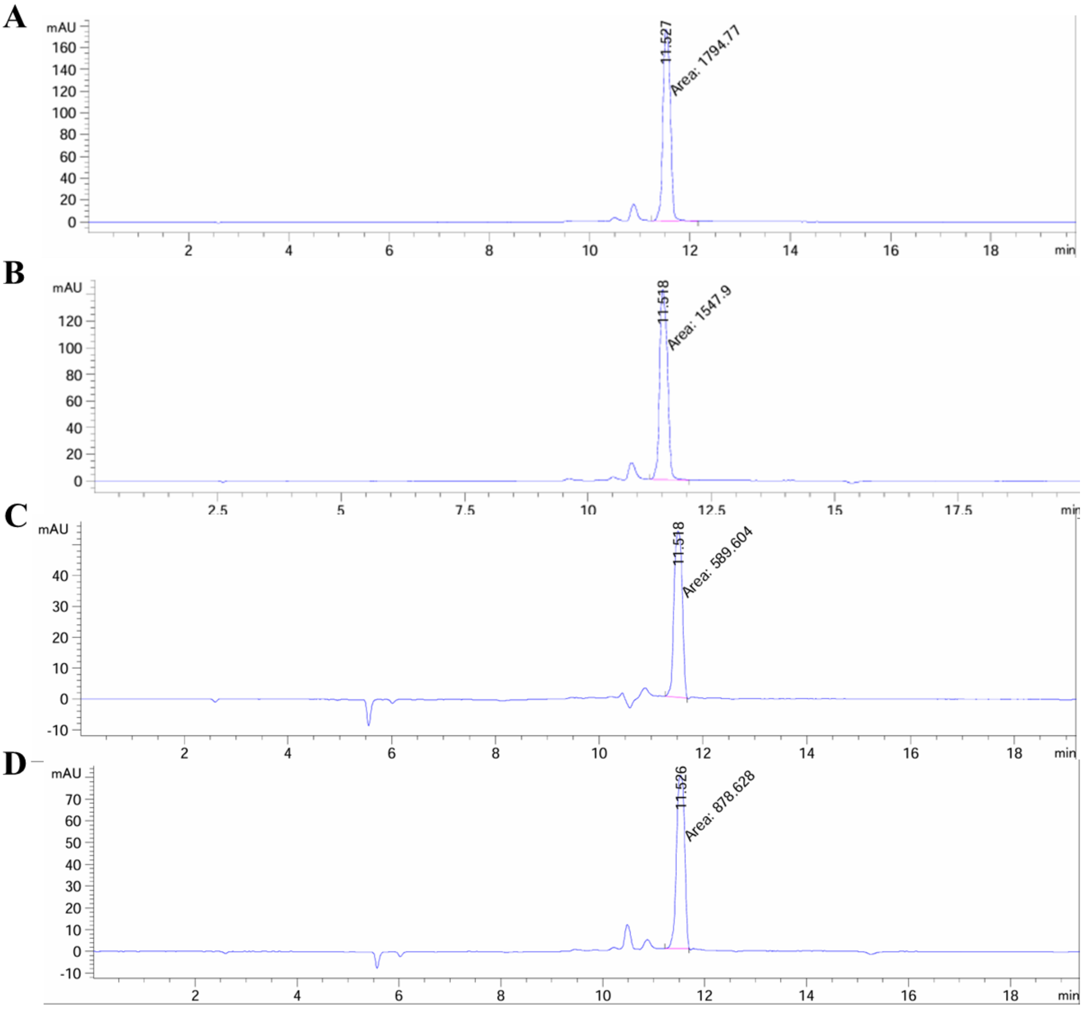

Peak area of N14G in image A was 1794.7.
Peak area of N14G in image B was 1547.9.
Peak area of N14G in image C was 589.6.
Peak area of N14G in image D was 878.628.
Machine: Agilent Technologies 1260 Infinity.
C18 column: HPLC Micsphere-C18, particle size 5 μm, dimensions 4.6 mm×250 mm.
Flow rate: 1 mL/min
Elution gradient for analysis:
0 min to 8 min: H_2O (+ TFA, 95%) / MeOH (5%) to H_2O (+TFA, 5%) / MeOH (95%);
8 min to 14 min: H_2O (+TFA, 5%) / MeOH (95%);
14 min to 16 min: H_2O (+ TFA, 5%) / MeOH (95%) to H_2O (+TFA, 95%) / MeOH (5%);
16 min to 20 min: H_2O (+TFA, 95%) / MeOH (5%).
Detection wavelength: 565 nm

Figure S21. Quantitative analysis by analytical HPLC about fluorescence enhancement in presence of excess of P10C. A: *M. smegmatis* was incubated with N14G (5 μM) for 1 h, then the supernatant fluorescence was analyzed. B: *M. smegmatis* was incubated with N14G (5 μM) and P10C (5 μM) for 1 h, then the supernatant fluorescence was analyzed. C: *M. smegmatis* was incubated with N14G (5 μM) for 1 h, then the lysate

fluorescence was analyzed. D: *M. smegmatis* was incubated with N14G (5 μM) and P10C (5 μM) for 1 h, then the lysate fluorescence was analyzed.

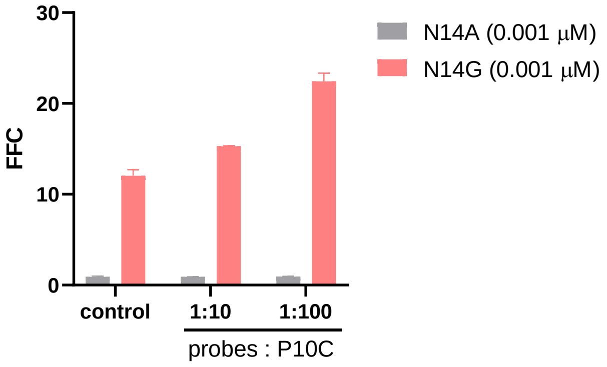

Figure S22. Flow cytometry analysis of ΔIrtAB *M. smegmatis* incubated with N14A (0.001 μM) and N14G (0.001 μM) in presence of excess of P10C (0.01 μM and 0.1 μM) for 1 h.

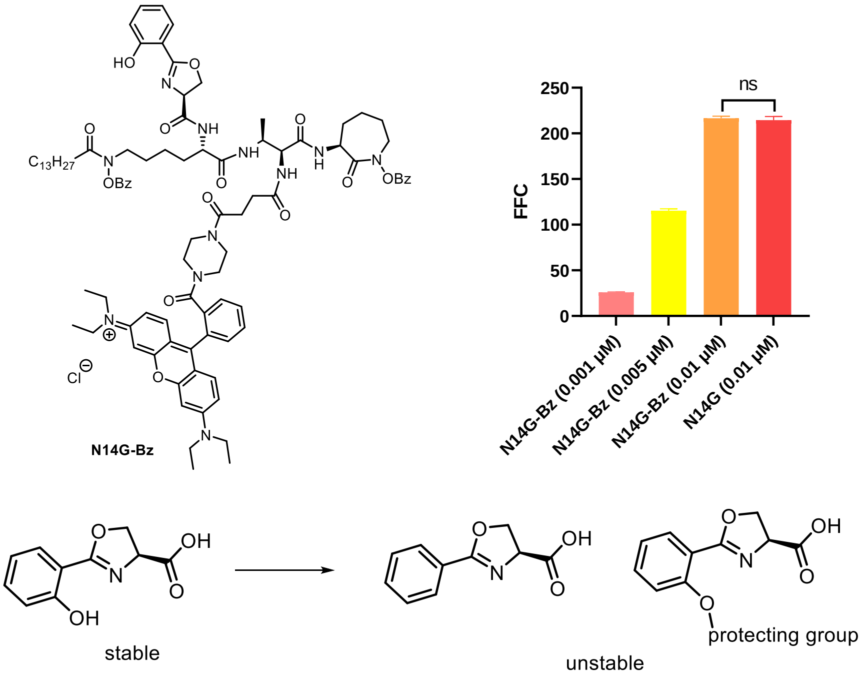

Figure S23. Structure of N14G-Bz and flow cytometry analysis of *M. smegmatis* incubated with N14G-Bz (0.001 μM, 0.005 μM, and 0.01 μM, respectively) for 1 hour.

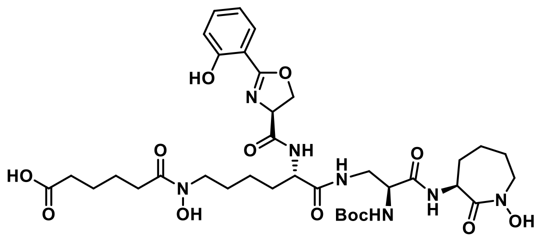

Figure S24. Structure of synthesized cMbT

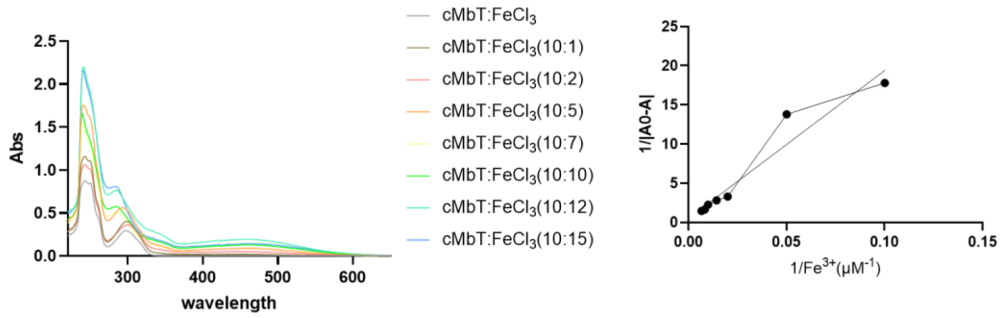

Figure S25. UV-visible spectra of cMbT (100 μM) in the presence of increasing concentrations of $FeCl_3$ (from 10 μM to 150 μM) in EtOH.

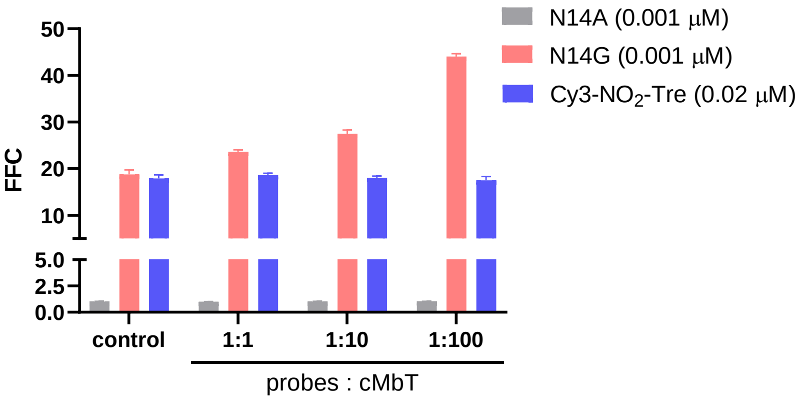

Figure S26. Flow cytometry analysis of *M. smegmatis* incubated with N14A (0.001 μM), N14G (0.001 μM), and Cy3-NO_2-Tre (0.02 μM), respectively, in the presence of excess of cMbT with different concentrations for 1 h.

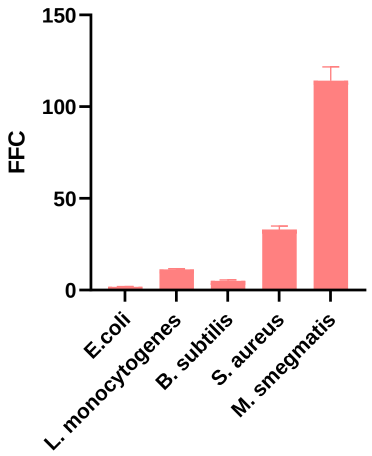

Figure S27. Flow cytometry analysis of *B. subtilis*, *E. coli*, *S. aureus*, *L. monocytogenes,* and *M. smegmatis*. All bacteria were treated with N14G-Fe (0.01 μM) for 1 h.

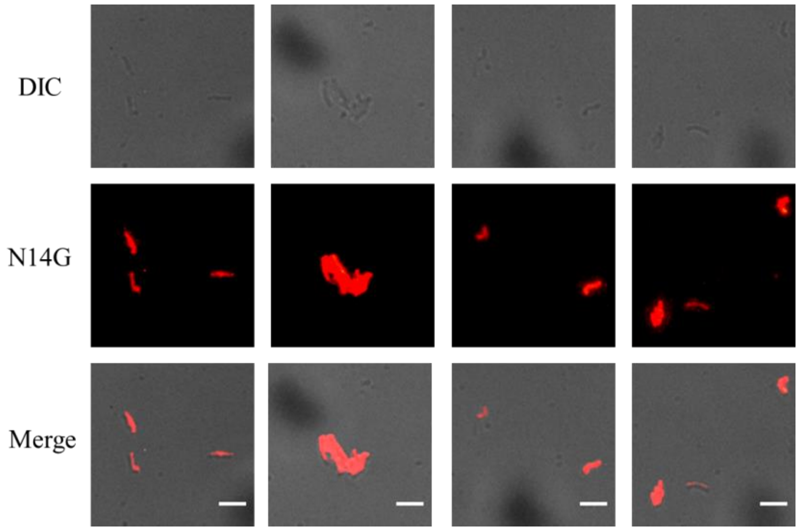

Figure S28. Imaging of *M. bovis* BCG with N14G. Freshly cultured *M. bovis* BCG were treated with N14G (0.1 μM) for 10 min, followed by centrifugation at 3000 g for 10 min. The bacteria were then washed with PBS buffer, resuspended in PBS buffer and imaged using a fluorescent confocal microscope. Scale bars, 5 μm.

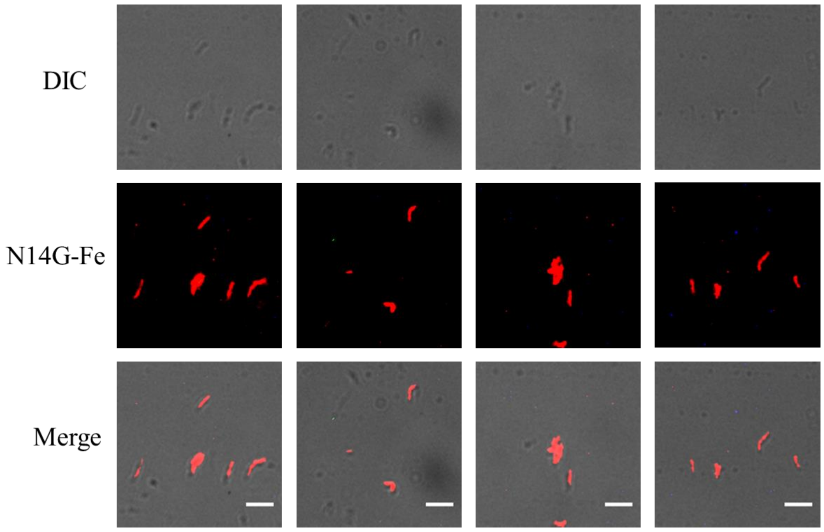

Figure S29. Imaging of *M. bovis* BCG with N14G-Fe. Freshly cultured *M. bovis* BCG were treated with N14G-Fe (0.1 μM) for 10 min, and then directly imaged using a fluorescent confocal microscope. Scale bars, 5 μm.

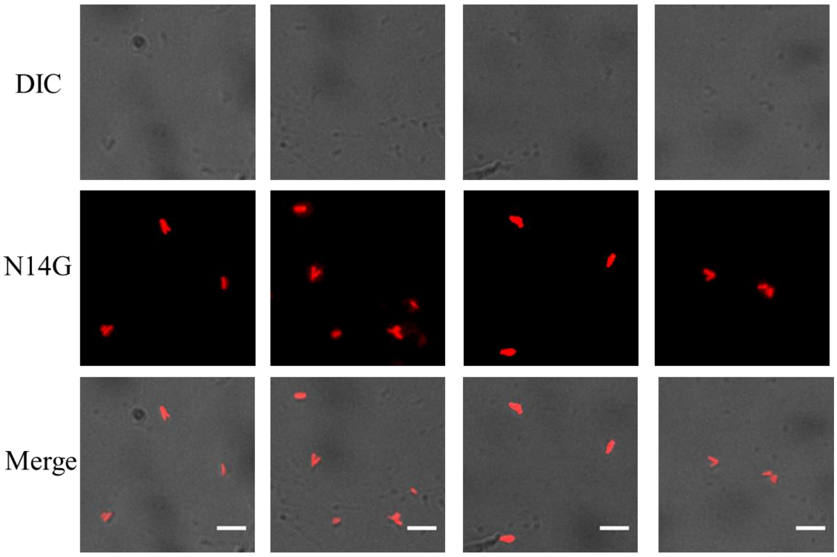

Figure S30. Imaging of H37Rv with N14G. Freshly cultured H37Rv were treated with N14G (0.1 μM) for 10 min, followed by centrifugation at 3000 g for 10 min. The bacteria were then washed with PBS buffer, resuspended in PBS buffer and imaged using a fluorescent confocal microscope. Scale bars, 5 μm.

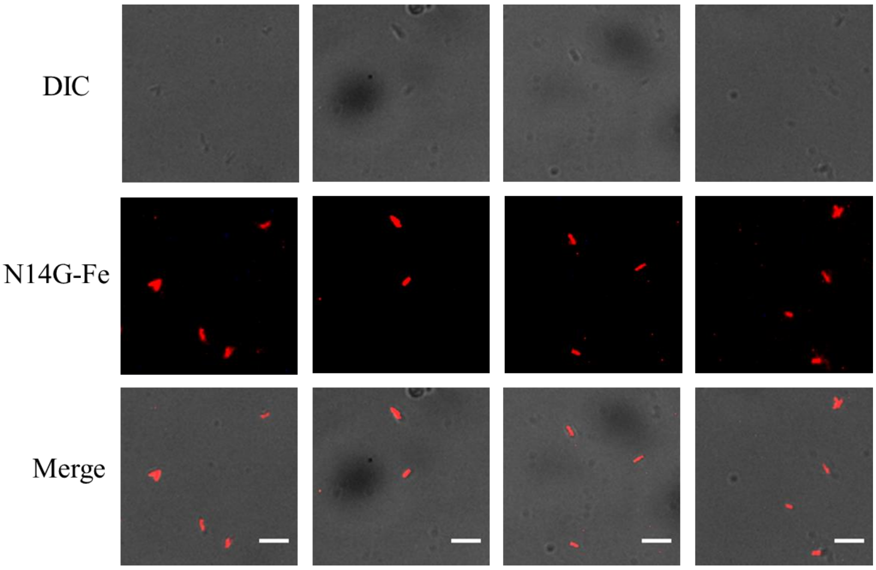

Figure S31. Imaging of H37Rv with N14G-Fe. Freshly cultured H37Rv were treated with N14G-Fe (0.1 μM) for 10 min, and then directly imaged using a fluorescent confocal microscope. Scale bars, 5 μm.

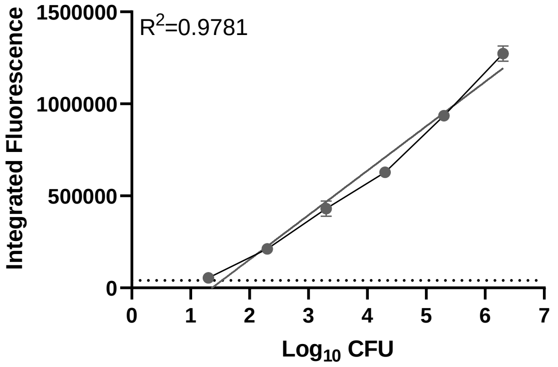

Figure S32. Correlation of integrated fluorescence and the number of H37Rv in culture medium. H37Rv were imaged after incubation with N14G (1.0 μM) for 1 h. Horizontal lines show the mean integrated fluorescence of control samples lacking H37Rv. ImageJ was used to calculated the integrated fluorescence.

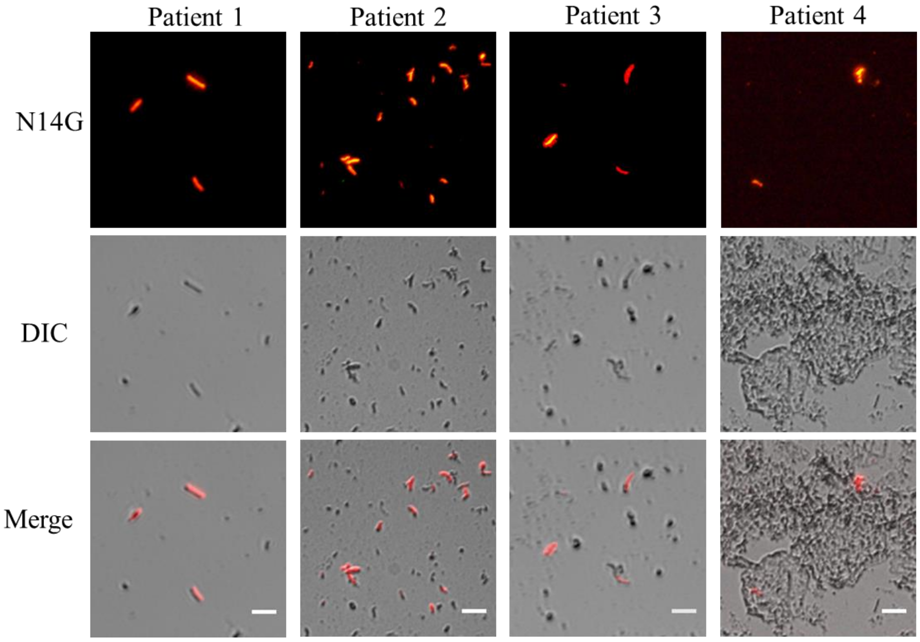

Figure S33. Images of sputum samples of patients 1–4 with TB treated with N14G (0.1 μM). Sputum samples were decontaminated with a NALC/NaOH mixture and then treated with N14G for 24 h. Images were collected in the brightfield (DIC) and N14G (red fluorescence) channels. “Merge” represents merged images from the above channels. Scale bars, 5 μm.

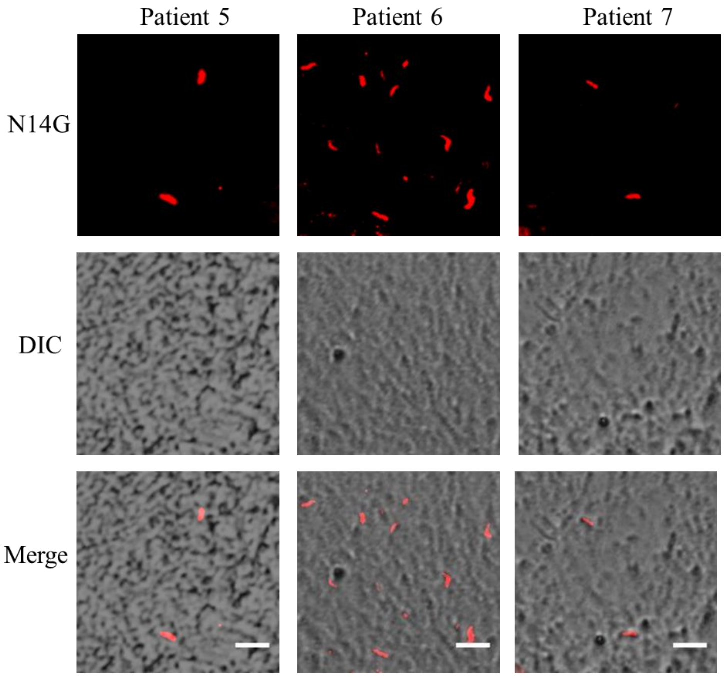

Figure S34. Images of sputum samples of patients 5–7 with TB treated with N14G (0.1 μM). Sputum samples were decontaminated with a NALC/NaOH mixture and then treated with N14G for 10 min. Images were collected in the brightfield (DIC) and Cy3 (red fluorescence) channels. “Merge” represents merged images from the above channels. Scale bars, 5 μm.

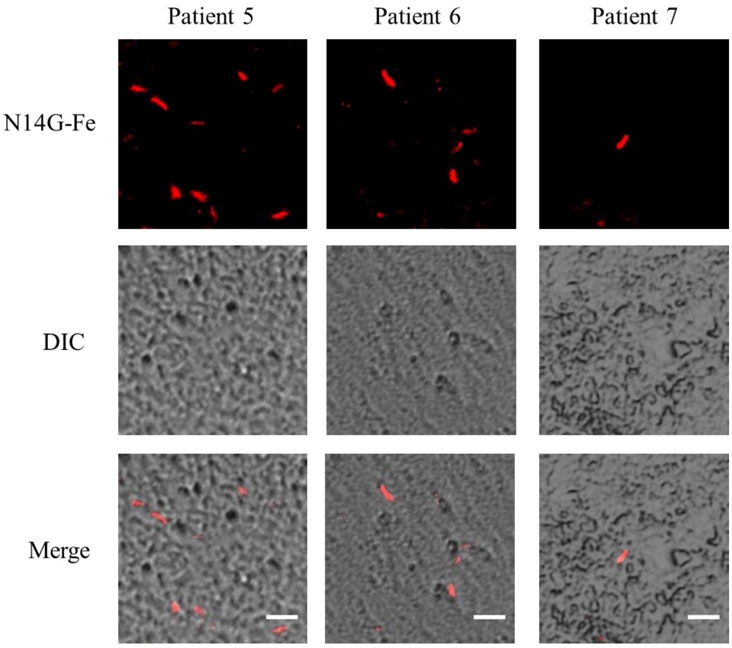

Figure S35. Images of sputum samples of patients 5–7 with TB treated with N14G-Fe (0.1 μM). Sputum samples were decontaminated with a NALC/NaOH mixture and then treated with N14G-Fe for 10 min. Images were collected in the brightfield (DIC) and Cy3 (red fluorescence) channels. “Merge” represents merged images from the above channels. Scale bars, 5 μm.

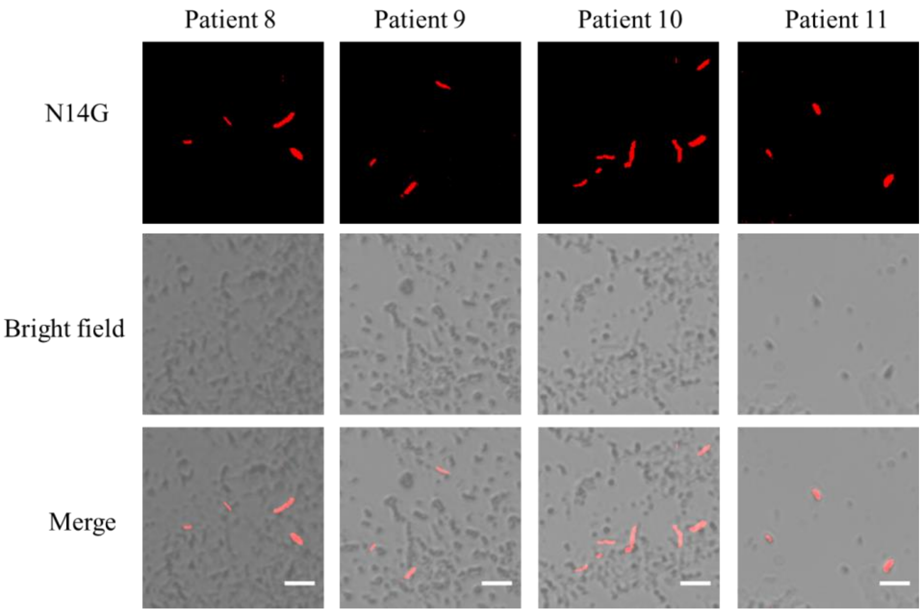

Figure S36. Images of sputum samples of patients 8–11 with TB treated with N14G (0.1 μM). Sputum samples were decontaminated with a NALC/NaOH mixture and then treated with N14G for 10 min. Images were collected in the brightfield (DIC) and Cy3 (Red fluorescence) channels. “Merge” represents merged images from the above channels. Scale bars, 5 μm.

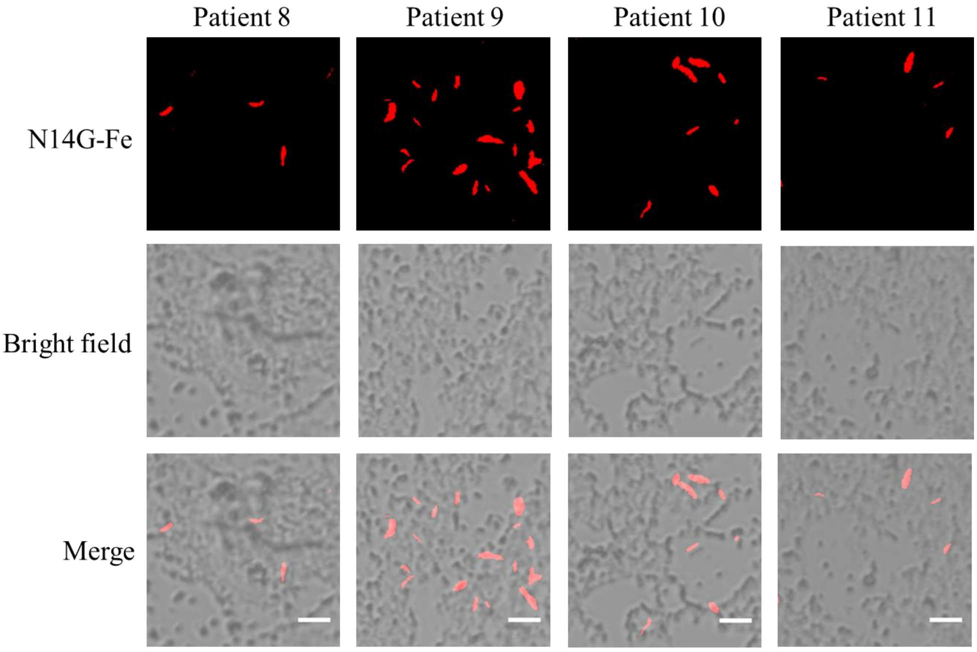

Figure S37. Images of sputum samples of patients 8–11 with TB treated with N14G-Fe (0.1 μM). Sputum samples were decontaminated with a NALC/NaOH mixture and then treated with N14G-Fe for 10 min. Images were collected in the brightfield (DIC) and Cy3 (red fluorescence) channels. “Merge” represents merged images from the above channels. Scale bars, 5 μm.

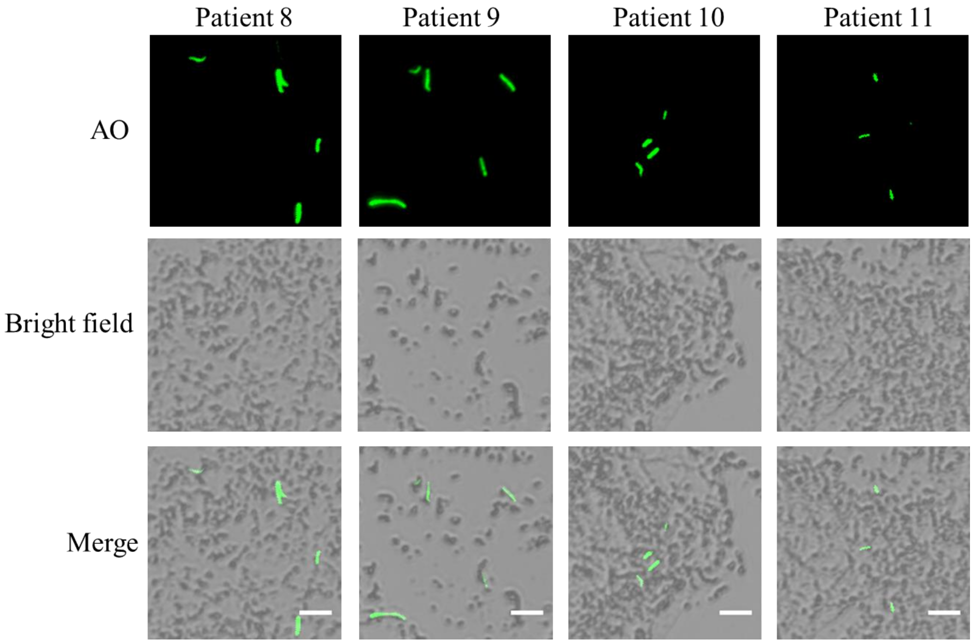

Figure S38. Images of sputum samples of patient 8–11 with TB treated with standard AO staining method. Images were collected in the brightfield (DIC) and FITC (green fluorescence) channels. “Merge” represents merged images from the above channels. Scale bars, 5 μm.

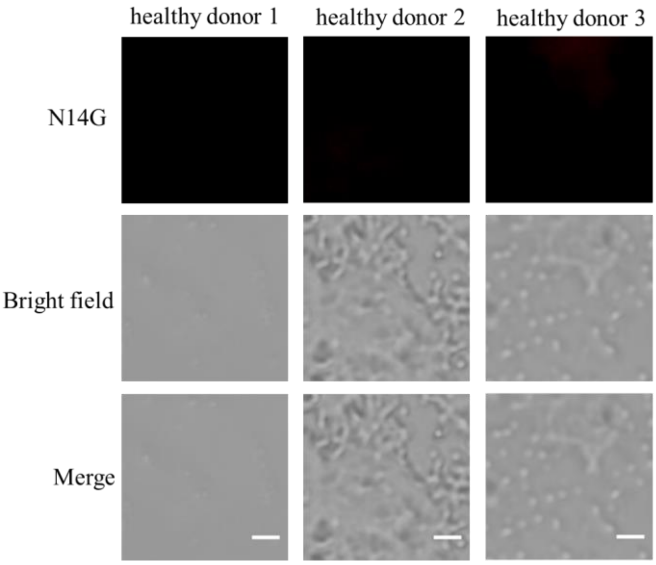

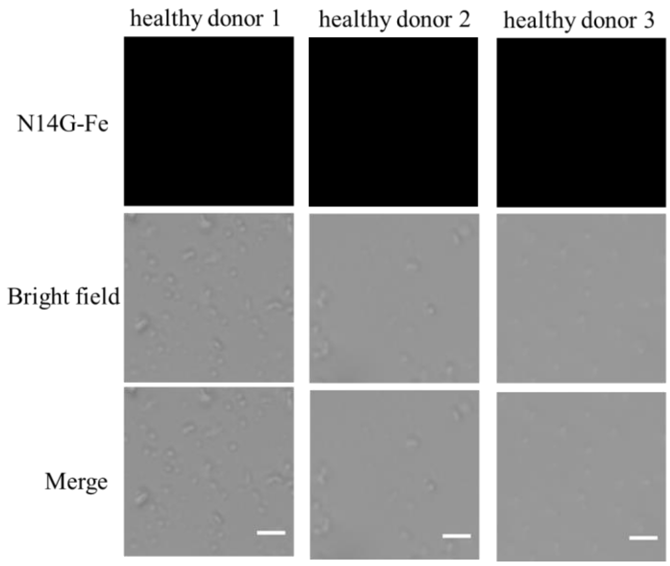

Figure S39. Images of sputum samples of healthy donors treated with N14G or N14G-Fe (0.01 μM). Sputum samples were decontaminated with a NALC/NaOH mixture and then treated with N14G or N14G-Fe for 10 min. Images were collected in the brightfield (DIC) and Cy3 (Red fluorescence) channels. “Merge” represents merged images from the above channels. Scale bars, 5 μm.

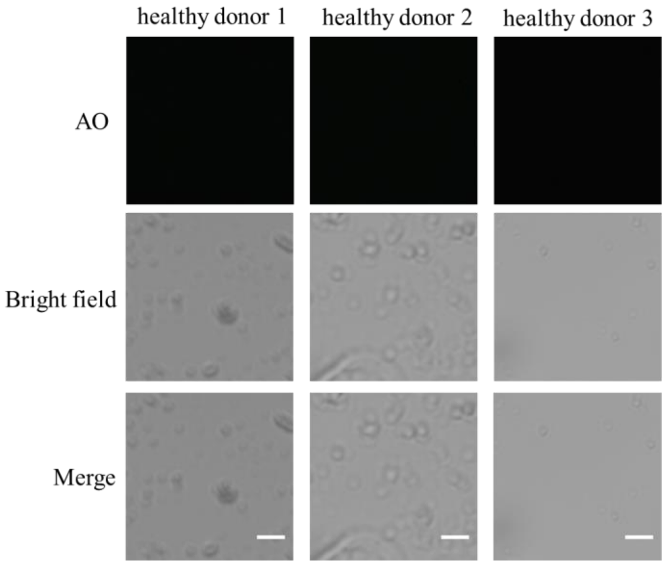

Figure S40. Images of sputum samples of healthy donors treated with standard AO staining method. Images were collected in the brightfield (DIC) and FITC (green fluorescence) channels. “Merge” represents merged images from the above channels. Scale bars, 5 μm.

II. Experimental Procedures of Chemistry

General experimental for synthesis

All chemicals were purchased as reagent grade and used without further purification unless otherwise noted. Analytical thin-layer chromatography (TLC) plates, were purchased from Qingdao Haiyang Chemical and detected by UV in 254 nm. Flash chromatography was performed with Sepaflash columns (silica gel column and reverse-phase C18 column) produced by Santai Technologies. Automatic liquid chromatography−mass spectrometry (LC-MS) analysis was performed on a Waters SQ Advantage mass spectrometer equipped with an ultraperformance LC (UPLC) system. The ion source was electrospray ionization (ESI). Proton nuclear magnetic resonance (^{1}H NMR), fluorine nuclear magnetic resonance (^{19}F NMR), and carbon nuclear magnetic resonance (^{13}C NMR) spectroscopy were performed on Bruker Advance 400M NMR, 600M NMR, and 800M NMR spectrometers.

2.1 Synthesis of mycobactin analogues P10C-16 and P10C

In this article, our previously reported general procedures[1] are further optimized for scaled-up synthesis of **P10C-16** and **P10C**, and significantly decrease the racemization to prepare various regarding chiral intermediates and the final compounds.

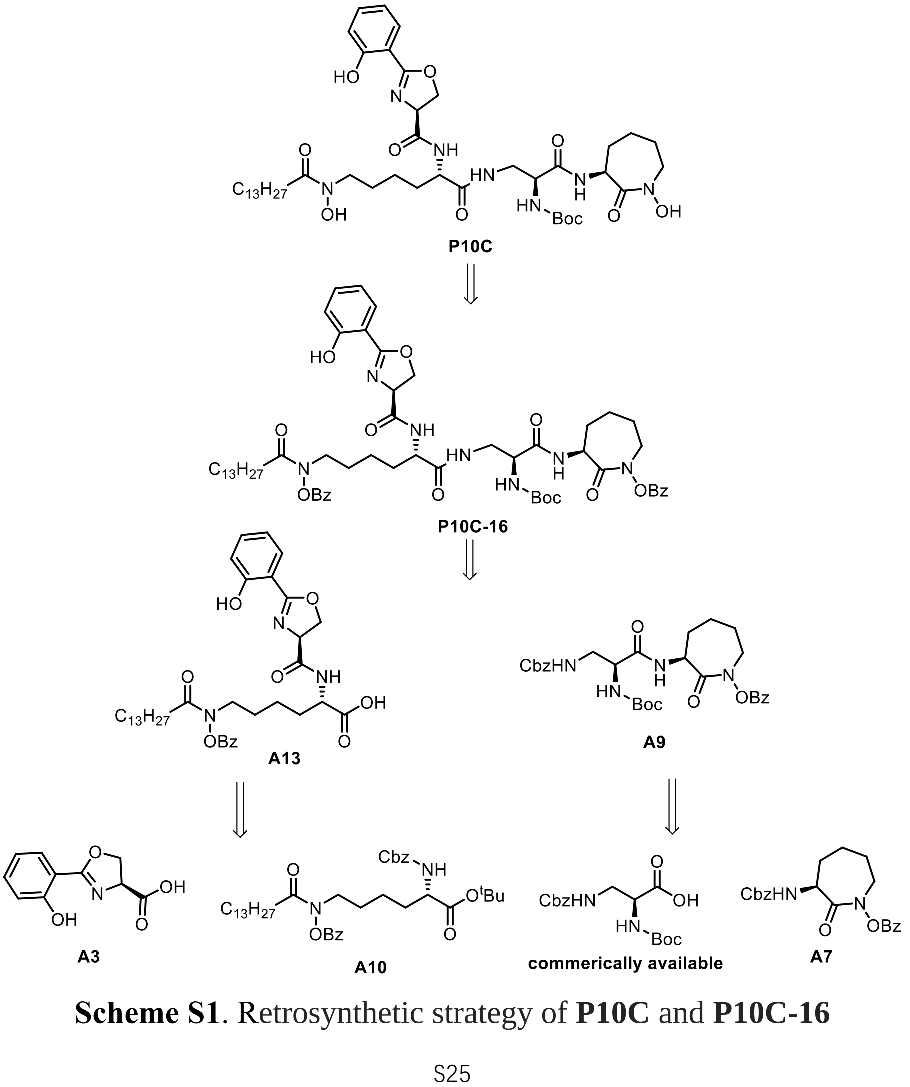

Scheme S1. Retrosynthetic strategy of **P10C** and **P10C-16**

2.1.1 Synthesis of building block A3

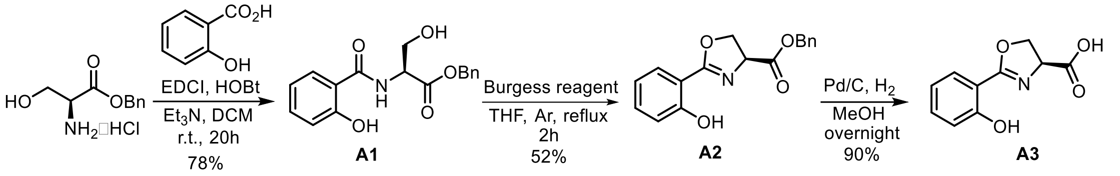

Scheme S2. Synthesis of building block **A3**

To a stirred solution of salicylic acid (2.76 g, 20.0 mmol) and L-serine benzyl ester hydrochloride (5.54 g, 24.0 mmol) in dry DCM (30 mL) was added 1-hydroxybenzotriazole (HOBt) (4.06 g, 30.0 mmol), EDCI (5.74 g, 30.0 mmol) and triethylamine (Et_3N) (8.08 g, 80.0 mmol), the mixture was stirred at r.t. for 20 h. The reaction was quenched with ice water (50.0 mL). The organic layers were evaporated in vacuo and purified via silica gel column chromatography (PE:EA/100:0 to PE:EA/60:40) to afford intermediate **A1**. R_f= 0.2 (PE:EA/3:1), colorless oil, yield 78%, 4.92 g.

^{1}H NMR (400 MHz, $CDCl_3$) δ 11.94 (s, 1H), 7.49 (dd, *J* = 8.0, 1.6 Hz, 1H), 7.44 – 7.39 (m, 1H), 7.39 – 7.33 (m, 5H), 7.32 – 7.28 (m, 1H), 6.98 (d, *J* = 8.4 Hz, 1H), 6.86 (t, *J* = 7.6 Hz, 1H), 5.30 – 5.21 (m, 2H), 4.90 – 4.85 (m, 1H), 4.12 (dd, *J* = 11.3, 3.5 Hz, 1H), 4.03 (dd, *J* = 11.3, 3.3 Hz, 1H).

^{13}C NMR (101 MHz, $CDCl_3$) δ 170.2, 170.1, 161.6, 135.0, 134.8, 128.8, 128.7, 128.3, 126.0, 118.9, 118.6, 113.7, 67.9, 63.1, 54.7.

HRMS (ESI-MS) *m/z*: $[M+H]^+$; Calcd for $C_{17}H_{18}NO_5$ 316.1179; Found 316.1176.

To a stirred solution of **A1** (4.72 g, 15.0 mmol) in dry THF (20 mL) was added Burgess's reagent [(methoxycarbonylsulfamoyl)triethylammonium hydroxide, inner salt, 8 g, 22.5 mmol, 1.5 equiv.]. After being refluxed for 2 h under Ar atmosphere, the reaction mixture was diluted with EA (50 mL), washed with H_2O (20 mL) and brine (20 mL), dried over Na_2SO_4. The **A2** was obtained after purification by silica gel column chromatography (PE:EA/100:0 to PE:EA/70:30). R_f = 0.5 (PE:EA/3:1), white solid, yield 52%, 2.3 g.

^{1}H NMR (400 MHz, $CDCl_3$) δ 11.70 (s, 1H), 7.67 (dd, *J* = 7.8, 1.8 Hz, 1H), 7.44 – 7.33 (m, 7H), 7.04 (dd, *J* = 8.4, 1.1 Hz, 1H), 6.89 (td, *J* = 7.6, 1.1 Hz, 1H), 5.25 (d, *J* = 3.7 Hz, 2H), 5.01 (dd, *J* = 10.4, 7.5 Hz, 1H), 4.68 (dd, *J* = 8.8, 7.5 Hz, 1H), 4.56 (dd, *J* = 10.5, 8.8 Hz, 1H).

^{13}C NMR (101 MHz, $CDCl_3$) δ 170.3, 167.7, 160.1, 135.3, 134.1, 128.8, 128.6, 128.5, 128.4, 118.9, 117.0, 110.2, 68.9, 67.5, 67.4.

HRMS (ESI-MS) *m/z*: $[M+H]^+$; Calcd for $C_{17}H_{16}NO_4$ 298.1074; Found 298.1072.

To a solution of the intermediate **A2** (2 g, 7.0 mmol) in MeOH (20 mL) was added 10% Pd/C (200 mg, 10% w/w). The mixture was stirred under H_2 atmosphere at r.t. overnight. After the reaction completed, the solution was filtrated, evaporated in vacuo and lyophilized to get **A3**. Pink solid, yield 90%, 1.3 g.

The ^{1}H, ^{13}C NMR, and MS of **A3** was identical to previous literature.[1]

2.1.2 Synthesis of building block A9

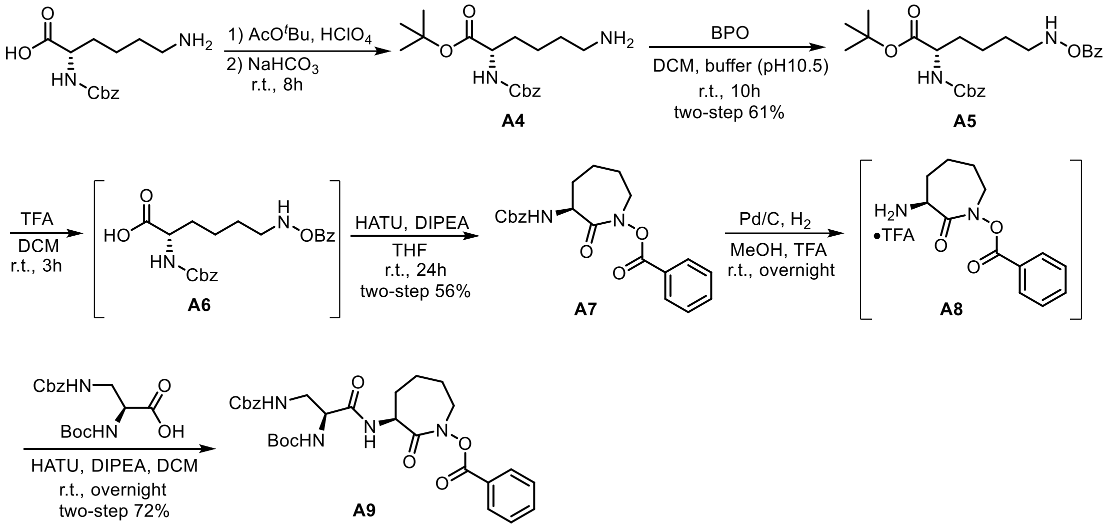

Scheme S3. Synthesis of building block **A9**

The synthesis of intermediates **A4** and **A5** were reported by our previously general procedures.[1]

(*S*)-3-(((benzyloxy)carbonyl)amino)-2-oxoazepan-1-yl benzoate

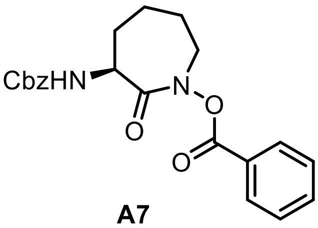

A7

To a stirred solution of the intermediate **A5** (1.8 g, 4.0 mmol) in DCM (20 mL) was added TFA (10 mL) dropwise at ice-bath water. Then the solution was stirred at r.t. until the **A5** disappeared in TLC detection and then evaporated in vacuo to completely removal of solvent. The residue was dissolved in EA (30 mL) and the 1 M $NaHCO_3$ (aq.) was added to adjust pH = 6. The organic layer was washed with brine (15 mL), dried over anhydrous Na_2SO_4 and evaporated in vacuo to get **A6** as colorless oil, that was used directly in the next step.

In a 250 mL round bottomed flask, HATU (20.0 mmol, 5.0 equiv.), DIPEA (28.0 mmol, 7.0 equiv.) and THF (100 mL) were added successively. Crude oil was dissolved in dry THF (50 mL) and then was placed in a 60 mL constant pressure drip funnel under r.t., and the drip rate was controlled to maintain a drip time of about 10 h. The reaction was further carried out under r.t. and monitored by LC-MS analysis, until the reaction was completed overnight. The reaction mixture was filtered and the filtrate was evaporated in vacuo, then EtOAc (50 mL) was added and sequentially washed with 1 M HCl (aq. 30 mL×2), H_2O (30 mL), brine (30 mL), dried over Na_2SO_4, and purified by silica gel column chromatography (PE:EA/100:0 to PE:EA/60:40) to afford **A7**. R_f = 0.3

(PE:EA/3:1), yellow oil, two-step yield 56%, 790 mg.
The ^{1}H, ^{13}C NMR and MS of **A7** were identical to previous literature.[1]

(*S*)-3-((*S*)-3-(((benzyloxy)carbonyl)amino)-2-((*tert*-butoxycarbonyl)amino)propanamido)-2-oxoazepan-1-yl benzoate

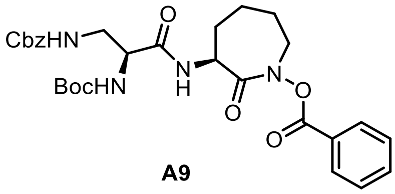

A9

To a solution of the intermediate **A7** (764 mg, 2.0 mmol) in MeOH (15 mL) was added 10% Pd/C (76 mg, 10% w/w) and TFA (150 μL). The mixture was stirred under H_2 atmosphere at r.t. overnight. After the reaction completed, the solution was filtrated and evaporated in vacuo to get **A8** as colorless oil, that was used directly without further purification.
The intermediate **A8** and carboxy acid (676 mg, 2.0 mmol, 1.0 equiv.) were dissolved in DCM (10 mL), followed by the addition of HATU (836 mg, 2.2 mmol, 1.1 equiv.) and DIPEA (774 mg, 6.0 mmol, 3.0 equiv.). After stirring at r.t. overnight, the reaction was diluted with EtOAc (30 mL) and washed sequentially with 1 M HCl (aq., 15 mL), H_2O (15 mL) and brine (15 mL). The organic layer was dried over anhydrous Na_2SO_4, filtered, and concentrated in vacuo. The mixture was purified by silica gel column chromatography (PE:EA/100:0 to PE:EA/50:50) to afford intermediate **A9**. R_f = 0.4 (PE:EA/1:1), white solid, two-step yield 72%, 817 mg.
The ^{1}H, ^{13}C NMR and MS of **A9** were identical to previous literature.[1]

2.1.3 Synthesis of building block A13

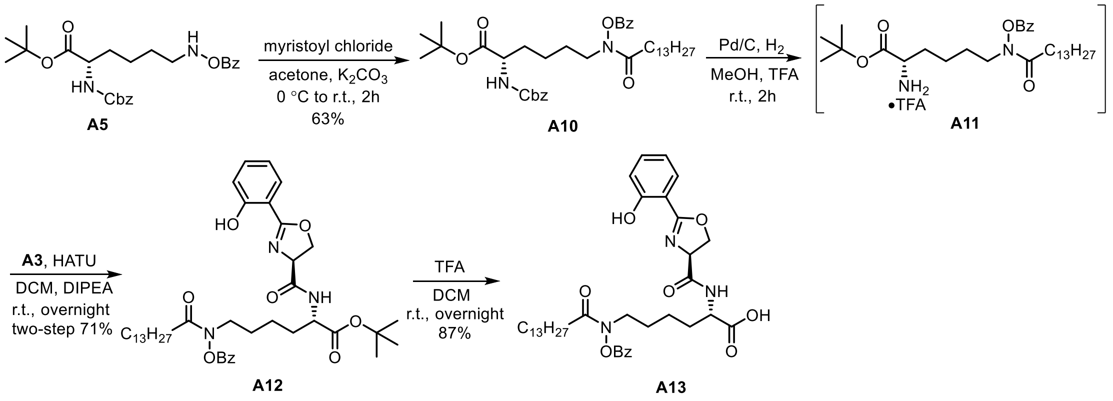

Scheme S4. Synthesis of building block **A13**

***Tert*-butyl *N*⁶-(benzoyloxy)-*N*²-((benzyloxy)carbonyl)-*N*⁶-tetradecanoyl-L-lysinate**

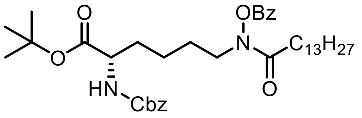

A10

To a solution of the **A5** (1.8 g, 4.0 mmol) and K_2CO_3 (1.1 g, 8.0 mmol, 2.0 equiv.) in

dry acetone (15 mL) was added myristoyl chloride (1.2 g, 4.8 mmol, 1.2 equiv.) dropwise at 0 °C. Then the mixture was stirred for additional 2 h at r.t. After the reaction completed, the solution was filtrated and concentrated in vacuo. The mixture was purified by silica gel column chromatography (PE:EA/100:0 to PE:EA/75:25) to afford intermediate **A10**. R_f = 0.6 (PE:EA/3:1), colorless oil, yield 63%, 1.6 g.

^{1}H NMR (400 MHz, $CDCl_3$) δ 8.14 – 8.03 (m, 2H), 7.67 (t, *J* = 7.4 Hz, 1H), 7.52 (t, *J* = 7.7 Hz, 2H), 7.39 – 7.27 (m, 5H), 5.33 (d, *J* = 8.2 Hz, 1H), 5.09 (s, 2H), 4.23 (td, *J* = 7.6, 5.0 Hz, 1H), 3.80 (t, *J* = 7.2 Hz, 2H), 2.27 (t, *J* = 7.6 Hz, 2H), 1.88 – 1.78 (m, 1H), 1.70 – 1.59 (m, 5H), 1.44 (s, 9H), 1.29 – 1.20 (m, 22H), 0.88 (t, *J* = 6.8 Hz, 3H).

^{13}C NMR (101 MHz, $CDCl_3$) δ 171.5, 164.5, 155.9, 136.4, 134.5, 130.0, 128.9, 128.5, 128.1, 128.1, 126.9, 82.1, 66.9, 54.2, 32.4, 31.9, 29.7, 29.6 (×2), 29.5, 29.4, 29.2, 28.0, 26.7, 24.8, 24.4, 22.7, 22.2, 14.1.

HRMS (ESI-MS) *m/z*: $[M+H]^+$; Calcd for $C_{39}H_{59}N_2O_7$ 667.4317; Found 667.4310.

***Tert*-butyl *N*^{6}-(benzoyloxy)-*N*^{2}-((*S*)-2-(2-hydroxyphenyl)-4,5-dihydrooxazole-4-carbonyl)-*N*^{6}-tetradecanoyl- L -lysinate**

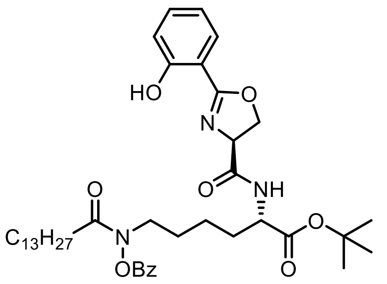

A12

To a solution of the intermediate **A10** (1.4 g, 2.2 mmol) in MeOH (15 mL) was added 10% Pd/C (140 mg, 10% w/w) and TFA (150 μL). The mixture was stirred under H_2 atmosphere at r.t. for 2 h. After the reaction completed, the solution was filtrated and evaporated in vacuo to get **A11** as colorless oil that was used directly without further purification.

The intermediate **A11** and **A3** (455 mg, 2.2 mmol, 1.0 equiv.) were dissolved in DCM (10 mL), followed by the addition of HATU (912 mg, 2.4 mmol, 1.1 equiv.) and DIPEA (852 mg, 6.6 mmol, 3.0 equiv.). After stirring at r.t. overnight, the reaction was diluted with EtOAc (30 mL) and washed sequentially with 1 M HCl (aq., 15 mL), H_2O (15 mL) and brine (15 mL). The organic layer was dried over anhydrous Na_2SO_4, filtered, and concentrated in vacuo. The mixture was purified by reverse-phase C18 column chromatography (H_2O:MeCN/95:5 to 5:95) to afford **A12**. Colorless oil, two-step yield 71%, 1.1 g.

^{1}H NMR (400 MHz, Acetone-*d*$_6$) δ 11.64 (s, 1H), 8.15 – 8.05 (m, 2H), 7.74 (t, *J* = 7.6 Hz, 1H), 7.70 – 7.62 (m, 2H), 7.62 – 7.52 (m, 2H), 7.43 (t, *J* = 7.8 Hz, 1H), 7.00 – 6.87 (m, 2H), 5.05 (t, *J* = 9.1 Hz, 1H), 4.75 – 4.62 (m, 2H), 4.43 – 4.32 (m, 1H), 3.87 – 3.73 (m, 2H), 2.29 (t, *J* = 7.5 Hz, 2H), 1.94 – 1.84 (m, 1H), 1.80 – 1.64 (m, 3H), 1.62 – 1.55 (m, 2H), 1.46 (s, 9H), 1.33 – 1.20 (m, 21H), 0.87 (t, *J* = 6.6 Hz, 3H).

^{13}C NMR (101 MHz, Acetone-*d*$_6$) δ 170.9, 169.7, 166.8, 159.9, 134.4, 133.9, 129.8, 129.0, 128.2, 118.8, 116.7, 110.3, 80.9, 69.4, 68.1, 52.9, 31.8 (×2), 31.3, 29.5 (×3), 29.3,

29.2, 29.0, 27.3, 24.3, 22.7, 22.5, 13.5.
HRMS (ESI-MS) *m/z*: $[M+H]^+$; Calcd for $C_{41}H_{60}N_3O_8$ 722.4375; Found 722.4371.

N^6-(benzoyloxy)-N^2-((*S*)-2-(2-hydroxyphenyl)-4,5-dihydrooxazole-4-carbonyl)-N^6-tetradecanoyl- L -lysine

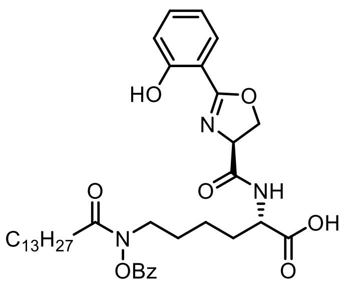

A13

To a stirred solution of the intermediate **A12** (1 g, 1.4 mmol) in DCM (20 mL) was added TFA (10 mL) dropwise at ice-bath water. Then the solution was stirred at r.t. until the **A12** disappeared in LC-MS detection and then evaporated in vacuo to completely removal of solvent. The mixture was purified by reverse-phase C18 column chromatography (H_2O: MeOH /95:5 to 5:95) to afford **A13**. Colorless oil, yield 87%, 809 mg.
^{1}H NMR (400 MHz, MeOD) δ 8.04 (d, *J* = 7.8 Hz, 2H), 7.67 (t, *J* = 7.6 Hz, 1H), 7.61 (d, *J* = 7.9 Hz, 1H), 7.51 (t, *J* = 7.8 Hz, 2H), 7.35 (t, *J* = 7.9 Hz, 1H), 6.92 – 6.80 (m, 2H), 5.02 (t, *J* = 9.0 Hz, 1H), 4.68 – 4.57 (m, 2H), 4.47 (dd, *J* = 9.6, 4.4 Hz, 1H), 3.87 – 3.75 (m, 2H), 2.27 (s, 2H), 2.02 – 1.91 (m, 1H), 1.85 – 1.75 (m, 1H), 1.73 – 1.63 (m, 2H), 1.61 – 1.45 (m, 4H), 1.31 – 1.16 (m, 20H), 0.88 (t, *J* = 6.7 Hz, 3H).
^{13}C NMR (101 MHz, MeOD) δ 173.7, 171.3, 167.0, 159.5, 134.3, 133.6, 129.6, 128.7, 128.1, 118.6, 116.3, 110.0, 68.9, 67.9, 52.1, 31.8, 31.7, 30.8, 29.4 (×3), 29.3, 29.2, 29.1, 29.0, 28.8, 26.4, 24.2, 22.8, 22.4, 13.1.
HRMS (ESI-MS) *m/z*: $[M+H]^+$; Calcd for $C_{37}H_{52}N_3O_8$ 666.3749; Found 666.3745.

2.1.4 Synthesis of MbT analogues P10C-16 and P10C

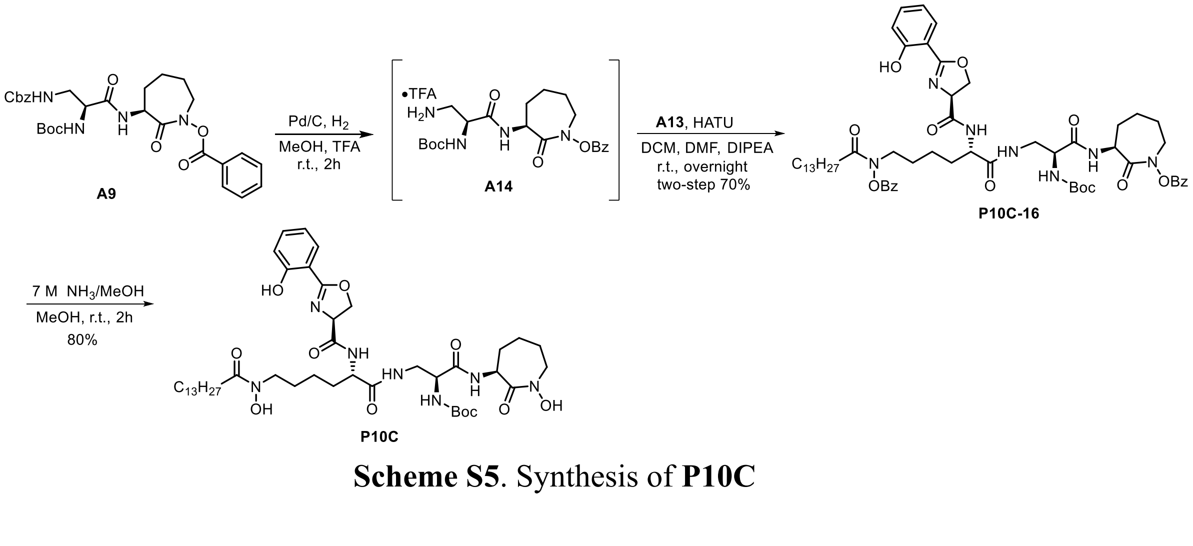

Scheme S5. Synthesis of **P10C**

(*S*)-3-((*S*)-3-((*S*)-6-(*N*-(benzoyloxy)tetradecanamido)-2-((*S*)-2-(2-hydroxypheny-l)

-4,5-dihydrooxazole-4-carboxamido)hexanamido)-2-((*tert*-butoxycarbonyl)amin-o)p-ropanamido)-2-oxoazepan-1-yl benzoate (P10C-16)

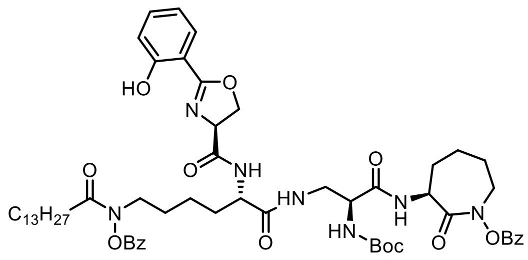

P10C-16

To a solution of the intermediate **A9** (681 mg, 1.2 mmol) in MeOH (15 mL) was added 10% Pd/C (68 mg, 10% w/w) and TFA (150 μL). The mixture was stirred under H_2 atmosphere at r.t. for 2 h. After the reaction completed, the solution was filtrated and evaporated in vacuo to get **A14** as colorless oil that was used directly without further purification.

The intermediate **A14** and **A13** (798 mg, 1.2 mmol, 1.0 equiv.) were dissolved in DCM (5 mL) and DMF (5 mL), followed by the addition of HATU (494 mg, 1.3 mmol, 1.1 equiv.) and DIPEA (464 mg, 3.6 mmol, 3.0 equiv.). After stirring at r.t. overnight, the reaction was diluted with EtOAc (20 mL) and washed sequentially with 1 M HCl (aq., 15 mL), H_2O (15 mL) and brine (15 mL). The organic layer was dried over anhydrous Na_2SO_4, filtered, and concentrated in vacuo. The mixture was purified by reverse-phase C18 column chromatography (H_2O:MeOH/95:5 to 5:95) to afford **P10C-16**. White solid, two-step yield 70%, 910 mg.

^{1}H NMR (800 MHz, DMSO-d_6) δ 11.78 (s, 1H), 8.43 (d, J = 8.0 Hz, 1H), 8.07 – 7.96 (m, 6H), 7.78 – 7.68 (m, 2H), 7.62 – 7.53 (m, 5H), 7.43 (t, J = 7.9 Hz, 1H), 6.99 – 6.88 (m, 3H), 4.98 (t, J = 8.8 Hz, 1H), 4.64 (t, J = 9.4 Hz, 1H), 4.57 (t, J = 9.3 Hz, 1H), 4.53 (t, J = 7.9 Hz, 1H), 4.27 – 4.15 (m, 2H), 4.05 (q, J = 7.1 Hz, 1H), 3.79 (d, J = 14.5 Hz, 1H), 3.72 – 3.58 (m, 2H), 3.40 – 3.35 (m, 1H), 3.27 – 3.19 (m, 1H), 2.20 (s, 2H), 1.91 (d, J = 11.6 Hz, 1H), 1.84 (d, J = 13.7 Hz, 1H), 1.80 – 1.67 (m, 4H), 1.64 – 1.44 (m, 6H), 1.35 (s, 9H), 1.27 – 1.14 (m, 22H), 0.84 (t, J = 7.2 Hz, 3H).

^{13}C NMR (201 MHz, DMSO-d_6) δ 171.6, 169.5, 165.7, 163.2, 159.0, 155.0, 134.3, 133.8, 129.5, 129.0, 128.9, 127.9, 126.5, 118.9, 116.4, 109.7, 78.3, 69.1, 67.1, 54.2, 52.5, 52.2, 51.2, 48.5, 31.4, 31.2, 30.1, 28.9, 28.9, 28.8, 28.7, 28.6, 28.3, 28.0, 27.9, 26.9, 25.7, 23.8, 22.4, 22.0, 13.8.

HRMS (ESI-MS) m/z: $[M+H]^+$; Calcd for $C_{58}H_{80}N_7O_{13}$ 1082.5806; found 1082.5772.

***Tert*-butyl ((*S*)-1-(((*S*)-1-hydroxy-2-oxoazepan-3-yl)amino)-3-((*S*)-2-((*S*)-2-(2-hy-droxyphenyl)-4,5-dihydrooxazole-4-carboxamido)-6-(*N*-hydroxytetradecanamid-o)hexanamido)-1-oxopropan-2-yl)carbamate (P10C).**

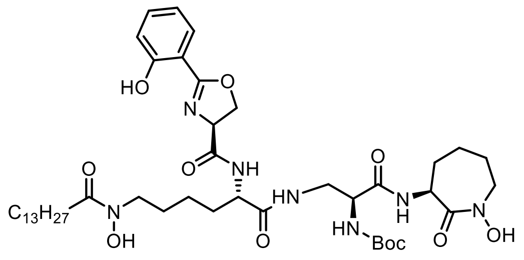

P10C

The **P10C-16** (50 mg, 0.045 mmol) was solved in MeOH (1 mL), and 7 M NH_3/MeOH was added dropwise. After complete conversion (monitored by LC-MS), the reaction was concentrated and purified by reverse-phase C18 column chromatography (H_2O:MeOH/95:5 to 5:95) and lyophilized to afford **P10C**. White solid, yield 80%, 32 mg.

^{1}H NMR (800 MHz, DMSO-*d*$_6$) δ 11.80 (s, 1H), 9.86 (s, 1H), 9.51 (s, 1H), 8.43 (d, *J* = 7.4 Hz, 1H), 8.18 – 8.11 (m, 1H), 7.95 – 7.89 (m, 1H), 7.63 (d, *J* = 7.8 Hz, 1H), 7.46 (t, *J* = 7.8 Hz, 1H), 7.00 – 6.98 (m, 1H), 6.94 (t, *J* = 7.6 Hz, 1H), 5.03 – 4.99 (m, 1H), 4.62 (t, *J* = 9.3 Hz, 1H), 4.53 (t, *J* = 8.0 Hz, 1H), 4.46 – 4.41 (m, 1H), 4.20 – 4.14 (m, 1H), 3.97 (q, *J* = 7.1 Hz, 1H), 3.93 – 3.88 (m, 1H), 3.52 – 3.47 (m, 1H), 3.46 – 3.41 (m, 2H), 3.37 – 3.33 (m, 1H), 3.29 – 3.24 (m, 1H), 2.34 – 2.26 (m, 2H), 1.81 – 1.63 (m, 5H), 1.63 – 1.51 (m, 2H), 1.51 – 1.39 (m, 5H), 1.38 (s, 9H), 1.28 – 1.17 (m, 22H), 0.85 (t, *J* = 7.0 Hz, 3H).

^{13}C NMR (201 MHz, DMSO-*d*$_6$) δ 173.2, 172.2, 170.2, 170.0, 169.2, 166.4, 159.6, 155.6, 134.5, 128.5, 119.5, 117.0, 110.3, 78.9, 69.8, 67.7, 54.7, 53.7, 52.8, 51.5, 47.4, 32.1, 31.9, 31.8, 30.4, 29.5 (×3), 29.4 (×2), 29.3, 29.2, 28.6, 27.4, 26.5, 26.1, 24.7, 22.9, 22.6, 14.4.

HRMS (ESI-MS) *m/z*: [M+H]$^+$; Calcd for $C_{44}H_{72}N_7O_{11}$ 874.5284; Found 874.5274.

2.1.5 Synthesis of enantiomer A2 and (*R*)-A2

The synthesis of **(*R*)-A2** was similar to that of **A2** that was described in **2.1.1**, but with D-serine benzyl ester hydrochloride as substrate. The enantiomeric excesses of products were determined by chiral phase HPLC analysis (see Figure S1).

The ^{1}H, ^{13}C NMR, and MS of **(*R*)-A2** was identical to **A2**.

2.1.6 Synthesis of enantiomer BocA7 and (*R*)-BocA7

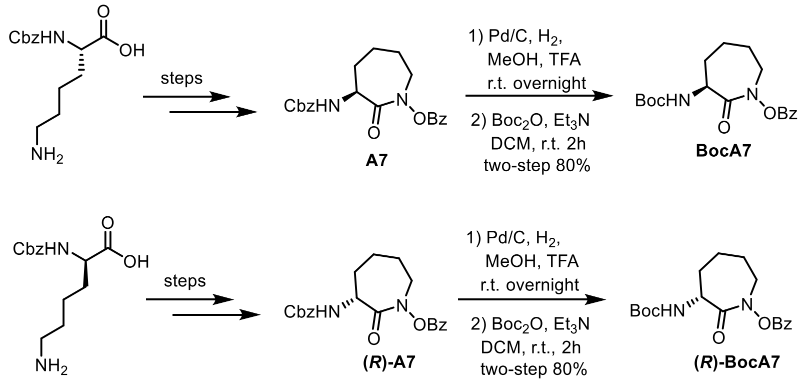

Scheme S6. Synthesis of **(*R*)-BocA7**

As described in **2.1.1**, L -lysine was converted to **A7** through multi-steps. To a solution of the **A7** (383 mg, 1.0 mmol) in MeOH (10 mL) was added 10% Pd/C (38 mg, 10% w/w) and TFA (100 μL). The mixture was stirred under H_2 atmosphere at r.t. overnight. After the reaction completed, the solution was filtrated and evaporated in vacuo to get crude amine (colorless oil). The intermediate oil was used directly without further purification.

To a solution of the above intermediate oil and Et_3N (1.5 mmol, 1.5 equiv.) in DCM (10 mL) was add Boc_2O (1.2 mmol, 1.2 equiv.) dropwise. After stirring at r.t. for 2 h, the reaction was concentrated and purified by silica gel column chromatography (PE:EA/100:0 to PE:EA/75:25) to afford **BocA7**. R_f = 0.6 (PE:EA/3:1), colorless oil, two-step yield 80%, 280 mg.

^{1}H NMR (400 MHz, $CDCl_3$) δ 8.13 – 8.06 (m, 2H), 7.67 – 7.61 (m, 1H), 7.52 – 7.46 (m, 2H), 5.82 (d, *J* = 6.2 Hz, 1H), 4.47 – 4.39 (m, 1H), 4.09 – 4.02 (m, 1H), 3.74 – 3.66 (m, 1H), 2.16 – 2.06 (m, 2H), 1.93 – 1.81 (m, 3H), 1.76 – 1.69 (m, 1H), 1.45 (s, 9H).

^{13}C NMR (101 MHz, $CDCl_3$) δ 169.2, 163.9, 155.3, 134.3, 130.3, 128.8, 126.9, 79.8, 53.6, 53.3, 32.3, 28.5, 28.1, 26.4.

HRMS (ESI-MS) *m/z*: $[M+H]^+$; Calcd for $C_{18}H_{25}N_2O_5$ 349.1758; Found 349.1756.

The synthesis of **(*R*)-BocA7** was similar to that of **BocA7** that was described above, but with D-lysine as substrate. The enantiomeric excesses of products were determined by chiral phase HPLC analysis (see Figure S2).

The ^{1}H, ^{13}C NMR, and MS of **(*R*)-BocA7** was identical to **BocA7**.

2.2 Synthesis of mycobactin analogues (*S*)-P10C-16 and (*R*)-P10C-16

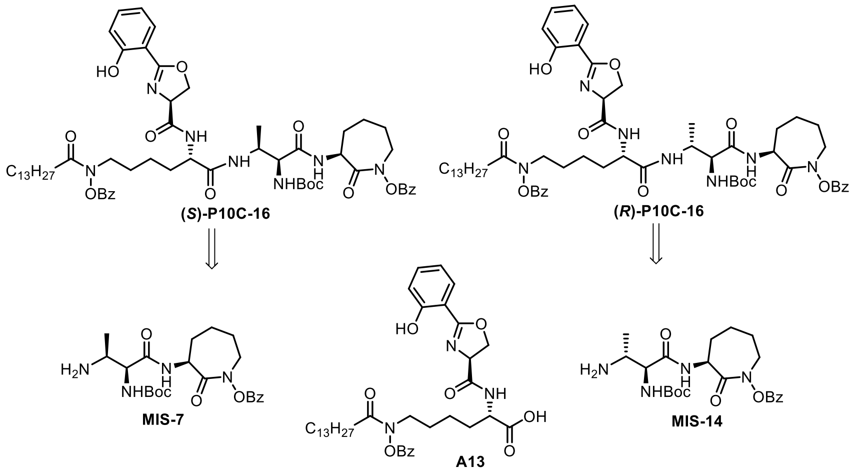

Scheme S7. Retrosynthetic strategy of **(*S*)-P10C-16** and **(*R*)-P10C-16**

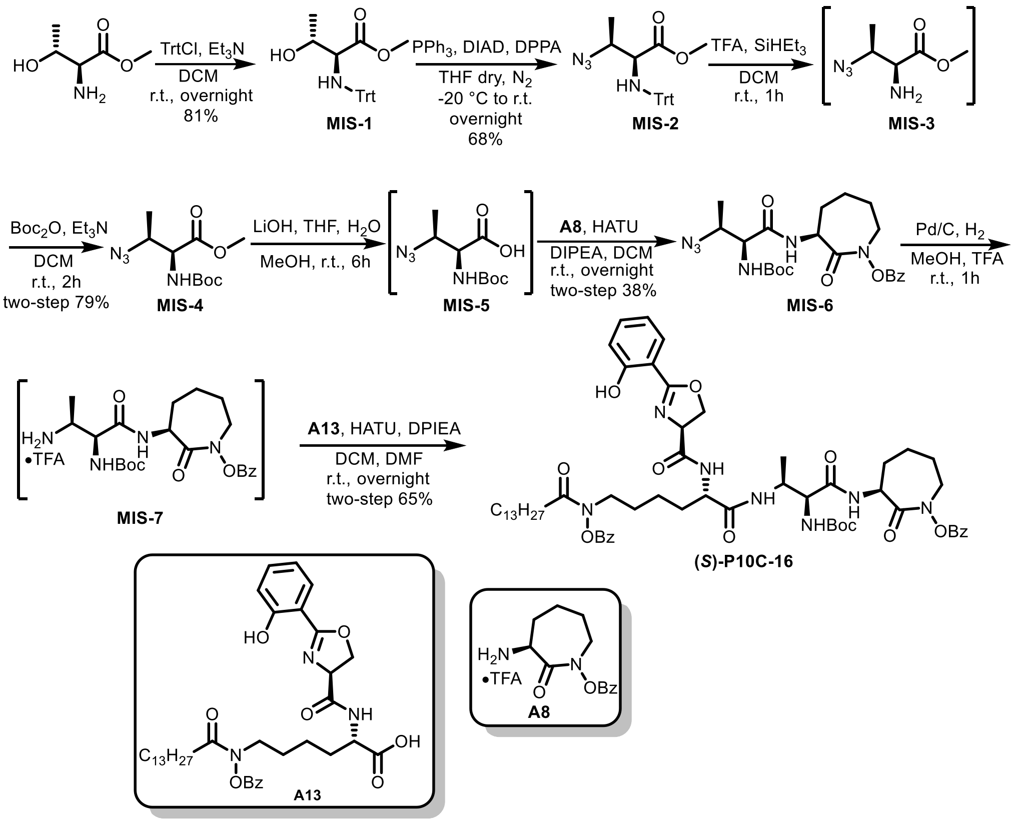

Scheme S8. Synthesis of **(*S*)-P10C-16**

The synthetic route to intermediate **MIS-2** was similar to literature.[2]

Methyl trityl-L-threoninate (MIS-1)

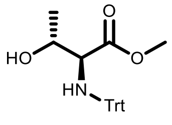

MIS-1

The commercially available methyl L-threoninate (1.3 g, 10.0 mmol) and Et_3N (3 g, 30.0 mmol, 3.0 equiv.) was dissolved in DCM (15 mL), then trityl chloride (5.5 g, 20.0 mmol, 2.0 equiv.) was added in three portions. After stirring at r.t. overnight, the reaction was concentrated and purified by silica gel column chromatography (PE:EA/100:0 to PE:EA/75:25) to afford **MIS-1**. R_f = 0.5 (PE:EA/3:1), colorless oil, yield 81%, 3 g.

^{1}H NMR (400 MHz, $CDCl_3$) δ 7.56 (d, *J* = 7.6 Hz, 6H), 7.41 – 7.24 (m, 10H), 3.88 (p, *J* = 6.4 Hz, 1H), 3.47 (d, *J* = 7.4 Hz, 1H), 3.24 (s, 3H), 1.29 (d, *J* = 6.2 Hz, 3H).

^{13}C NMR (101 MHz, $CDCl_3$) δ 173.7, 145.4, 128.9, 127.9, 126.7, 70.8, 69.8, 62.5, 51.7, 18.9.

The data matched with the literature.[2]

Methyl (2*S*,3*S*)-3-azido-2-(tritylamino)butanoate (MIS-2)

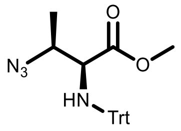

MIS-2

To a flask with **MIS-1** (1.5 g, 4 mmol) and triphenylphosphine (1.6 g, 6.08 mmol, 1.52 equiv.) in dry THF (20 mL), a solution of diisopropyl azodicarboxylate (1.2 g, 6.0 mmol, 1.5 equiv.) in dry THF (5 mL) was added dropwise at -20 °C. As followed a solution of diphenylphosphoryl azide (1.78 g, 6.4 mmol, 1.6 equiv.) in dry THF (4 mL) was added dropwise over 5 min. Then the reaction mixture was stirred at r.t. overnight. The solvent were removed in vacuo, and the residue was purified with silica gel column chromatography (PE:EA/100:0 to PE:EA/80:20) to afford **MIS-2**. R_f = 0.6 (PE:EA/10:1), colorless oil, yield 68%, 1.08 g.

^{1}H NMR (400 MHz, $CDCl_3$) δ 7.54 – 7.50 (m, 6H), 7.30 – 7.25 (m, 6H), 7.23 – 7.17 (m, 3H), 3.88 – 3.80 (m, 1H), 3.50 – 3.42 (m, 1H), 3.21 (s, 3H), 2.97 (d, J = 8.8 Hz, 1H), 1.23 (d, J = 6.9 Hz, 3H).

^{13}C NMR (101 MHz, $CDCl_3$) δ 171.9, 145.7, 128.9, 128.1, 126.7, 71.3, 61.5, 60.7, 51.9, 16.4.

The data matched with the literature.[2]

Methyl (2*S*,3*S*)-3-azido-2-((*tert*-butoxycarbonyl)amino)butanoate (MIS-4)

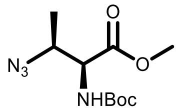

MIS-4

To a solution of the intermediate **MIS-2** (800 mg, 2.0 mmol) and $SiHEt_3$ (348 mg, 3.0 mmol, 1.5 equiv.) in DCM (10 mL) was added TFA (10 mL) dropwise at ice bath. Then the solution was stirred at r.t. for 1 h until the **MIS-2** disappeared in TLC detection and then evaporated in vacuo to afford crude amine (colorless oil), that was used directly in the next step.

To a solution of the above intermediate oil and Et_3N (400 mg, 4.0 mmol, 2.0 equiv.) in DCM (10 mL) was add Boc_2O (523 mg, 2.4 mmol, 1.2 equiv.) dropwise at r.t. After stirring at r.t. for 2 h, the mixture was concentrated and purified by silica gel column chromatography (PE:EA/100:0 to PE:EA/75:25) to afford **MIS-4**. R_f = 0.7 (PE:EA/3:1), colorless oil, two-step yield 79%, 408 mg.

^{1}H NMR (400 MHz, $CDCl_3$) δ 5.31 (d, J = 8.6 Hz, 1H), 4.47 – 4.33 (m, 1H), 3.87 – 3.79 (m, 1H), 3.78 (s, 3H), 1.45 (s, 9H), 1.34 (d, J = 6.9 Hz, 3H).

^{13}C NMR (101 MHz, $CDCl_3$) δ 170.1, 155.1, 80.5, 58.8, 57.4, 52.6, 28.3, 15.5.

HRMS is not detected because of instability of N_3 group.

(*S*)-3-((2*S*,3*S*)-3-azido-2-((*tert*-butoxycarbonyl)amino)butanamido)-2-oxoazepan-1-yl benzoate (MIS-6)

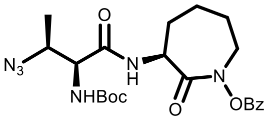

MIS-6

To a stirred solution of **MIS-4** (361 mg, 1.5 mmol) in THF (5 mL)/MeOH (5 mL)/was added LiOH (108 mg, 4.5 mmol, 3 equiv. in 5 mL H_2O) dropwise at r.t. After stirring for 6 h, the reaction was acidified by 1 M HCl (aq.) and then extracted by EtOAc three times (10 mL × 3). Combined organic phase was washed by brine (15 mL) and dried over anhydrous Na_2SO_4 to afford intermediate **MIS-5** as colorless oil, which was used directly in the next step.

The intermediate **MIS-5** and **A8** (519 mg, 1.5 mmol, 1.0 equiv.) were dissolved in DCM (10 mL), followed by the addition of HATU (627 mg, 1.65 mmol, 1.1 equiv.) and DIPEA (582 mg, 4.5 mmol, 3.0 equiv.). After stirring at r.t. overnight, the reaction was diluted with EtOAc (30 mL) and washed sequentially with 1 M HCl (aq., 15 mL), H_2O (15 mL) and brine (15 mL). The organic layer was dried over anhydrous Na_2SO_4, filtered, and concentrated in vacuo. The mixture was purified by silica gel column chromatography (PE:EA/100:0 to PE:EA/50:50) to afford intermediate **MIS-6**. R_f = 0.6 (PE:EA/1:1), white solid, 270 mg, two-step yield 38%.

^{1}H NMR (400 MHz, $CDCl_3$) δ 8.13 – 8.06 (m, 2H), 7.69 – 7.60 (m, 1H), 7.49 (t, *J* = 7.7 Hz, 2H), 7.31 (d, *J* = 6.2 Hz, 1H), 5.28 (d, *J* = 8.5 Hz, 1H), 4.64 (dd, *J* = 11.0, 6.2 Hz, 1H), 4.23 (s, 1H), 4.08 (dd, *J* = 16.3, 10.5 Hz, 1H), 3.82 (p, *J* = 6.6 Hz, 1H), 3.73 (dd, *J* = 16.7, 4.5 Hz, 1H), 2.11 (d, *J* = 11.1 Hz, 2H), 1.90 (p, *J* = 12.6, 12.0 Hz, 3H), 1.79 – 1.71 (m, 1H), 1.44 (s, 9H), 1.33 (d, *J* = 6.7 Hz, 3H).

^{13}C NMR (126 MHz, $CDCl_3$) δ 168.2 (×2), 163.7, 155.2, 134.3, 130.2, 128.8, 126.6, 80.6, 58.6, 57.9, 53.5, 52.4, 31.3, 28.3, 27.8, 26.1, 15.3.

HRMS (ESI-MS) *m/z*: $[M+H]^+$; Calcd for $C_{22}H_{31}N_6O_6$ 475.2300; Found 475.2295.

(*S*)-3-((2*S*,3*S*)-3-((*S*)-6-(*N*-(benzoyloxy)tetradecanamido)-2-((*S*)-2-(2-hydroxyphenyl)-4,5-dihydrooxazole-4-carboxamido)hexanamido)-2-((*tert*-butoxycarbonyl)amino)butanamido)-2-oxoazepan-1-yl benzoate [(*S*)-P10C-16]

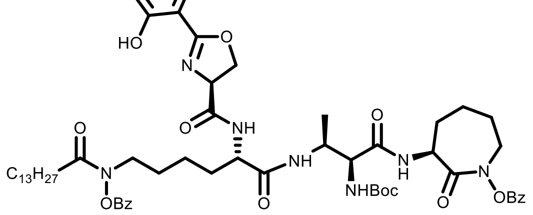

(*S*)-P10C-16

To a stirred solution of the **MIS-6** (192 mg, 0.5 mmol) in MeOH (5 mL) was added 10% Pd/C (19 mg, 10% w/w, 10%) and TFA (50 μL). The mixture was stirred under H_2 atmosphere at r.t. 1 h. After the reaction completed, the solution was filtrated and evaporated in vacuo to get crude amine (colorless oil), that was used directly in the next step.

The above intermediate oil and **A13** (333 mg, 0.5 mmol, 1.0 equiv.) were dissolved in

DCM (2 mL) and DMF (2 mL), followed by the addition of HATU (209 mg, 0.55 mmol, 1.1 equiv.) and DIPEA (194 mg, 1.5 mmol, 3.0 equiv.). After stirring at r.t. overnight, the reaction was diluted with EtOAc (15 mL) and washed sequentially with 1 M HCl (aq., 8 mL), H_2O (8 mL) and brine (8 mL). The organic layer was dried over anhydrous Na_2SO_4, filtered, and concentrated in vacuo. The mixture was purified by reverse-phase C18 column chromatography (H_2O:MeOH/95:5 to 5:95) to afford **(*S*)-P10C-16**. White solid, two-step yield 65%, 356 mg.

^{1}H NMR (800 MHz, DMSO-d_6) δ 11.84 (s, 1H), 8.46 (s, 1H), 8.05 – 7.98 (m, 4H), 7.95 (s, 1H), 7.85 (s, 1H), 7.78 – 7.72 (m, 2H), 7.63 – 7.53 (m, 5H), 7.47 – 7.42 (m, 1H), 7.00 (s, 1H), 6.96 – 6.90 (m, 2H), 5.04 – 4.99 (m, 1H), 4.67 – 4.59 (m, 2H), 4.56 (s, 1H), 4.28 – 4.15 (m, 4H), 3.80 (d, *J* = 16.1 Hz, 1H), 3.69 (s, 2H), 2.22 (s, 2H), 1.93 (s, 1H), 1.84 (d, *J* = 13.5 Hz, 1H), 1.79 – 1.66 (m, 4H), 1.64 – 1.51 (m, 4H), 1.49 – 1.44 (m, 2H), 1.34 (s, 9H), 1.31 – 1.10 (m, 22H), 0.99 (s, 3H), 0.88 – 0.83 (m, 3H).

^{13}C NMR (201 MHz, DMSO-d_6) δ 171.0, 170.0, 169.4, 166.2, 163.8, 159.5, 155.9, 134.9, 134.4, 130.1, 129.6, 128.4, 127.1, 119.4, 117.0, 110.3, 78.9, 69.7, 67.7, 57.8, 55.4, 52.8, 51.7, 46.3, 32.1, 31.8, 31.1, 29.5 (×2), 29.4, 29.3, 29.2, 28.9, 28.6, 28.4, 27.6, 26.3, 24.4, 23.1, 22.6, 16.0, 14.4.

HRMS (ESI-MS) *m/z*: [M+H]$^+$; Calcd for $C_{59}H_{82}N_7O_{13}$ 1096.5965; Found 1096.5953.

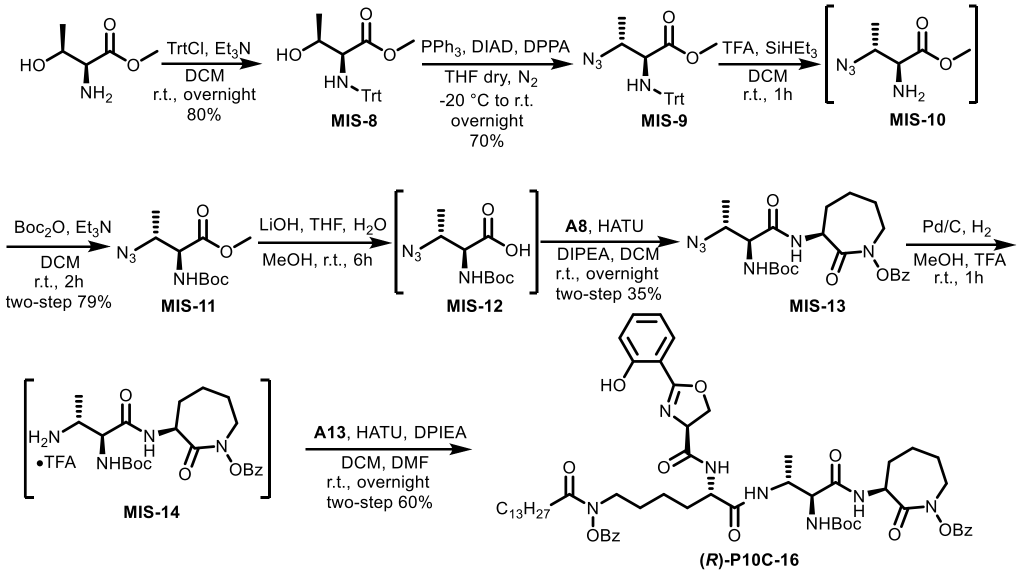

Scheme S9. Synthesis of **(*R*)-P10C-16**

The synthetic route to intermediate **MIS-9** was similar to literature.[2]

Methyl trityl-L-allothreoninate (MIS-8)

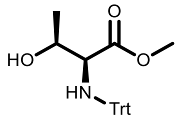

MIS-8

Following the same procedure in preparation of **MIS-1**, the **MIS-8** was afforded as white solid. R_f = 0.5 (PE:EA/3:1), yield 80%.

^{1}H NMR (400 MHz, $CDCl_3$) δ 7.54 – 7.47 (m, 6H), 7.30 – 7.23 (m, 7H), 7.22 – 7.16 (m, 3H), 4.02 – 3.95 (m, 1H), 3.40 (d, *J* = 3.9 Hz, 1H), 3.23 (s, 3H), 3.01 (s, 1H), 2.39 (s, 1H), 1.11 (d, *J* = 6.6 Hz, 3H).
^{13}C NMR (101 MHz, $CDCl_3$) δ 173.1, 145.7, 128.8, 127.9, 126.6, 71.0, 69.9, 61.1, 51.5, 19.6.
The data matched with the literature.[2]

Methyl (2*S*,3*R*)-3-azido-2-(tritylamino)butanoate (MIS-9)

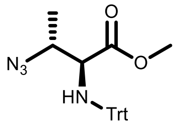

MIS-9

Following the same procedure in preparation of **MIS-2**, the **MIS-9** was afforded as colorless oil. R_f = 0.7 (PE:EA/10:1), yield 70%.
^{1}H NMR (400 MHz, $CDCl_3$) δ 7.55 – 7.50 (m, 5H), 7.35 – 7.17 (m, 10H), 3.78 (p, *J* = 6.5 Hz, 1H), 3.51 (dd, *J* = 10.8, 5.8 Hz, 1H), 3.21 (s, 3H), 2.81 (d, *J* = 10.8 Hz, 1H), 1.31 (d, *J* = 6.6 Hz, 3H).
^{13}C NMR (101 MHz, $CDCl_3$) δ 172.7, 145.5, 128.8, 127.9, 126.6, 71.1, 60.6, 60.2, 52.0, 15.5.
The data matched with the literature.[2]

Methyl (2*S*,3*R*)-3-azido-2-((*tert*-butoxycarbonyl)amino)butanoate (MIS-11)

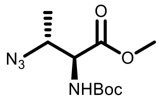

MIS-11

Following the same procedure in preparation of **MIS-4**, the **MIS-11** was afforded as white solid. R_f = 0.7 (PE:EA/3:1), two-step yield 79%.
^{1}H NMR (400 MHz, $CDCl_3$) δ 5.17 (d, *J* = 9.5 Hz, 1H), 4.39 (dd, *J* = 9.6, 2.7 Hz, 1H), 4.22 – 4.12 (m, 1H), 3.81 (s, 3H), 1.48 (s, 9H), 1.37 (d, *J* = 6.7 Hz, 3H).
^{13}C NMR (101 MHz, $CDCl_3$) δ 170.6, 155.8, 80.4, 58.7, 57.2, 52.7, 28.2, 16.0.
HRMS is not detected because of instability of N_3 group.

(*S*)-3-((2*S*,3*R*)-3-azido-2-((*tert*-butoxycarbonyl)amino)butanamido)-2-oxoazepan-1-yl benzoate (MIS-13)

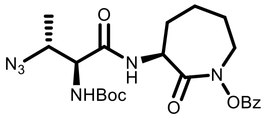

MIS-13

Following the same procedure in preparation of **MIS-6**, the **MIS-13** was afforded as white solid. R_f = 0.6 (PE:EA/1:1), two-step yield 35%.
^{1}H NMR (400 MHz, $CDCl_3$) δ 8.12 – 8.07 (m, 2H), 7.67 – 7.62 (m, 1H), 7.59 (d, *J* = 5.9 Hz, 1H), 7.52 – 7.47 (m, 2H), 5.18 (d, *J* = 8.9 Hz, 1H), 4.64 (dd, *J* = 10.8, 6.0 Hz,

1H), 4.35 (d, J = 6.3 Hz, 1H), 4.23 (d, J = 8.9 Hz, 1H), 4.09 (dd, J = 16.3, 10.6 Hz, 1H), 3.72 (dd, J = 16.5, 4.6 Hz, 1H), 2.16 – 2.06 (m, 2H), 1.95 – 1.82 (m, 3H), 1.79 – 1.72 (m, 1H), 1.44 (s, 9H), 1.28 (d, J = 6.7 Hz, 3H).
^{13}C NMR (126 MHz, $CDCl_3$) δ 168.6, 168.3, 163.7, 155.7, 134.3, 130.2, 128.7, 126.6, 80.7, 58.5, 57.9, 53.5, 52.5, 31.3, 28.2, 27.8, 26.2, 16.0.
HRMS (ESI-MS) *m/z*: $[M+H]^+$; Calcd for $C_{22}H_{31}N_6O_6$ 475.2300; Found 475.2295.

(*S*)-3-((2*S*,3*R*)-3-((*S*)-6-(*N*-(benzoyloxy)tetradecanamido)-2-((*S*)-2-(2-hydroxyphenyl)-4,5-dihydrooxazole-4-carboxamido)hexanamido)-2-((*tert*-butoxycarbonyl)-amino)butanamido)-2-oxoazepan-1-yl benzoate [(*R*)-P10C-16]

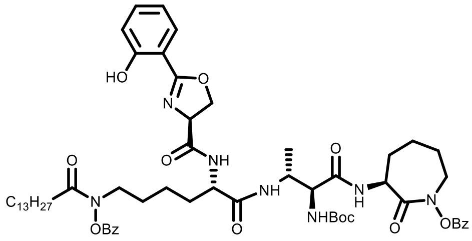

(*R*)-P10C-16

Following the same procedure in preparation of **(*S*)-P10C-16**, the **(*R*)-P10C-16** was afforded as white solid. Two-step yield 60%.
^{1}H NMR (600 MHz, DMSO-d_6) δ 11.79 (s, 1H), 8.44 (d, J = 8.2 Hz, 1H), 8.06 – 7.95 (m, 5H), 7.88 (d, J = 9.0 Hz, 1H), 7.76 – 7.70 (m, 2H), 7.62 – 7.53 (m, 5H), 7.46 – 7.42 (m, 1H), 6.97 – 6.87 (m, 3H), 5.02 – 4.98 (m, 1H), 4.65 – 4.57 (m, 2H), 4.51 (t, J = 7.9 Hz, 1H), 4.23 – 4.13 (m, 3H), 4.05 – 3.99 (m, 1H), 3.82 – 3.76 (m, 1H), 3.71 – 3.60 (m, 2H), 2.20 (s, 2H), 1.90 – 1.84 (m, 1H), 1.81 (d, J = 13.5 Hz, 1H), 1.77 – 1.64 (m, 4H), 1.61 – 1.49 (m, 4H), 1.47 – 1.42 (m, 2H), 1.37 (s, 9H), 1.28 – 1.13 (m, 22H), 1.01 (d, J = 6.8 Hz, 3H), 0.84 (t, J = 6.9 Hz, 3H).
^{13}C NMR (151 MHz, DMSO-d_6) δ 170.4, 169.3, 168.8, 165.7, 163.1, 159.0, 155.1, 134.3, 133.9, 129.5, 129.0, 127.9, 126.5, 118.9, 116.4, 109.7, 78.5, 69.1, 67.1, 58.2, 52.6, 52.2, 51.3, 45.7, 31.3, 31.2, 30.1, 28.9 (×2), 28.8, 28.7, 28.6, 28.3, 28.0, 27.8, 26.9, 25.7, 23.8, 22.3, 22.0, 16.9, 13.8.
HRMS (ESI-MS) *m/z*: $[M+H]^+$; Calcd for $C_{59}H_{82}N_7O_{13}$ 1096.5965; Found 1096.5961.

2.3 Synthesis of (*S*)-P10C and (*R*)-P10C

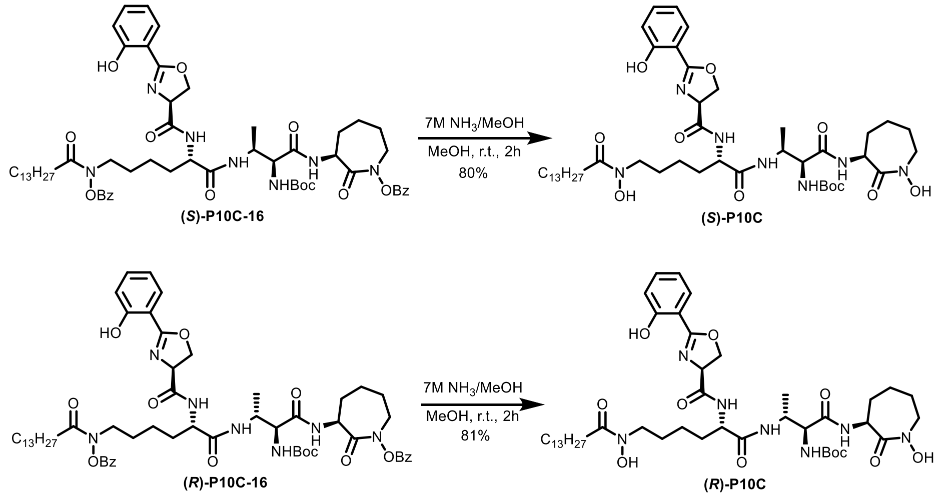

Scheme S10. Synthesis of **(*S*)-P10C** and **(*R*)-P10C**

The **(*S*)-P10C-16** (50 mg, 0.045 mmol) was solved in MeOH (1 mL), and 7 M NH_3/MeOH was added dropwise. After complete conversion (monitored by LC-MS), the reaction was concentrated and purified by reverse C18 column chromatography (H_2O:MeOH/95:5 to 5:95) and lyophilized to afford **(*S*)-P10C**. White solid, yield 80%, 32 mg.

^{1}H NMR (600 MHz, DMSO-d_6) δ 11.82 (s, 1H), 9.82 (s, 1H), 9.49 (s, 1H), 8.40 (d, *J* = 7.9 Hz, 1H), 7.87 (d, *J* = 6.6 Hz, 1H), 7.81 (d, *J* = 7.4 Hz, 1H), 7.65 – 7.61 (m, 1H), 7.48 – 7.43 (m, 1H), 7.04 (d, *J* = 8.4 Hz, 1H), 6.99 (d, *J* = 8.3 Hz, 1H), 6.94 (t, *J* = 7.5 Hz, 1H), 5.04 – 4.99 (m, 1H), 4.65 – 4.59 (m, 1H), 4.53 (t, *J* = 7.9 Hz, 1H), 4.47 – 4.41 (m, 1H), 4.23 – 4.13 (m, 3H), 3.93 – 3.86 (m, 1H), 3.52 – 3.46 (m, 1H), 3.45 – 3.40 (m, 2H), 2.30 (t, *J* = 7.6 Hz, 2H), 1.86 – 1.80 (m, 1H), 1.76 (d, *J* = 13.3 Hz, 1H), 1.73 – 1.57 (m, 4H), 1.55 – 1.48 (m, 2H), 1.47 – 1.41 (m, 4H), 1.39 (s, 9H), 1.25 – 1.20 (m, 22H), 0.99 (d, *J* = 6.2 Hz, 3H), 0.85 (t, *J* = 6.9 Hz, 3H).

^{13}C NMR (151 MHz, DMSO-d_6) δ 172.6, 170.5, 169.5, 168.7, 168.1, 165.8, 159.0, 155.4, 133.9, 127.9, 118.9, 116.5, 109.8, 78.4, 69.3, 67.1, 57.2, 52.8, 52.3, 50.7, 47.0, 45.7, 31.6, 31.2, 30.7, 28.9 (×3), 28.8 (×2), 28.7, 28.6, 28.1, 26.9, 25.9, 25.5, 24.1, 22.5, 22.0, 15.5, 13.9.

HRMS (ESI-MS) *m/z*: $[M+H]^+$; Calcd for $C_{45}H_{74}N_7O_{11}$ 888.5441; Found 888.5440.

The synthesis of **(*R*)-P10C** was identical to that above. White solid, yield 81%, 33 mg.

^{1}H NMR (600 MHz, DMSO-d_6) δ 11.79 (s, 1H), 9.87 (s, 1H), 9.50 (s, 1H), 8.43 (d, *J* = 7.3 Hz, 1H), 7.99 (d, *J* = 9.2 Hz, 1H), 7.89 (d, *J* = 6.9 Hz, 1H), 7.65 – 7.61 (m, 1H), 7.48 – 7.44 (m, 1H), 6.99 (d, *J* = 8.3 Hz, 1H), 6.96 – 6.91 (m, 2H), 5.04 – 4.98 (m, 1H), 4.65 – 4.60 (m, 1H), 4.49 (t, *J* = 7.8 Hz, 1H), 4.46 – 4.41 (m, 1H), 4.24 – 4.18 (m, 1H), 4.14 – 4.08 (m, 1H), 4.01 – 3.96 (m, 1H), 3.92 – 3.86 (m, 1H), 3.50 – 3.40 (m, 3H), 2.30 (t, *J* = 7.8 Hz, 2H), 1.77 – 1.62 (m, 5H), 1.62 – 1.54 (m, 2H), 1.54 – 1.41 (m, 5H), 1.39 (s, 9H), 1.26 – 1.20 (m, 22H), 1.01 (d, *J* = 6.8 Hz, 3H), 0.85 (t, *J* = 6.9 Hz, 4H).

^{13}C NMR (151 MHz, DMSO-d_6) δ 172.6, 170.2, 169.5, 168.7, 168.5, 165.8, 159.0, 155.1, 133.9, 127.9, 119.0, 116.4, 109.7, 78.5, 69.3, 67.0, 57.9, 53.1, 52.4, 50.9, 46.7,

45.4, 31.6, 31.2, 29.9, 28.9 (×3), 28.8 (×2), 28.7, 28.6, 28.0, 26.9, 25.9, 25.5, 24.1, 22.3, 22.0, 16.6, 13.8.
HRMS (ESI-MS) *m/z*: $[M+H]^+$; Calcd for $C_{45}H_{74}N_7O_{11}$ 888.5441; Found 888.5439.

2.4 Synthesis of fluorophores

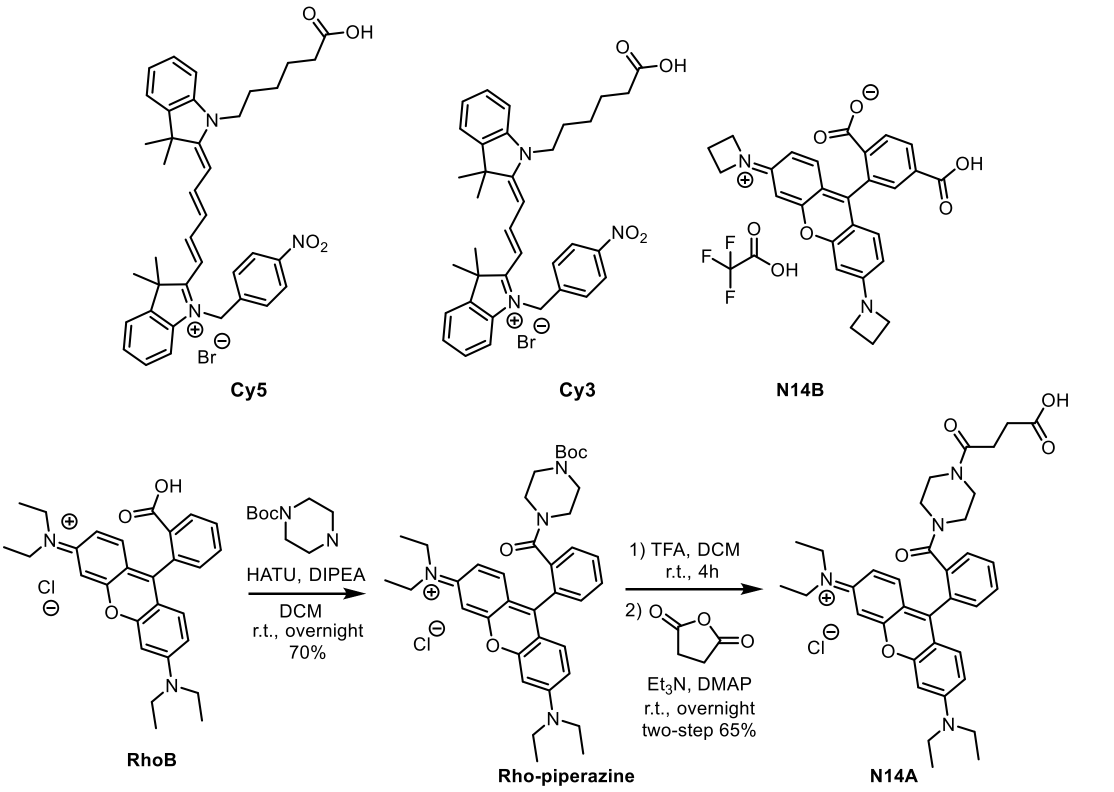

Scheme S11. Structures of fluorophores and synthesis of **N14A**

2-((1*E*,3*E*)-5-((*E*)-1-(5-carboxypentyl)-3,3-dimethylindolin-2-ylidene)penta-1,3-dien-1-yl)-3,3-dimethyl-1-(4-nitrobenzyl)-3*H*-indol-1-ium (Cy5) and 2-((*E*)-3-((*E*)-1-(5-carboxypentyl)-3,3-dimethylindolin-2-ylidene)prop-1-en-1-yl)-3,3-dimethyl-1-(4-nitrobenzyl)-3*H*-indol-1-ium (Cy3)

The synthesis of compounds **Cy3** and **Cy5** followed the literature method reported by Hong et al., 2024.[3]

The **N14A** was reported by Francis and Nguyen et al,[4] and it was synthesized by different methods in this article.

The synthesis of **N14B** followed the literature methods reported by Grimm et al.[5]

***N*-(9-(2-(4-(3-carboxypropyl)piperazine-1-carbonyl)phenyl)-6-(diethylamino)-3*H*-xanthen-3-ylidene)-*N*-ethylethanaminium (N14A)**

Commercially available compound **RhoB** (1.9 g, 4.0 mmol) and *N*-Boc-piperazine (815 mg, 4.4 mmol, 1.1 equiv.) were dissolved in DCM (15 mL), followed by the addition of HATU (1.6 g, 4.4 mmol, 1.1equiv.) and DIPEA (1.5g, 12.0 mmol, 3.0 equiv.). After stirring at r.t. overnight, the reaction was diluted with DCM (30 mL) and washed sequentially with 1 M HCl (aq., 25 mL), H_2O (25 mL) and brine (25 mL). The organic

layer was dried over anhydrous Na_2SO_4, filtered, and concentrated in vacuo. The reaction was purified by reverse-phase C18 column chromatography (H_2O:MeCN/95:5 to 5:95) to afford intermediate **Rho-piperazine**. Red solid, yield 70%, 1.8 g.
^{1}H NMR (400 MHz, $CDCl_3$) δ 7.72 – 7.63 (m, 2H), 7.55 – 7.48 (m, 1H), 7.37 – 7.29 (m, 1H), 7.21 (d, *J* = 9.4 Hz, 2H), 6.91 (d, *J* = 9.4 Hz, 2H), 6.76 (s, 2H), 3.67 – 3.54 (m, 8H), 3.41 – 3.23 (m, 8H), 1.41 (s, 9H), 1.30 (t, *J* = 7.1 Hz, 12H).
^{13}C NMR (101 MHz, $CDCl_3$) δ 167.8, 157.8, 155.7, 154.6, 135.2, 132.1, 130.8, 130.4, 130.3, 130.2, 127.6, 114.2, 113.8, 96.4, 80.6, 47.5, 46.2, 41.9, 28.5, 28.4, 12.6.
HRMS (ESI-MS) *m/z*: $[M]^+$; Calcd for $C_{37}H_{47}N_4O_4$ 611.3592; Found 611.3591.

To a stirred solution of intermediate **Rho-piperazine** (1.29 g, 2.0 mmol) in DCM (10 mL) added TFA (10 mL) dropwise at ice bath. After stirring at r.t. for 4 h, the reaction was concentrated to afford intermediate amine as red oil, that was used directly in the next step.
The above oil intermediate, Et_3N (404 mg, 4.0 mmol, 2.0 equiv.), DMAP (366 mg, 3.0 mmol, 1.5 equiv.) and succinic anhydride (300 mg, 3.0 mmol, 1.5 equiv.) were dissolved in DCM (5 mL) at ice bath. After stirring at r.t. overnight, the reaction was concentrated and purified by reverse-phase C18 column chromatography (H_2O:MeCN/95:5 to 5:95) to afford intermediate **N14A**. Red solid, yield 65%, 750 mg.
^{1}H NMR (400 MHz, MeOD) δ 7.82 – 7.74 (m, 2H), 7.73 – 7.68 (m, 1H), 7.55 – 7.49 (m, 1H), 7.28 (d, *J* = 9.5 Hz, 2H), 7.07 (dd, *J* = 9.5, 2.5 Hz, 2H), 6.96 (d, *J* = 2.5 Hz, 2H), 3.69 (q, *J* = 7.1 Hz, 8H), 3.54 – 3.35 (m, 8H), 2.63 – 2.50 (m, 4H), 1.31 (t, *J* = 7.1 Hz, 12H).
^{13}C NMR (101 MHz, MeOD) δ 176.7, 172.8, 169.6, 159.3, 157.2, 157.0, 136.6, 133.2, 132.3, 131.8, 131.3, 131.3, 129.9, 128.9, 115.4, 114.9, 97.4, 79.6, 79.3, 79.0, 46.9, 42.9, 30.1, 28.8, 12.8.
HRMS (ESI-MS) *m/z*: $[M]^+$: Calcd for $C_{36}H_{43}N_4O_5$ 611.3228; Found 611.3226.

2.5 Synthesis of MbTFCps (N14C~N14L)
To a stirred solution of intermediate **P10C-16** (50 mg, 0.046 mmol), **(*S*)-P10C-16** or **(*R*)-P10C-16** (50 mg, 0.046 mmol) in DCM (1 mL) was added TFA (1 mL) dropwise at ice bath. After stirring at r.t. for 30 min, the reaction was concentrated to afford intermediate amine as colorless oil, that was used directly in the next step.
The above oil intermediate, Et_3N (19 mg, 0.184 mmol, 4.0 equiv.), HATU (18 mg, 0.046 mmol, 1.0 equiv.) and fluorophores (i.e. **Cy3** or **Cy5** or **N14A** or **N14B** or analogs, 0.046 mmol, 1.0 equiv.) were dissolved in DCM (1 mL). After stirring at r.t. for 30 min, the reaction was diluted with DCM (10 mL) and washed sequentially with 1 M HCl (aq., 5 mL), H_2O (5 mL) and brine (5 mL), The organic layer was dried over anhydrous Na_2SO_4, filtered, and concentrated in vacuo to afford blue or red solid as intermediate, which was used directly in the next step (except for **N14G-Bz**, which was purified by reverse-phase C18 column chromatography).
The above intermediate was resolved in MeOH (1 mL), and 7 M NH_3/MeOH was added dropwise. After complete conversion (monitored by LC-MS), the reaction was concentrated and purified by reverse C18 column chromatography (H_2O:MeOH/95:5

to 5:95) and lyophilized to afford a series of conjugates (**N14C~N14L**). Blue solid (**N14C**) and red solid (other conjugates), yields 23-36%, 12–23 mg.

N14C, blue powder, yield 25%, 15 mg.
^{1}H NMR (600 MHz, DMSO-d_6) δ 11.80 (s, 1H), 9.86 (s, 1H), 9.55 (s, 1H), 8.43 (d, J = 7.4 Hz, 1H), 8.39 – 8.28 (m, 2H), 8.23 (d, J = 8.3 Hz, 2H), 8.12 (t, J = 5.9 Hz, 1H), 8.00 (d, J = 6.7 Hz, 1H), 7.94 (d, J = 7.5 Hz, 1H), 7.69 – 7.58 (m, 3H), 7.55 – 7.47 (m, 3H), 7.43 (q, J = 8.6 Hz, 2H), 7.36 – 7.25 (m, 3H), 7.21 (t, J = 7.3 Hz, 1H), 6.97 (d, J = 8.3 Hz, 1H), 6.92 (t, J = 7.5 Hz, 1H), 6.51 – 6.41 (m, 2H), 6.15 (d, J = 13.4 Hz, 1H), 5.51 (s, 2H), 5.05 – 4.98 (m, 1H), 4.60 (t, J = 9.3 Hz, 1H), 4.54 (t, J = 7.9 Hz, 1H), 4.41 (dd, J = 11.9, 6.9 Hz, 1H), 4.28 (q, J = 7.1 Hz, 1H), 4.17 – 4.11 (m, 2H), 3.88 (dd, J = 16.0, 11.4 Hz, 1H), 3.52 – 3.40 (m, 3H), 3.38 – 3.34 (m, 1H), 3.30 – 3.24 (m, 1H), 2.33 – 2.23 (m, 2H), 2.12 (t, J = 7.4 Hz, 2H), 2.05 – 1.93 (m, 2H), 1.78 – 1.72 (m, 6H), 1.70 – 1.63 (m, 8H), 1.60 – 1.50 (m, 4H), 1.50 – 1.32 (m, 9H), 1.26 – 1.18 (m, 24H), 0.84 (t, J = 7.1 Hz, 3H).
^{13}C NMR (151 MHz, DMSO-d_6) δ 174.6, 174.2, 172.6, 172.0, 171.5, 170.9, 169.7, 169.3, 168.6, 165.8, 159.0, 155.3, 152.9, 146.9, 143.3, 142.3, 141.5 (×2), 140.3, 133.9, 129.5, 128.4, 127.9, 127.6, 126.0, 125.5, 124.0, 122.5, 118.9, 116.4, 111.8, 110.2, 109.7, 105.0, 102.3, 69.2, 67.1, 53.2, 52.4, 52.3, 51.0, 49.4, 48.4, 46.8, 45.5, 43.7, 35.0, 34.8, 31.6, 31.2, 29.7, 28.9 (×3), 28.8 (×2), 28.7, 28.6, 28.5, 27.4, 26.8, 26.4, 25.9, 25.6, 25.5, 25.0, 24.6, 24.1, 22.3, 22.0, 13.8.
HRMS (ESI-MS) m/z: [M]$^+$; Calcd for $C_{77}H_{103}N_{10}O_{12}$ 1359.7751; found 1359.7731.

N14E, red powder, yield 26%, 20 mg.
^{1}H NMR (800 MHz, DMSO-d_6) δ 11.79 (s, 1H), 9.86 (s, 1H), 9.53 (s, 1H), 8.42 (d, J = 7.2 Hz, 2H), 8.26 (d, J = 8.3 Hz, 2H), 8.11 (t, J = 6.0 Hz, 1H), 8.02 (d, J = 6.7 Hz, 1H), 7.91 (d, J = 7.8 Hz, 1H), 7.70 – 7.65 (m, 2H), 7.61 (d, J = 7.9 Hz, 1H), 7.55 – 7.50 (m, 3H), 7.45 (t, J = 7.8 Hz, 2H), 7.37 (t, J = 7.7 Hz, 1H), 7.33 (t, J = 7.6 Hz, 1H), 7.31 – 7.26 (m, 2H), 6.98 (d, J = 8.4 Hz, 1H), 6.93 (t, J = 7.7 Hz, 1H), 6.48 (t, J = 13.1 Hz, 2H), 5.58 (s, 2H), 5.01 (t, J = 8.8 Hz, 1H), 4.60 (t, J = 9.2 Hz, 1H), 4.54 (t, J = 7.9 Hz, 1H), 4.43 – 4.39 (m, 1H), 4.29 (q, J = 7.1 Hz, 1H), 4.16 – 4.09 (m, 3H), 3.87 (dd, J = 16.0, 11.5 Hz, 1H), 3.51 – 3.48 (m, 1H), 3.45 – 3.41 (m, 3H), 3.27 – 3.24 (m, 1H), 2.29 (t, J = 7.7 Hz, 2H), 2.15 – 2.09 (m, 2H), 1.80 – 1.66 (m, 16H), 1.59 – 1.53 (m, 3H), 1.50 – 1.35 (m, 9H), 1.27 – 1.17 (m, 24H), 0.84 (t, J = 7.1 Hz, 3H).
^{13}C NMR (201 MHz, DMSO-d_6) δ 175.6, 174.3, 173.2, 172.5, 172.1, 170.3, 169.9, 169.2, 166.4, 159.5, 150.7, 147.6, 142.9, 142.1, 141.5, 140.6, 134.5, 129.2 (×2), 128.5, 128.1, 126.3, 125.6, 124.7, 123.2, 123.0, 119.5, 117.0, 112.6, 111.6, 110.3, 104.2, 102.5, 69.8, 67.7, 53.9, 52.9 (×2), 51.6, 49.8, 49.4, 47.4, 44.5, 35.4, 32.1, 31.8, 31.7, 30.5, 30.2, 29.5 (×3), 29.4, 29.3 (×2), 29.2, 28.2, 27.7, 27.6, 27.4, 26.5, 26.2, 26.1, 25.2, 24.7, 22.9, 22.6, 14.4.
HRMS (ESI-MS) m/z: [M]$^+$; Calcd for $C_{75}H_{101}N_{10}O_{12}$ 1333.7595; found 1333.7553.

N14F, red powder, yield 36%, 23 mg.
^{1}H NMR (600 MHz, DMSO-d_6) δ 11.80 (s, 1H), 9.80 (s, 1H), 9.54 (s, 1H), 8.50 (d, J =

7.5 Hz, 1H), 8.42 – 8.33 (m, 1H), 8.26 (d, *J* = 8.2 Hz, 2H), 8.04 (d, *J* = 6.4 Hz, 1H), 7.97 – 7.78 (m, 2H), 7.76 – 7.64 (m, 3H), 7.61 (d, *J* = 7.8 Hz, 1H), 7.55 – 7.50 (m, 2H), 7.48 – 7.42 (m, 2H), 7.39 – 7.31 (m, 2H), 7.31 – 7.24 (m, 2H), 6.99 – 6.90 (m, 2H), 6.49 (t, *J* = 11.8 Hz, 2H), 5.57 (s, 2H), 5.04 (t, *J* = 9.1 Hz, 1H), 4.61 (t, *J* = 9.4 Hz, 1H), 4.57 – 4.47 (m, 2H), 4.43 – 4.37 (m, 1H), 4.20 – 4.09 (m, 3H), 3.90 – 3.83 (m, 1H), 3.51 – 3.40 (m, 3H), 2.32 – 2.24 (m, 2H), 2.21 – 2.14 (m, 2H), 2.03 – 1.96 (m, 1H), 1.82 – 1.76 (m, 6H), 1.72 – 1.62 (m, 9H), 1.60 – 1.50 (m, 4H), 1.48 – 1.34 (m, 9H), 1.24 – 1.18 (m, 24H), 1.04 – 0.99 (m, 3H), 0.85 (t, *J* = 6.7 Hz, 3H).
^{13}C NMR (151 MHz, DMSO-*d*$_6$) δ 175.0, 173.7, 172.6, 172.4, 170.5, 169.9, 168.5, 168.1, 165.7, 159.0, 150.2, 147.0, 142.3, 141.5, 140.9, 133.9, 129.5, 128.6, 127.9, 127.6, 125.8, 125.0, 124.1, 122.6, 122.5, 118.9, 118.3, 116.5, 112.0, 111.0, 109.8, 103.7, 101.9, 69.2, 67.2, 55.1, 53.3, 52.3, 50.8, 49.2, 48.8, 46.8, 46.0, 44.0, 35.0, 34.8, 31.6, 31.2, 31.1, 30.3, 28.9 (×3), 28.8 (×2), 28.7, 28.6, 27.6, 27.1, 26.9, 25.9, 25.6, 25.5, 25.0, 24.7, 24.1, 22.4, 22.0, 16.0, 13.8.
HRMS (ESI-MS) *m/z*: [M]$^+$; Calcd for $C_{76}H_{103}N_{10}O_{12}$ 1347.7751; found 1347.7752.

N14G, red powder, yield 21%, 20 mg.
^{1}H NMR (600 MHz, DMSO-*d*$_6$) δ 11.81 (s, 1H), 9.79 (s, 1H), 9.54 (s, 1H), 8.44 (d, *J* = 7.8 Hz, 1H), 8.02 (d, *J* = 8.4 Hz, 2H), 7.80 – 7.57 (m, 5H), 7.54 – 7.48 (m, 1H), 7.45 (t, *J* = 7.8 Hz, 1H), 7.27 – 7.03 (m, 5H), 6.98 (d, *J* = 8.3 Hz, 1H), 6.95 – 6.92 (m, 2H), 5.07 – 5.02 (m, 1H), 4.61 (t, *J* = 9.3 Hz, 1H), 4.58 – 4.49 (m, 2H), 4.41 (dd, *J* = 11.7, 6.6 Hz, 1H), 4.24 (q, *J* = 7.1 Hz, 1H), 4.13 (q, *J* = 7.2 Hz, 1H), 3.91 – 3.85 (m, 1H), 3.69 – 3.60 (m, 8H), 3.52 – 3.23 (m, 12H), 2.63 – 2.53 (m, 2H), 2.45 – 2.37 (m, 2H), 2.28 (t, *J* = 7.6 Hz, 2H), 2.04 – 1.95 (m, 1H), 1.87 – 1.80 (m, 1H), 1.77 (d, *J* = 13.2 Hz, 1H), 1.71 – 1.60 (m, 3H), 1.52 – 1.35 (m, 7H), 1.26 – 1.16 (m, 32H), 1.05 – 0.97 (m, 3H), 0.84 (t, *J* = 6.9 Hz, 3H).
^{13}C NMR (151 MHz, DMSO-*d*$_6$) δ 174.2, 172.6, 172.1, 170.3, 170.1, 169.7, 168.4, 168.2, 166.5, 165.7, 159.0, 157.8, 157.6, 157.0, 155.5, 155.0, 135.1, 133.9, 131.7, 130.3, 129.7, 129.5, 127.9, 127.4, 119.9, 118.9, 117.9, 116.5, 115.9, 114.2, 112.9, 109.8, 95.8, 69.3, 67.1, 55.1, 53.0, 52.2, 50.8, 46.9, 46.0, 45.3, 44.5, 44.1, 41.0, 35.0, 31.6, 31.3, 31.2, 30.3, 29.0, 28.9 (×2), 28.8 (×3), 28.6, 28.5, 27.9, 26.9, 26.5, 26.0, 25.5, 25.0, 24.1, 22.4, 22.0, 15.6, 13.8, 12.3.
HRMS (ESI-MS) *m/z*: [M]$^+$; Calcd for $C_{76}H_{106}N_{11}O_{13}$ 1380.7966; found 1380.7970.

N14H, red powder, yield 23%, 12 mg.
^{1}H NMR (600 MHz, DMSO-*d*$_6$) δ 11.77 (s, 1H), 9.87 (s, 1H), 9.52 (s, 1H), 8.48 – 8.33 (m, 2H), 8.29 – 8.21 (m, 2H), 8.10 – 8.00 (m, 2H), 7.87 – 7.79 (m, 1H), 7.71 – 7.57 (m, 3H), 7.55 – 7.50 (m, 2H), 7.49 – 7.42 (m, 2H), 7.41 – 7.31 (m, 2H), 7.31 – 7.22 (m, 2H), 7.02 – 6.87 (m, 3H), 6.54 – 6.37 (m, 2H), 5.58 (s, 2H), 5.04 – 4.98 (m, 1H), 4.64 – 4.58 (m, 1H), 4.51 – 4.41 (m, 2H), 4.32 – 4.26 (m, 1H), 4.13 – 4.06 (m, 3H), 3.91 – 3.83 (m, 1H), 3.51 – 3.41 (m, 3H), 2.34 – 2.26 (m, 2H), 2.19 – 2.07 (m, 2H), 1.81 – 1.64 (m, 13H), 1.63 – 1.56 (m, 3H), 1.55 – 1.34 (m, 13H), 1.29 – 1.18 (m, 24H), 1.01 – 0.96 (m, 3H), 0.84 (t, *J* = 6.8 Hz, 3H).
^{13}C NMR (151 MHz, DMSO-*d*$_6$) δ 175.0, 173.7, 172.6, 171.9, 170.2, 169.7, 169.0,

168.2, 165.8, 163.0, 160.7, 159.0, 150.2, 147.0, 141.5, 140.9, 133.9, 128.7, 127.9, 127.6, 125.8, 125.0, 124.1, 122.6, 118.9, 116.4, 112.0, 111.0, 109.7 (×2), 107.2, 103.7, 101.9, 69.3, 67.0, 55.8, 53.5, 52.4, 51.0, 49.2, 48.8, 46.8, 45.4, 44.0, 34.7, 31.6 (×2), 31.2, 29.5, 28.9 (×3), 28.8 (×2), 28.7, 28.6, 28.2, 27.9, 27.6, 27.1, 26.8, 26.0, 25.6, 25.5, 24.8, 24.1, 22.3, 22.0, 16.1, 13.8.
HRMS (ESI-MS) *m/z*: $[M]^+$; Calcd for $C_{76}H_{103}N_{10}O_{12}$ 1347.7751; found 1347.7751.

N14I, red powder, yield 24%, 26 mg.
^{1}H NMR (600 MHz, DMSO-d_6) δ 11.78 (s, 1H), 9.90 (s, 1H), 9.63 (s, 1H), 8.53 – 8.43 (m, 1H), 8.11 – 7.95 (m, 3H), 7.79 – 7.73 (m, 2H), 7.72 – 7.68 (m, 1H), 7.61 (d, *J* = 7.8 Hz, 1H), 7.52 (s, 1H), 7.45 (t, *J* = 7.9 Hz, 1H), 7.19 – 7.07 (m, 4H), 6.99 (d, *J* = 8.4 Hz, 1H), 6.96 – 6.89 (m, 3H), 5.01 (t, *J* = 8.6 Hz, 1H), 4.62 (t, *J* = 9.5 Hz, 1H), 4.50 (t, *J* = 7.9 Hz, 1H), 4.46 – 4.40 (m, 1H), 4.32 – 4.25 (m, 1H), 4.19 – 4.08 (m, 2H), 3.91 – 3.83 (m, 1H), 3.65 (q, *J* = 7.5 Hz, 8H), 3.52 – 3.36 (m, 6H), 3.32 – 3.19 (m, 6H), 2.45 – 2.36 (m, 2H), 2.34 – 2.25 (m, 2H), 1.82 – 1.33 (m, 13H), 1.32 – 1.11 (m, 33H), 1.08 (t, *J* = 7.0 Hz, 1H), 1.01 (d, *J* = 6.8 Hz, 3H), 0.84 (t, *J* = 6.8 Hz, 3H).
^{13}C NMR (151 MHz, DMSO-d_6) δ 172.6, 171.4, 170.2, 169.9, 169.7, 168.9, 168.2, 166.5, 165.8, 159.0, 157.0, 155.5, 155.0, 151.7, 147.9, 135.2, 133.9, 131.7, 130.6, 130.3, 129.7, 127.9, 127.4, 119.0, 116.4, 114.2, 112.9, 112.9, 109.7, 107.8, 107.7, 97.4, 95.8, 69.3, 67.1, 58.8, 55.9, 53.4, 52.4, 51.0, 46.8, 45.4, 45.3, 44.5, 44.1, 43.6, 31.6, 31.2, 30.3, 30.1, 29.5, 29.0, 28.9 (×2), 28.8 (×3), 28.6, 27.8, 27.7, 26.8, 26.0, 25.5, 24.1, 22.3, 22.0, 16.3, 13.8, 12.3 (×2).
HRMS (ESI-MS) *m/z*: $[M]^+$; Calcd for $C_{76}H_{106}N_{11}O_{13}$ 1380.7966; found 1380.7975.

N14J, red powder, yield 28%, 29 mg.
^{1}H NMR (800 MHz, DMSO-d_6) δ 13.37 (s, 1H), 11.77 (s, 1H), 9.83 (s, 1H), 9.50 (s, 1H), 8.77 (d, *J* = 7.7 Hz, 1H), 8.41 (d, *J* = 7.4 Hz, 1H), 8.32 – 8.12 (m, 4H), 7.84 (s, 1H), 7.59 (d, *J* = 7.9 Hz, 1H), 7.46 (t, *J* = 7.9 Hz, 1H), 7.06 – 6.84 (m, 4H), 6.75 – 6.38 (m, 4H), 4.94 (t, *J* = 8.8 Hz, 1H), 4.56 – 4.50 (m, 2H), 4.46 – 4.38 (m, 2H), 4.26 – 4.11 (m, 7H), 3.89 – 3.84 (m, 1H), 3.53 – 3.43 (m, 4H), 2.45 – 2.39 (m, 3H), 2.32 – 2.26 (m, 2H), 2.06 – 1.94 (m, 1H), 1.78 (dd, *J* = 34.7, 13.3 Hz, 2H), 1.70 – 1.61 (m, 3H), 1.56 – 1.50 (m, 1H), 1.46 – 1.39 (m, 6H), 1.37 – 1.33 (m, 1H), 1.25 – 1.20 (m, 24H), 0.85 (t, *J* = 6.9 Hz, 3H).
^{19}F NMR (376 MHz, DMSO-d_6) δ -73.6.
^{13}C NMR (201 MHz, DMSO-d_6) δ 174.2, 172.8, 172.6, 171.7, 169.6, 168.8, 168.5, 165.7, 164.8, 159.0, 157.8, 157.7, 157.5, 139.1, 133.9, 129.5, 129.0, 128.2, 127.9, 127.4, 124.1, 119.4, 118.9, 117.9, 116.4 (×2), 114.9, 109.8, 102.1, 71.3, 69.2, 67.1, 53.7, 53.1, 52.2, 51.7, 51.1, 46.7, 35.0, 34.3, 31.6, 31.2, 31.1, 30.3, 29.7, 29.0, 28.9 (×2), 28.8 (×2), 28.7, 28.6, 28.5, 26.8, 26.5, 25.9, 25.5, 25.0, 24.1, 22.3, 22.0, 15.4, 13.8.
HRMS (ESI-MS) *m/z*: $[M]^+$; Calcd for $C_{66}H_{84}N_9O_{13}$ 1210.6183; found 1210.6190.

N14K, red powder, yield 30%, 25 mg.
^{1}H NMR (800 MHz, DMSO-d_6) δ 11.81 (s, 1H), 9.85 (s, 1H), 9.55 (s, 1H), 8.43 (s, 1H), 8.13 – 7.96 (m, 3H), 7.80 – 7.66 (m, 3H), 7.62 (d, *J* = 7.9 Hz, 1H), 7.55 – 7.49 (m, 1H),

7.45 (t, J = 8.0 Hz, 1H), 7.19 – 7.07 (m, 4H), 6.98 (d, J = 8.3 Hz, 1H), 6.96 – 6.89 (m, 3H), 5.02 (t, J = 9.0 Hz, 1H), 4.61 (t, J = 9.4 Hz, 1H), 4.57 – 4.51 (m, 1H), 4.41 (t, J = 9.3 Hz, 1H), 4.29 (q, J = 7.6 Hz, 1H), 4.20 – 4.13 (m, 1H), 3.88 (t, J = 13.8 Hz, 1H), 3.65 (q, J = 7.8 Hz, 8H), 3.52 – 3.42 (m, 4H), 3.39 – 3.29 (m, 6H), 2.52 (s, 1H), 2.42 – 2.33 (m, 2H), 2.32 – 2.25 (m, 2H), 1.79 (d, J = 13.3 Hz, 1H), 1.75 – 1.60 (m, 4H), 1.59 – 1.53 (m, 1H), 1.51 – 1.46 (m, 2H), 1.44 – 1.40 (m, 3H), 1.37 – 1.32 (m, 1H), 1.28 – 1.17 (m, 38H), 0.84 (t, J = 7.3 Hz, 3H).
^{13}C NMR (201 MHz, DMSO-d_6) δ 173.2, 172.1, 171.9, 170.5, 170.2, 169.8, 169.2, 167.1, 166.3, 159.6, 158.4, 158.3, 157.5, 156.1, 155.6, 139.7, 135.7, 134.5, 132.2, 130.9, 130.2, 128.5, 128.0, 124.6, 119.5, 118.0, 117.0, 116.5, 114.7, 113.5 (×2), 110.3, 96.4, 70.3, 69.8, 67.7, 53.7, 52.9, 52.8, 51.6, 47.4, 45.9, 45.1, 44.7, 41.9, 41.6, 35.6, 34.9, 32.1, 31.8, 30.9, 30.8, 30.2, 29.5 (×3), 29.4 (×2), 29.3, 29.2, 28.3, 27.4, 26.5, 26.1, 24.7, 22.9, 22.6, 14.4, 12.9.
HRMS (ESI-MS) *m/z*: [M]$^+$; Calcd for $C_{75}H_{104}N_{11}O_{13}$ 1366.7810; found 1366.7814.

N14L, red powder, yield 30%, 23 mg.
^{1}H NMR (600 MHz, DMSO-d_6) δ 13.34 (s, 1H), 11.79 (s, 1H), 9.85 (s, 1H), 9.54 (s, 1H), 8.42 (d, J = 7.6 Hz, 1H), 8.26 (d, J = 7.9 Hz, 1H), 8.08 – 7.99 (m, 3H), 7.80 (d, J = 8.1 Hz, 1H), 7.61 (d, J = 7.7 Hz, 1H), 7.49 – 7.42 (m, 2H), 7.07 – 6.95 (m, 3H), 6.92 (t, J = 7.6 Hz, 1H), 6.66 – 6.51 (m, 4H), 5.01 (dd, J = 10.0, 7.5 Hz, 1H), 4.61 (t, J = 9.2 Hz, 1H), 4.53 (t, J = 7.9 Hz, 1H), 4.41 (dd, J = 11.9, 6.9 Hz, 1H), 4.33 – 4.22 (m, 8H), 4.19 – 4.15 (m, 1H), 3.91 – 3.85 (m, 1H), 3.62 – 3.49 (m, 8H), 3.34 – 3.31 (m, 2H), 2.62 – 2.53 (m, 2H), 2.48 – 2.42 (m, 4H), 2.41 – 2.35 (m, 2H), 2.28 (t, J = 7.5 Hz, 2H), 1.81 – 1.53 (m, 7H), 1.49 – 1.39 (m, 6H), 1.25 – 1.19 (m, 24H), 0.85 (t, J = 6.9 Hz, 3H).
^{19}F NMR (376 MHz, DMSO) δ -73.7.
^{13}C NMR (151 MHz, DMSO-d_6) δ 172.7, 171.7, 171.5, 170.2, 169.3, 168.7, 167.4, 165.9, 164.6, 159.1, 157.9, 157.7, 156.5, 156.0, 134.0, 130.8, 128.6, 128.0, 119.0, 118.1, 116.5, 116.1, 112.9, 109.8, 94.0, 69.3, 67.2, 53.2, 52.5, 52.4, 51.8, 51.1, 46.9, 31.7, 31.3, 30.4, 29.7, 29.1, 29.0 (×3), 28.9 (×2), 28.7, 27.9, 26.9, 26.1, 25.6, 24.2, 22.4, 22.1, 15.5, 14.0.
HRMS (ESI-MS) *m/z*: [M]$^+$; Calcd for $C_{74}H_{96}N_{11}O_{15}$ 1378.7082; found 1378.7086.

N14G-Bz, red powder, yield 61%, 38 mg.
^{1}H NMR (500 MHz, DMSO-d_6) δ 11.80 (s, 1H), 8.45 (d, J = 8.1 Hz, 1H), 8.09 (d, J = 6.8 Hz, 1H), 8.03 – 7.93 (m, 4H), 7.78 – 7.63 (m, 5H), 7.63 – 7.34 (m, 7H), 7.20 – 6.99 (m, 4H), 6.94 – 6.88 (m, 3H), 5.03 (dd, J = 9.9, 7.6 Hz, 1H), 4.68 – 4.46 (m, 4H), 4.26 (q, J = 6.8 Hz, 1H), 4.23 – 4.11 (m, 2H), 3.82 – 3.75 (m, 1H), 3.71 – 3.59 (m, 9H), 3.58 – 3.42 (m, 8H), 3.39 – 3.14 (m, 7H), 2.60 – 2.51 (m, 2H), 2.46 – 2.33 (m, 2H), 2.28 – 2.13 (m, 2H), 1.97 – 1.90 (m, 1H), 1.88 – 1.81 (m, 1H), 1.80 – 1.69 (m, 3H), 1.69 – 1.58 (m, 2H), 1.57 – 1.37 (m, 5H), 1.34 – 1.09 (m, 31H), 0.99 (d, 3H), 0.83 (t, J = 6.9 Hz, 3H).
^{13}C NMR (126 MHz, DMSO) δ 172.0, 170.2, 169.5, 168.5, 166.5, 165.7, 163.2, 158.9, 157.8, 157.5, 156.9, 155.5, 155.0, 135.1, 134.3, 133.8, 131.6, 130.3, 129.7, 129.5, 128.9, 127.9, 127.4, 126.5, 118.9, 118.1, 116.4, 114.1, 112.9, 109.7, 95.8, 69.1, 67.1, 55.0,

52.7, 52.2, 51.2, 45.9, 45.3, 31.3, 31.2, 30.2, 28.9 (×2), 28.8, 28.7, 28.6, 28.3, 26.9, 25.7, 22.0, 15.6, 13.8, 12.3.
HRMS (ESI-MS) *m/z*: $[M]^+$; Calcd for $C_{90}H_{114}N_{11}O_{15}$ 1588.8490; found 1588.8475.

2.6 Synthesis of P10C-Fe, N14G-Fe and N14I-Fe

To a stirred solution of **P10C** (6 mg, 0.007 mmol) or **N14G** (10 mg, 0.007 mmol) or **N14I** (10 mg, 0.007 mmol) in MeOH (1 mL) was added $FeCl_3$ (2.5 mg, 0.014 mmol, 2 equiv.). After stirring at r.t. for 10 min, the reaction was concentrated and purified by a reverse C18 column chromatography (H_2O:MeOH/95:5 to 5:95) and lyophilized to afford **P10C-Fe**, **N14G-Fe** and **N14I-Fe**, yields 90–95%, 7–12 mg.

2.7 Synthesis of cMbT

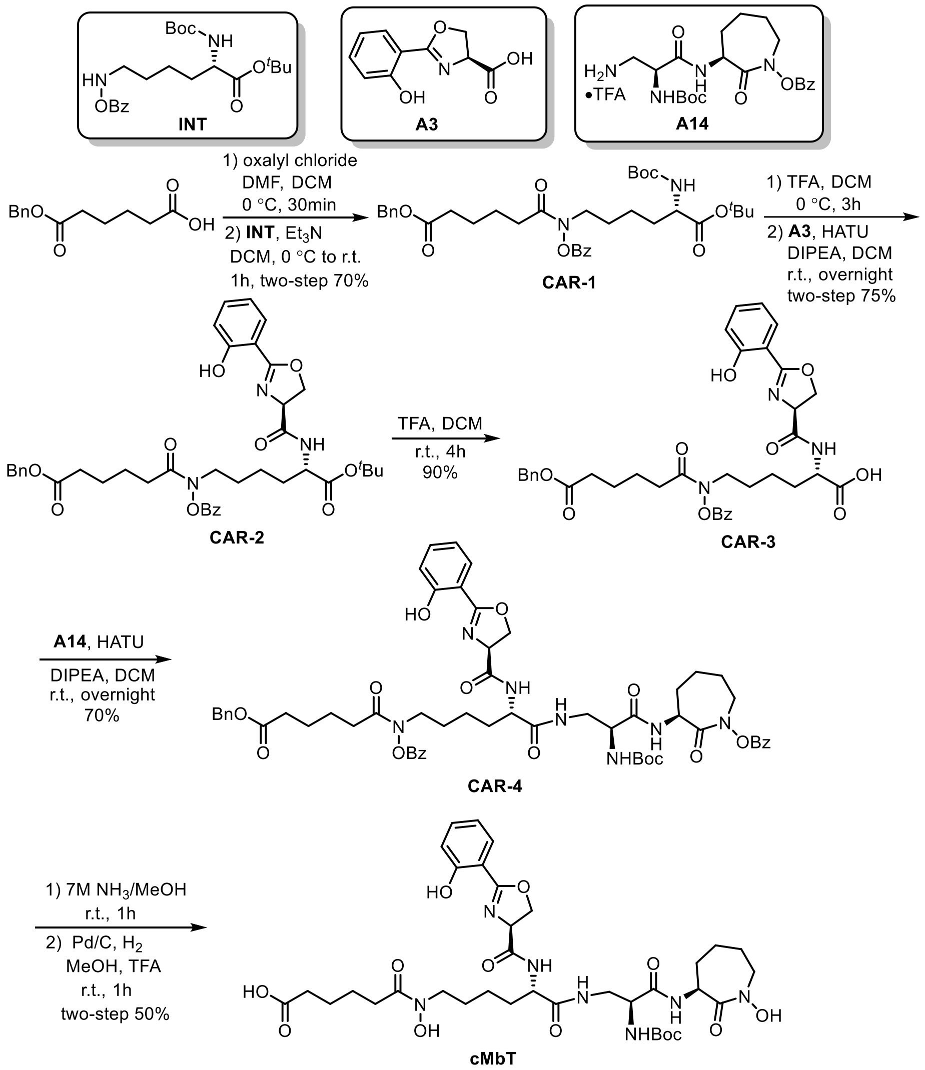

Scheme S12. Synthesis of **cMbT**

INT was prepared and characterized as reference.[6]

Benzyl (*S*)-6-((benzoyloxy)(5-(((benzyloxy)carbonyl)amino)-6-(*tert*-butoxy)-6-oxohexyl)amino)-6-oxohexanoate (CAT-1)

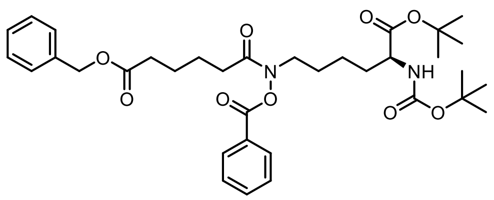

CAR-1

To a stirred solution of 6-(benzyloxy)-6-oxohexanoic acid (1.18 g, 5 mmol) in dry DCM (10 mL) was added oxalyl chloride (762 mg, 6 mmol, 1.2 equiv.) and dry DMF (a drop) at ice bath. After stirring at ice bath for 30 min, the reaction was concentrated in vacuo to afford crude intermediate acyl chloride as colorless oil, which was resolved in dry DCM (5 mL) and add to the stirred solution of **INT** (2.2 g, 5 mmol, 1 equiv.) and Et_3N (1.5 g, 15 mmol, 3 equiv.) in dry DCM (10 mL) at ice bath. After stirring at r.t. for 1 h, the reaction was concentrated and purified by silica gel column chromatography (PE:EA/100:0 to PE:EA/75:25) to afford **CAR-1**. R_f = 0.7 (PE:EA/3:1), colorless oil, two-step yield 70 %, 2.36 g.

^{1}H NMR (400 MHz, $CDCl_3$) δ 8.09 (d, J = 7.3 Hz, 2H), 7.68 (t, J = 7.5 Hz, 1H), 7.52 (t, J = 7.7 Hz, 2H), 7.38 – 7.28 (m, 5H), 5.12 – 4.98 (m, 3H), 4.15 (q, J = 7.2 Hz, 1H), 3.79 (t, J = 7.2 Hz, 2H), 2.41 – 2.24 (m, 4H), 1.84 – 1.57 (m, 10H), 1.44 (d, J = 8.7 Hz, 18H).

^{13}C NMR (101 MHz, $CDCl_3$) δ 173.2, 171.9, 164.5, 155.4, 136.0, 134.5, 130.0, 129.0, 128.5, 128.2, 126.8, 81.8, 79.6, 66.2, 53.8, 34.0, 32.6, 31.8, 28.3, 28.0, 26.7, 24.4, 23.7, 22.4.

HRMS (ESI-MS) m/z: $[M+H]^+$; Calcd for $C_{35}H_{49}N_2O_9$ 641.3433; Found 641.3427.

Benzyl 6-((benzoyloxy)((*S*)-6-(*tert*-butoxy)-5-((*S*)-2-(2-hydroxyphenyl)-4,5-dihydrooxazole-4-carboxamido)-6-oxohexyl)amino)-6-oxohexanoate (CAR-2)

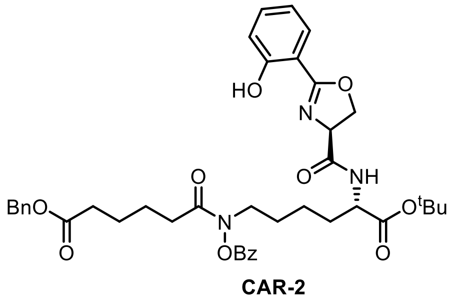

CAR-2

To a stirred solution of the **CAR-1** (1.5 g, 2.0 mmol) in DCM (10 mL) was added TFA (5 mL) dropwise at ice bath, and stirred at ice bath for 3 h (monitored by LC-MS analysis, if Boc incompletely deprotected, then continued drop TFA until complete conversion). After the reaction completed, the solution was evaporated in vacuo to get crude amine as a colorless oil, that was used directly in the next step.

The above intermediate oil and **A3** (446 mg, 2.0 mmol, 1.0 equiv.) were dissolved in DCM (10 mL), followed by the addition of HATU (836 mg, 2.2 mmol, 1.1 equiv.) and DIPEA (775 mg, 6.0 mmol, 3.0 equiv.). After stirring at r.t. overnight, the reaction was diluted with EtOAc (20 mL) and washed sequentially with 1 M HCl (aq., 15 mL), H_2O (15 mL) and brine (15 mL). The organic layer was dried over anhydrous Na_2SO_4,

filtered, and concentrated in vacuo. The mixture was purified by silica gel column chromatography (PE:EA/100:0 to PE:EA/50:50) to afford **CAR-2**. R_f = 0.7 (PE:EA/1:1), colorless oil, two-step yield 75%, 1 g.

^{1}H NMR (400 MHz, $CDCl_3$) δ 11.38 (s, 1H), 8.09 – 7.98 (m, 2H), 7.70 – 7.62 (m, 2H), 7.50 (t, J = 7.7 Hz, 2H), 7.42 – 7.36 (m, 1H), 7.35 – 7.26 (m, 5H), 7.00 (d, J = 8.4 Hz, 1H), 6.91 – 6.83 (m, 2H), 5.07 (s, 2H), 4.97 – 4.91 (m, 1H), 4.70 – 4.58 (m, 2H), 4.50 – 4.41 (m, 1H), 3.75 (t, J = 7.1 Hz, 2H), 2.38 – 2.22 (m, 4H), 1.88 – 1.79 (m, 1H), 1.70 – 1.57 (m, 7H), 1.47 (s, 9H), 1.40 – 1.31 (m, 2H).

^{13}C NMR (101 MHz, $CDCl_3$) δ 173.2, 170.7, 170.3, 167.8, 164.5, 159.8, 136.0, 134.5, 134.3, 130.0, 128.9, 128.6, 128.5, 128.2, 126.7, 119.1, 117.0, 110.0, 82.5, 69.7, 68.1, 66.1, 52.6, 34.0, 32.2, 31.8, 28.0, 26.6, 24.4, 23.7, 22.4.

HRMS (ESI-MS) m/z: $[M+H]^+$; Calcd for $C_{40}H_{48}N_3O_{10}$ 730.3334; Found 730.3330.

N^6-(benzoyloxy)-N^6-(6-(benzyloxy)-6-oxohexanoyl)-N^2-((*S*)-2-(2-hydroxyphenyl)-4,5-dihydrooxazole-4-carbonyl)-L-lysine (CAR-3)

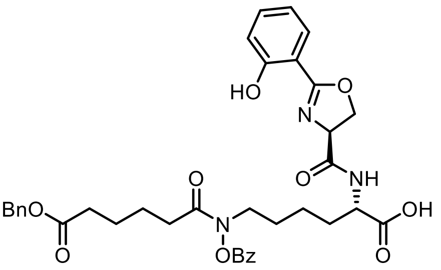

CAR-3

To a solution of the **CAR-2** (875 mg, 1.2 mmol) in DCM (10 mL) was added TFA (10 mL) dropwise at ice bath. Then the solution was stirred at r.t. for 4 h until the **CAR-2** disappeared in LC-MS analysis and then evaporated in vacuo, purified by reverse C18 column chromatography (H_2O:MeOH/95:5 to 5:95) and lyophilized to afford **CAR-3** as colorless oil. Yield, 90%, 724 mg.

^{1}H NMR (400 MHz, $CDCl_3$) δ 8.04 (d, J = 7.6 Hz, 2H), 7.68 – 7.62 (m, 2H), 7.49 (t, J = 7.7 Hz, 2H), 7.39 – 7.28 (m, 6H), 7.20 – 7.14 (m, 1H), 6.96 (d, J = 8.3 Hz, 1H), 6.86 (t, J = 7.6 Hz, 1H), 5.08 (s, 2H), 4.98 (t, J = 9.5 Hz, 1H), 4.68 – 4.57 (m, 3H), 3.89 – 3.70 (m, 2H), 2.38 – 2.23 (m, 4H), 1.97 – 1.77 (m, 2H), 1.68 – 1.55 (m, 6H), 1.50 – 1.36 (m, 2H).

^{13}C NMR (101 MHz, $CDCl_3$) δ 173.7, 170.8, 167.8, 164.4, 159.8, 135.9, 134.5, 134.2, 130.1, 129.0, 128.6, 128.2 (×2), 126.7, 119.1, 117.0, 110.1, 69.5, 68.1, 66.4, 52.1, 34.0, 31.7, 31.4, 26.5, 24.4, 23.8, 22.2.

HRMS (ESI-MS) m/z: $[M+H]^+$; Calcd for $C_{36}H_{40}N_3O_{10}$ 674.2708; Found 674.2705.

Benzyl (6*S*,10*S*)-15-(benzoyloxy)-6-(((*S*)-1-(benzoyloxy)-2-oxoazepan-3-yl)carbamoyl)-10-((*S*)-2-(2-hydroxyphenyl)-4,5-dihydrooxazole-4-carboxamido)-2,2-dimethyl-4,9,16-trioxo-3-oxa-5,8,15-triazahenicosan-21-oate (CAR-4)

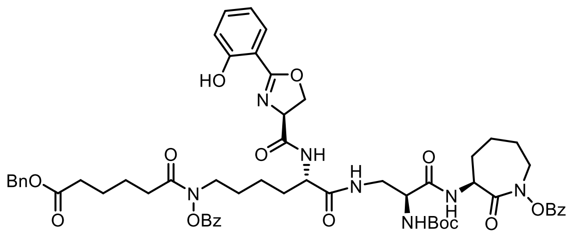

CAR-4

The **CAR-3** (538 mg, 0.8 mmol) and **A14** (424 mg, 0.8 mmol, 1.0 equiv.) were dissolved in DCM (5 mL) and DMF (5 mL), followed by the addition of HATU (334 mg, 0.88 mmol, 1.1 equiv.) and DIPEA (310 mg, 2.4 mmol, 3.0 equiv.). After stirring at r.t. overnight, the reaction was diluted with EtOAc (10 mL) and washed sequentially with 1 M HCl (aq., 10 mL), H_2O (10 mL) and brine (10 mL). The organic layer was dried over anhydrous Na_2SO_4, filtered, and concentrated in vacuo, then purified by reverse-phase C18 column chromatography (H_2O:MeOH/95:5 to 5:95) and lyophilized to afford **CAR-4**. White solid, yield 70%, 609 mg.

^{1}H NMR (800 MHz, DMSO-d_6) δ 11.78 (s, 1H), 8.44 (d, J = 8.0 Hz, 1H), 8.07 – 7.92 (m, 6H), 7.76 – 7.68 (m, 2H), 7.62 – 7.50 (m, 5H), 7.42 (t, J = 8.0 Hz, 1H), 7.38 – 7.26 (m, 5H), 7.00 – 6.88 (m, 3H), 5.05 (s, 2H), 4.97 (t, J = 8.3 Hz, 1H), 4.63 (t, J = 9.4 Hz, 1H), 4.59 – 4.51 (m, 2H), 4.27 – 4.16 (m, 2H), 4.04 (q, J = 7.4 Hz, 1H), 3.81 – 3.76 (m, 1H), 3.66 (s, 2H), 3.40 – 3.34 (m, 1H), 3.27 – 3.20 (m, 1H), 2.35 – 2.19 (m, 4H), 1.90 (d, J = 12.0 Hz, 1H), 1.84 (d, J = 13.9 Hz, 1H), 1.79 – 1.66 (m, 4H), 1.63 – 1.46 (m, 8H), 1.37 – 1.25 (m, 11H).

^{13}C NMR (201 MHz, DMSO-d_6) δ 172.6, 171.7, 169.6, 165.8, 163.2, 159.0, 155.1, 136.2, 134.4, 133.9, 129.6 (×2), 129.1, 129.0, 128.4, 127.9 (×2), 126.6, 119.0, 116.5, 109.8, 78.4, 69.2, 67.2, 65.3, 54.3, 52.6, 52.3, 51.3, 33.2, 31.5, 31.1, 30.2, 28.1, 27.0, 25.8, 23.8, 22.5.

HRMS (ESI-MS) m/z: $[M+H]^+$; Calcd for $C_{57}H_{68}N_7O_{15}$ 1090.4768; Found 1090.4767.

6-(hydroxy((*S*)-6-((3-(((*S*)-1-hydroxy-2-oxoazepan-3-yl)amino)-3-oxopropyl)amino)-5-((*S*)-2-(2-hydroxyphenyl)-4,5-dihydrooxazole-4-carboxamido)-6-oxohexyl)-amino)-6-oxohexanoic acid (cMbT)

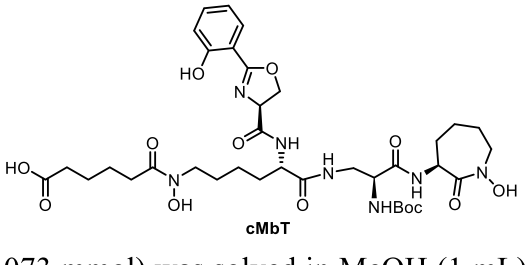

cMbT

The **CAR-4** (80 mg, 0.073 mmol) was solved in MeOH (1 mL), and 7 M NH_3/MeOH was added dropwise. After complete conversion (monitored by LC-MS), the mixture was concentrated in vacuo to afford hydroxamate intermediate as a colorless oil, that was used directly in the next step.

To a stirred solution of the above intermediate oil in MeOH (2 mL) was added 10% Pd/C (16 mg, 10% w/w, 20 %) and TFA (10 μL). The mixture was stirred under H_2 atmosphere at r.t. until complete conversion (monitored by LC-MS), the mixture was

filtrated and filtrate was concentrated in vacuo, then purified by reverse-phase C18 column chromatography (H_2O:MeOH/95:5 to 5:95) and lyophilized to afford **cMbT**. White solid, yield 50 %, 24 mg.

^{1}H NMR (800 MHz, DMSO-d_6) δ 11.82 (s, 2H), 9.87 (s, 1H), 9.54 (s, 1H), 8.45 (d, J = 7.5 Hz, 1H), 8.15 (s, 1H), 7.93 (d, J = 7.0 Hz, 1H), 7.64 (d, J = 7.7 Hz, 1H), 7.46 (t, J = 7.9 Hz, 1H), 7.02 – 6.98 (m, 2H), 6.95 (t, J = 6.5 Hz, 1H), 5.01 (t, J = 8.3 Hz, 1H), 4.62 (t, J = 9.4 Hz, 1H), 4.54 (t, J = 8.0 Hz, 1H), 4.44 (t, J = 9.1 Hz, 1H), 4.20 – 4.15 (m, 1H), 3.97 (q, J = 7.4 Hz, 1H), 3.91 (t, J = 13.9 Hz, 1H), 3.49 (d, J = 11.0 Hz, 1H), 3.44 (t, J = 7.3 Hz, 2H), 3.37 – 3.34 (m, 1H), 3.30 – 3.24 (m, 1H), 2.35 – 2.30 (m, 2H), 2.21 – 2.17 (m, 2H), 1.82 – 1.62 (m, 6H), 1.60 – 1.55 (m, 1H), 1.51 – 1.46 (m, 6H), 1.41 – 1.36 (m, 10H), 1.28 – 1.20 (m, 2H).

^{13}C NMR (201 MHz, DMSO-d_6) δ 174.4, 172.4, 171.7, 169.7, 169.6, 168.7, 165.9, 159.1, 155.1, 134.0, 128.0, 119.1, 116.6, 109.9, 78.4, 69.4, 67.2, 54.3, 53.2, 52.4, 51.0, 47.0, 33.5, 31.4 (×2), 30.0, 28.1, 28.0, 26.9, 26.0, 25.6, 24.3, 23.8, 22.5.

HRMS (ESI-MS) m/z: $[M+H]^+$; Calcd for $C_{36}H_{54}N_7O_{13}$ 792.3774; Found 792.3767.

2.8 HPLC analysis of representative target compounds

The liquid chromatography analysis was performed on an Agilent 1260 HPLC system. The column used Micsphere-C18, particle size 5 μm, dimensions 4.6 mm×250 mm. The eluent was a mixture of H_2O (with 0.5‰ TFA as additive) and MeOH with a linear gradient from 95:5 (v/v) to 5:95 (v/v) within 25 min at a 1 mL/min flow rate for analysis. The UV detection was carried out at UV wavelength of 635 nm (for **N14C**) or 550 nm (for **N14D**, **N14E**, **N14F**, **N14G**, **N14H**, **N14I**, **N14J**, **N14K**, **N14L**, **N14G-Fe**, **N14I-Fe** and **N14G-Bz**).

Elution gradient for analysis:

0 min to 10 min: H_2O (+ TFA, 95%) / MeOH (5%) to H_2O (+TFA, 5%) / MeOH (95%);

10 min to 19 min: H_2O (+TFA, 5%) / MeOH (95%);

19 min to 21 min: H_2O (+ TFA, 5%) / MeOH (95%) to H_2O (+TFA, 95%) / MeOH (5%);

21 min to 25 min H_2O (+TFA, 95%) / MeOH (5%).

Purity of the conjugate **N14C**

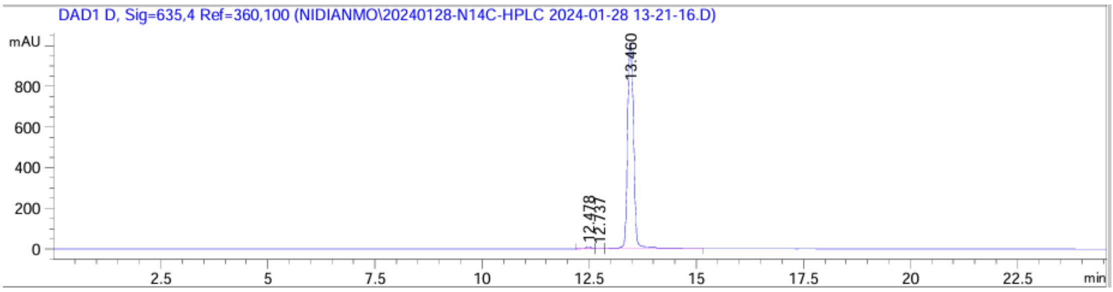

Signal 4: DAD1 D, Sig=635,4 Ref=360,100

Peak #	RetTime [min]	Type	Width [min]	Area [mAU*s]	Height [mAU]	Area %
1	12.478	BV	0.1441	76.32697	8.39249	0.7594
2	12.737	VV	0.1545	19.09120	1.88012	0.1899
3	13.460	VB	0.1590	9955.34375	1010.36957	99.0506
Totals :				1.00508e4	1020.64218	

Purity of the conjugate (**N14D**)

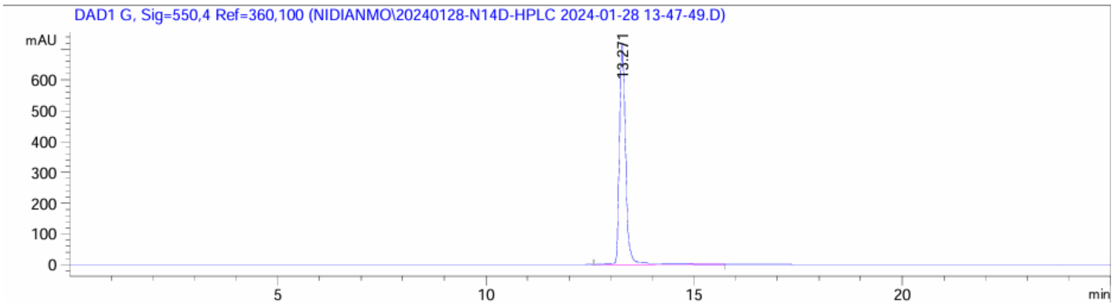

Signal 5: DAD1 G, Sig=550,4 Ref=360,100

Peak #	RetTime [min]	Type	Width [min]	Area [mAU*s]	Height [mAU]	Area %
1	13.271	BB	0.1618	7515.35547	719.94757	100.0000
Totals :				7515.35547	719.94757	

Purity of the conjugate (**N14E**)

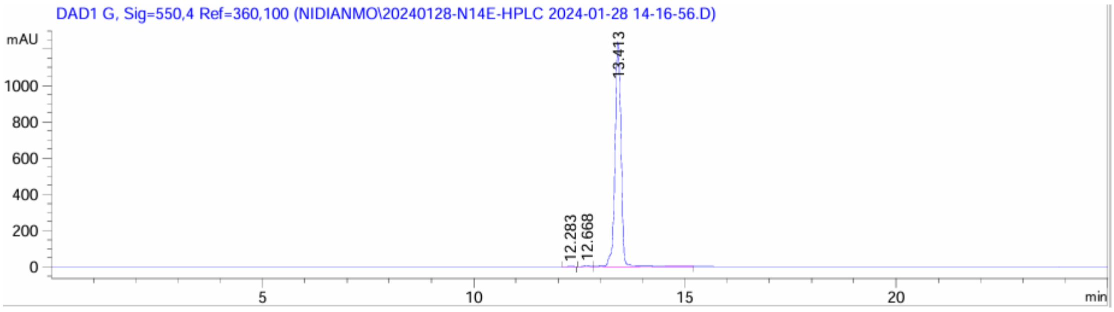

Signal 5: DAD1 G, Sig=550,4 Ref=360,100

Peak #	RetTime [min]	Type	Width [min]	Area [mAU*s]	Height [mAU]	Area %
1	12.283	BB	0.1366	20.23119	2.39329	0.1638
2	12.668	BV	0.1463	57.80488	6.11476	0.4681
3	13.413	VB	0.1596	1.22709e4	1238.11243	99.3681
Totals :				1.23490e4	1246.62048	

Purity of the conjugate (**N14F**)

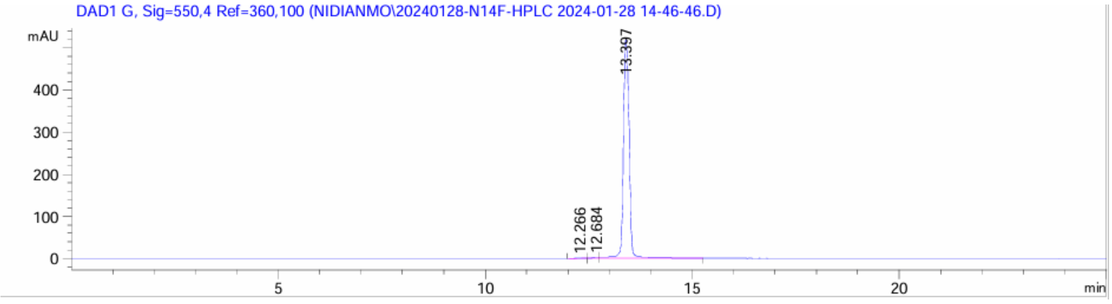

Signal 5: DAD1 G, Sig=550,4 Ref=360,100

Peak #	RetTime [min]	Type	Width [min]	Area [mAU*s]	Height [mAU]	Area %
1	12.266	BB	0.1449	9.92234	1.06273	0.1975
2	12.684	BV	0.1413	21.15607	2.34501	0.4211
3	13.397	VB	0.1559	4992.93652	520.70917	99.3814
Totals :				5024.01494	524.11691	

Purity of the conjugate (**N14G**)

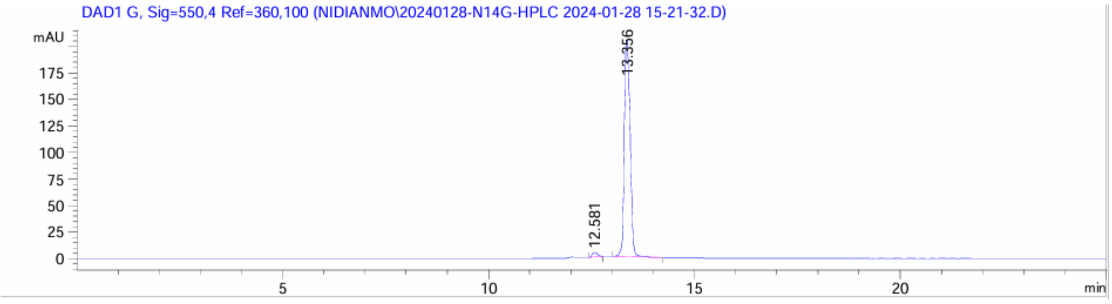

Signal 5: DAD1 G, Sig=550,4 Ref=360,100

Peak #	RetTime [min]	Type	Width [min]	Area [mAU*s]	Height [mAU]	Area %
1	12.581	BB	0.1429	38.15799	4.24472	1.8821
2	13.356	BB	0.1576	1989.27161	204.36584	98.1179
Totals :				2027.42959	208.61057	

Purity of the conjugate (**N14H**)

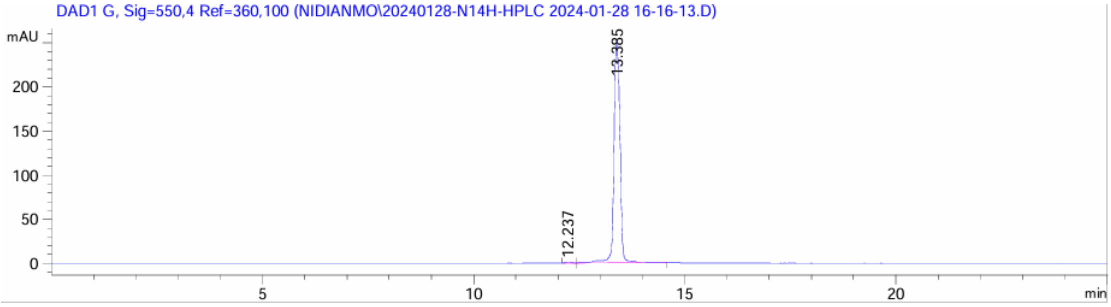

Signal 5: DAD1 G, Sig=550,4 Ref=360,100

Peak #	RetTime [min]	Type	Width [min]	Area [mAU*s]	Height [mAU]	Area %
1	12.237	BB	0.1365	9.28393	1.07795	0.3695
2	13.385	BB	0.1592	2503.56934	253.50333	99.6305

Totals : 2512.85326 254.58127

Purity of the conjugate (**N14I**)

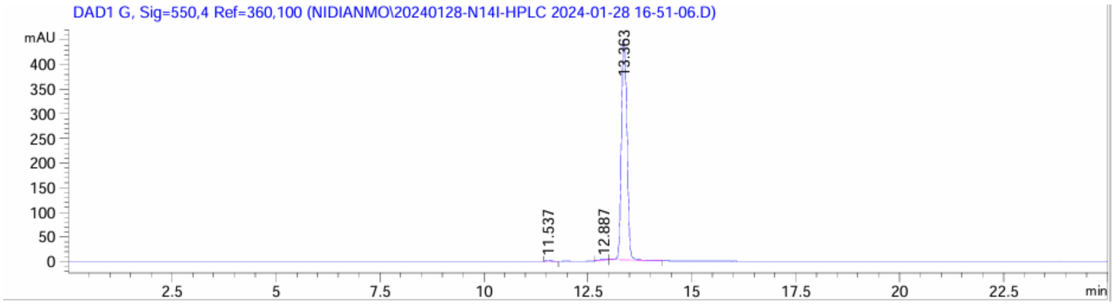

Signal 5: DAD1 G, Sig=550,4 Ref=360,100

Peak #	RetTime [min]	Type	Width [min]	Area [mAU*s]	Height [mAU]	Area %
1	11.537	BB	0.1260	23.79445	3.08592	0.5579
2	12.887	BB	0.1895	16.55133	1.24206	0.3881
3	13.363	BB	0.1564	4224.39014	446.55005	99.0540

Totals : 4264.73592 450.87803

Purity of the conjugate (**N14J**)

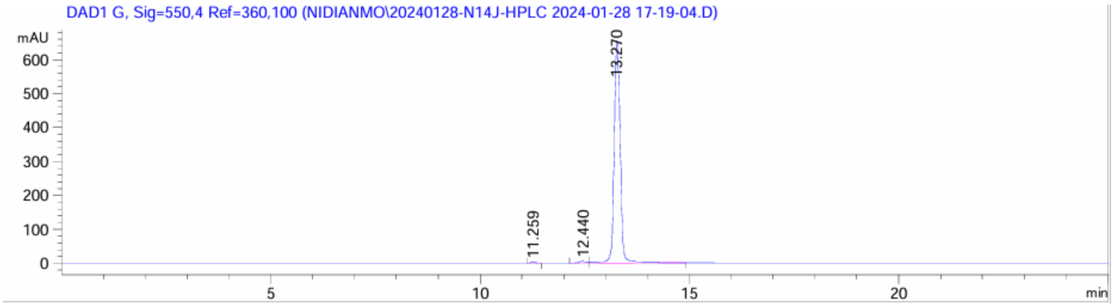

Signal 5: DAD1 G, Sig=550,4 Ref=360,100

Peak #	RetTime [min]	Type	Width [min]	Area [mAU*s]	Height [mAU]	Area %
1	11.259	BB	0.1273	21.43573	2.62454	0.3215
2	12.440	BV	0.1693	55.22907	4.90615	0.8283
3	13.270	VB	0.1613	6590.70654	655.27789	98.8501

Totals : 6667.37135 662.80858

Purity of the conjugate (**N14K**)

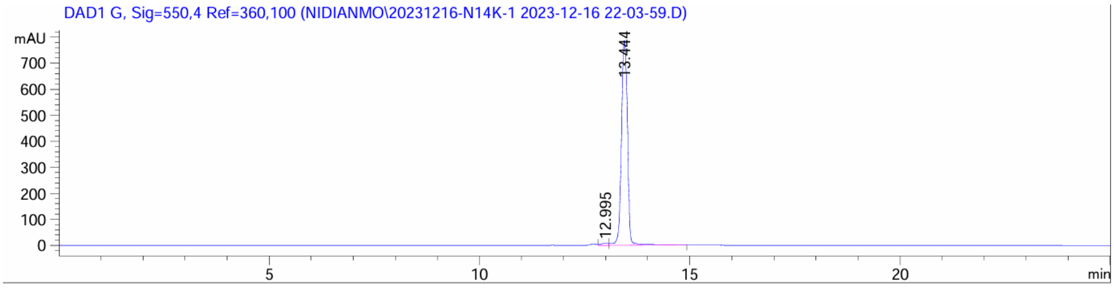

Signal 4: DAD1 G, Sig=550,4 Ref=360,100

Peak #	RetTime [min]	Type	Width [min]	Area [mAU*s]	Height [mAU]	Area %
1	12.995	VV	0.1804	88.82962	7.17768	1.1194
2	13.444	VB	0.1624	7846.33203	786.66351	98.8806
Totals :				7935.16165	793.84119	

Purity of the conjugate (**N14L**)

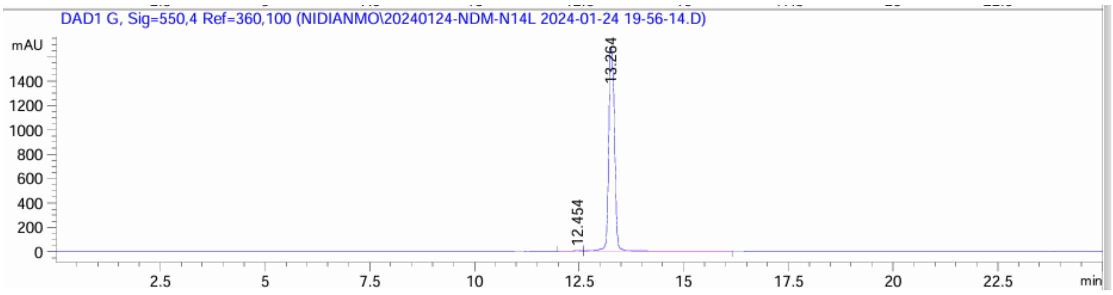

Signal 4: DAD1 G, Sig=550,4 Ref=360,100

Peak #	RetTime [min]	Type	Width [min]	Area [mAU*s]	Height [mAU]	Area %
1	12.454	BV	0.1806	120.73195	9.87774	0.7205
2	13.264	VB	0.1615	1.66360e4	1680.61011	99.2795
Totals :				1.67568e4	1690.48784	

Purity of the conjugate (**N14G-Fe**)

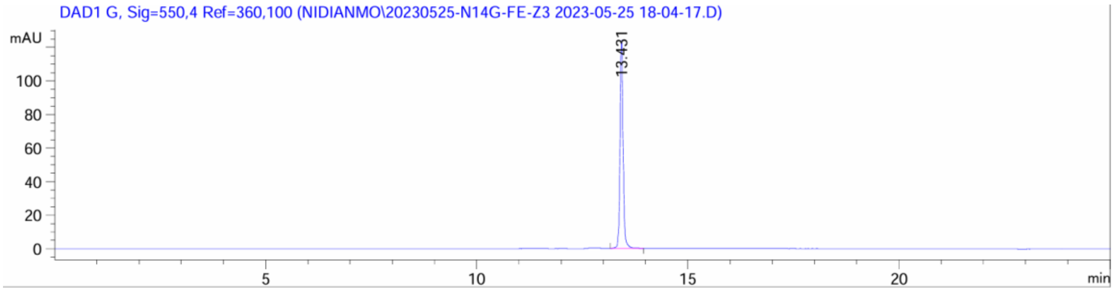

Signal 3: DAD1 G, Sig=550,4 Ref=360,100

Peak #	RetTime [min]	Type	Width [min]	Area [mAU*s]	Height [mAU]	Area %
1	13.431	BB	0.0771	628.28552	124.09647	100.0000
Totals :				628.28552	124.09647	

Purity of the conjugate (**N14I-Fe**)

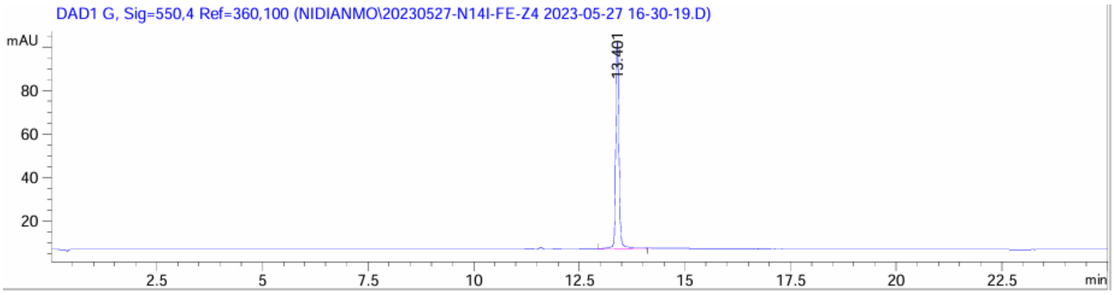

Signal 3: DAD1 G, Sig=550,4 Ref=360,100

Peak #	RetTime [min]	Type	Width [min]	Area [mAU*s]	Height [mAU]	Area %
1	13.401	BB	0.0791	499.08682	95.33665	100.0000
Totals :				499.08682	95.33665	

Purity of the conjugate (**N14G-Bz**)

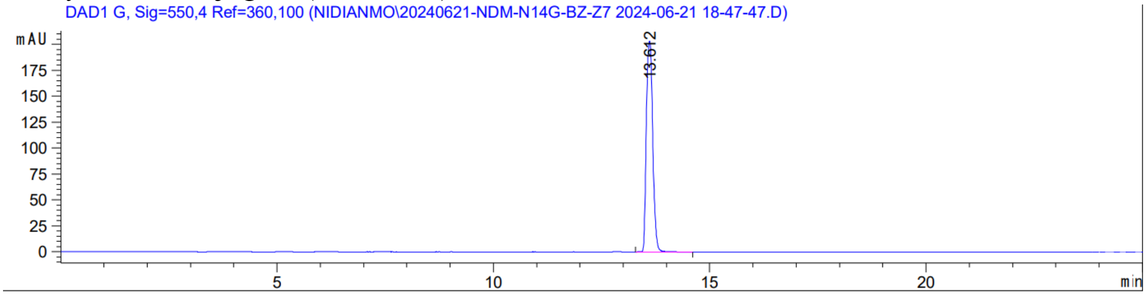

Signal 4: DAD1 G, Sig=550,4 Ref=360,100

Peak #	RetTime [min]	Type	Width [min]	Area [mAU*s]	Height [mAU]	Area %
1	13.612	BB	0.1639	2088.51733	203.23225	100.0000
Totals :				2088.51733	203.23225	

III. Experimental Procedures of Biology

3.1 Bacterial culture condition

M. bovis BCG [American Type Culture Collection (ATCC) 35734], *Mycobacterium smegmatis* (*M. smegmatis*, ATCC 700084), an IrtAB knockout strain ΔIrtAB *M. smegmatis* and complemented strain ΔIrtAB::ΔIrtAB *M. smegmatis* were purchased from Shanghai Gene Optimal Biotechnology Company. *Staphylococcus aureus* (*S. aureus*, ATCC 25923), *Escherichia coli* (*E. coli*, ATCC 700926), *Bacillus subtilis* (*B. subtilis*, ATCC 6633), and *Listeria monocytogenes* (*L. monocytogenes*, ATCC 19114) were kindly donated by Prof. Jingren Zhang.

M. bovis BCG and *M. smegmatis* were grown in iron-limiting Middlebrook 7H9 supplemented with 0.2% glycerol, 0.05% Tween-80, and 10% oleate-albumin-dextrose-catalase (OADC) enrichment. *E. coli*, *L. monocytogenes*, and *B. subtilis* were grown in LB medium, whereas *S. aureus* was grown in THY medium. All strains were grown in a shaking incubator at 180 rpm at 37 °C, except for *S. aureus*, which was grown in a static state.

3.2 Construction of knockout strain ΔIrtAB *M. smegmatis*

The left arm (914 bp) and right arm (1015 bp) were amplified using Phusion High Fidelity DNA Polymerase by PCR. The reaction conditions and systems followed the manufacturer's instructions. After amplifying the target DNA fragments, agarose gel electrophoresis was performed, and the target DNA fragments were recovered using a gel extraction kit. The recovered left and right arm DNA fragments were then digested with Van91I and SfiI restriction enzymes, respectively. The digested DNA fragments were purified using a quick recovery kit and stored for subsequent use. The p0004s plasmid was also digested with Van91I, and the target DNA fragment was recovered using a gel extraction kit. The DNA fragments obtained from the above steps were ligated using T4 DNA ligase. The ligation mixture was transformed into *E. coli* DH5α competent cells. Positive clones were screened and sequenced to verify the correct construction of the plasmid (p0004s-AES), which was then stored for further use.

The plasmids phAE159 and p0004s-AES were extracted using the Omega Plasmid Kit. Both plasmids were digested with PacI, and the target fragments were recovered using the Omega Quick Purification Kit. The two linearized plasmid fragments were ligated using T4 DNA ligase. The ligation mixture was transformed into *E. coli* HB101 competent cells using the packaging kit (EPICENTRE Biotechnologies, MP5120). Single colonies grown on hygromycin B (150 μg/mL) plates were picked and inoculated into liquid media containing hygromycin B (150 μg/mL) and cultured at 37 °C. Plasmids were extracted, and the presence of the target insert was confirmed by digestion with PacI. The resulting plasmid, phAE159-AES, was stored for further use.

A fresh single colony of *M. smegmatis* mc^{2}155 was inoculated into 5 mL of 7H9 liquid medium and cultured at 37 °C with shaking until the logarithmic growth phase (OD_{600} = 0.5–1.0). The culture was then diluted 1:100 into 100 mL of 7H9 medium and incubated overnight at 37 °C until the OD_{600} reached approximately 0.6. The culture was placed on ice for 0.5–1 hour, then centrifuged at 5000 rpm for 10 minutes at 4 °C

to collect the cells. The cell pellet was washed at least twice with pre-chilled 10% sterile glycerol and resuspended in an appropriate volume of pre-chilled 10% glycerol. The suspension was aliquoted into 200 μL portions and stored at -80 °C for future use.
An appropriate amount of the positive phAE159-AES plasmid was mixed with *M. smegmatis* mc^{2}155 electrocompetent cells. The mixture was transferred to a 2 mm cuvette and electroporated using a Bio-Rad electroporator with the following parameters: 2.5 kV voltage, 1000 Ω resistance, and 25 μF capacitance. After electroporation, 7H9 medium was added, and the cells were incubated overnight at 37 °C. The culture was then mixed with an appropriate amount of top agar and poured onto 7H10 solid plates. The plates were incubated at 30 °C for 2–3 days to screen for phage plaques. Plaques containing phages were picked and resuspended in MP buffer, incubated overnight at 4 °C. The phage-containing liquid was then mixed with freshly grown *M. smegmatis* mc^{2}155 cells and an appropriate amount of top agar, and the mixture was poured onto plates. The plates were incubated at 30 °C for 2–3 days. MP buffer was added to the plates containing phage plaques and incubated overnight at 4 °C. The phage-containing liquid was collected and filtered through a 0.22 μm sterile filter to obtain high-titer phages, which were stored at 4 °C.
High-titer phage lysate was mixed with *mycobacteria* grown to the logarithmic phase (previously washed with MP buffer) and incubated overnight at 37 °C. The culture was then centrifuged to remove the supernatant, and an appropriate amount of 7H9 liquid medium was added. The culture was incubated overnight at 37 °C, and the cells were collected by centrifugation and plated on 7H10 plates supplemented with OADC and hygromycin B (150 μg/mL). The plates were incubated at 37 °C for 3 days. Several clones were picked and inoculated into 7H9 medium containing hygromycin B at a final concentration of 150 μg/mL, respectively. After culturing at 37 °C for 3 days, the bacterial cells were collected, and genomic DNA was extracted for PCR and sequencing validation.

3.3 Construction of complemented strain ΔIrtAB::ΔIrtAB *M. smegmatis*
The full-length target gene was amplified using Phusion High Fidelity DNA Polymerase by PCR. The reaction conditions and systems followed the manufacturer's instructions. After amplifying the target DNA fragment, agarose gel electrophoresis was performed, and the target DNA fragment was recovered using the Omega Gel Extraction and Purification Kit. The specific steps for gel recovery were performed according to the kit's manual. The recovered DNA fragment was then digested with HindIII and EcoRI restriction enzymes. The digested DNA fragment was purified using a quick recovery kit and stored for subsequent use.
The pMV361 plasmid was extracted using the Tiangen Plasmid Extraction Kit and digested with HindIII and EcoRI restriction enzymes. The digested plasmid was purified using a gel extraction kit. The purified DNA fragments obtained from the above steps were ligated using a seamless cloning method. The ligation mixture was transformed into *E. coli* DH5α competent cells, following the methods described in the Molecular Cloning Laboratory Manual. Positive clones were screened and sequenced to verify the correctness of the plasmid. The verified positive plasmid was stored for

further use.

A fresh single colony of the *M. smegmatis* mc^{2}155 knockout strain was inoculated into 5 mL of 7H9 liquid medium containing hygromycin B at a final concentration of 150 μg/mL. The culture was incubated at 37 °C with shaking until the logarithmic growth phase (OD_{600} = 0.5–1.0). The culture was then diluted 1:100 into 100 mL of 7H9 medium and incubated overnight at 37 °C until the OD_{600} reached approximately 0.6. The culture was placed on ice for 0.5–1 hour, then centrifuged at 5000 rpm for 10 minutes at 4 °C to collect the cells. The cell pellet was washed at least twice with pre-chilled 10% sterile glycerol and resuspended in an appropriate volume of pre-chilled 10% glycerol. The suspension was aliquoted into 200 μL portions and stored at -80 °C for future use.

An appropriate amount of the positive plasmid was mixed with the *M. smegmatis* mc^{2}155 electrocompetent cells. The mixture was transferred to a 2 mm cuvette and electroporated using a Bio-Rad electroporator with the following parameters: 2.5 kV voltage, 1000 Ω resistance, and 25 μF capacitance. After electroporation, 7H9 medium was added, and the cells were incubated overnight at 37 °C. The culture was then centrifuged, and the supernatant was discarded. The cell pellet was resuspended in approximately 100 μL of the remaining liquid medium. The cell suspension was spread on two 7H10 solid plates containing hygromycin B at a final concentration of 150 μg/mL and kanamycin at a final concentration of 30 μg/mL. The plates were incubated at 37 °C for 4–5 days. Single colonies were picked and cultured in 7H9 medium containing hygromycin B at a final concentration of 150 μg/mL and kanamycin at a final concentration of 30 μg/mL. The cultures were incubated at 37 °C for 3 days. The bacterial cells were collected, and genomic DNA was extracted for PCR and sequencing validation.

3.4 Fluorescence fold change of bacteria analyzed by flow cytometry

M. smegmatis and *M. bovis* BCG were separately cultured in iron-limiting 7H9 medium until they reached the logarithmic growth phase. After incubation with probes, bacteria were analyzed by flow cytometry using a BD LSRFortessa equipped with a four-laser, 14-color analyzer from the Tsinghua University Instrument Sharing Platform. Bacteria treated with **N14A**, **N14B**, **Cy3-NO_2-Tre**, **N14E**, **N14F**, **N14H**, **N14J**, **N14G**, **N14G-Fe**, **N14I**, **N14I-Fe**, **N14K**, **N14L** and **N14G-Bz** were excited using a 561 nm violet laser and filtered through a 586/15 nm laser for the PE channel. Bacteria treated with **N14C** were excited using a 640 nm violet laser and filtered with a 670 nm laser for the APC channel. Bacteria treated with **17a-Tre** were excited using a 405 nm violet laser and filtered with a 450/50 nm laser for the pacific blue channel. Bacteria treated with **DMN-Tre** were excited using a 405 nm violet laser and filtered with a 488 nm laser for the FITC channel. Flow cytometry data analysis was performed using FlowJo v10 software. The mean fluorescence intensity (MFI) was recorded and fluorescence fold change (FFC) was calculated by dividing the MFI of the bacteria treated with probes by that of the bacteria treated without probes. A paired sample *t*-test was also performed for the FFC to determine significant differences between each experimental group. Figures were prepared using GraphPad Prism v8.0.

3.5 Heat-killing experiment

M. smegmatis was cultured in iron-limiting 7H9 medium until they reached the logarithmic growth phase. Before incubation with probes, it was heated at different temperatures (95 °C, 75 °C, and 55 °C, respectively) in oil bath for 30 min. Then samples were analyzed by flow cytometry.

3.6 Drug treating experiment

M. smegmatis was cultured in iron-limiting 7H9 medium until they reached the logarithmic growth phase. Before incubation with probes, it was treated with BTZ043 in different concentrations (50 μM, 100 μM, and 250 μM, respectively) for 5 h. Then samples were analyzed by flow cytometry.

3.7 Fluorescence microscopy

For the experiment of selective labeling, green fluorescent protein (GFP)-expressing *M. smegmatis*, *E. coli*, *S. aureus*, *B. subtilis* and *L. monocytogenes* were cultured to logarithmic growth phase and mixed well. Mixed bacteria samples were incubated with 0.01 μM of **N14G** for 1 h. The samples were centrifuged (3000 g for 15 min) and resuspended in phosphate buffered saline (PBS). A DeltaVision wide-field fluorescence microscope was used for specific labeling experiments. Images were collected in the differential interference contrast (DIC), GFP, or red fluorescence channels. "Merge" represents merged images from all channels.

For the experiment of imaging of *M. smegmatis*, *M. smegmatis* was incubated with 0.01 μM of **N14G** or **N14G-Fe** for some time. The bacteria treated with **N14G** were centrifuged (8000 g for 5 min) and resuspended in PBS before imaging. The bacteria incubated with **N14G-Fe** were imaged directly. A Super-resolution Nikon SIM Microscope was used to image the fluorescence distribution of **N14G** or **N14G-Fe** within *M. smegmatis*.

For the experiment of imaging of *M. bovis* BCG and H37Rv*M. bovis* BCG and H37Rv were incubated with 0.1 μM of **N14G** or **N14G-Fe** for 10 min. The bacteria treated with **N14G** were centrifuged (8000 g for 5 min) and resuspended in PBS before imaging by an Olympus FV1000 confocal microscope. The bacteria incubated with **N14G-Fe** were imaged directly.

3.8 Photophysical properties

The excitation and emission spectra of probes were recorded on a HORIBA UV-800C Fluorometer. The excitation and emission spectra were measured at the maximum emission and excitation wavelengths in ddH_2O, respectively.

3.9 UV-Visible absorption spectroscopy

P10C, **(*S*)-P10C** and **cMbT** of 10 mM stock solution were prepared in DMSO and used for further experiments. Measurements were conducted in EtOH at r.t. The Fe^{3+} ion was titrated with increasing concentration from 10 μM to 150 μM. The change in absorbance spectra of MbT/cMbT was recorded and analyzed for metal binding. The absorbance

values were fitted using the Benesi-Hildebrand equation according to published literature.[7]

3.10 Detection of bacteria in culture

Cultures were grown until reaching an OD_{600} of 0.6–0.8 and then were centrifuged and discard the supernatant, followed by diluting in PBS to reach the desired cell concentration (1.1×10^9 CFU/mL, 1.1×10^8 CFU/mL, 1.1×10^7 CFU/mL, 1.1×10^6 CFU/mL, 1.1×10^5 CFU/mL, and 1.1×10^4 CFU/mL, respectively). Each of bacterial samples was added to a centrifuge tube followed by incubation with **N14G** (1 μM). PBS was added to a centrifuge tube followed by incubation with **N14G** (1 μM) as control group. All samples were incubated in 37 ℃ for 1 h, then measured by fluorescent microscope. Figure was prepared using GraphPad Prism v8.0.

3.11 Staining of sputum samples with N14G, N14G-Fe and AO reagent

Sputum samples were collected from patients with TB and digested with *N*-acetyl-l-cysteine/sodium hydroxide (NALC/NaOH) buffer, neutralized, and centrifuged (3000 g for 15 min) according to the standard protocol. The resulting sediments were resuspended in 2.0 mL of iron-limiting 7H9 culture medium and then incubated at 37 ℃ for 24 h. For patients 1–4, **N14G** was added to the culture medium and incubated for 24 h. For patients 5–11, after incubating the sputum samples in the iron-limiting 7H9 culture medium for 24 h, further supplement with **N14G** (0.1 μM) or **N14G-Fe** (0.1 μM), and then continue incubation at 37 °C for 10 min. Samples incubated with **N14G-Fe** were smeared onto microscope slides directly, while those treated with **N14G** were centrifugated (3000 g for 15 min), washed with PBS and then smeared onto microscope slides. All the sputum samples were heated at 70 ℃ for 30 s, de-stained with 3% HCl-EtOH solution for 3 min and imaged using an Olympus FV1000 confocal microscope. Images were collected in the DIC for the bright filed and red fluorescence channel for **N14G** and **N14G-Fe**. Excitation at 530/40 nm and emission at 605/55 nm were used for **N14G** and **N14G-Fe**.

The staining procedures with AO referred to the standard operation of the reagent kit.

All sputum samples used in this study were provided by Beijing Chest Hospital. Ethical application for this study was approved by the Beijing Chest Hospital affiliated with the Capital Medical University.

3.12 Statistical analysis

All results are mean ± SD of the measurements from triplicate wells. All data were plotted and analyzed using GraphPad Prism 8.3.0. Significant differences were determined using *t*-test. ns $P > 0.05$, $*P < 0.05$, $**P < 0.01$, $***P < 0.001$ and $****P < 0.0001$.

IV. CD Spectral Analysis and Molecular Docking

4.1 CD spectral analysis

All the CD spectra were recorded on a Jasco J815 spectrometer (Jasco Inc., Japan) and a 1 mm quartz cell. The conditions of measurement were as follows: scanning speed, 200 nm/min; bandwidth, 1 nm; and 3 accumulations.

4.2 Molecular docking studies

The crystal structure of siderophore interaction domain (SID) (PDB ID: 6TEK) were obtained from RCSB Protein Data Bank (PDB) (https://www.rcsb.org). The ligands were prepared by using energy minimization, and the protein structure was prepared by using QuickPrep Wizard (Chemical Computing Group ULC, 910-1010 Sherbrooke St. W., Montreal, QC H3A 2R7, Canada, 2024). The oriented compounds then saved as a new data base by MDB format. The ligands were docked to the prepared protein using the binding pocket defined by residues R55, Q237, and R244. Thirty poses were generated for each docking process, which were regulated by the London dG-scoring function, and adjusted twice using the triangle Matcher method. The best poses obtained by comparing the interaction with key residues were output among the docking poses. The results are presented using Pymol software.

V. ^{1}H,^{13}C and ^{19}F NMR Spectra

^{1}H NMR spectra of **A1**

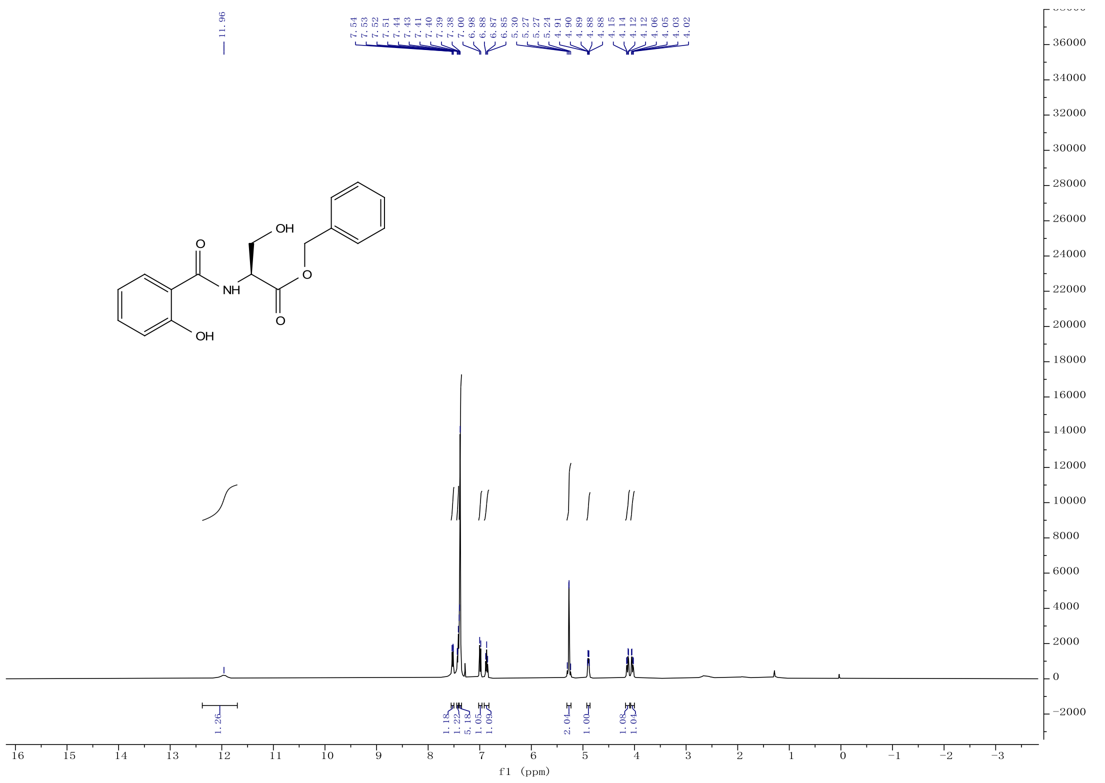

^{13}C NMR spectra of **A1**

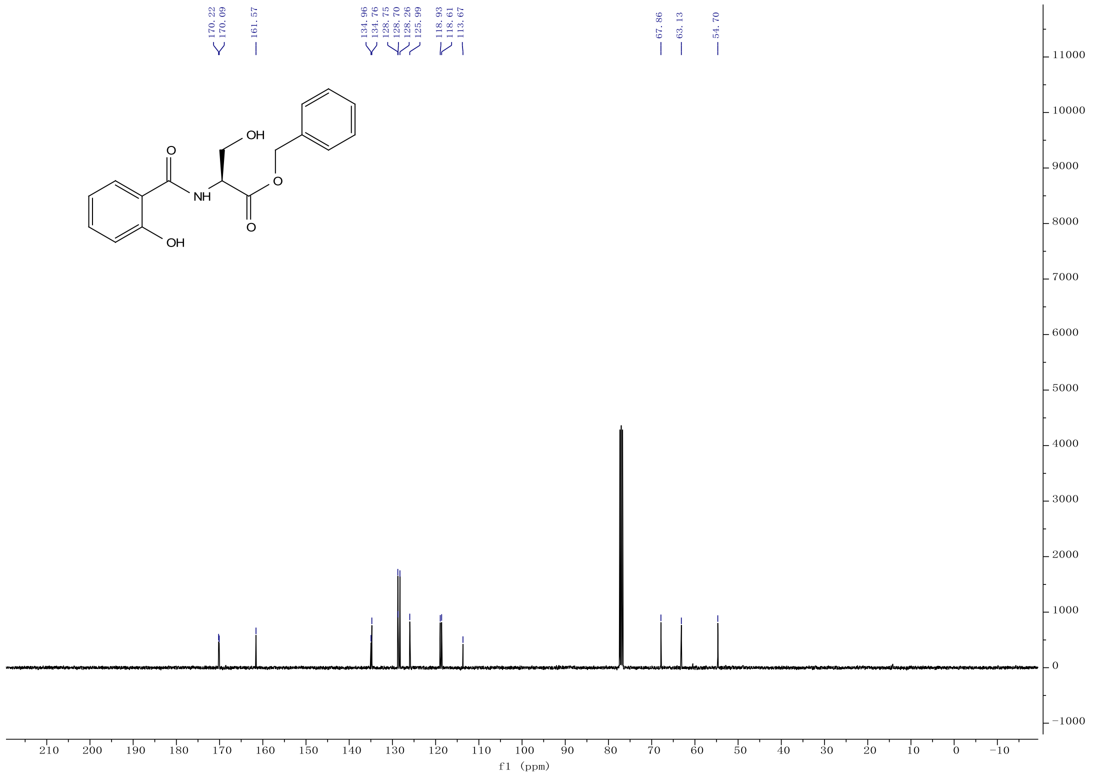

^{1}H NMR spectra of **A2**

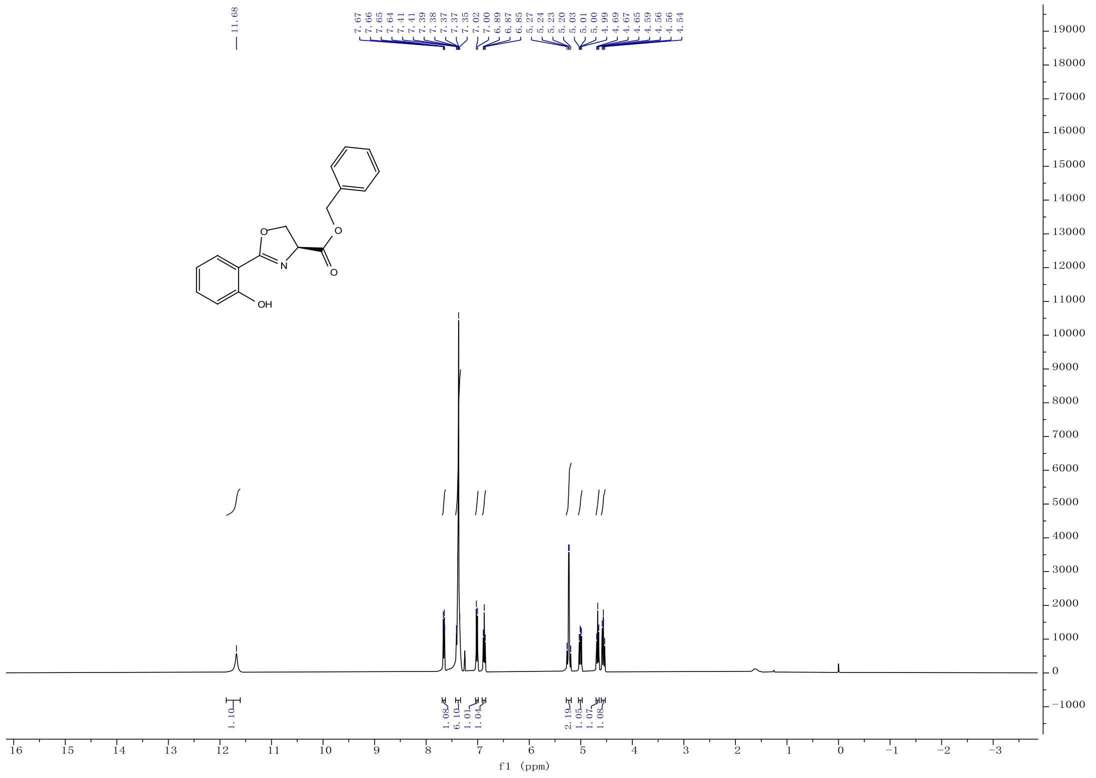

^{13}C NMR spectra of **A2**

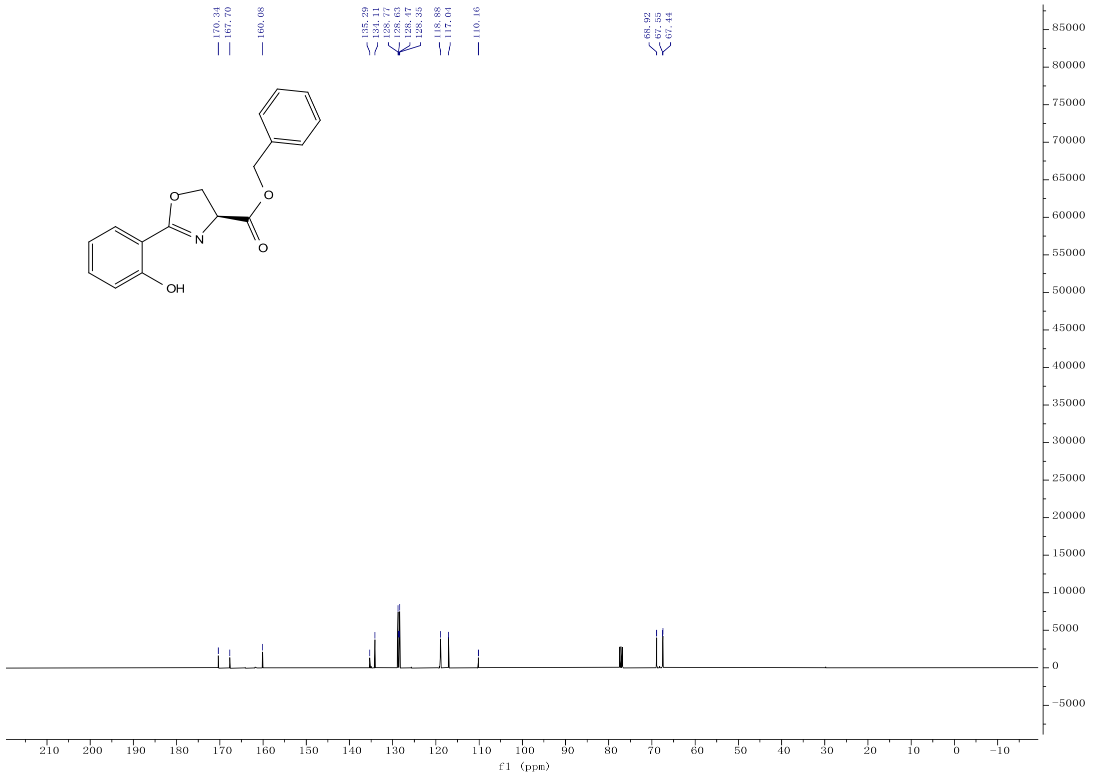

^{1}H NMR spectra of **A10**

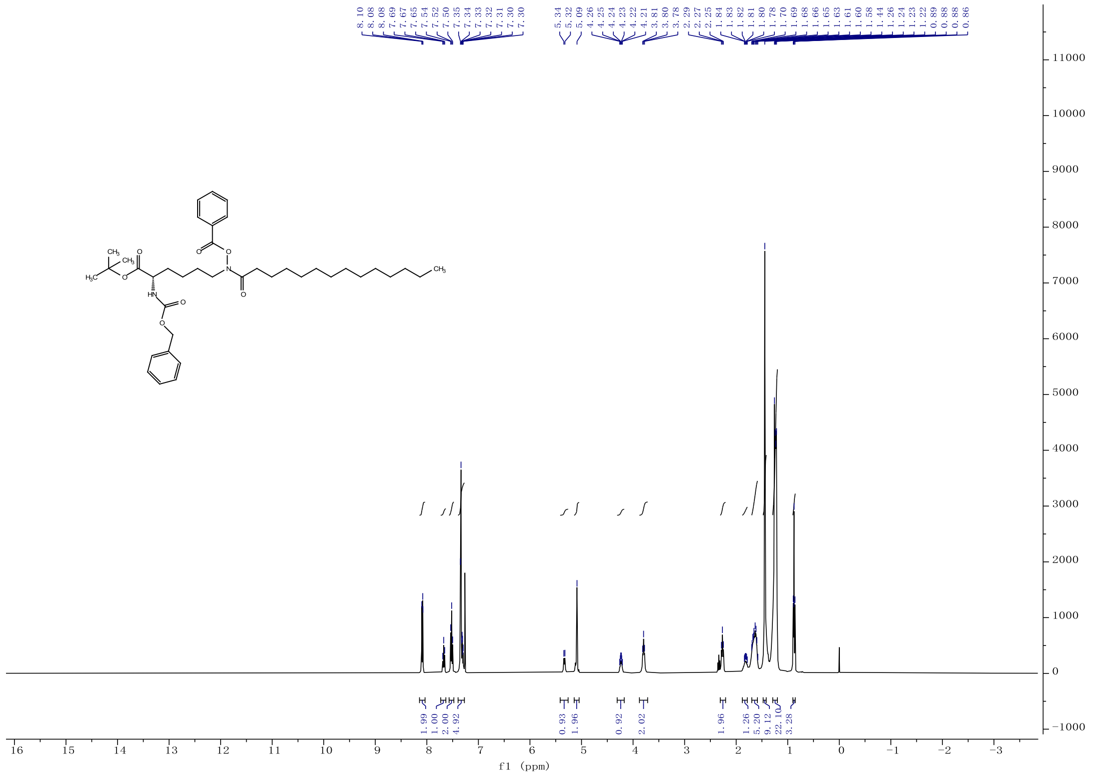

^{13}C NMR spectra of **A10**

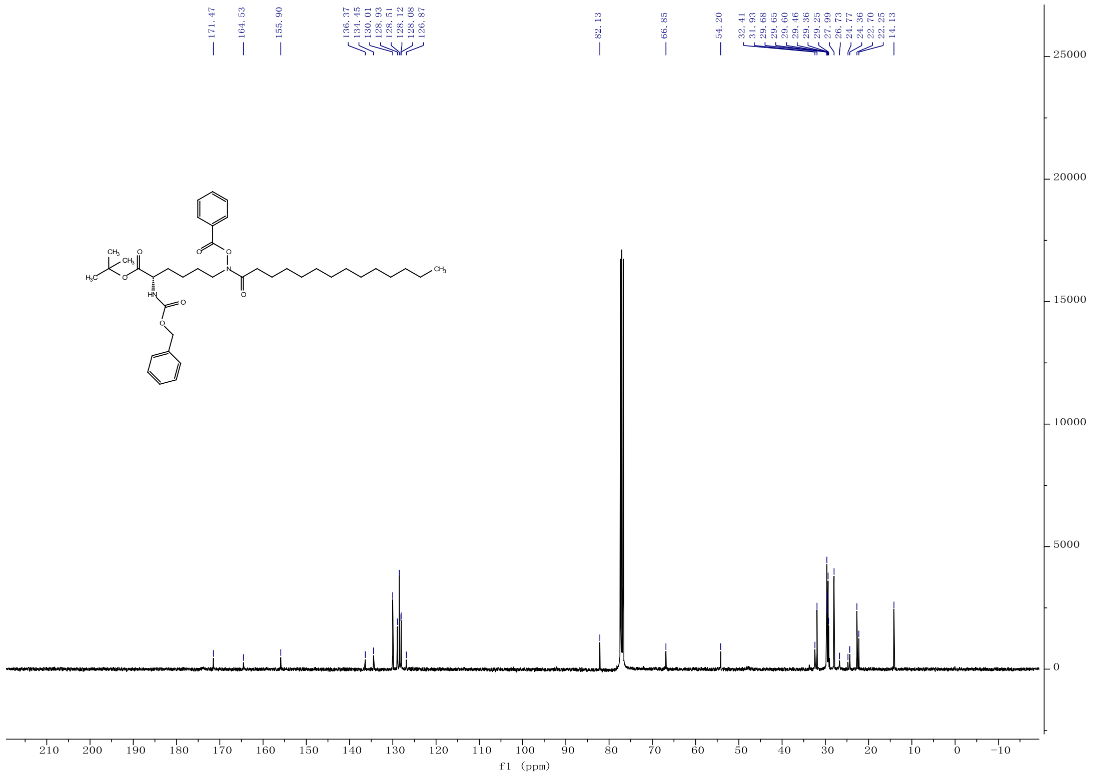

^{1}H NMR spectra of **A12**

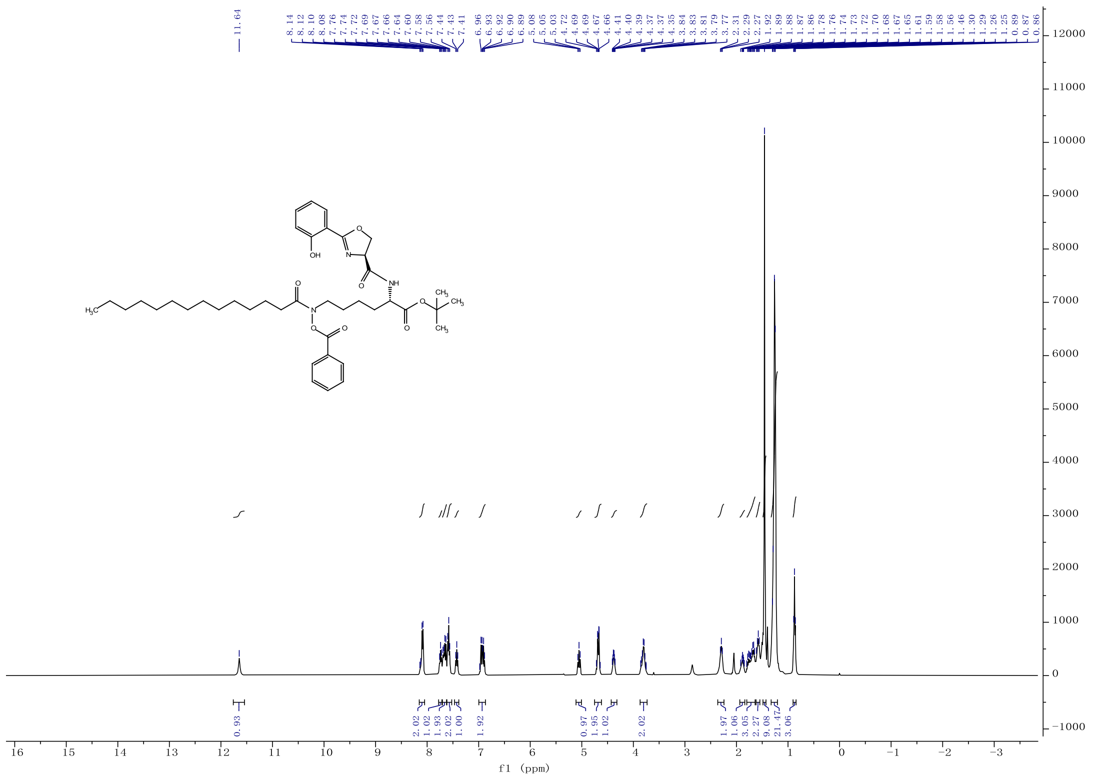

^{13}C NMR spectra of **A12**

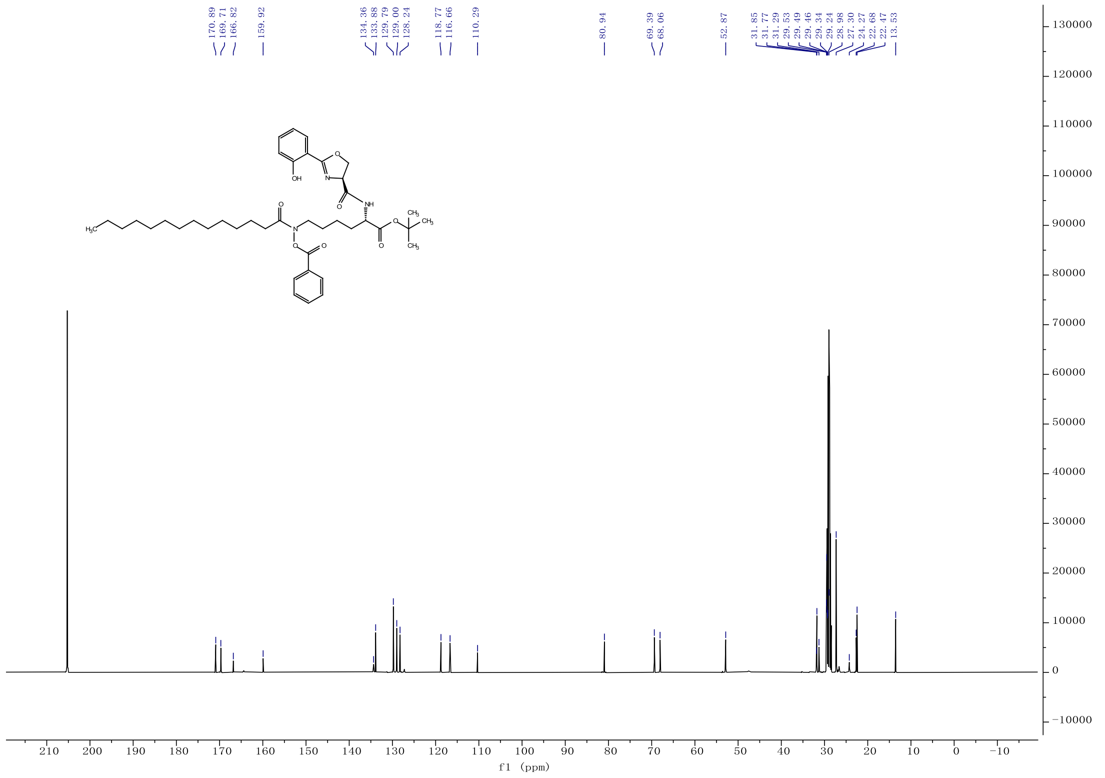

^{1}H NMR spectra of **A13**

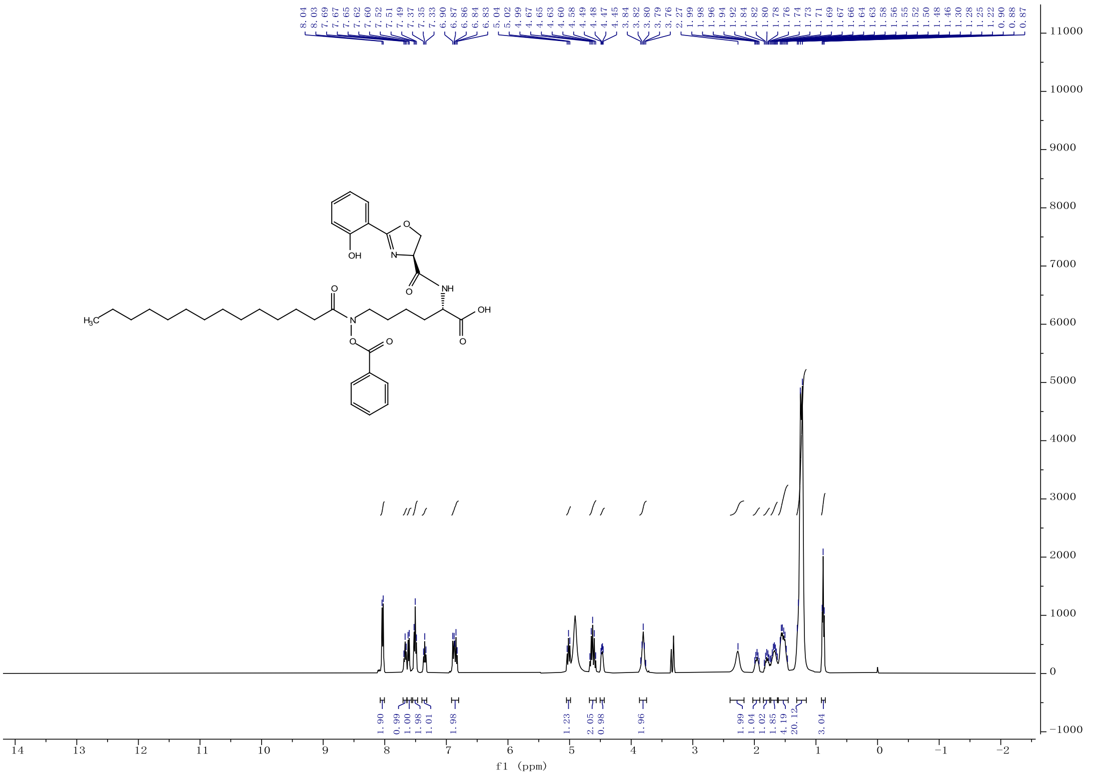

^{13}C NMR spectra of **A13**

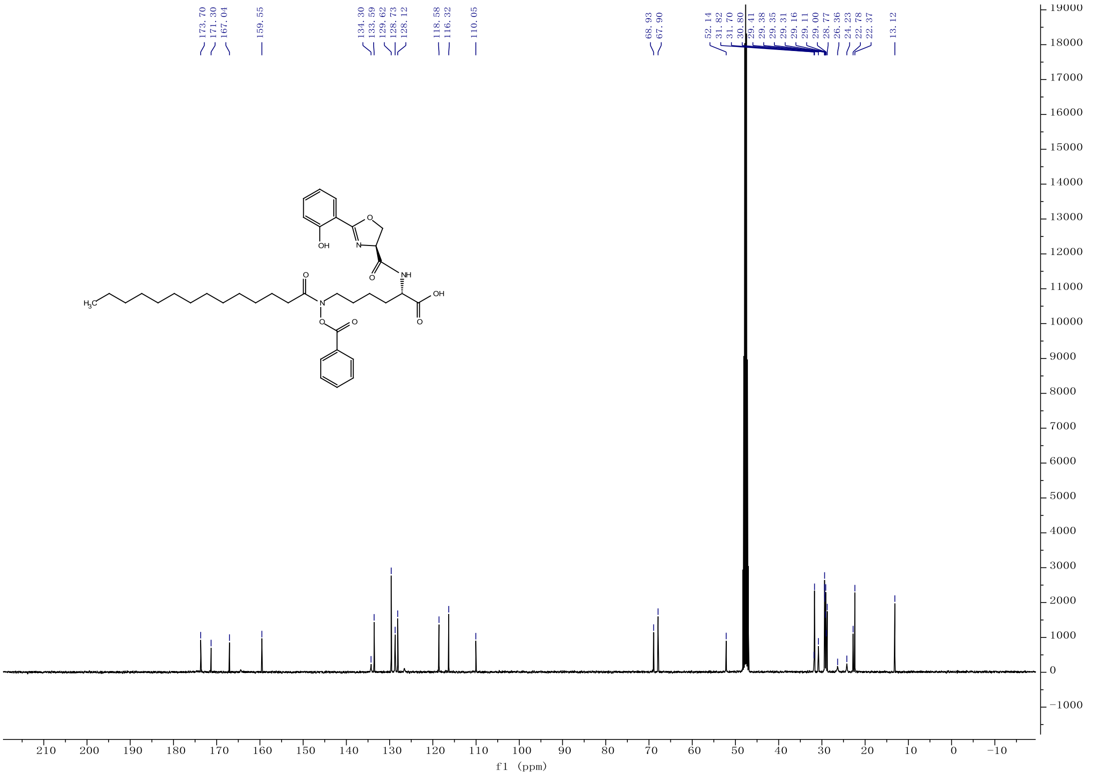

^{1}H NMR spectra of **MIS-1**

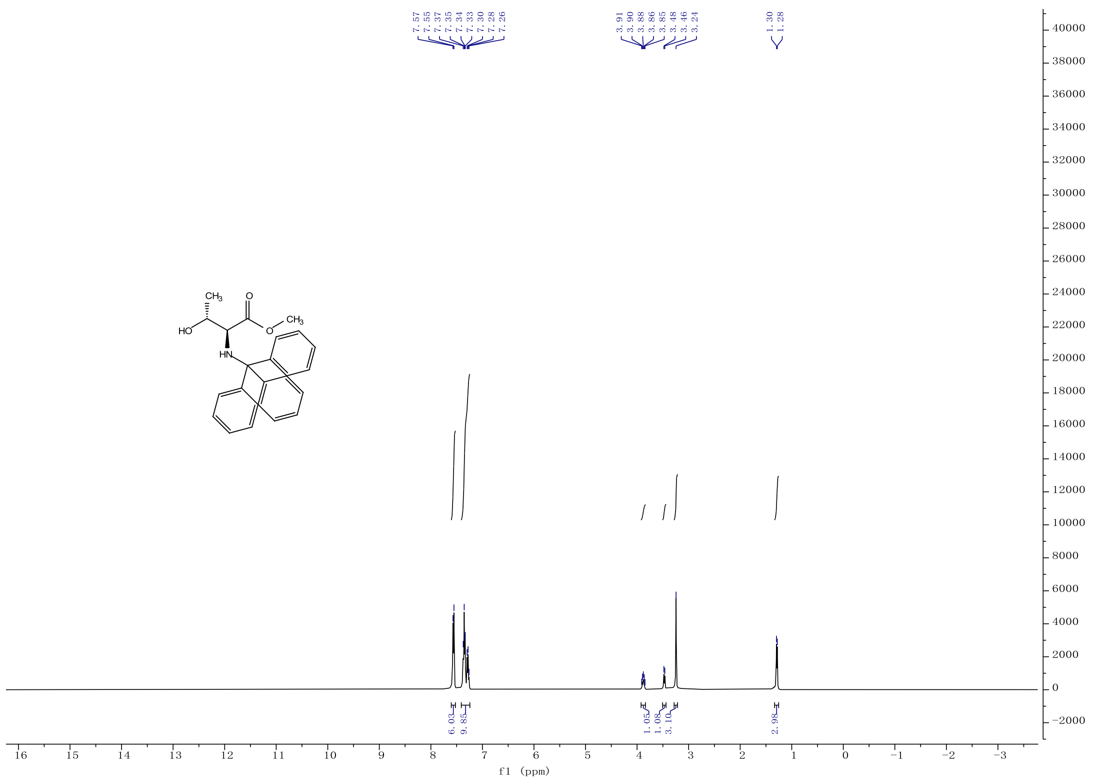

^{13}C NMR spectra of **MIS-1**

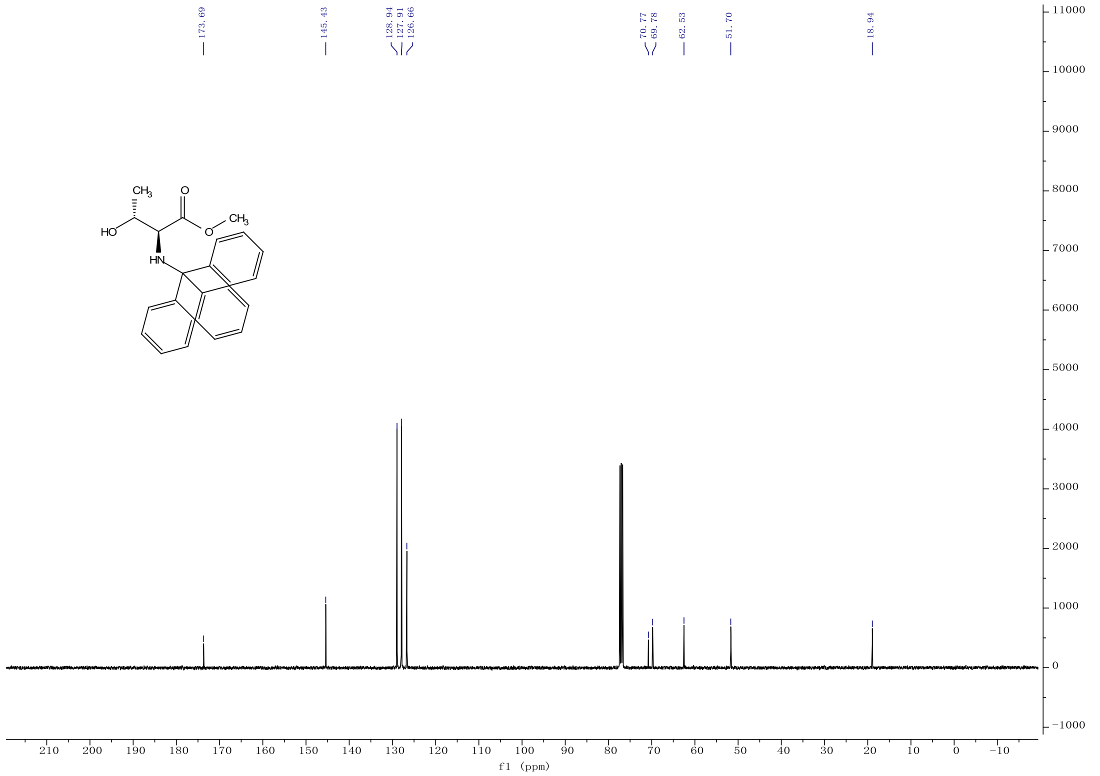

^{1}H NMR spectra of **MIS-2**

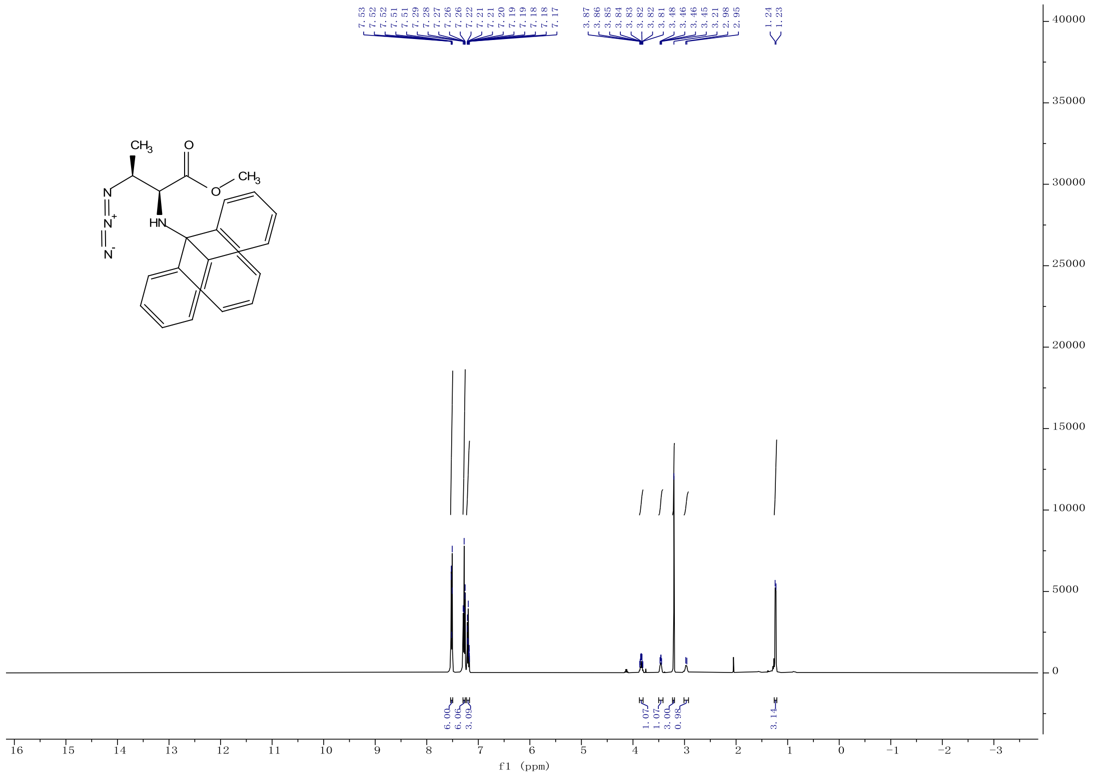

^{13}C NMR spectra of **MIS-2**

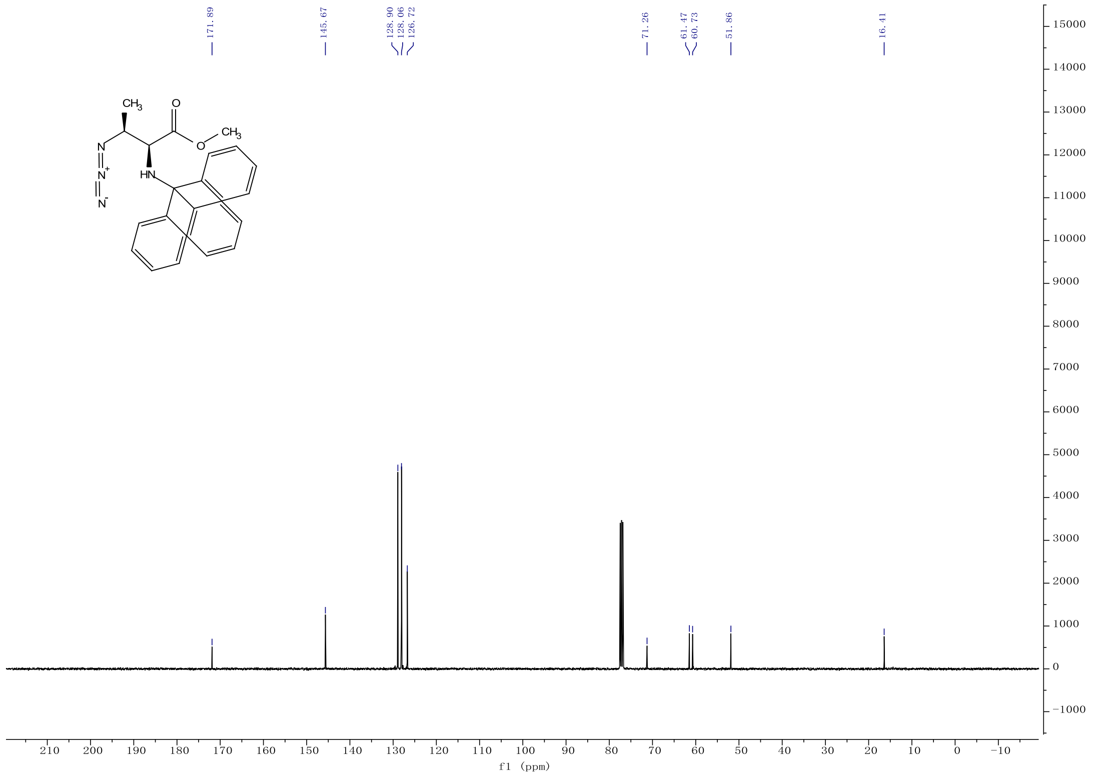

^{1}H NMR spectra of **MIS-4**

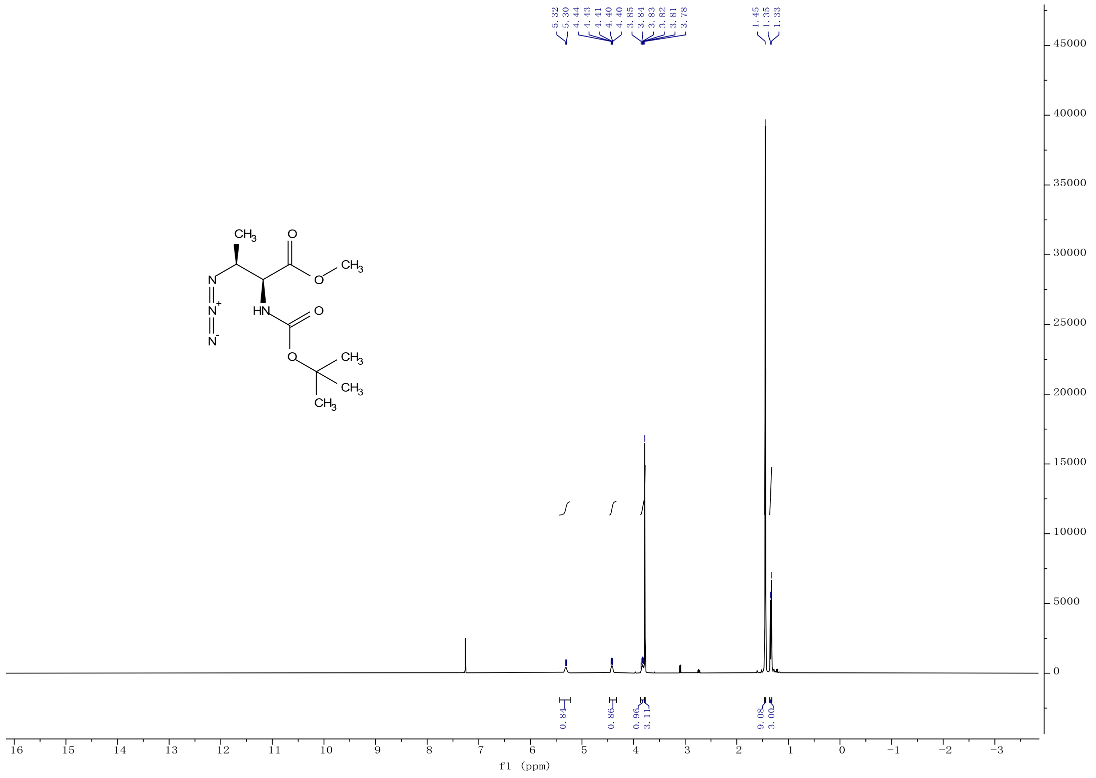

^{13}C NMR spectra of **MIS-4**

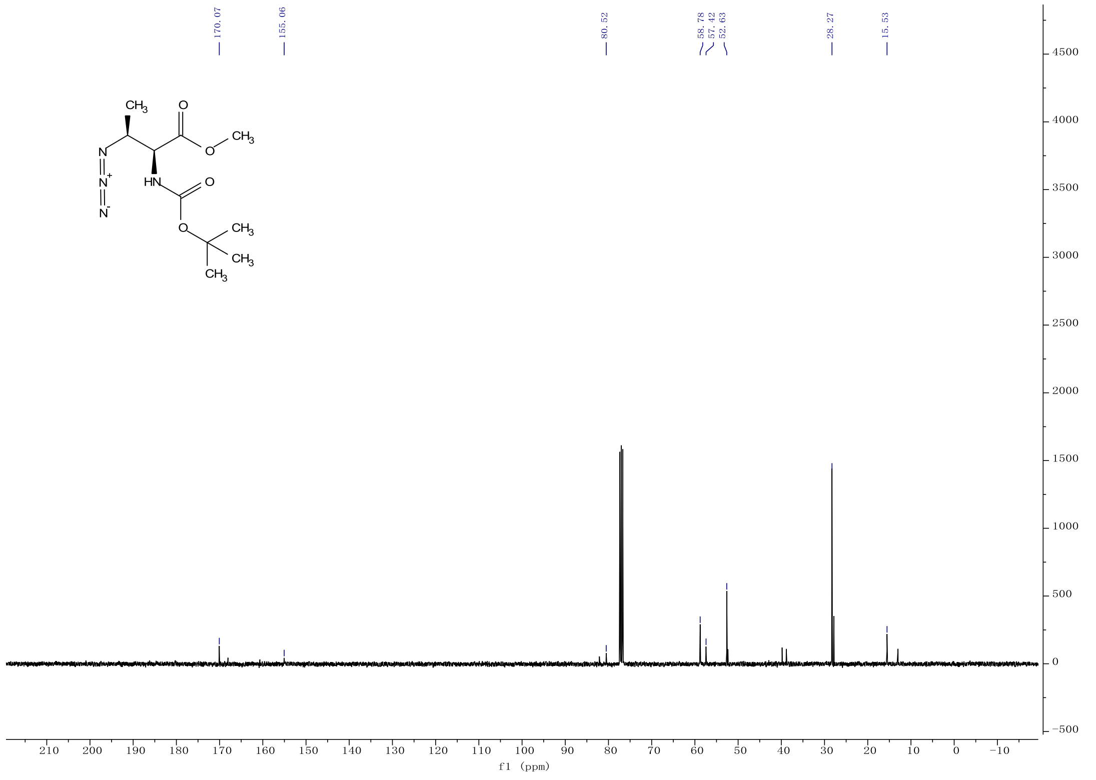

^{1}H NMR spectra of **MIS-6**

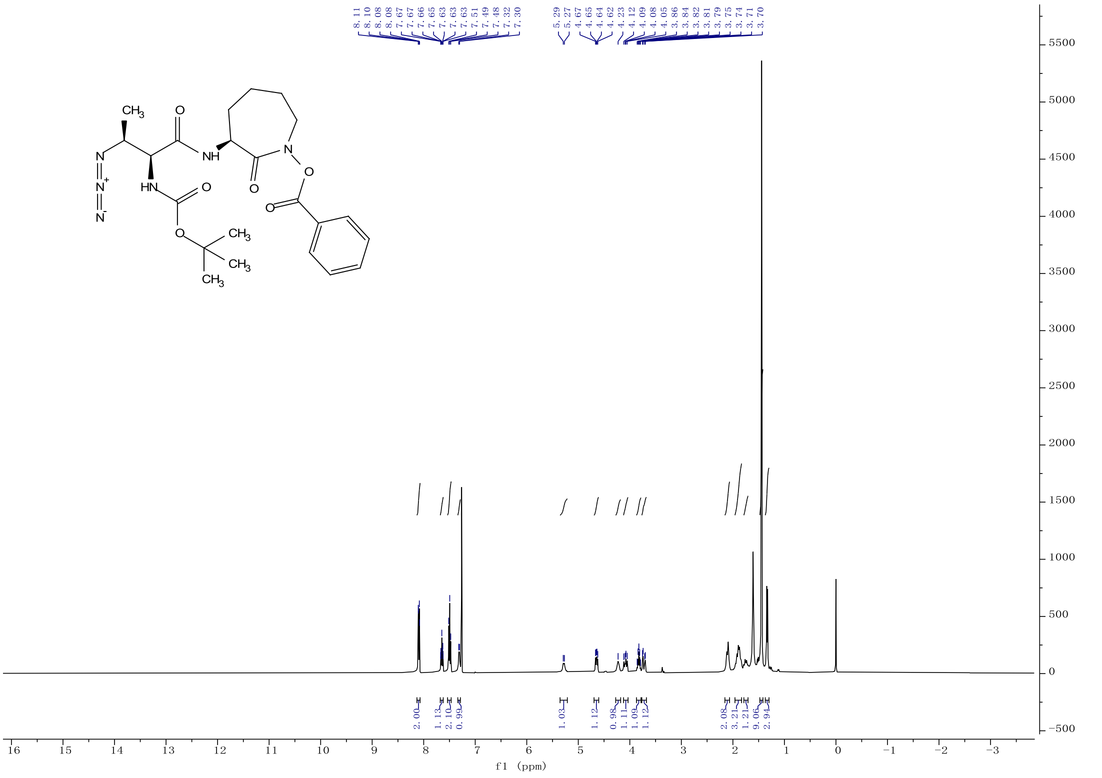

^{13}C NMR spectra of **MIS-6**

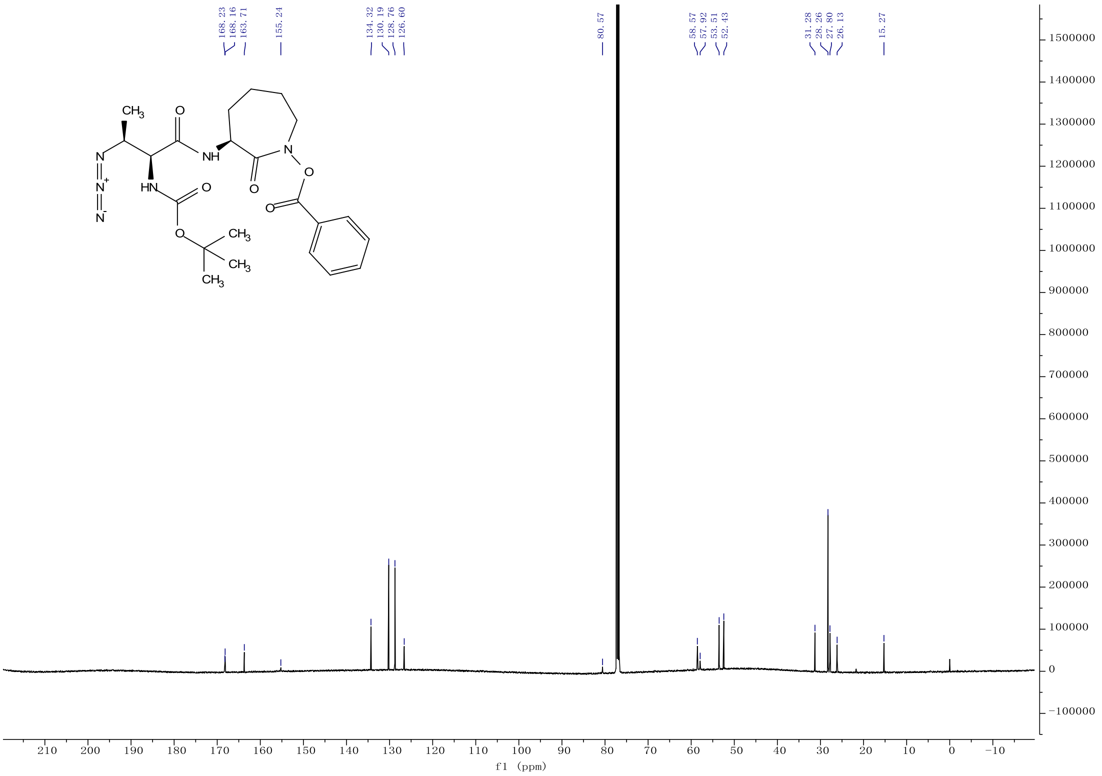

^{1}H NMR spectra of **MIS-8**

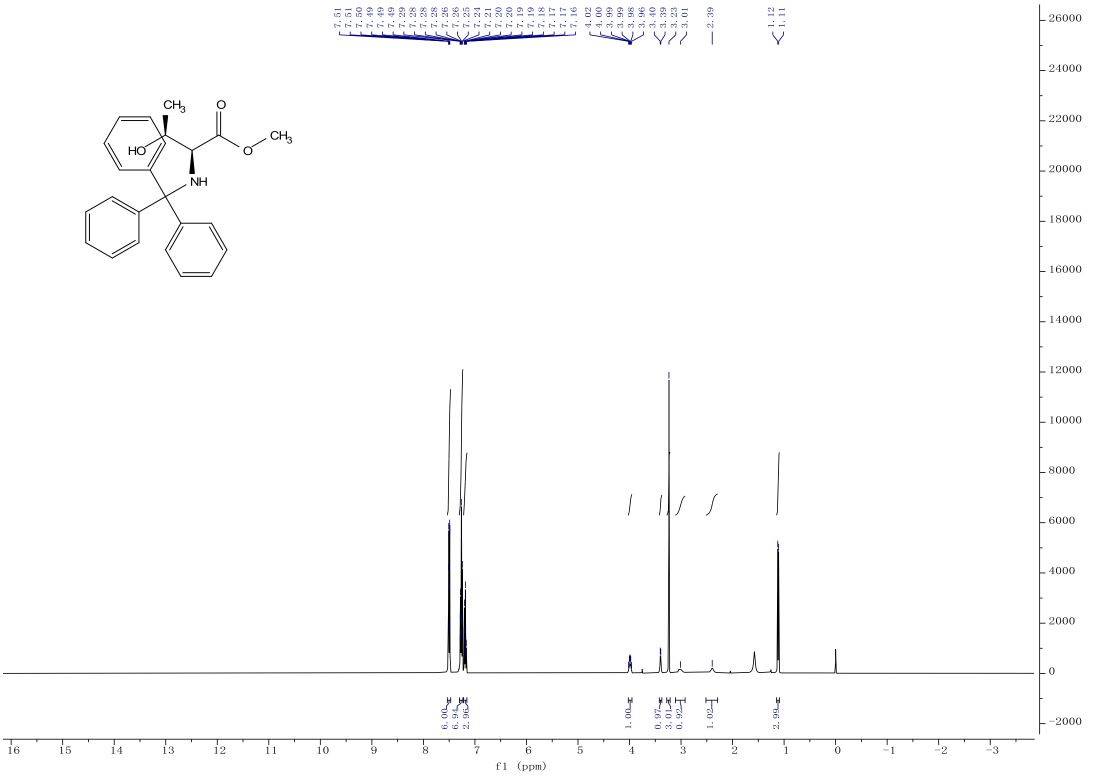

^{13}C NMR spectra of **MIS-8**

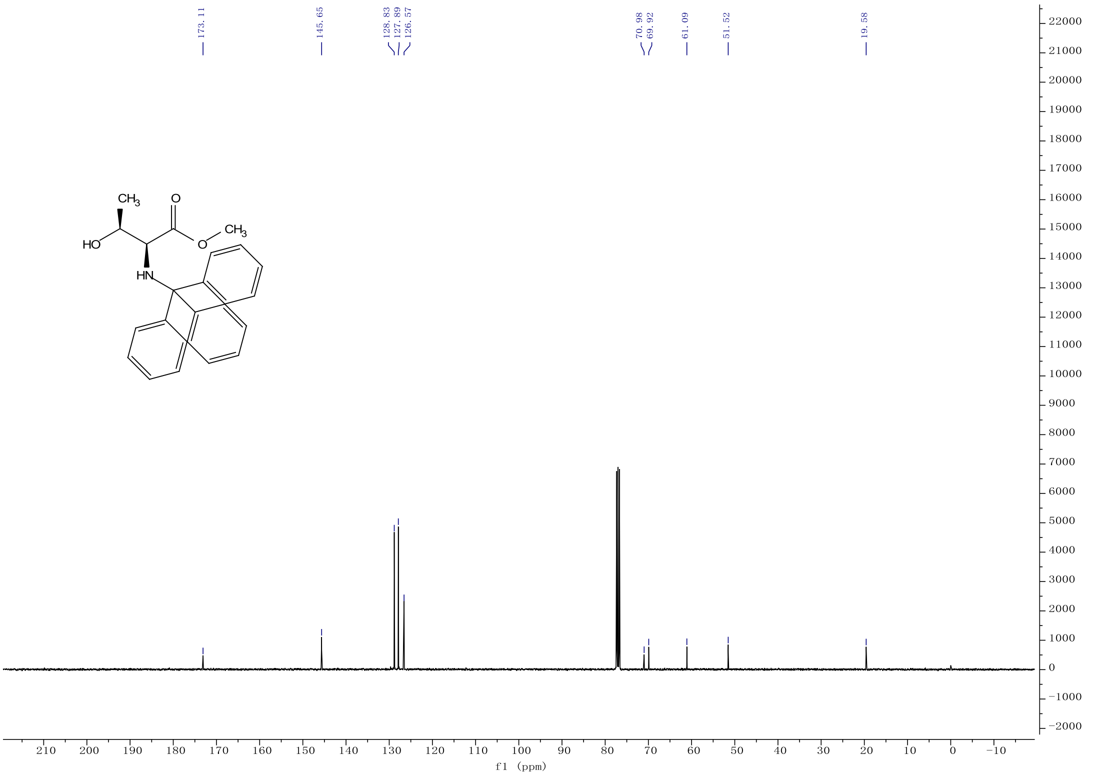

^{1}H NMR spectra of **MIS-9**

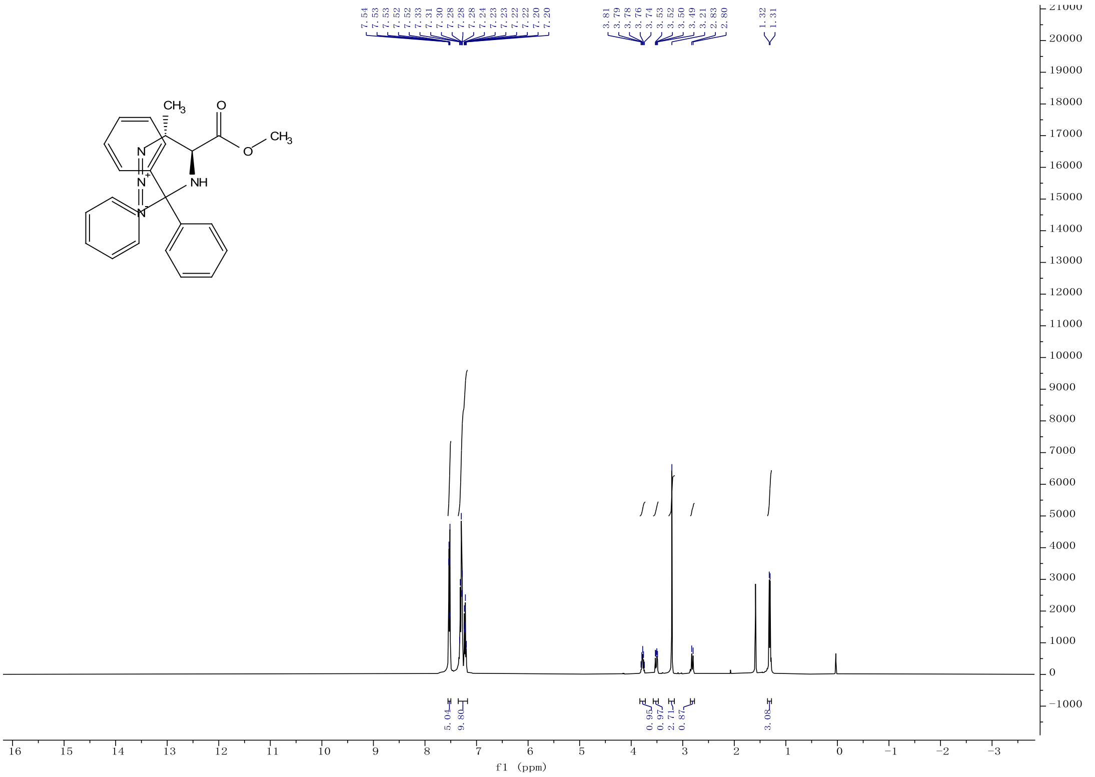

^{13}C NMR spectra of **MIS-9**

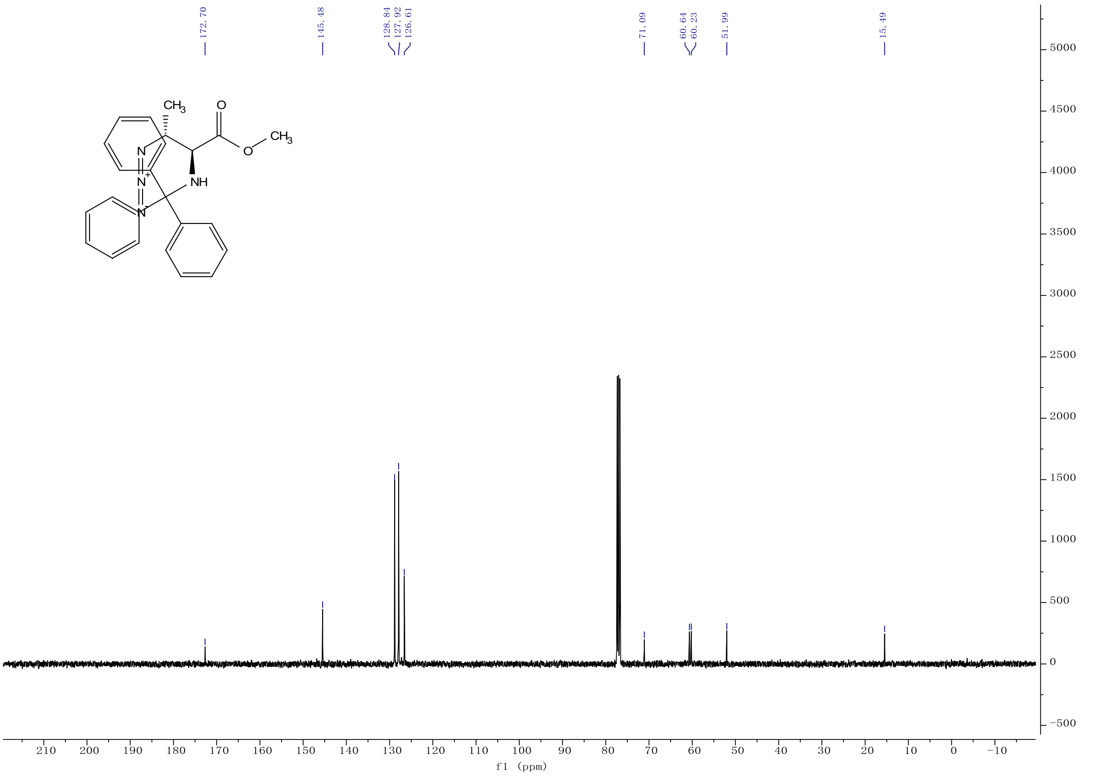

^{1}H NMR spectra of **MIS-11**

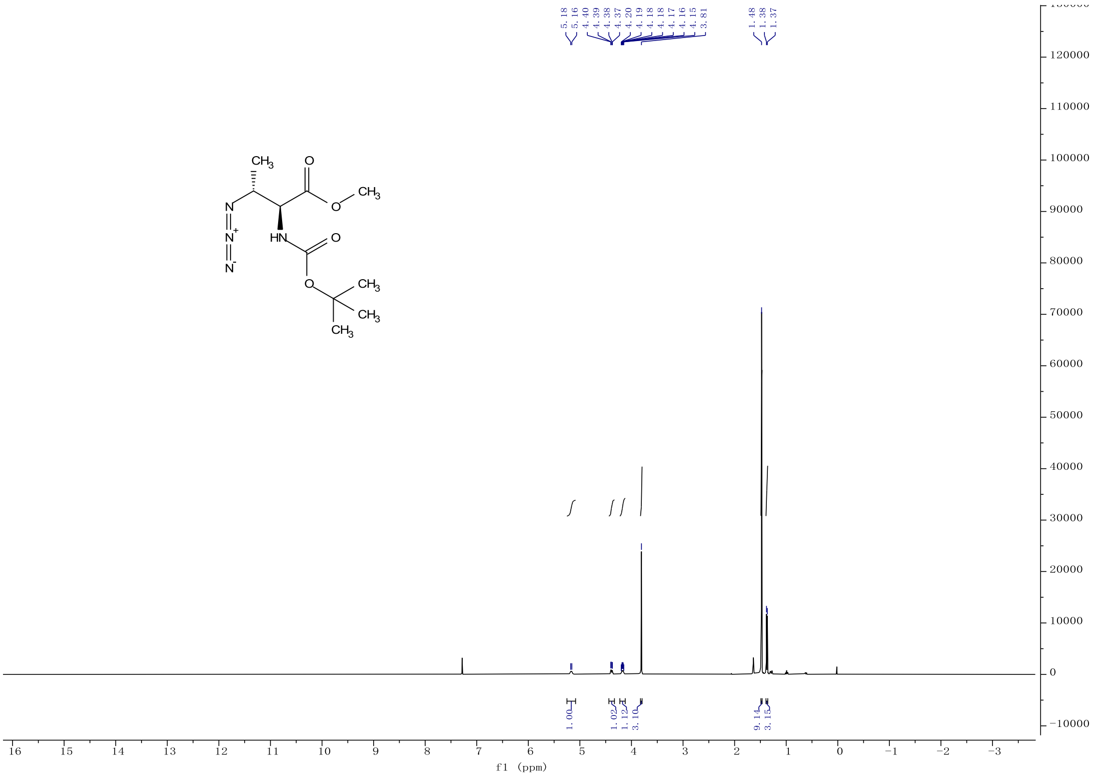

^{13}C NMR spectra of **MIS-11**

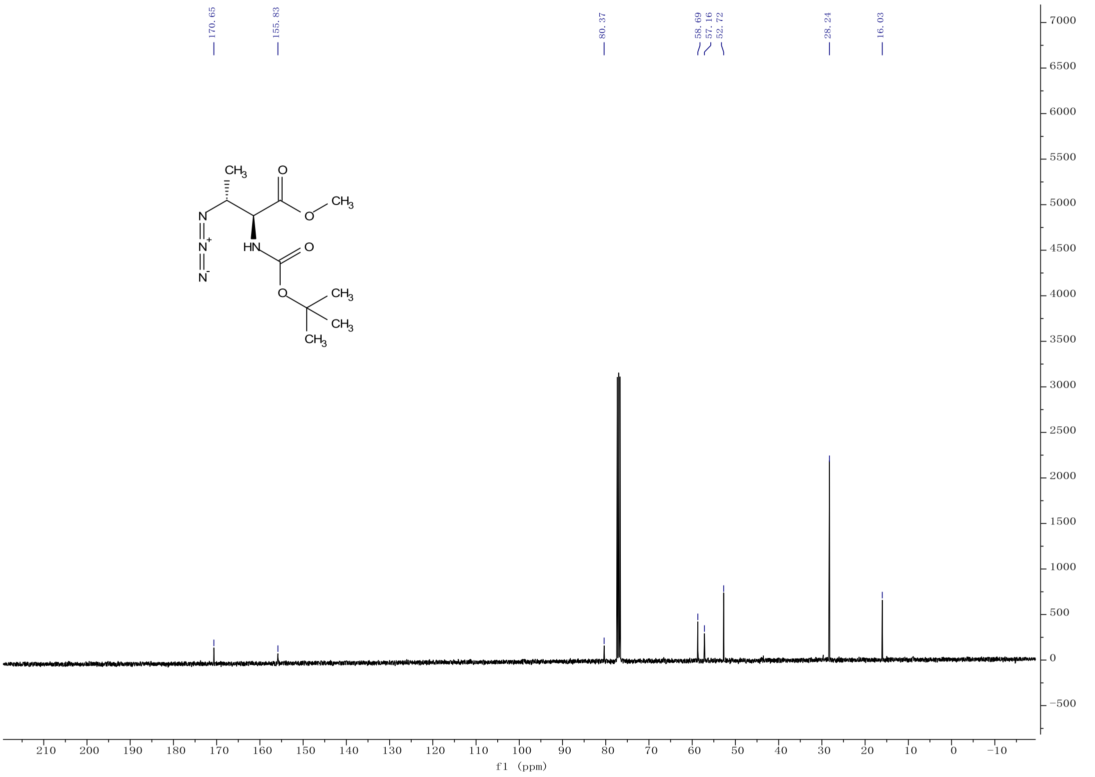

^{1}H NMR spectra of **MIS-13**

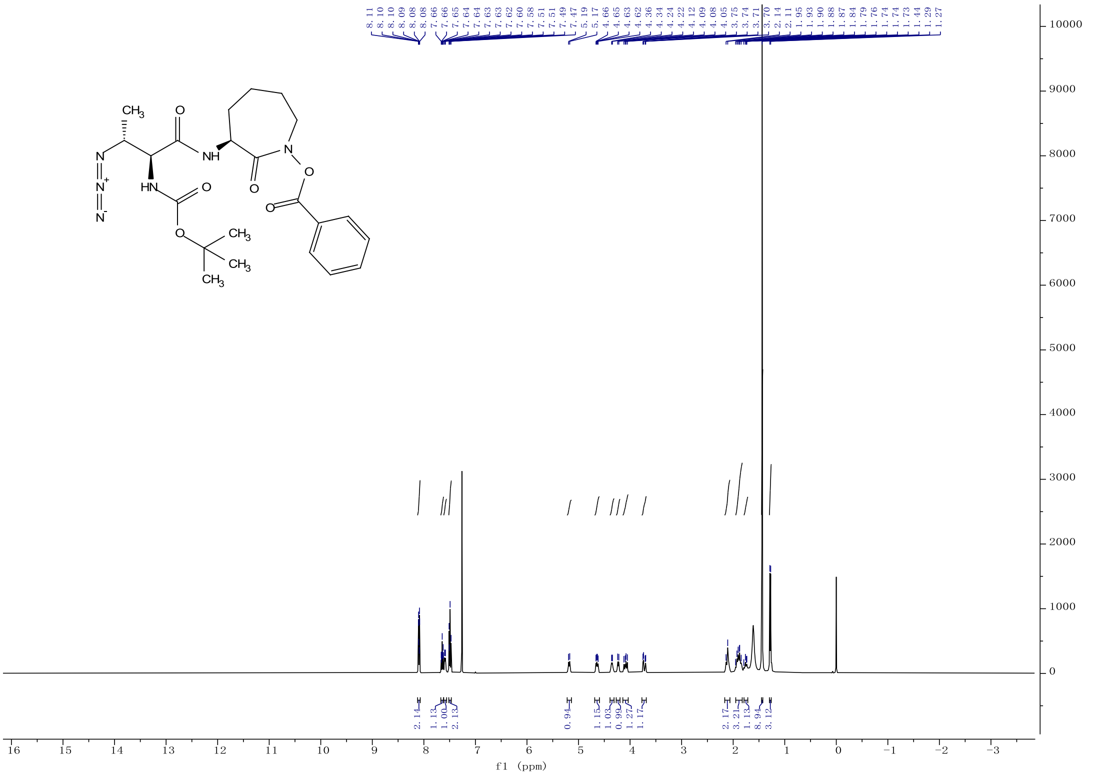

^{13}C NMR spectra of **MIS-13**

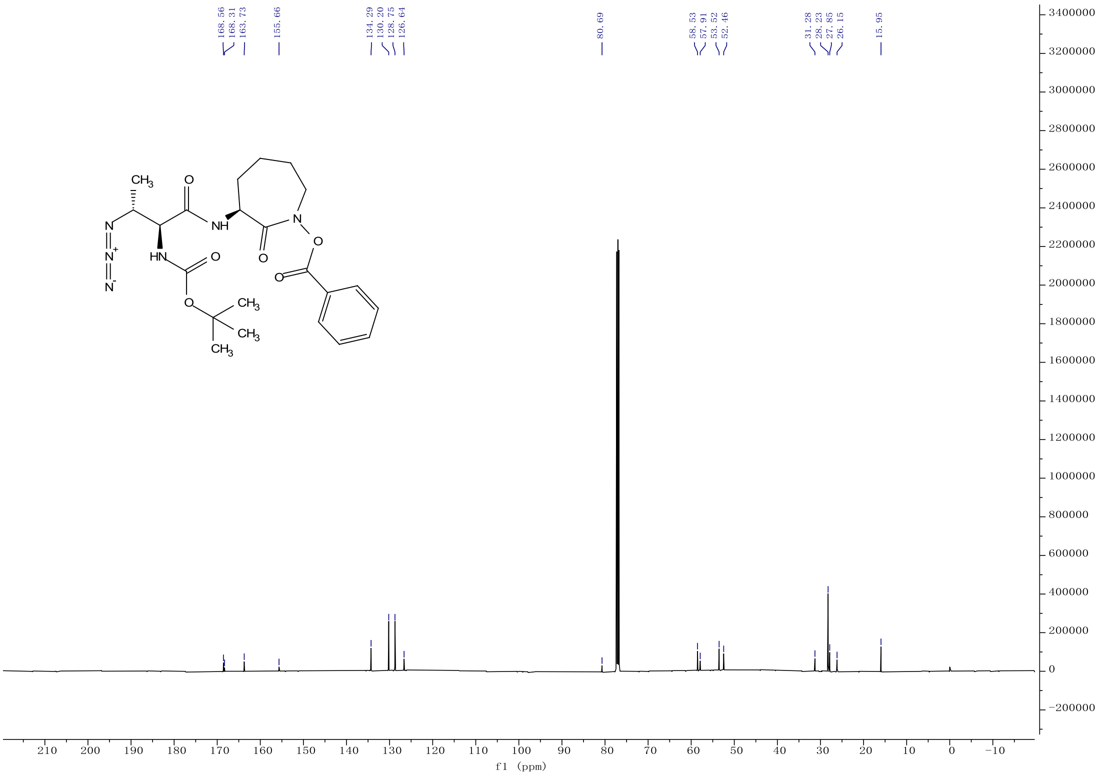

^{1}H NMR spectra of **Rho-piperazine**

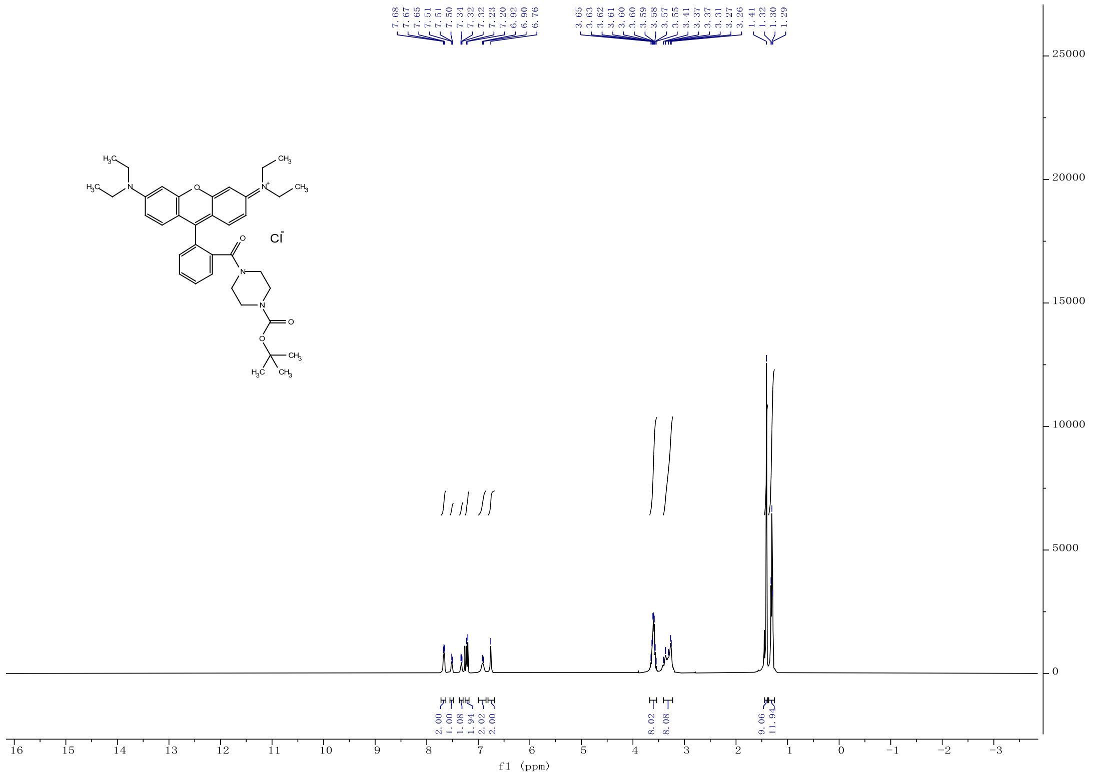

^{13}C NMR spectra of **Rho-piperazine**

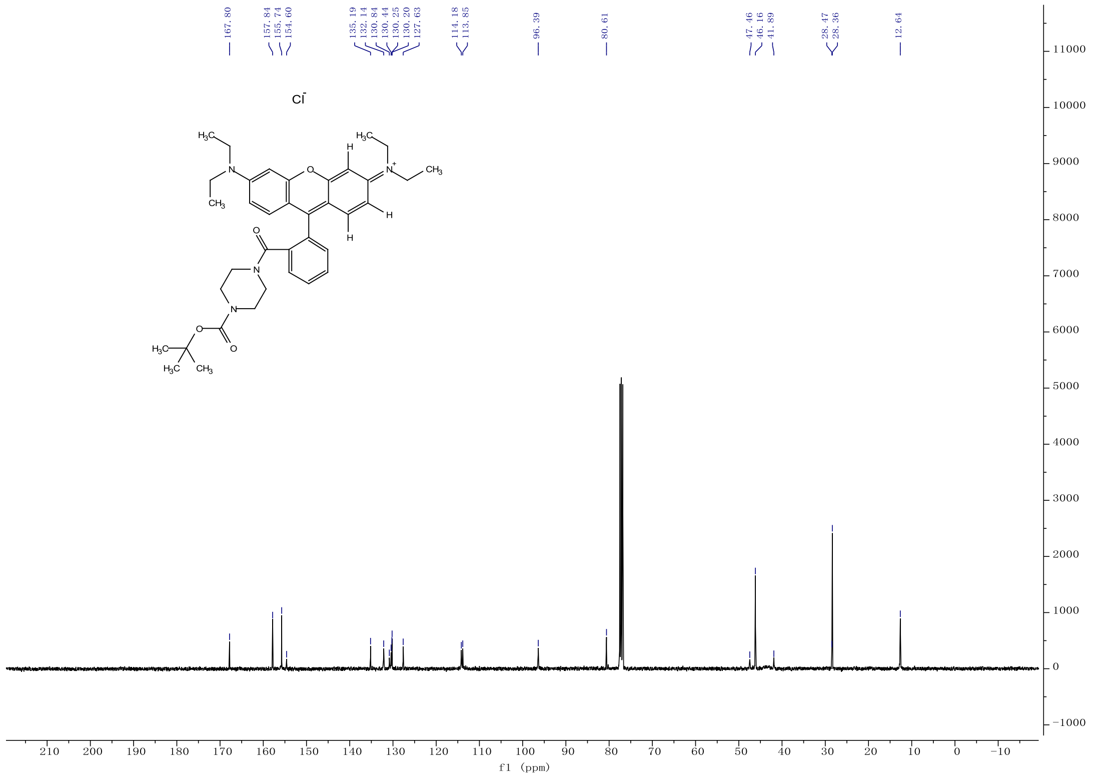

^{1}H NMR spectra of **N14A**

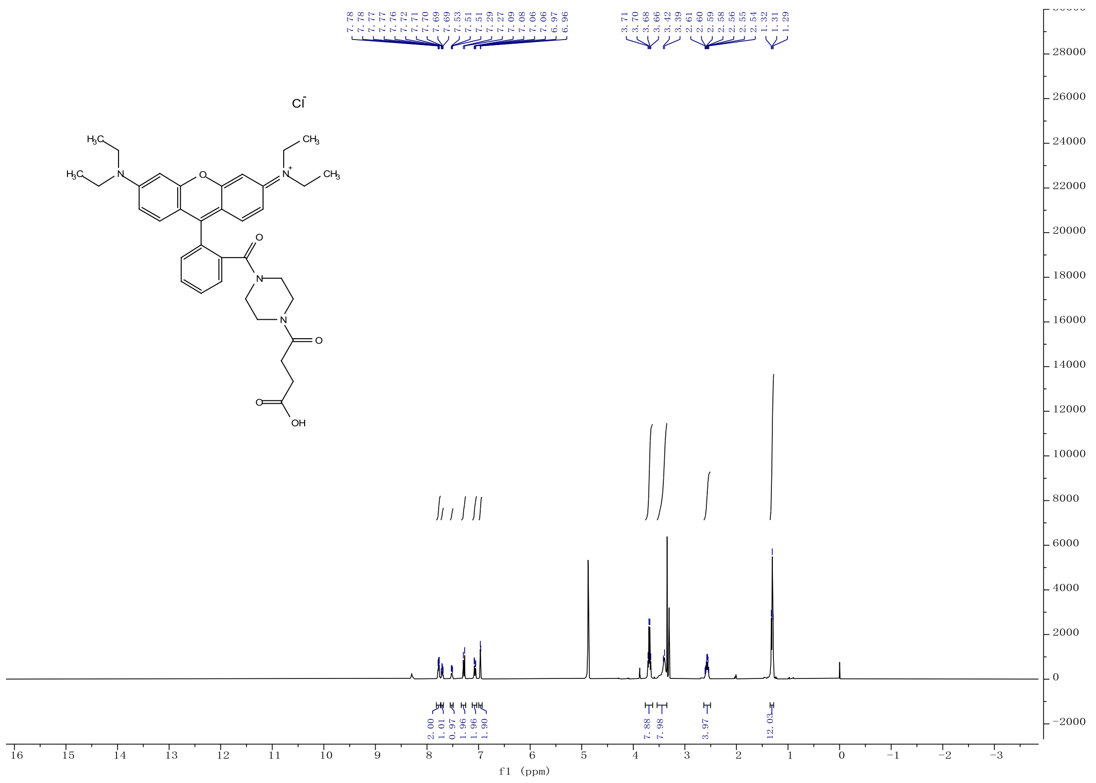

^{13}C NMR spectra of **N14A**

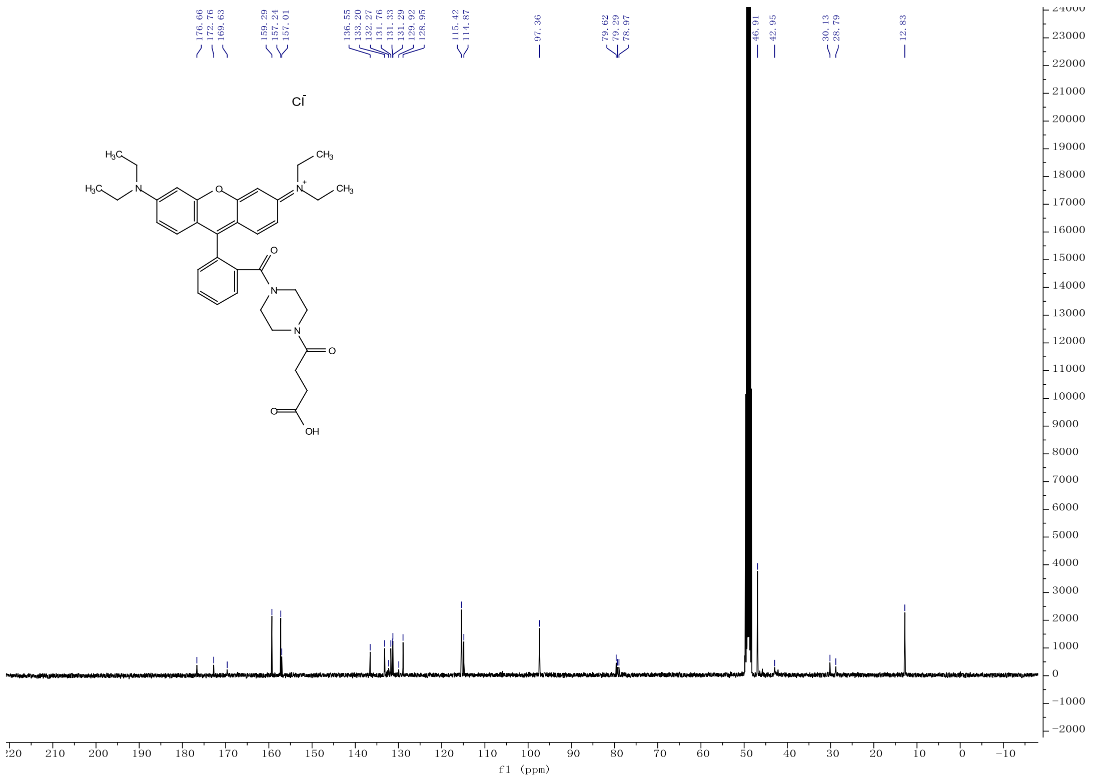

^{1}H NMR spectra of **P10C-16**

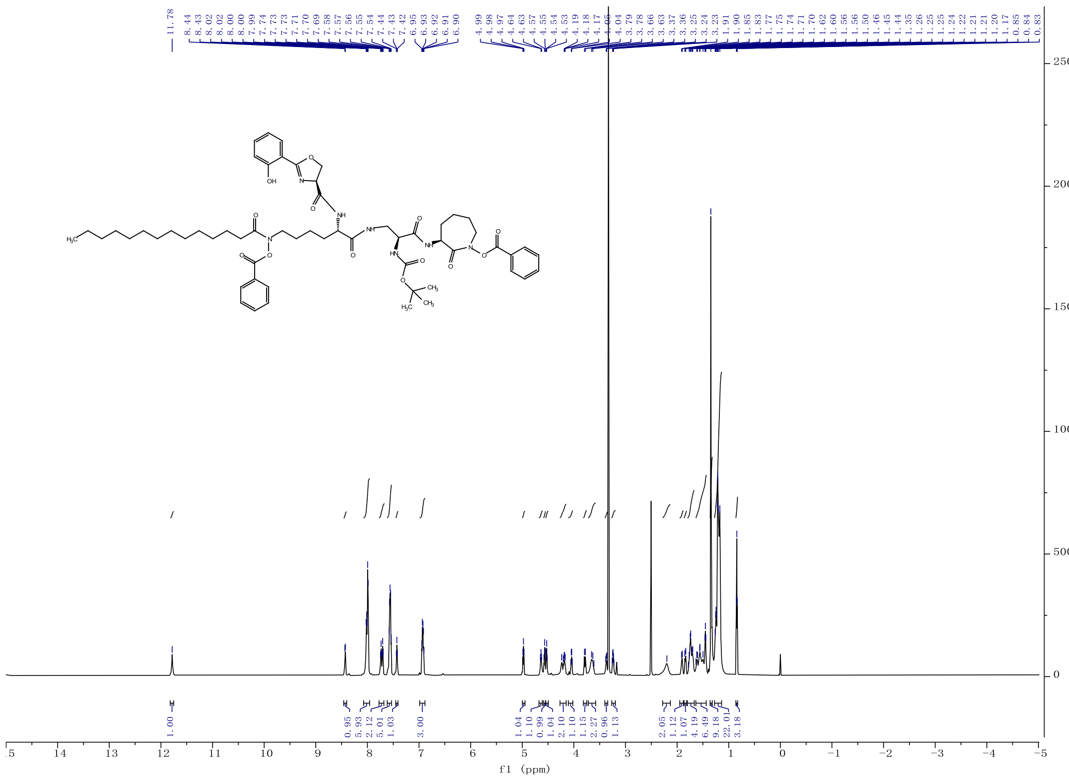

^{13}C NMR spectra of **P10C-16**

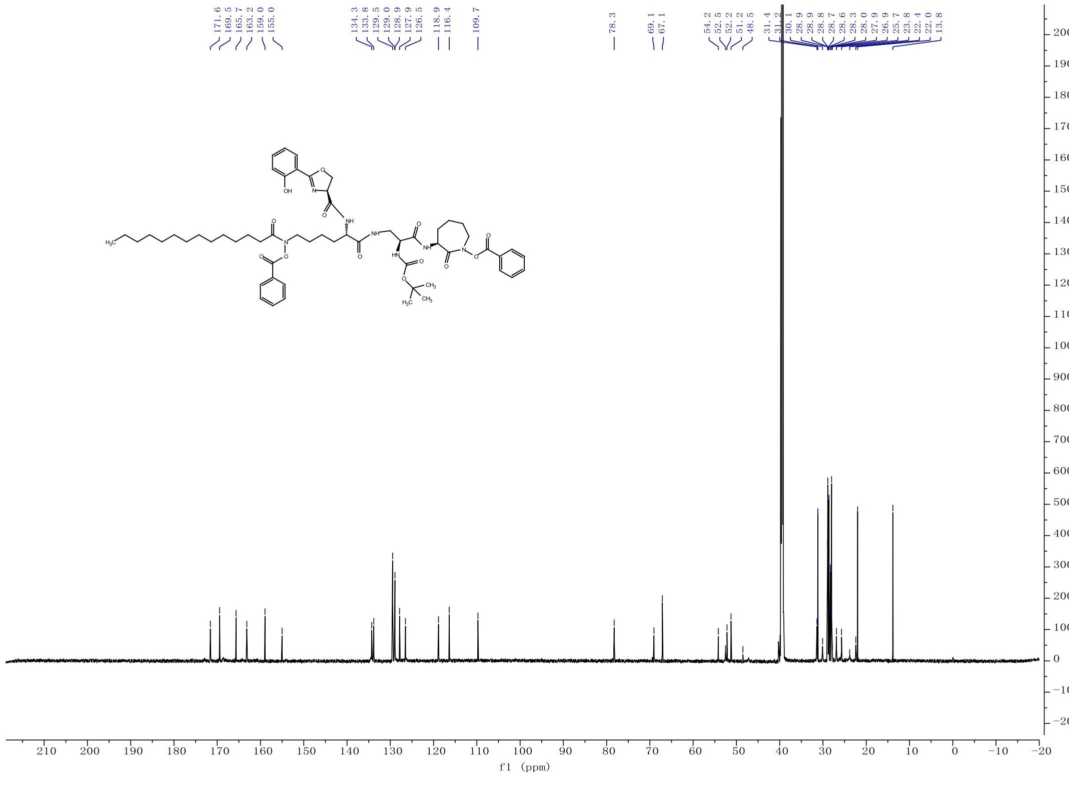

^{1}H NMR spectra of **P10C**

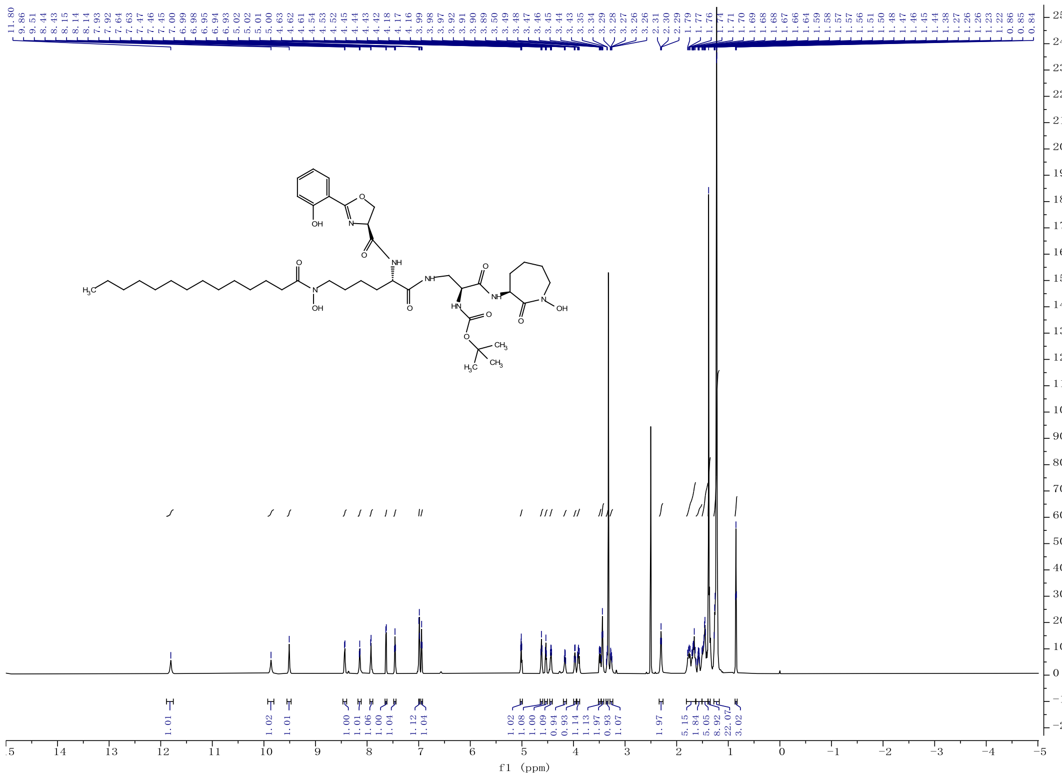

^{13}C NMR spectra of **P10C**

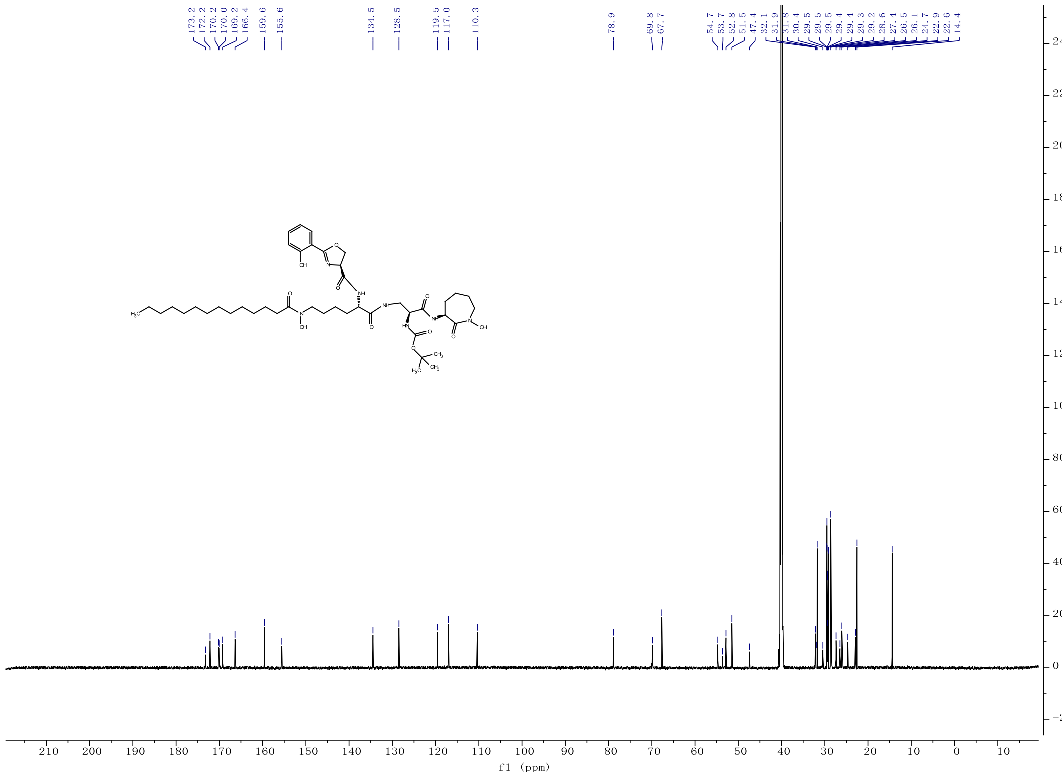

^{1}H NMR spectra of ***(S)*-P10C-16**

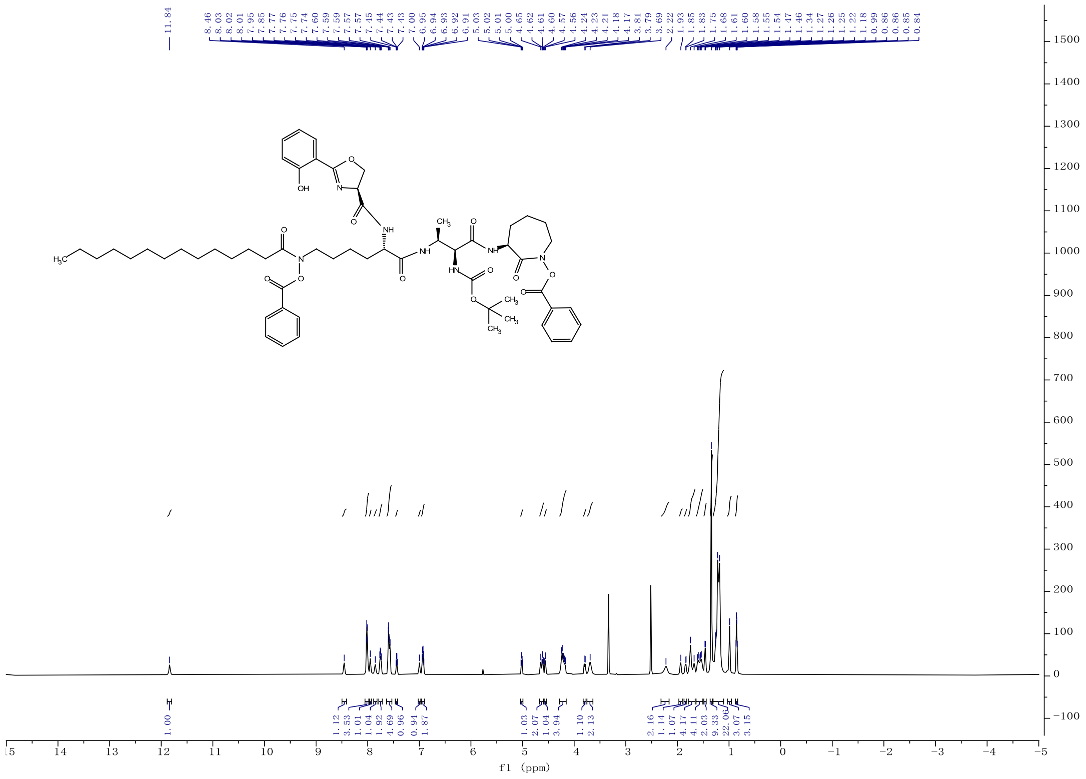

^{13}C NMR spectra of ***(S)*-P10C-16**

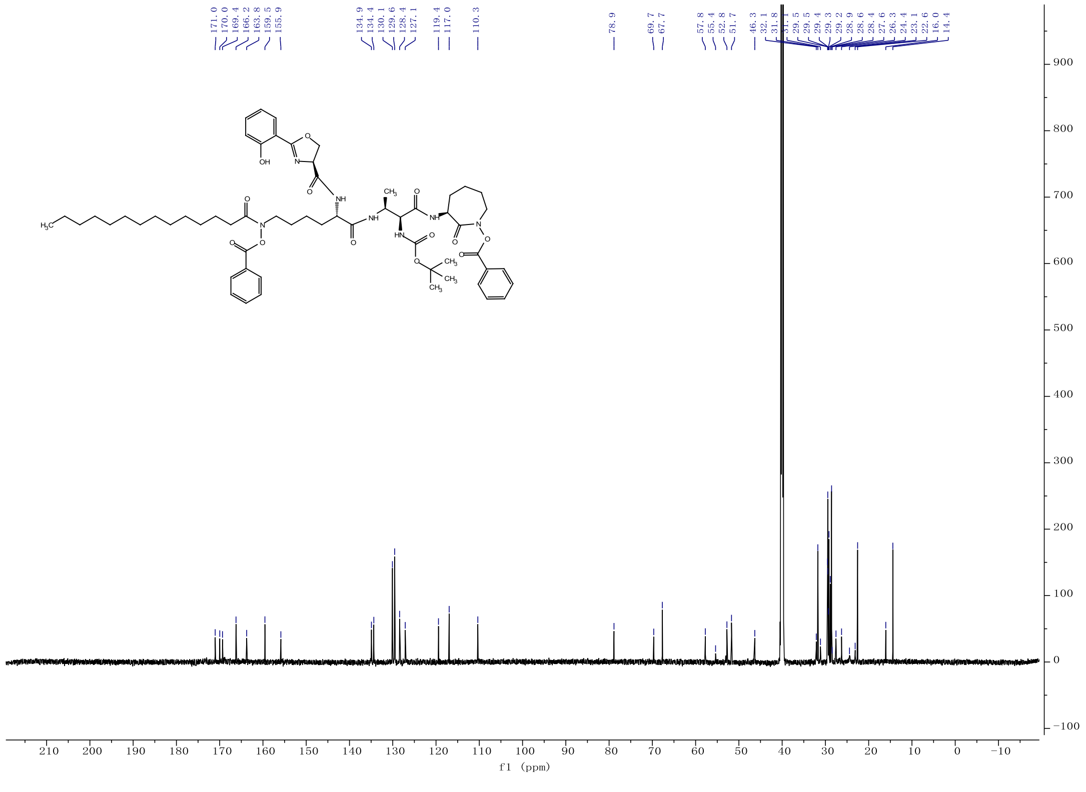

^{1}H NMR spectra of **(*S*)-P10C**

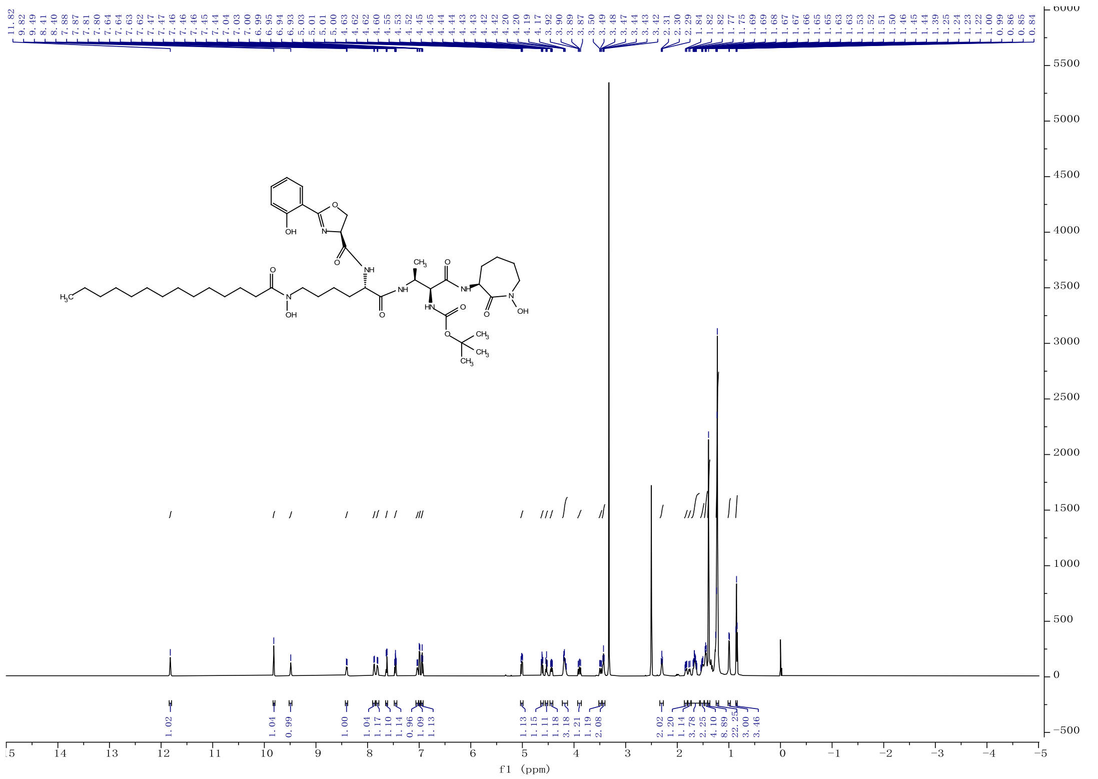

^{13}C NMR spectra of **(*S*)-P10C**

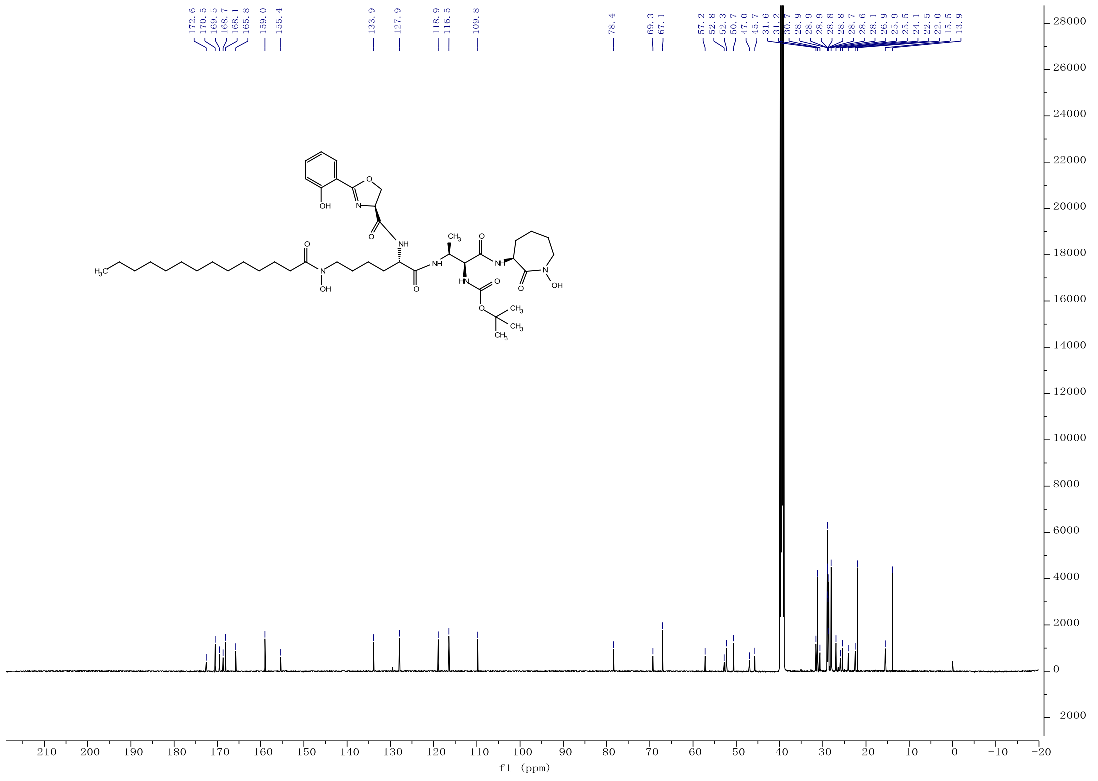

^{1}H NMR spectra of **(*R*)-P10C-16**

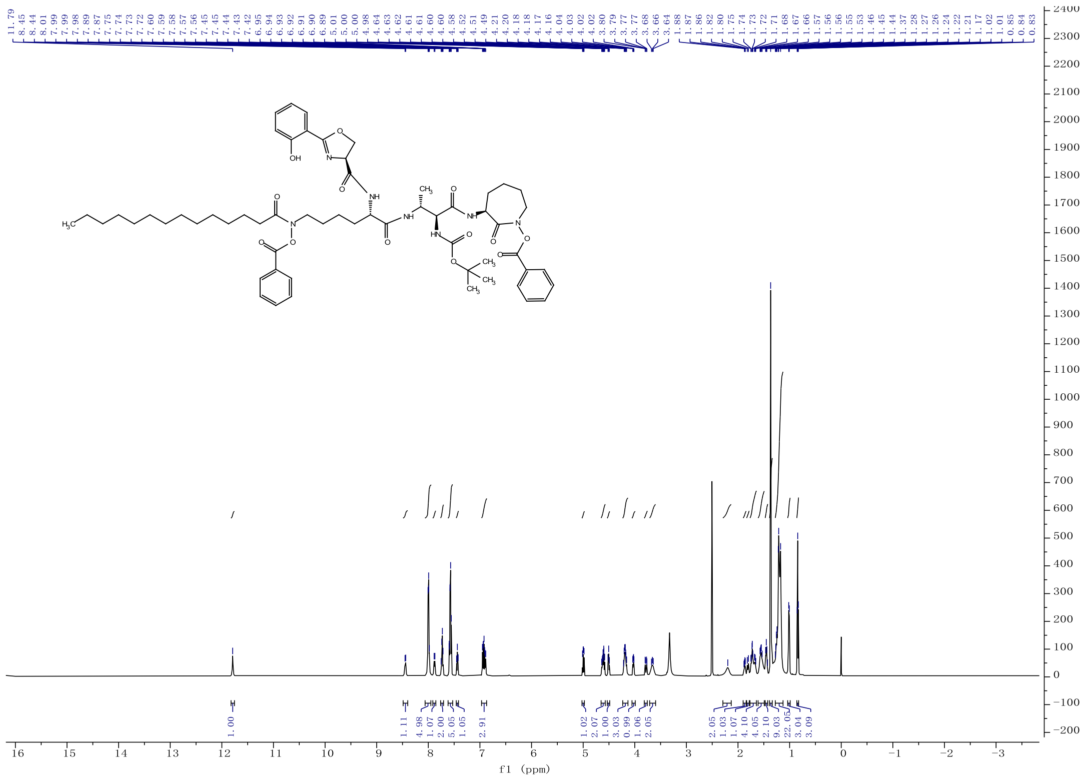

^{13}C NMR spectra of **(*R*)-P10C-16**

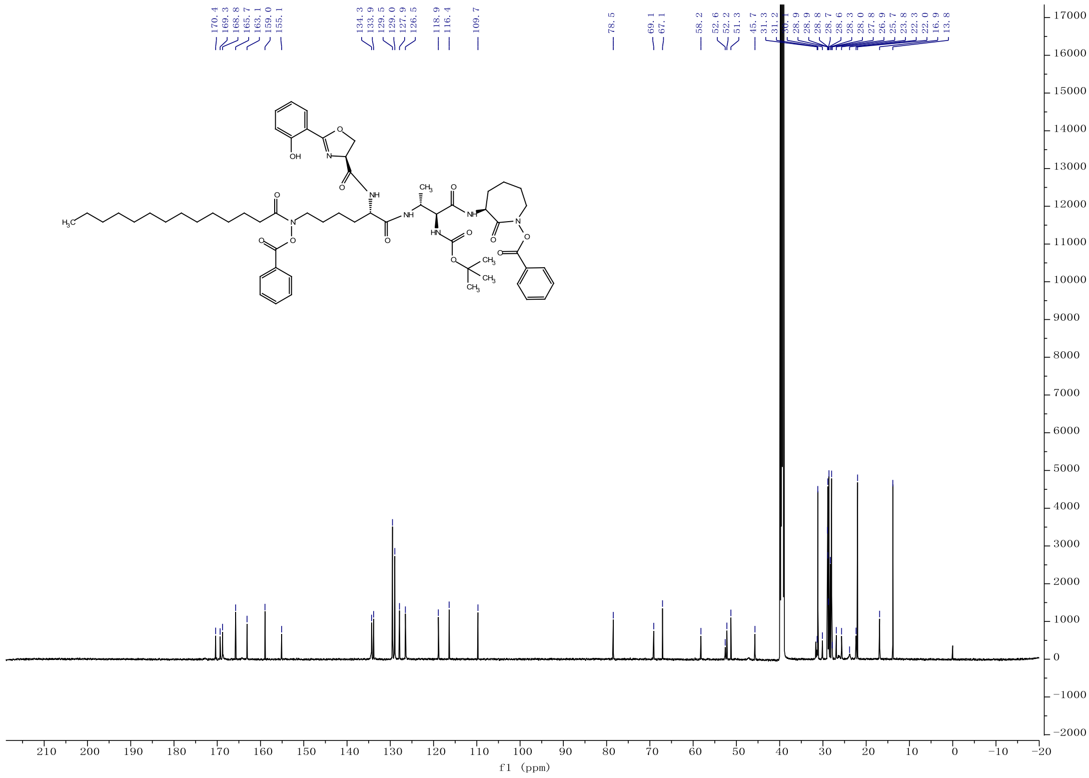

^{1}H NMR spectra of ***(R)*-P10C**

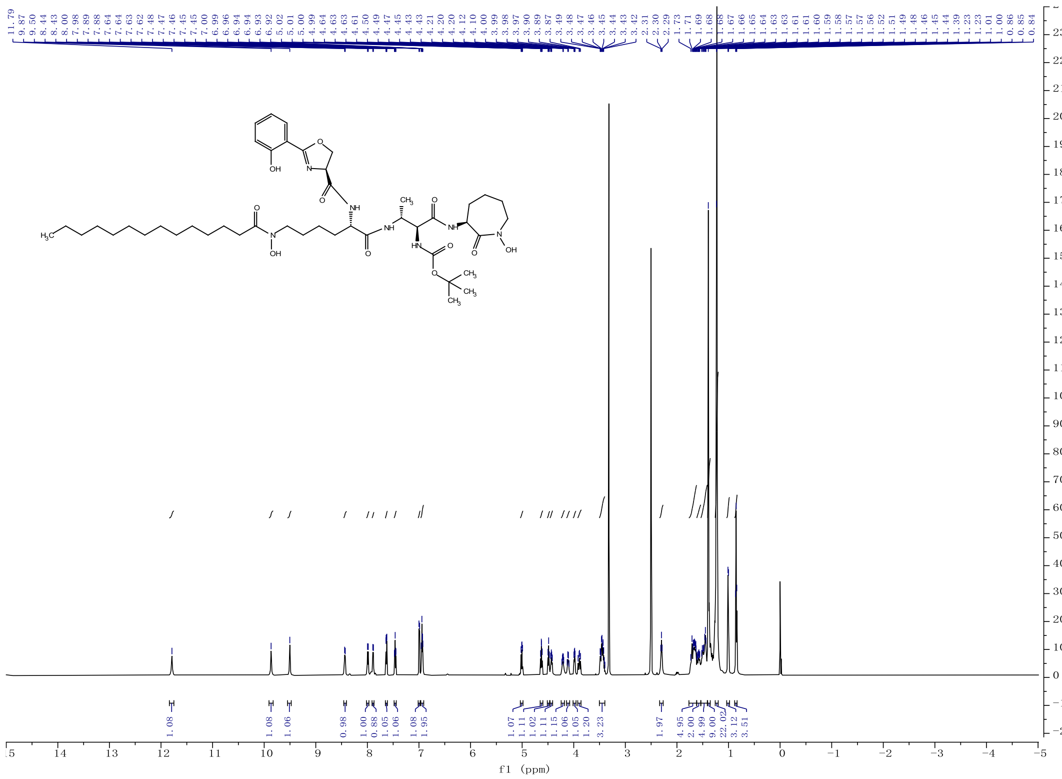

^{13}C NMR spectra of ***(R)*-P10C**

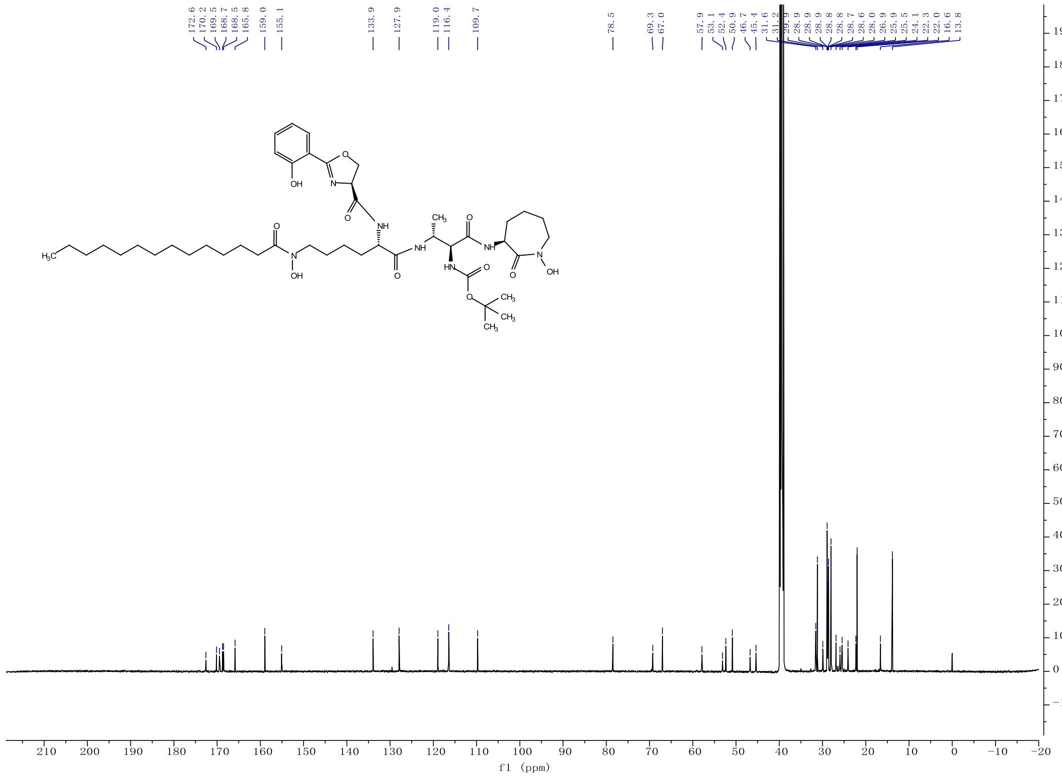

^{1}H NMR spectra of **N14C**

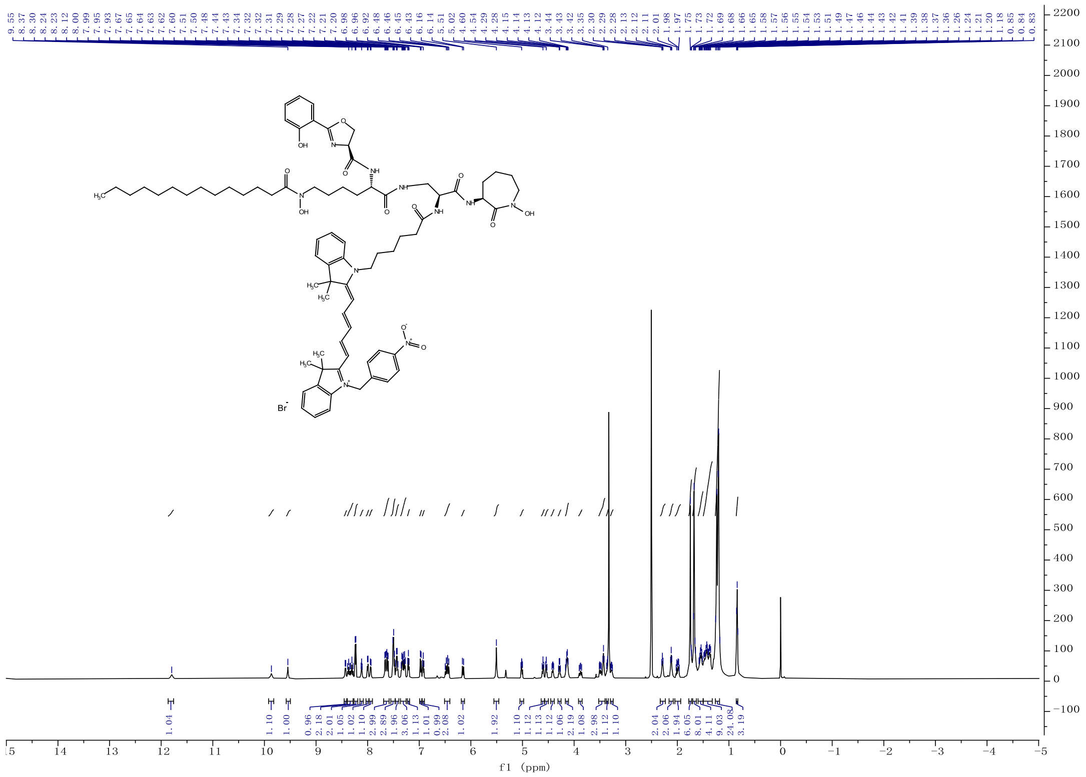

^{13}C NMR spectra of **N14C**

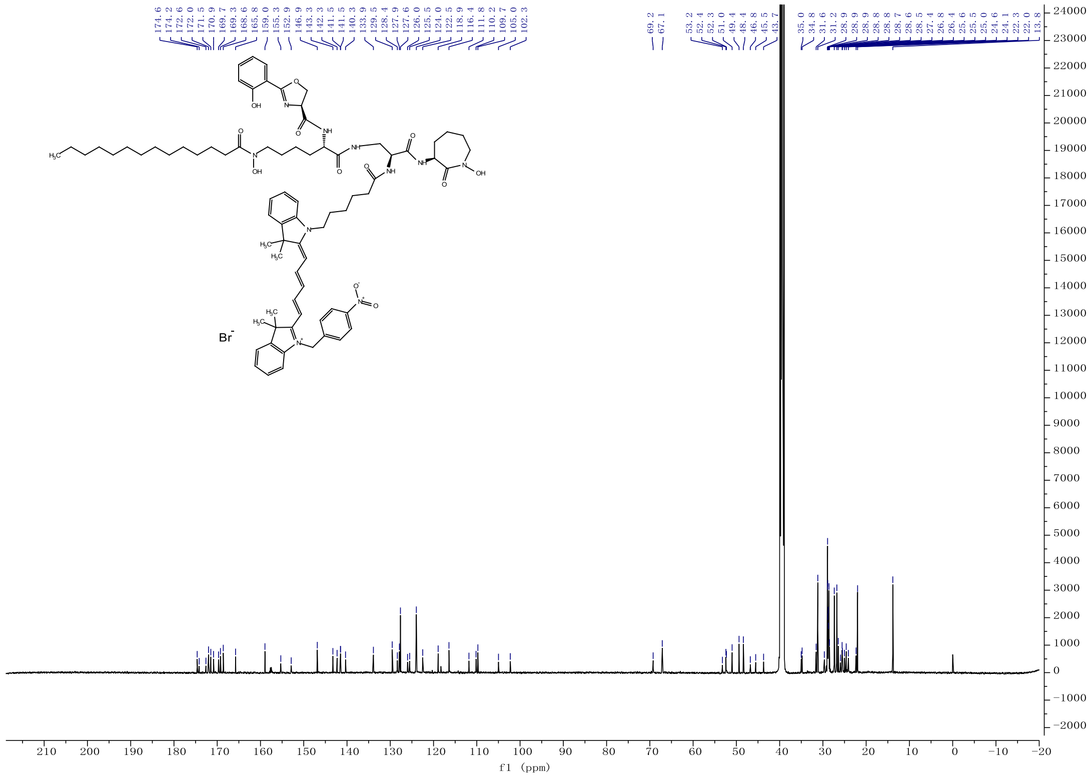

^{1}H NMR spectra of **N14E**

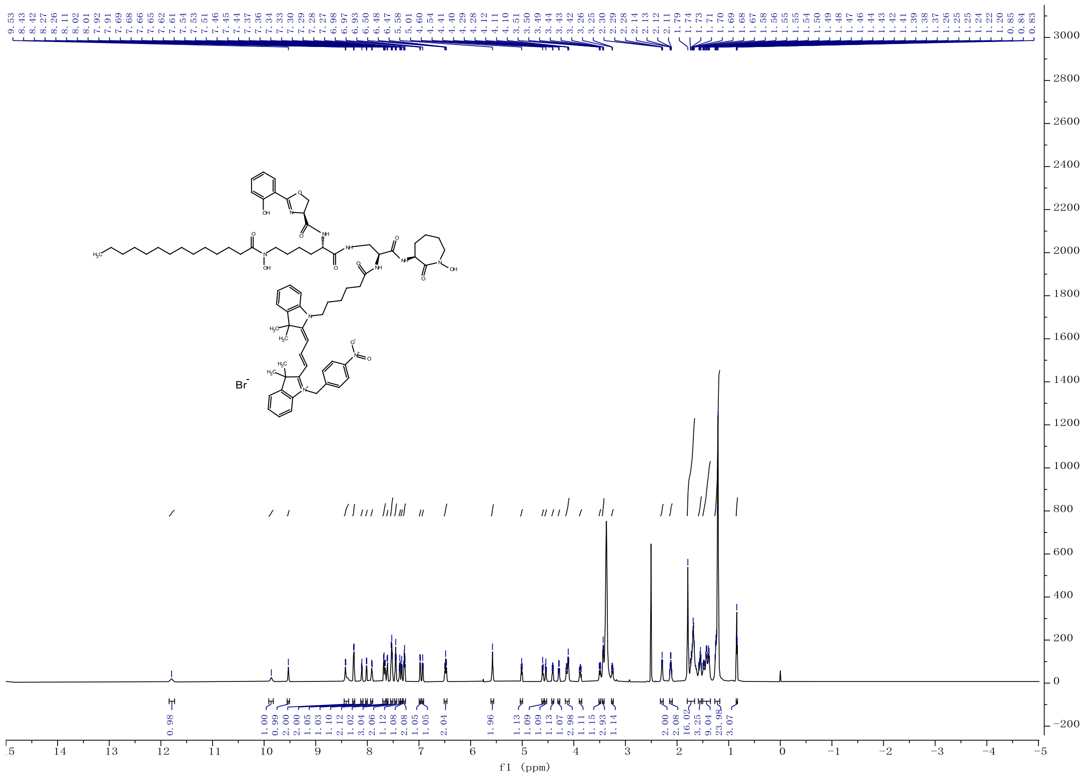

^{13}C NMR spectra of **N14E**

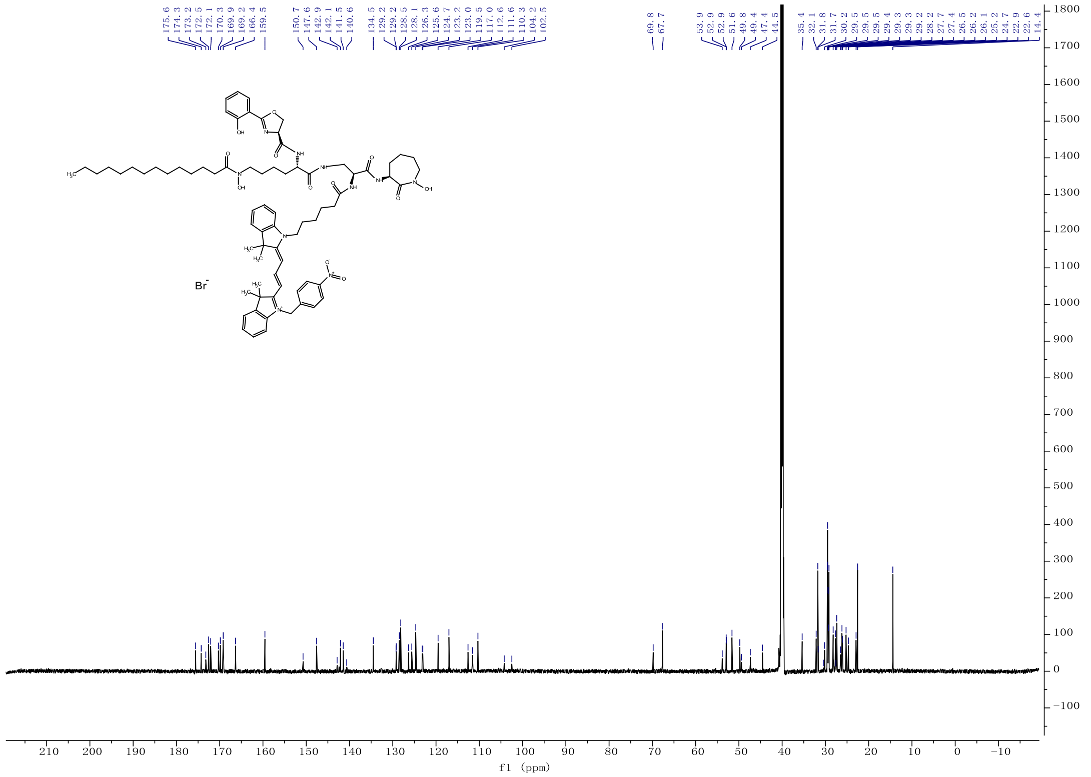

^{1}H NMR spectra of **N14F**

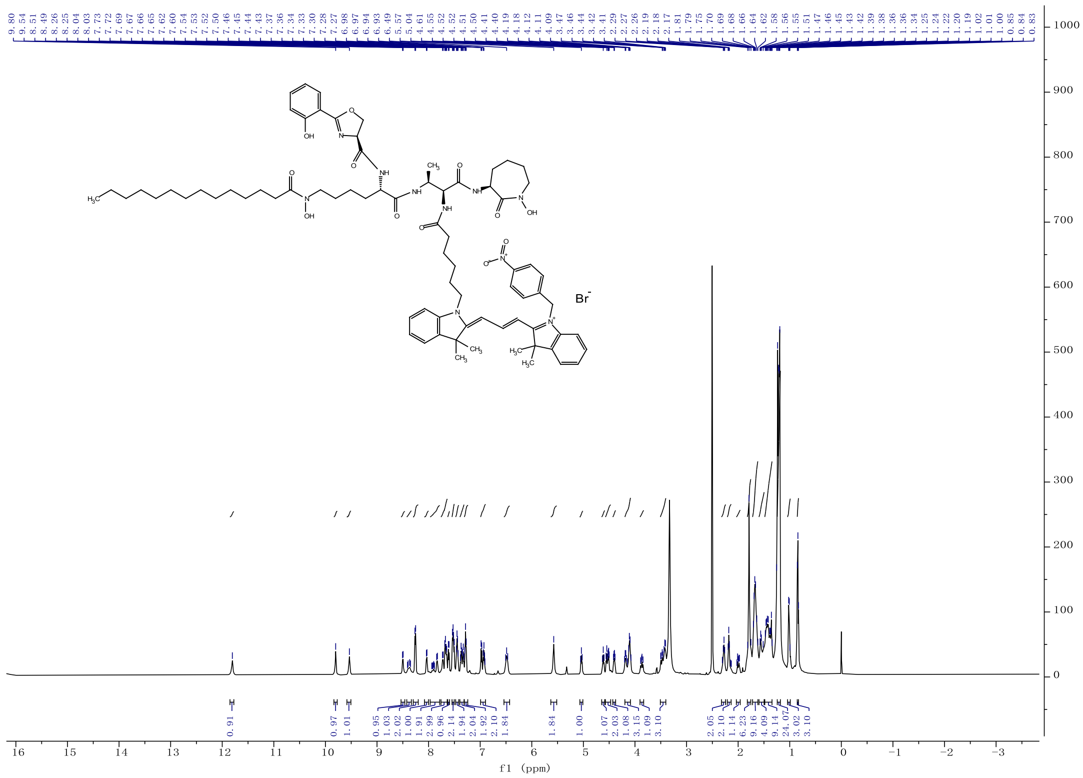

^{13}C NMR spectra of **N14F**

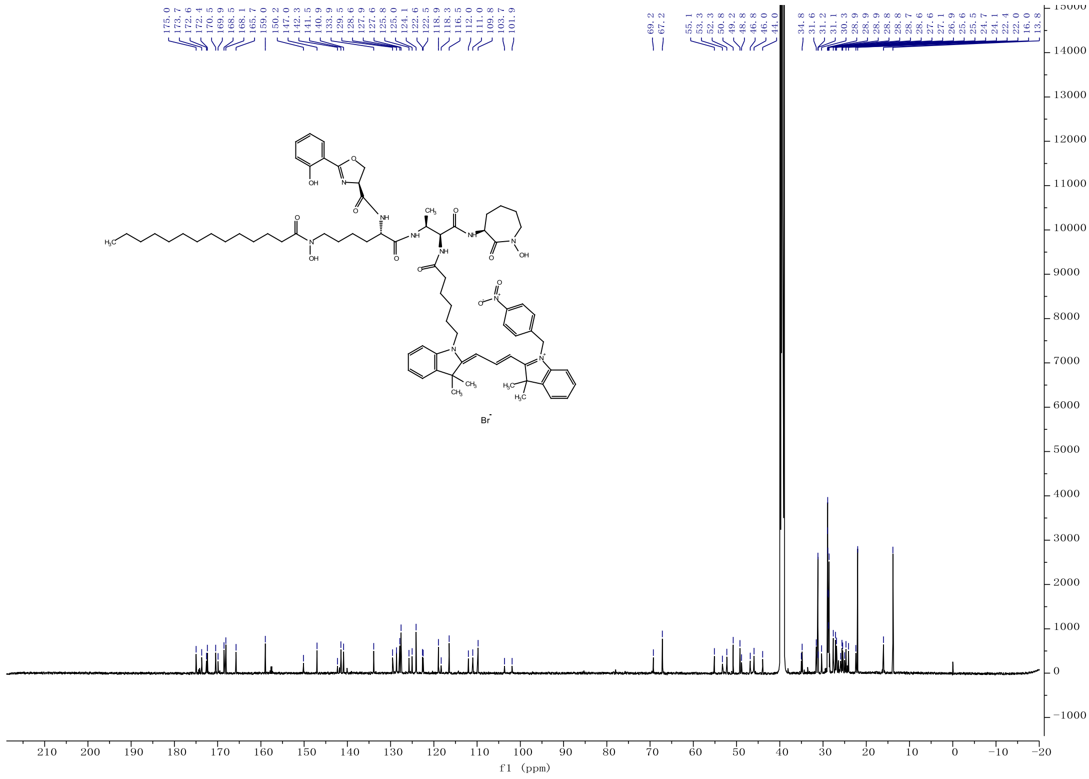

^{1}H NMR spectra of **N14G**

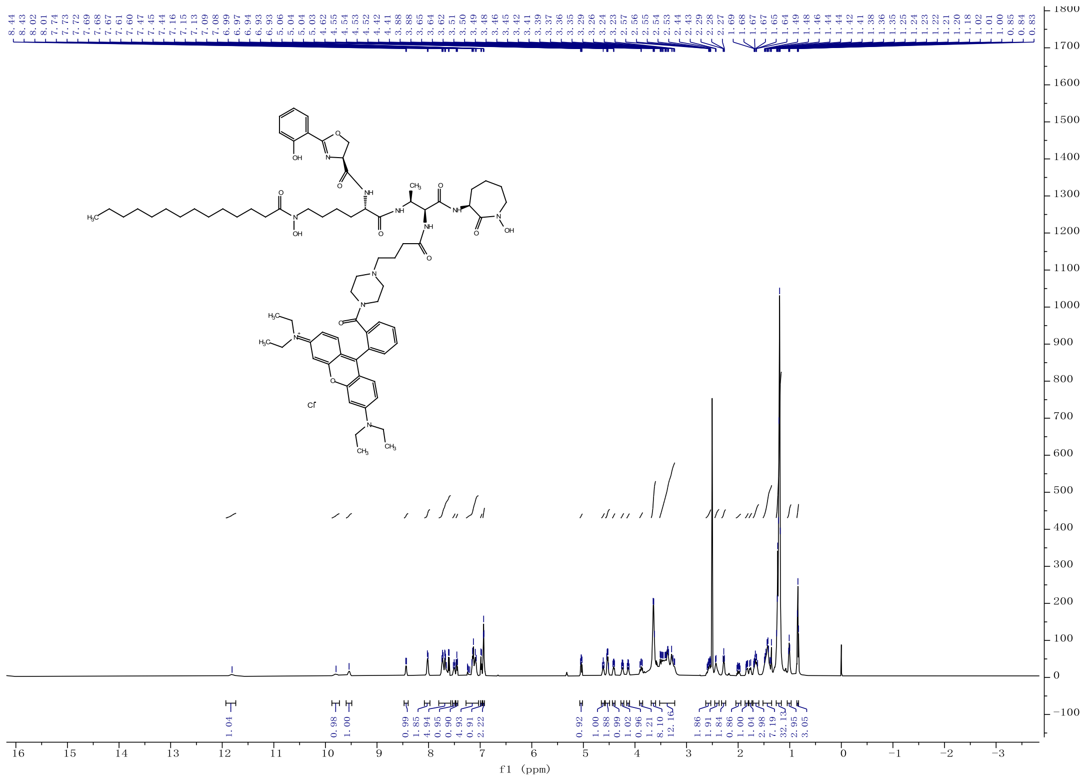

^{13}C NMR spectra of **N14G**

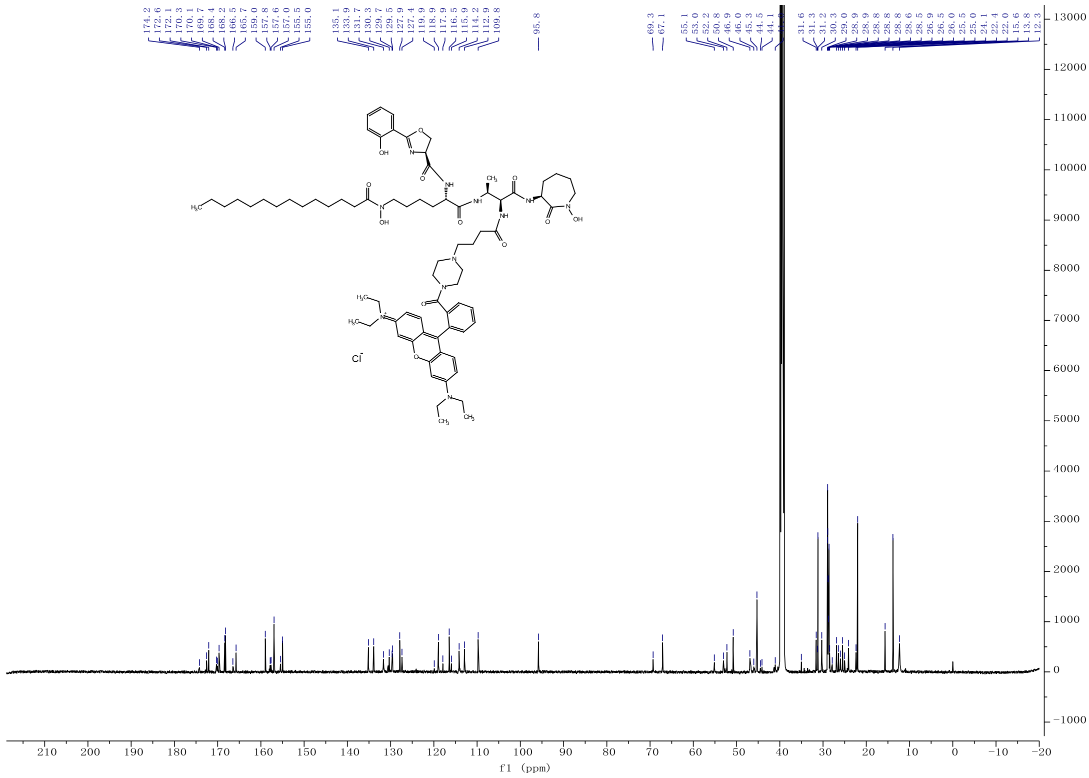

^{1}H NMR spectra of **N14H**

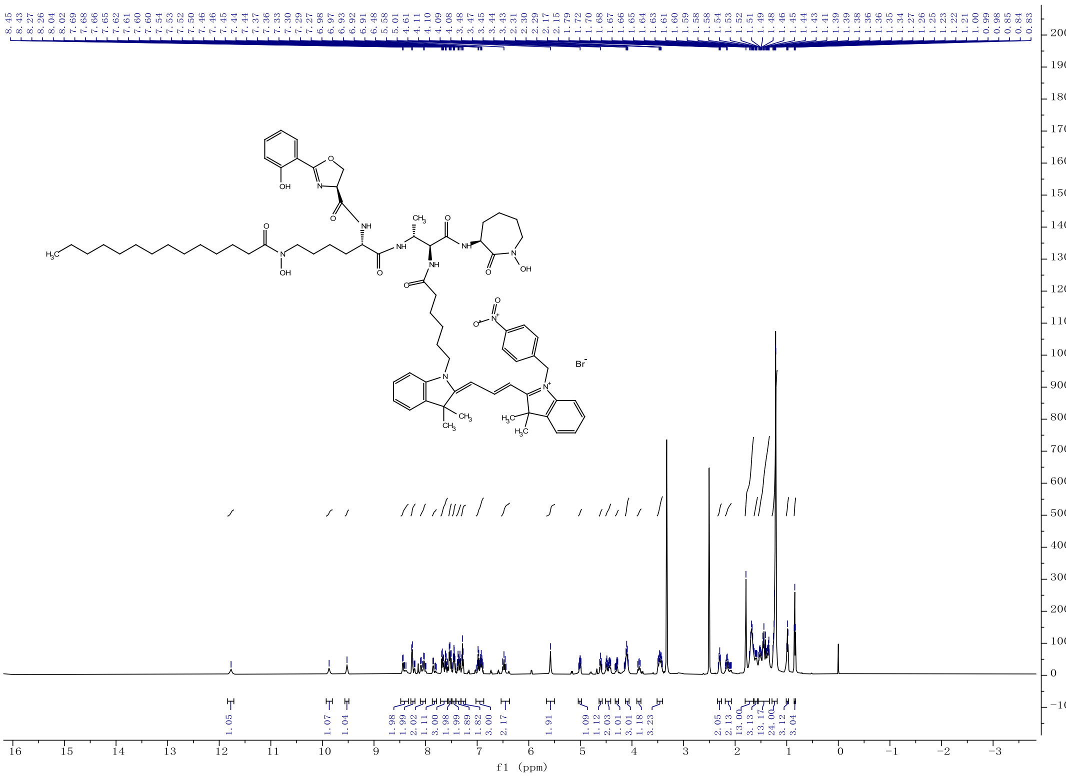

^{13}C NMR spectra of **N14H**

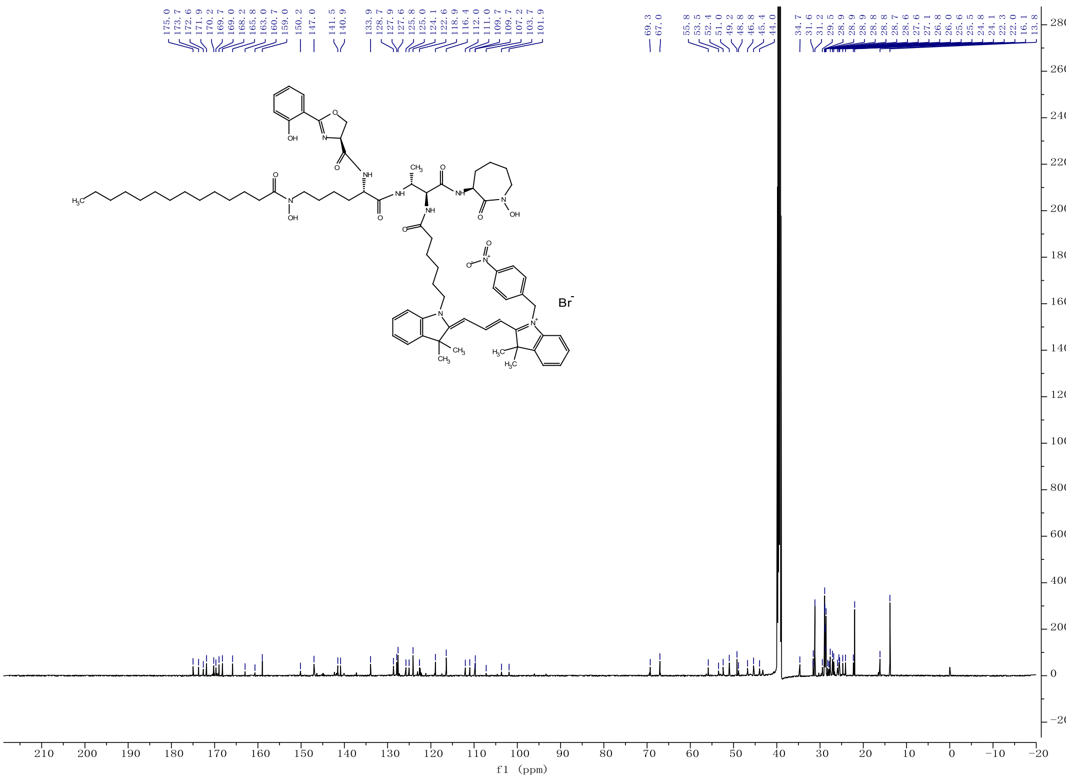

^{1}H NMR spectra of **N14I**

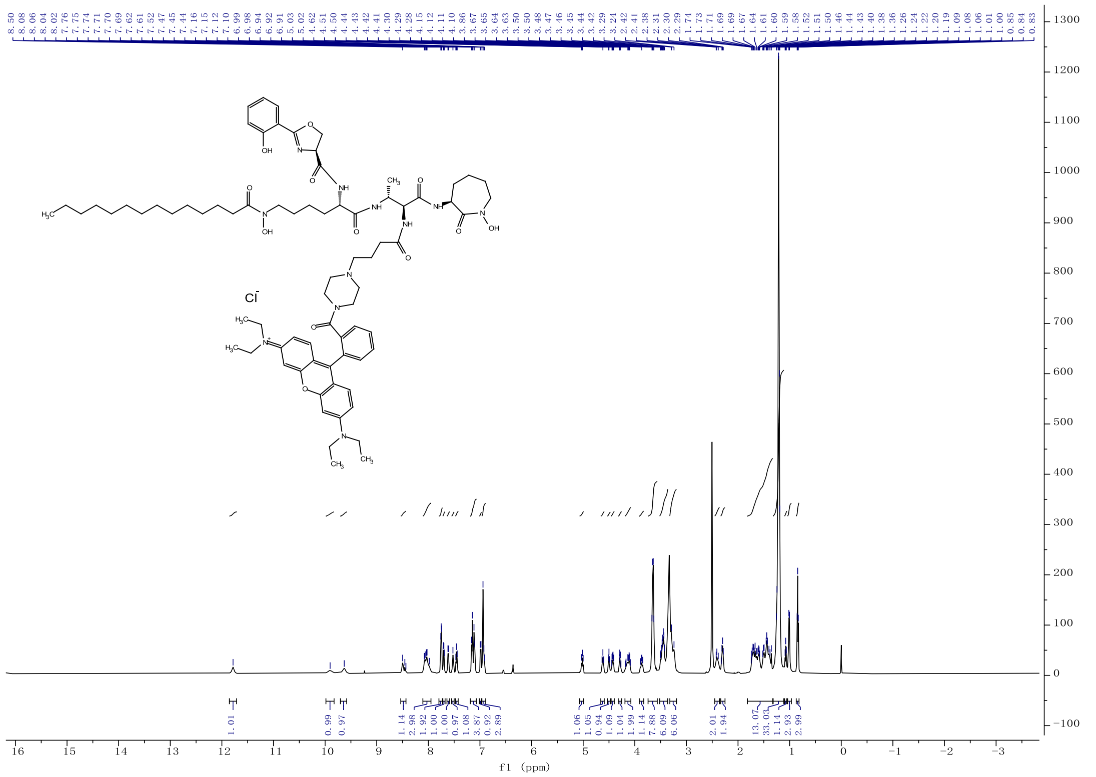

^{13}C NMR spectra of **N14I**

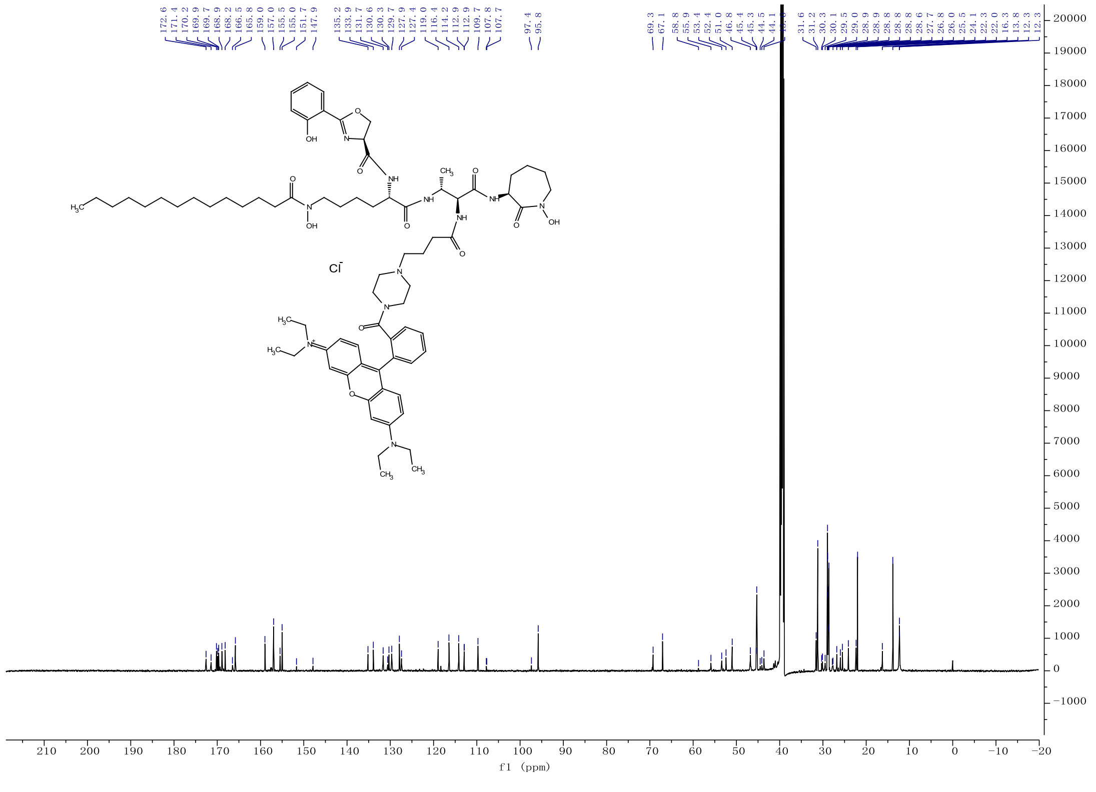

^{1}H NMR spectra of **N14J**

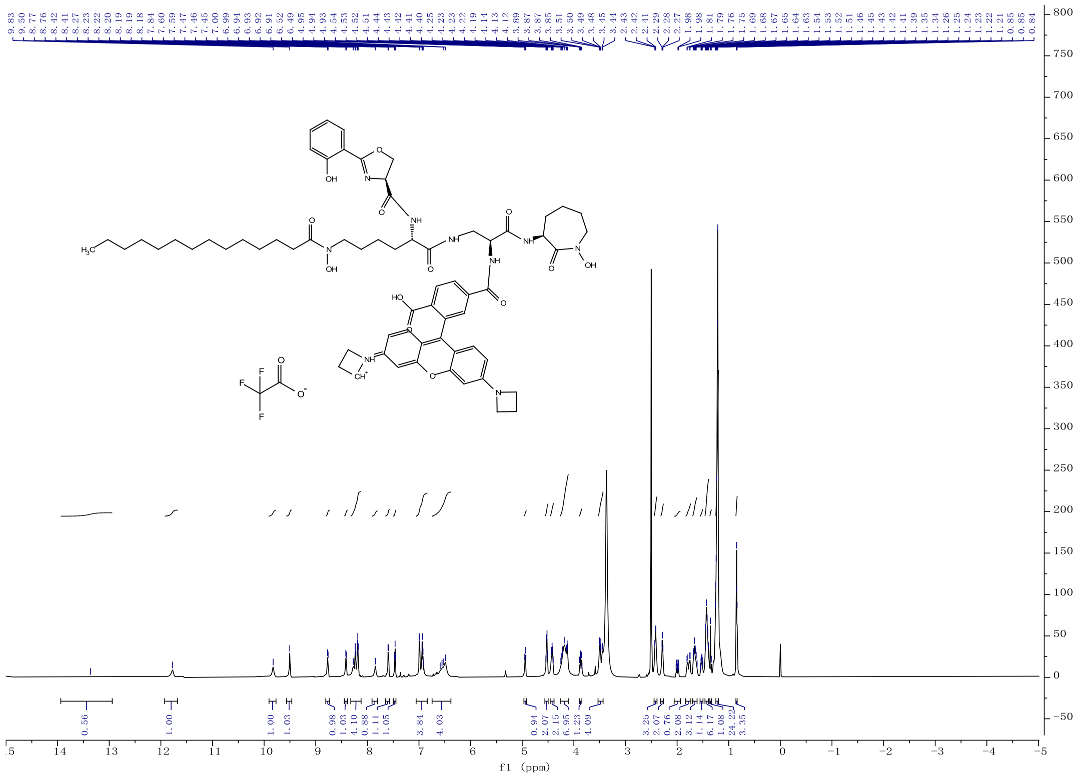

^{19}F NMR spectra of **N14J**

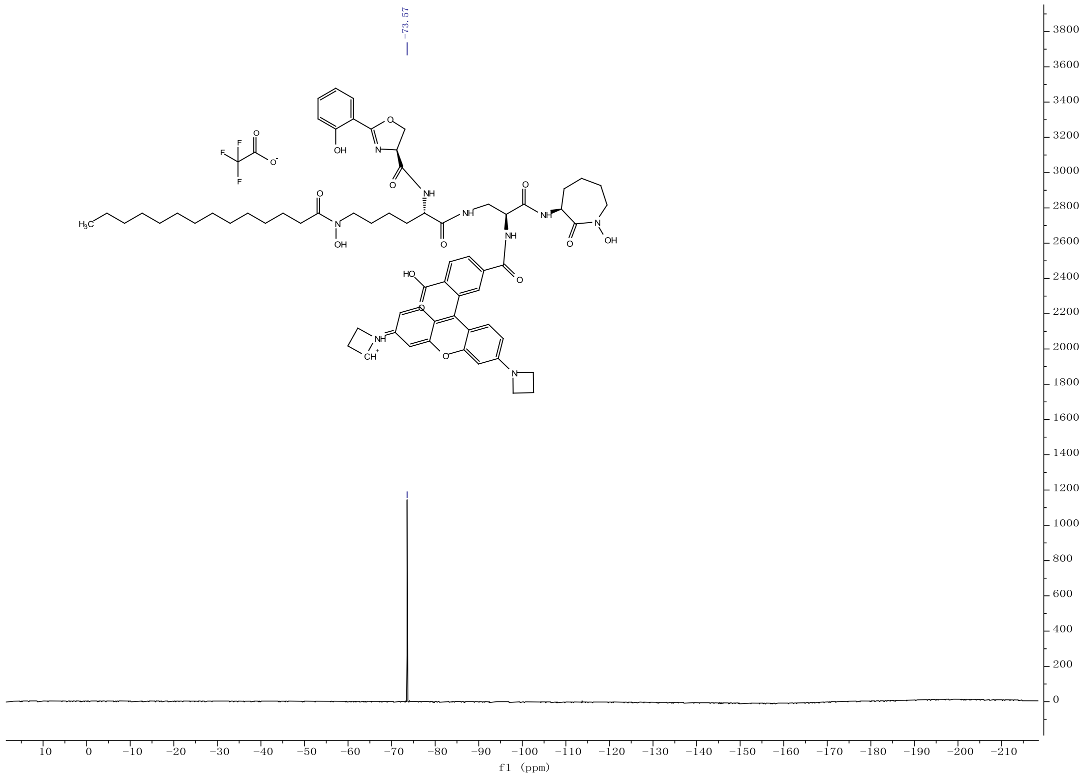

^{13}C NMR spectra of **N14J**

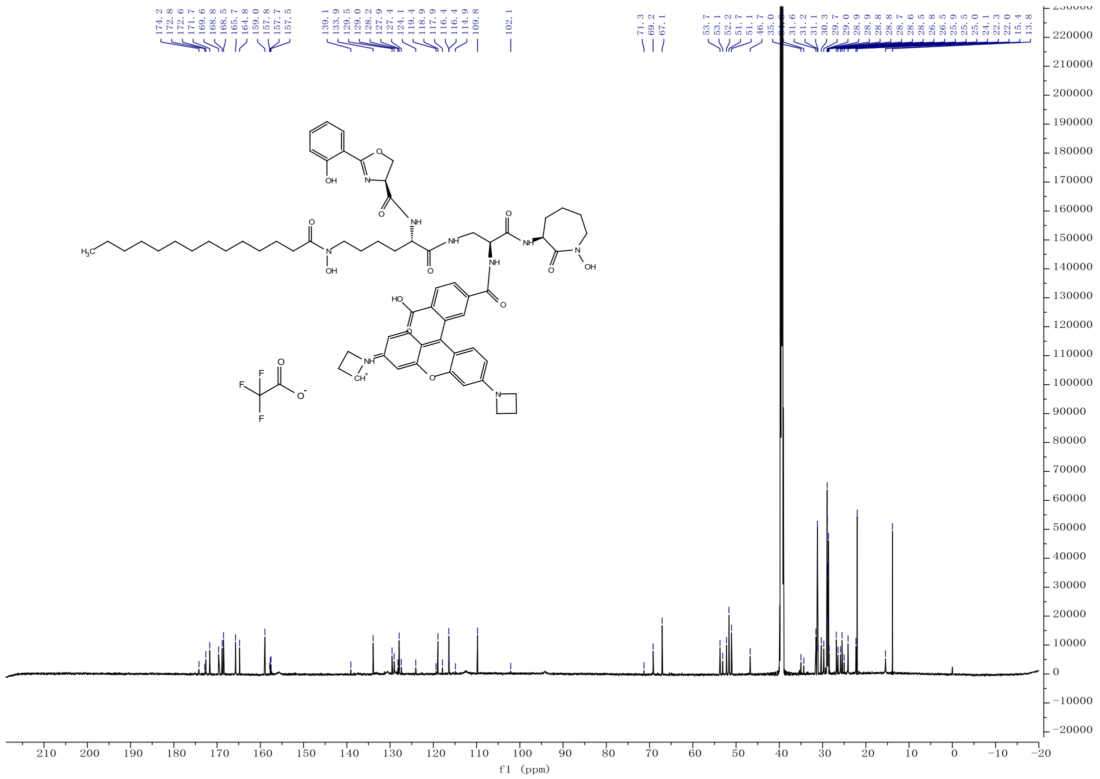

^{1}H NMR spectra of **N14K**

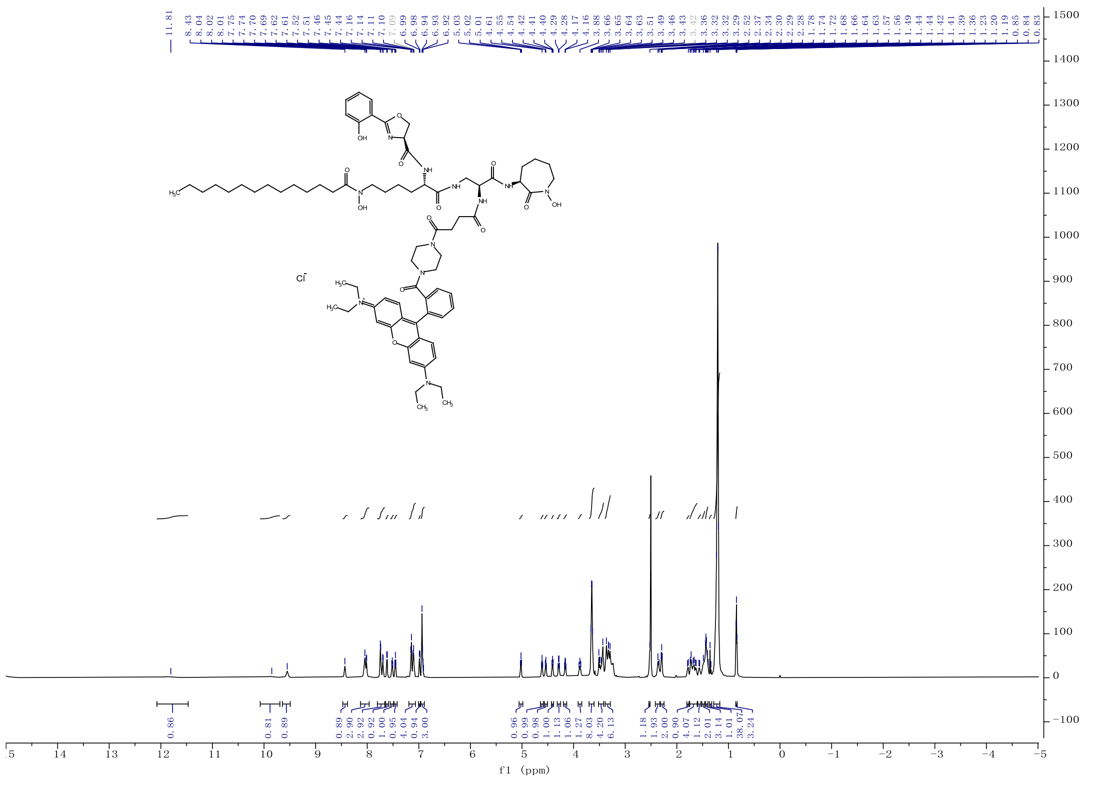

^{13}C NMR spectra of **N14K**

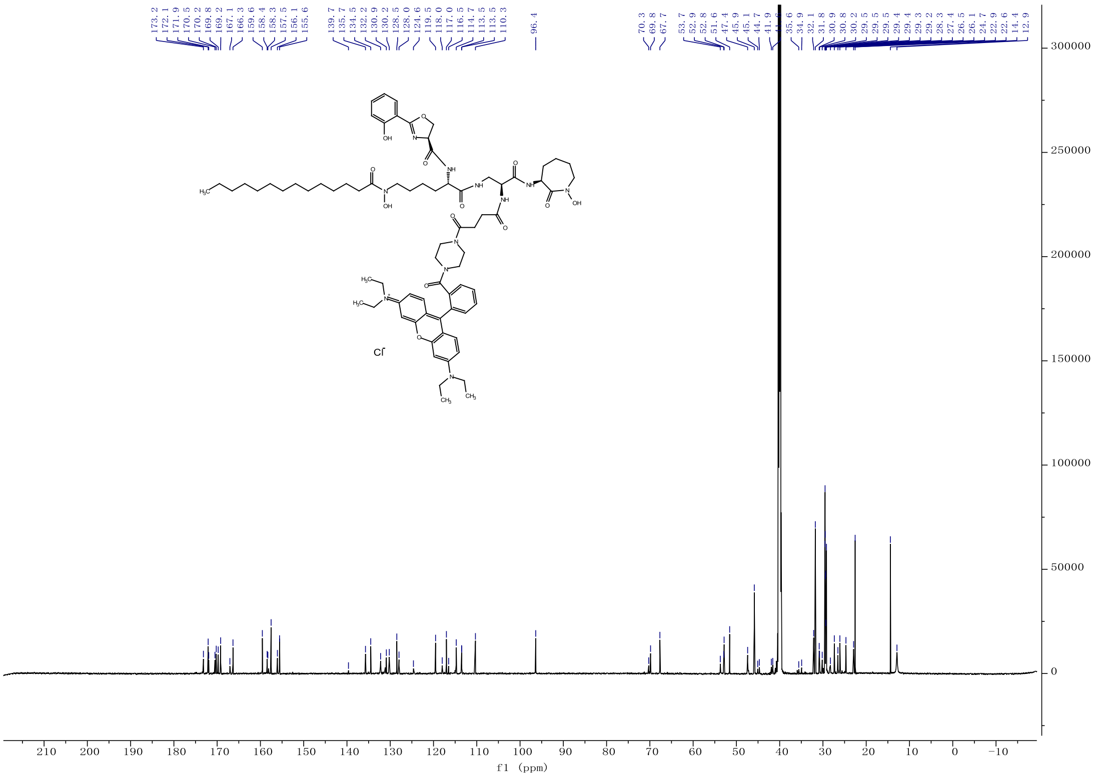

^{1}H NMR spectra of **N14L**

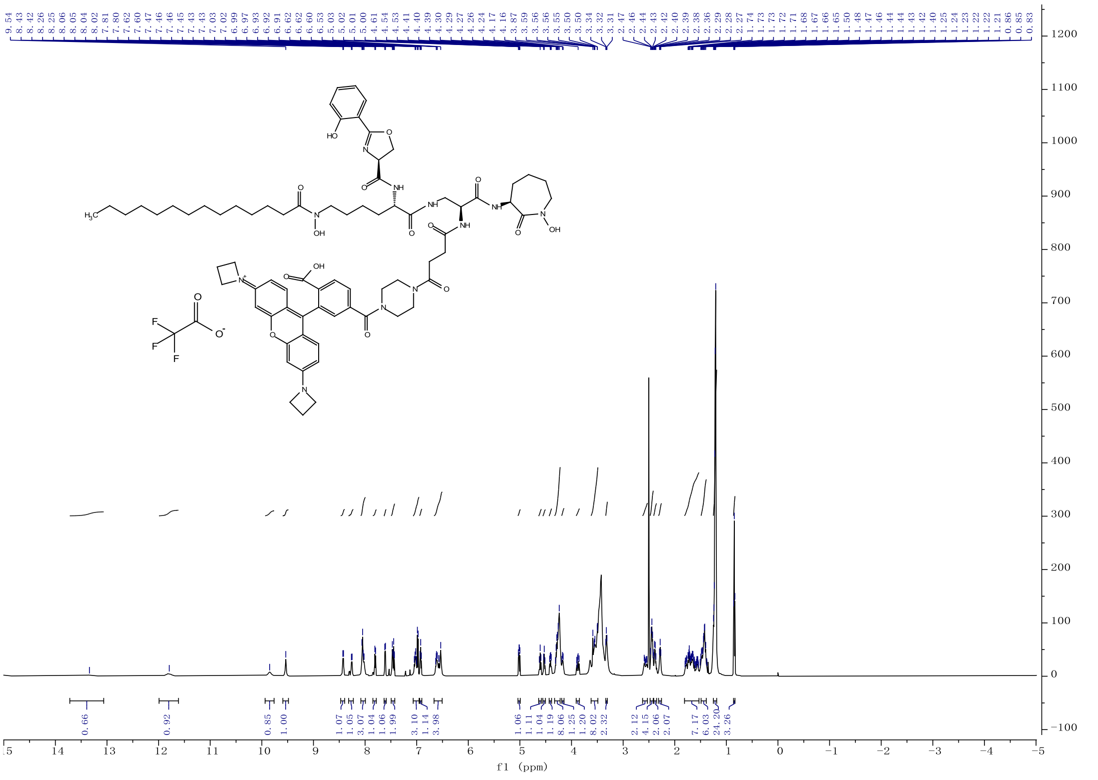

^{19}F NMR spectra of **N14L**

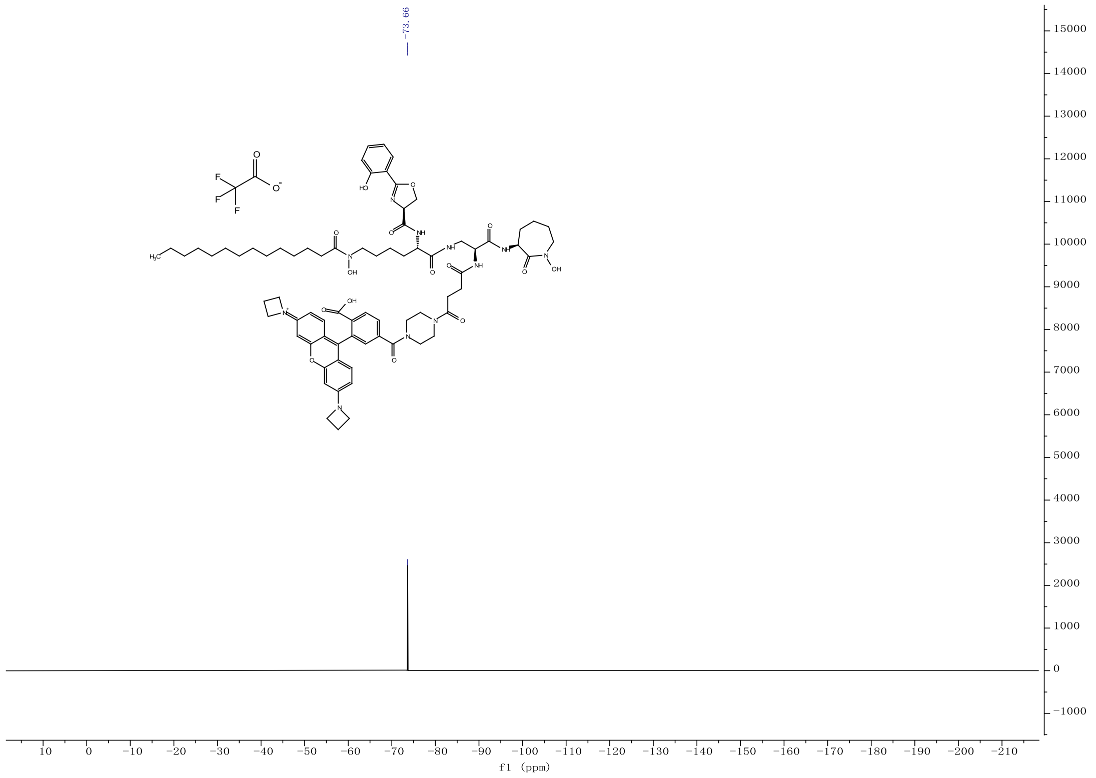

^{13}C NMR spectra of **N14L**

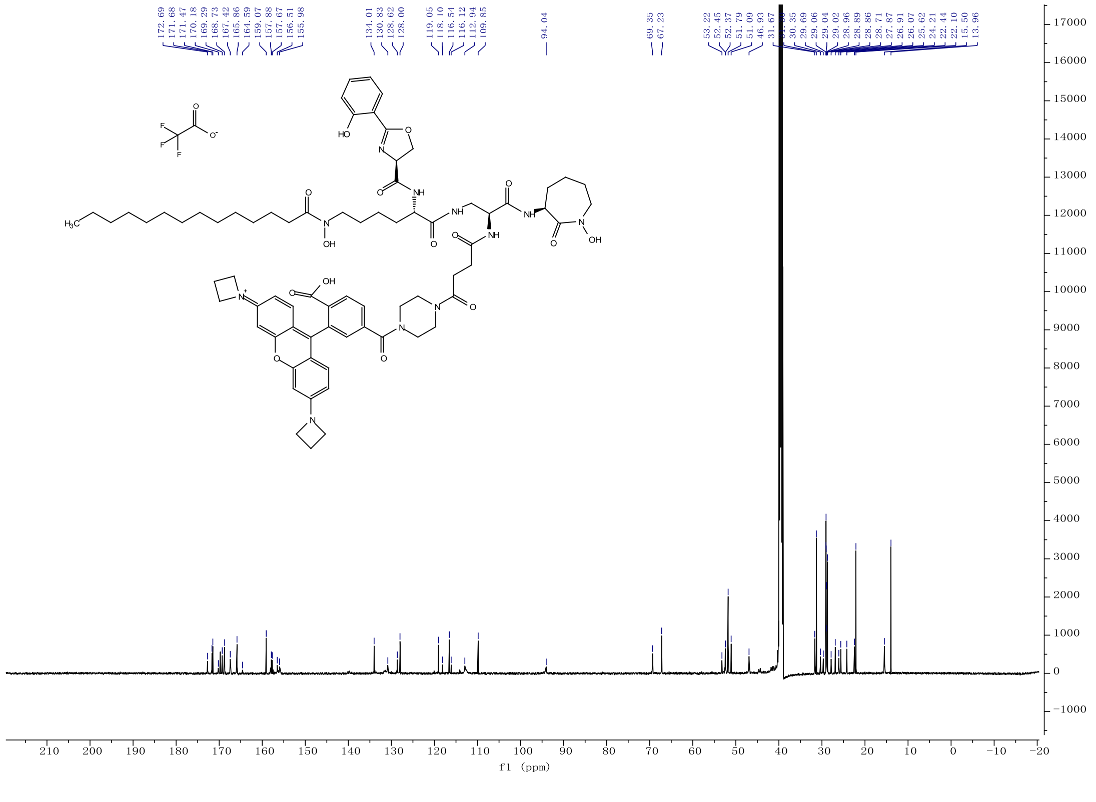

^{1}H NMR spectra of **CAR-1**

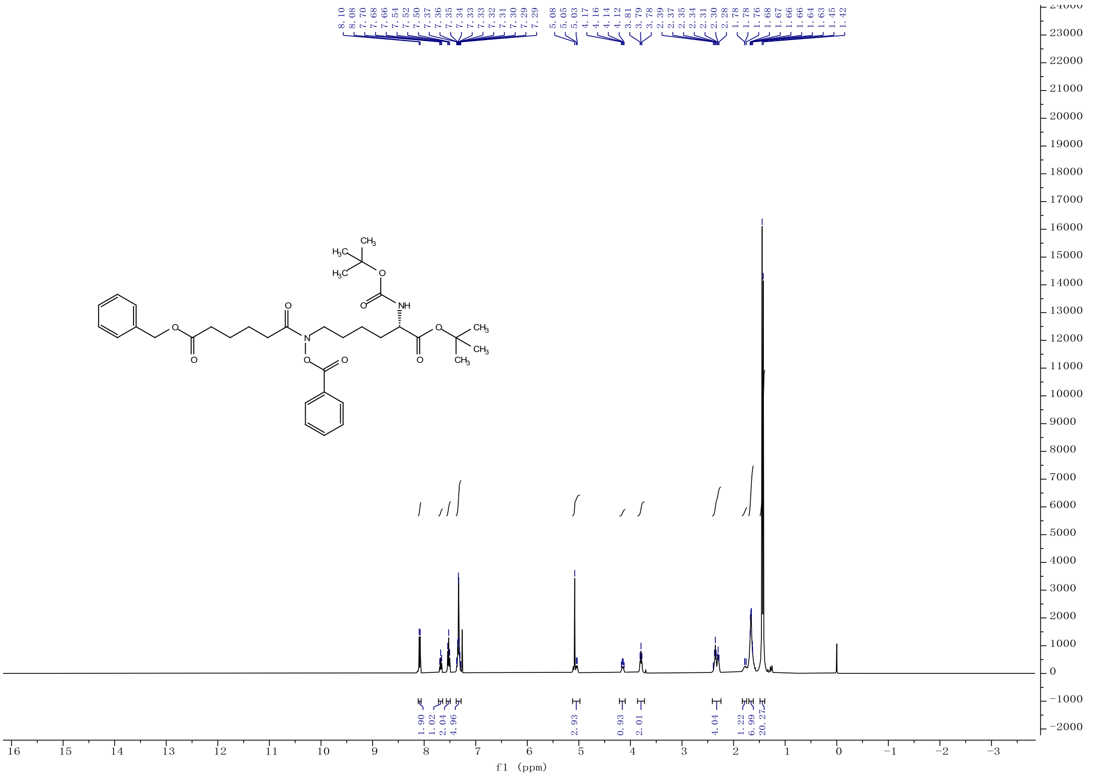

^{13}C NMR spectra of **CAR-1**

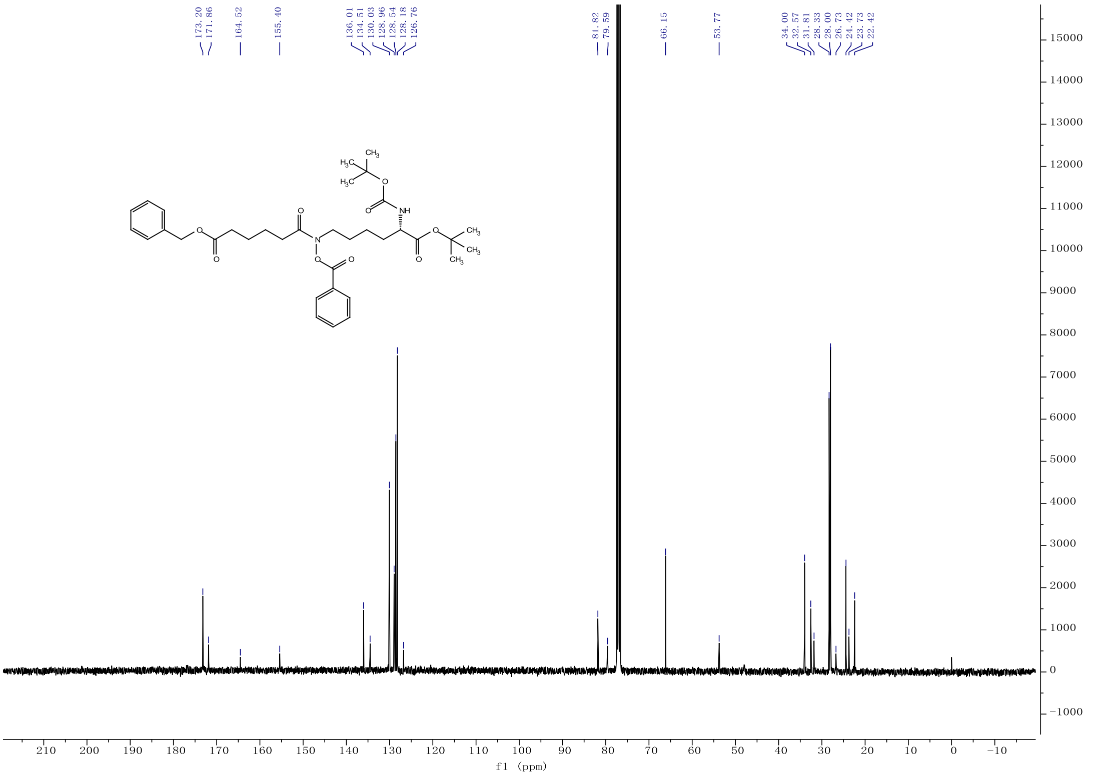

^{1}H NMR spectra of **CAR-2**

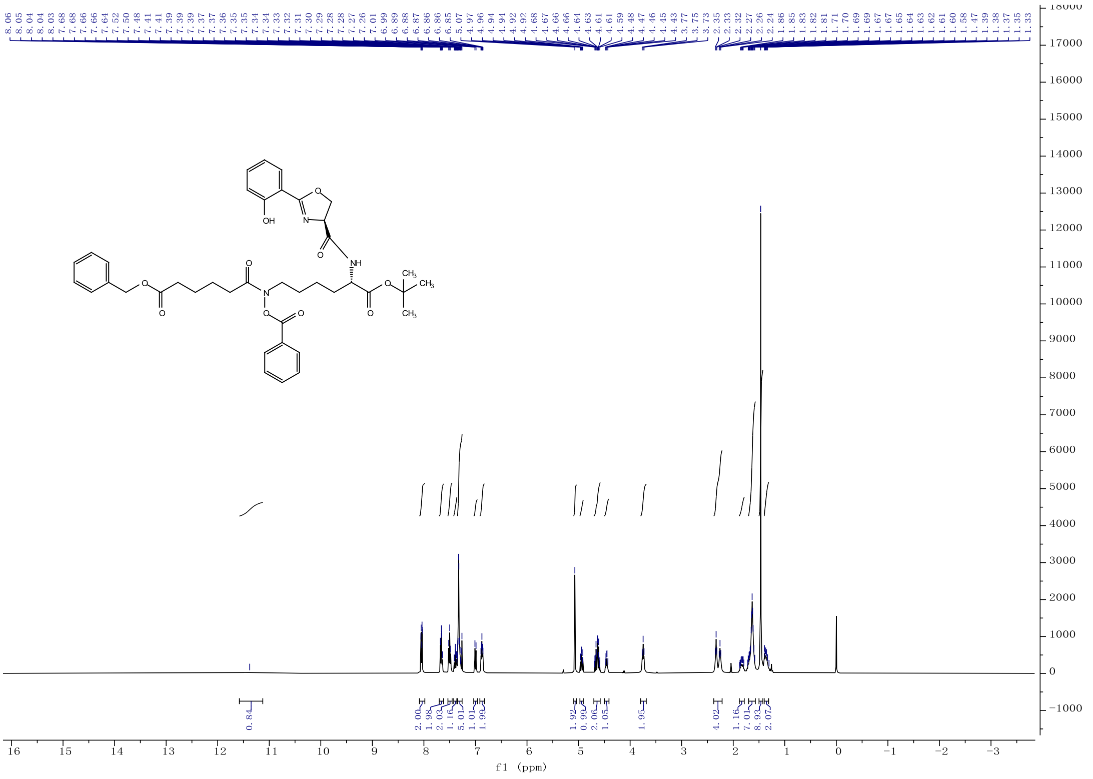

^{13}C NMR spectra of **CAR-2**

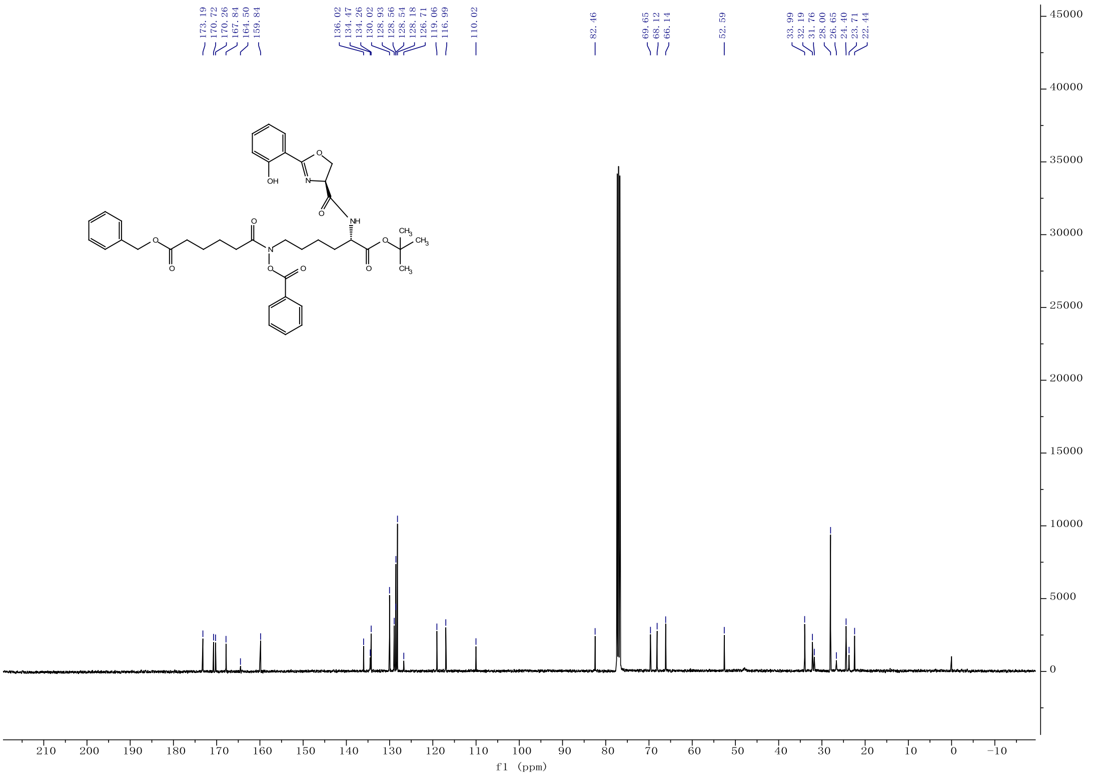

^{1}H NMR spectra of **CAR-3**

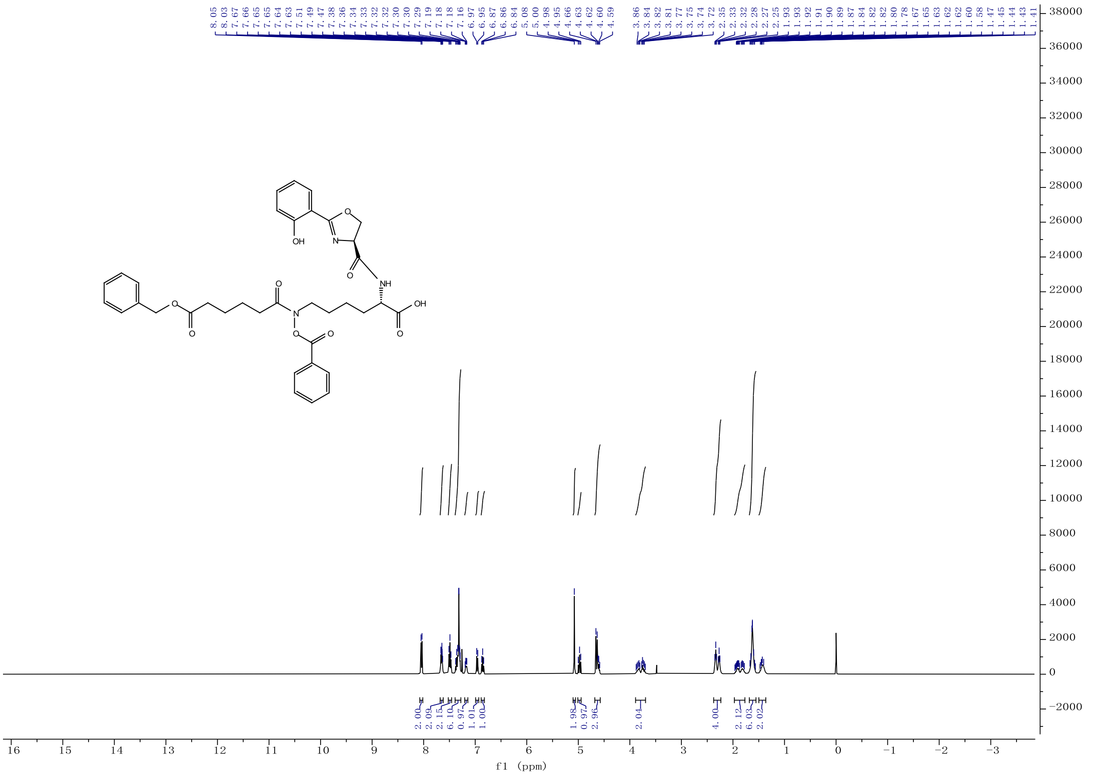

^{13}C NMR spectra of **CAR-3**

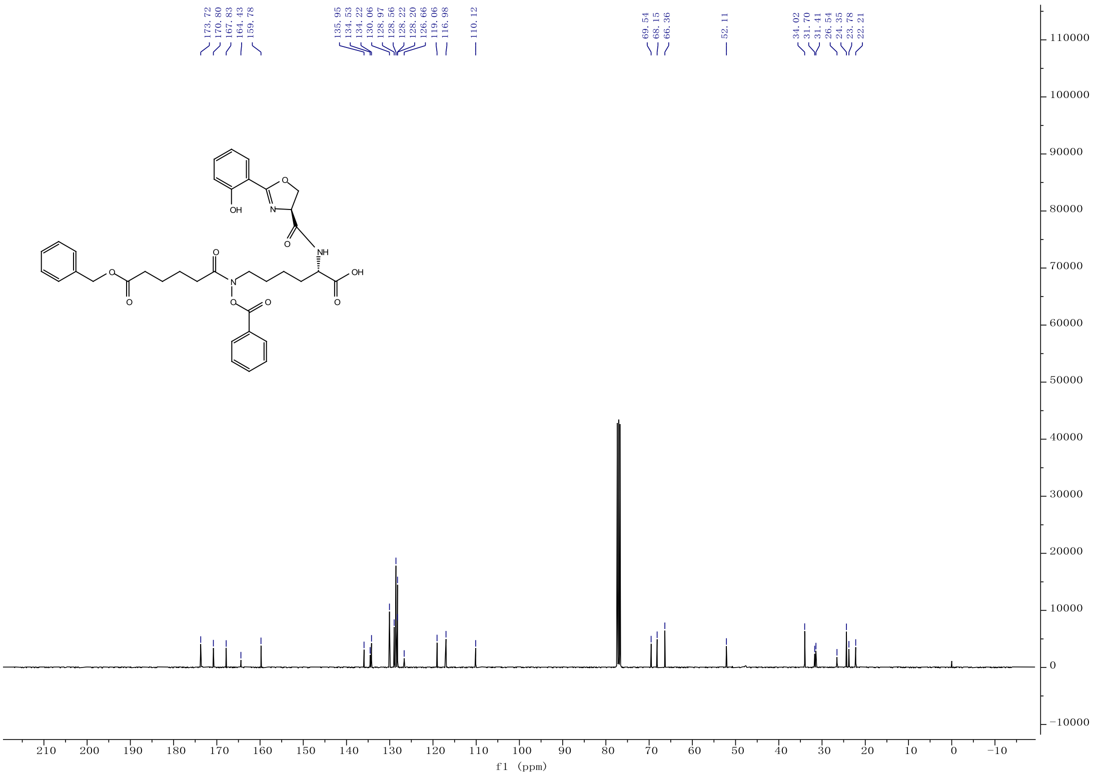

^{1}H NMR spectra of **CAR-4**

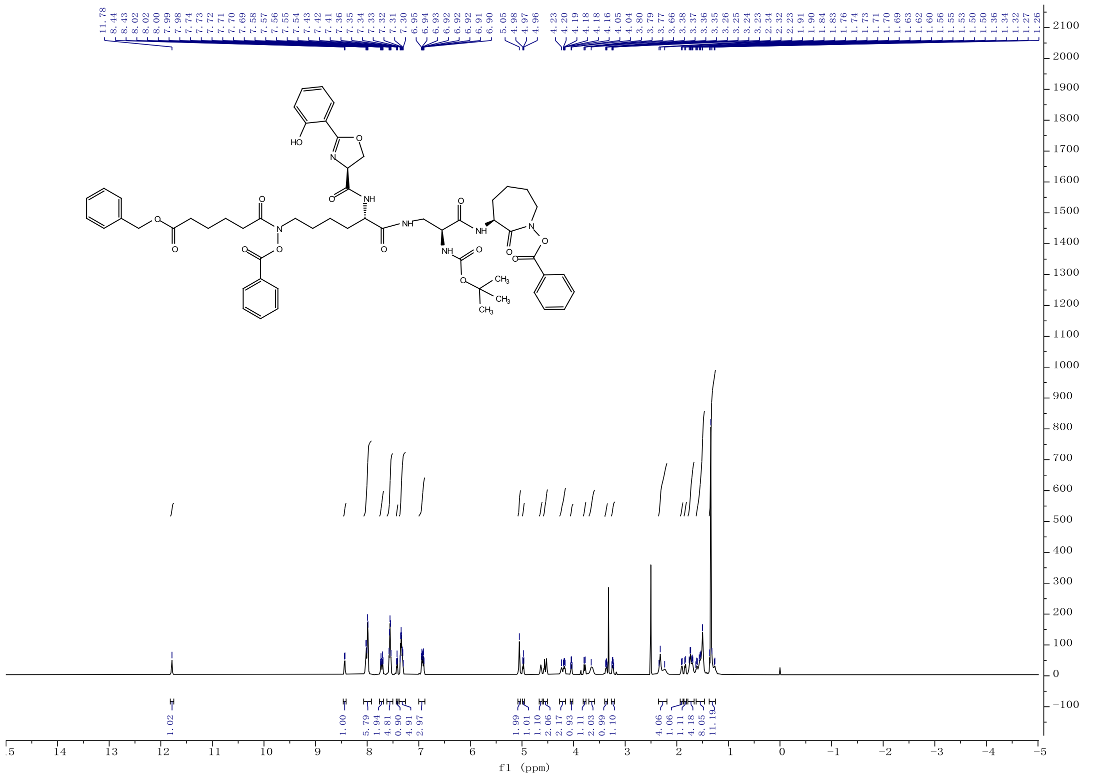

^{13}C NMR spectra of **CAR-4**

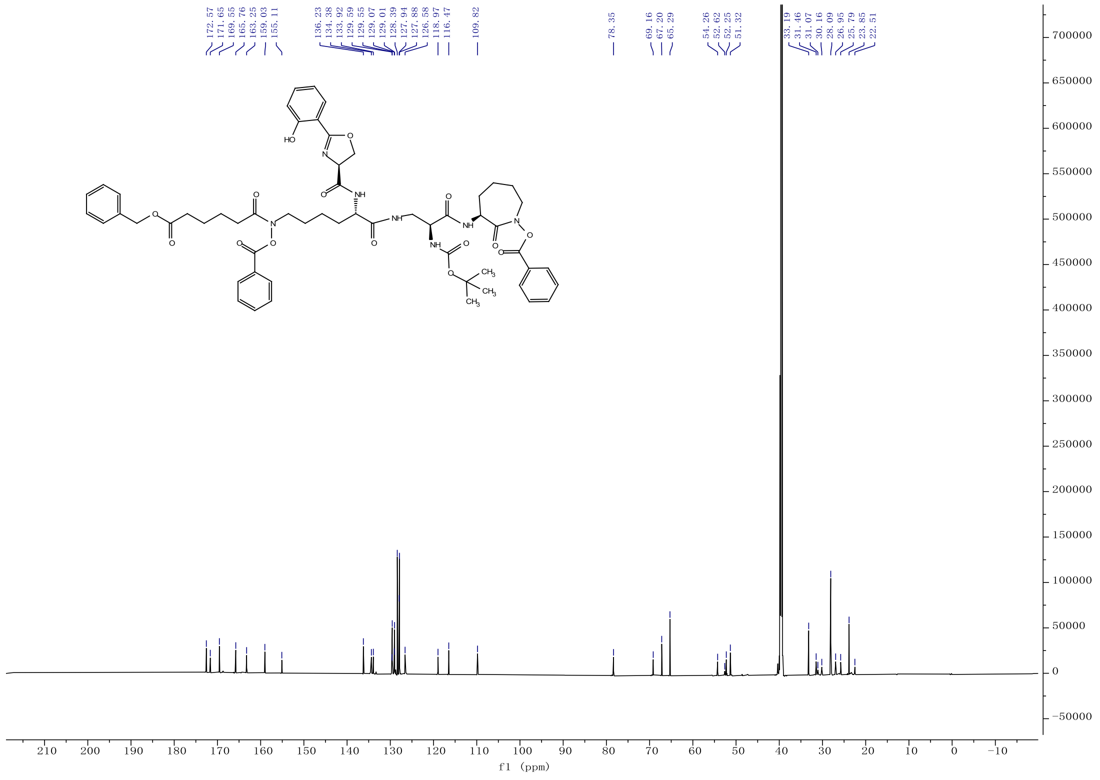

^{1}H NMR spectra of **cMbT**

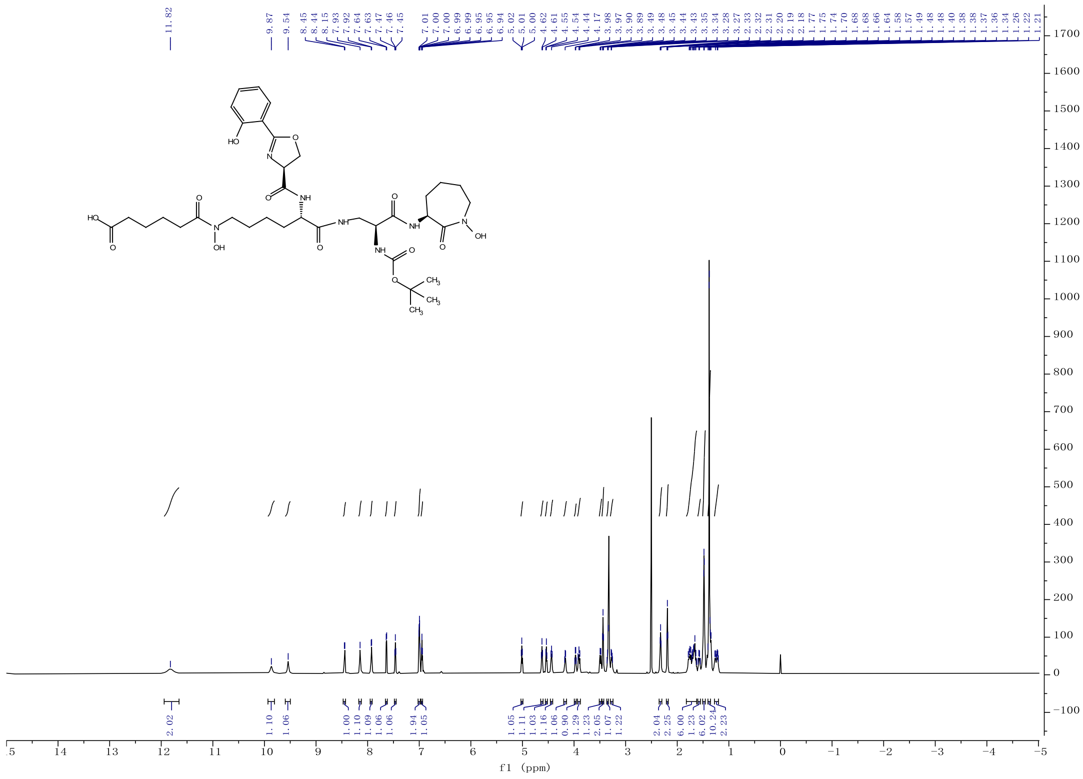

^{13}C NMR spectra of **cMbT**

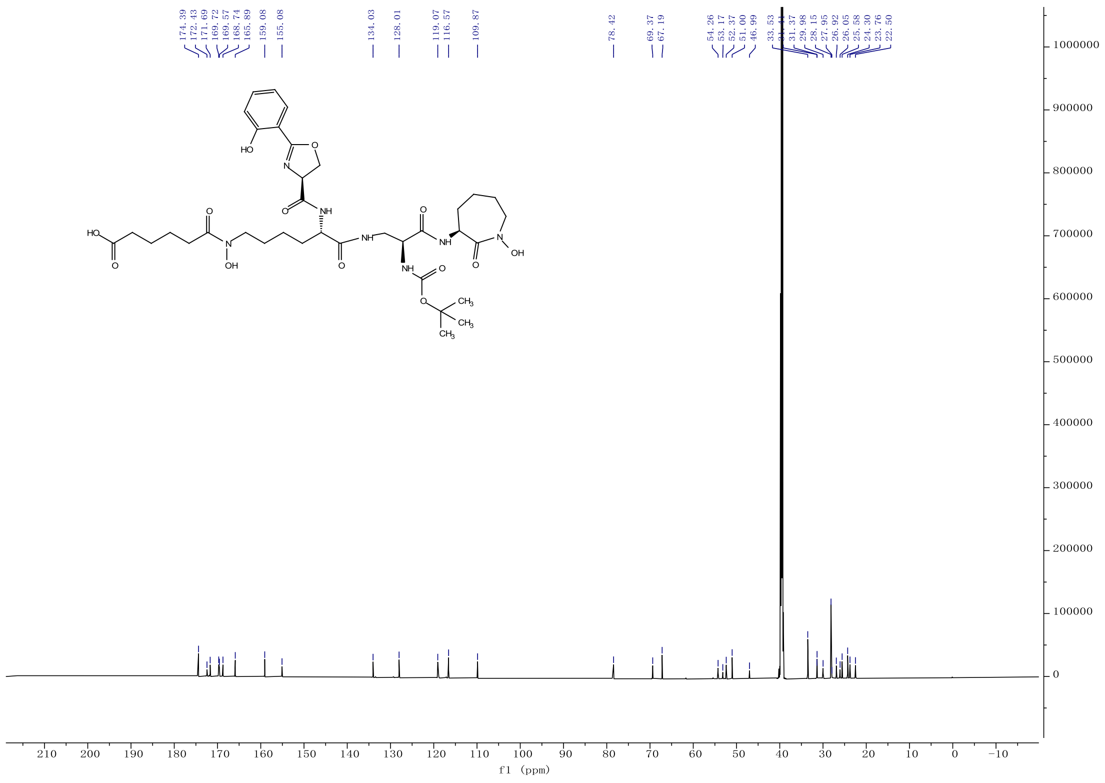

^{1}H NMR spectra of **N14G-Bz**

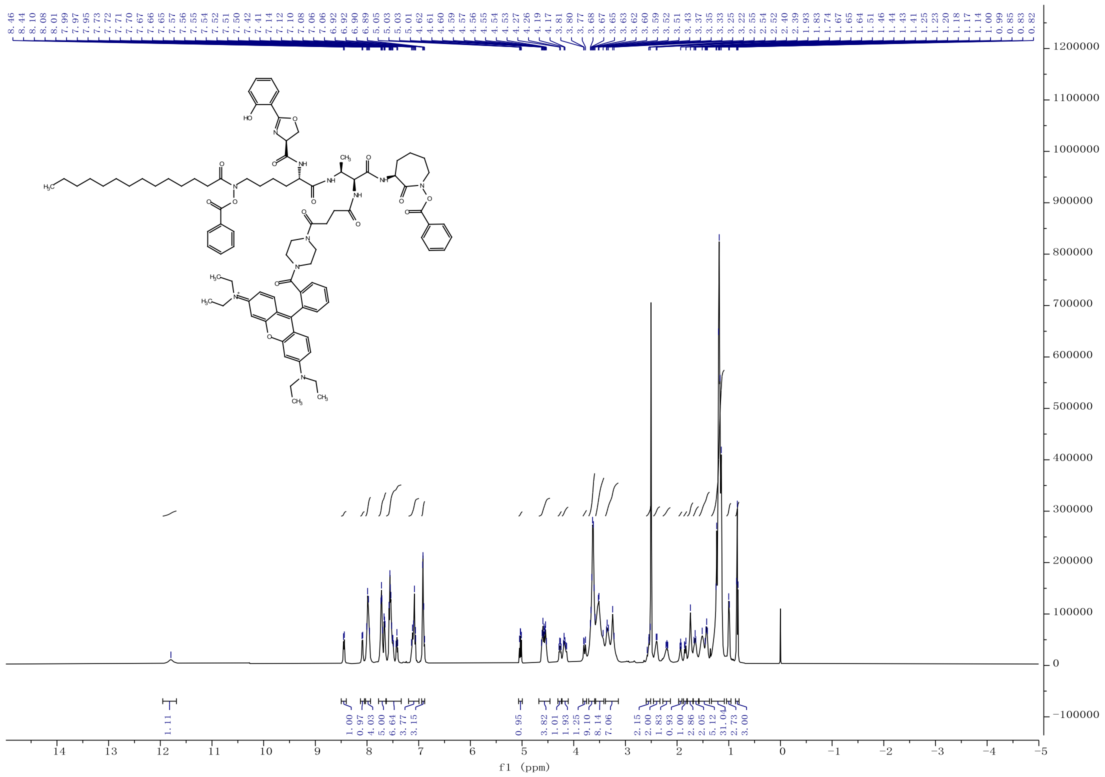

^{13}C NMR spectra of **N14G-Bz**

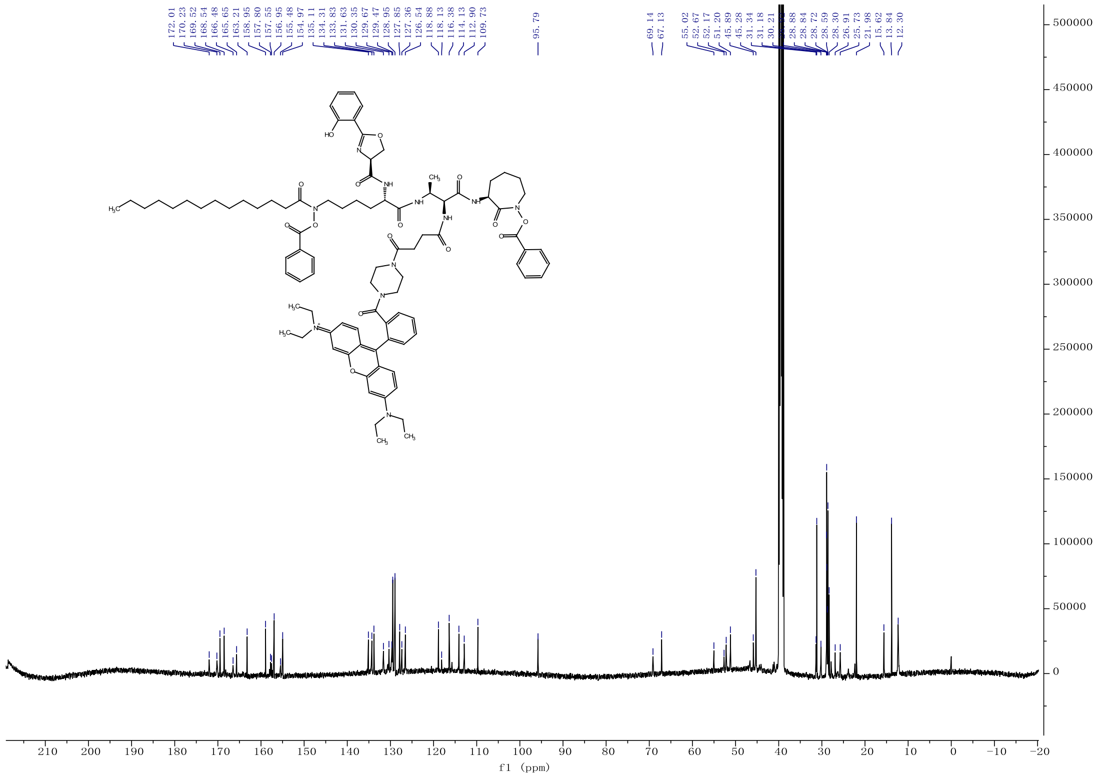

References

(1) Wu, J.; Mu, R.; Liu, Z.-J.; Lu, S.-C.; Liu, G. Scalable total synthesis of a mycobactin T analogue utilizing a novel synthetic and protection strategy. *Org. Chem. Front.* **2019**, *6* (14), 2467-2470.

(2) Sun, Z.; Shang, Z.; Forelli, N.; Po, K. H. L.; Chen, S.; Brady, S. F.; Li, X. Total Synthesis of Malacidin A by β-Hydroxyaspartic Acid Ligation-Mediated Cyclization and Absolute Structure Establishment. *Angew. Chem., Int. Ed. Engl.* **2020**, *59* (45), 19868-19872.

(3) Hong, X.; Geng, P.; Tian, N.; Li, X.; Gao, M.; Nie, L.; Sun, Z.; Liu, G. From bench to clinic: a nitroreductase Rv3368c-responsive cyanine-based probe for the specific detection of live *Mycobacterium tuberculosis*. *Anal. Chem.* **2024**, *96* (4), 1576-1586.

(4) Nguyen, T.; Francis, M. B. Practical synthetic route to functionalized rhodamine dyes. *Org. Lett.* **2003**, *5* (18), 3245-3248.

(5) Grimm, J. B.; English, B. P.; Chen, J.; Slaughter, J. P.; Zhang, Z.; Revyakin, A.; Patel, R.; Macklin, J. J.; Normanno, D.; Singer, R. H.; et al. A general method to improve fluorophores for live-cell and single-molecule microscopy. *Nat. Methods* **2015**, *12* (3), 244-250.

(6) Outzen, L.; Münzmay, M.; Frangioni, J. V.; Maison, W. Synthesis of modular desferrioxamine analogues and evaluation of zwitterionic derivatives for zirconium complexation. *ChemMedChem* **2023**, *18* (13), e202300112.

(7) Baruah, P.; Moorthy, H.; Ramesh, M.; Padhi, D.; Govindaraju, T. A natural polyphenol activates and enhances GPX4 to mitigate amyloid-β induced ferroptosis in Alzheimer's disease. *Chem. Sci.* **2023**, *14* (35), 9427-9438.
